# Low‐Concentration Electrolytes Based on Weakly Coordinating Anions for Applications in Lithium‐Ion‐Batteries and Lithium‐Metal‐Batteries

**DOI:** 10.1002/anie.202523246

**Published:** 2025-12-23

**Authors:** Stephan Burger, Katharina Tölke, Hendrik Koger, Noah Schmidt‐Meinzer, Antoine Barthélemy, Harald Scherer, Torsten Remmler, Berthold Hoge, Ingo Krossing

**Affiliations:** ^1^ Institute for Inorganic and Analytical Chemistry University of Freiburg Albertstr. 21 D‐79104 Freiburg im Breisgau Germany; ^2^ Inorganic Chemistry II University of Bielefeld Universitätsstraße 25 D‐33615 Bielefeld Germany; ^3^ Freiburg Materials Research Center (FMF) University of Freiburg Stefan‐Meier‐Str. 21 D‐79104 Freiburg im Breisgau Germany; ^4^ Freiburg Center for Interactive Materials and Bioinspired Technologies (FIT) University of Freiburg Georges‐Koehler‐Allee 105 D‐79110 Freiburg im Breisgau Germany; ^5^ Cluster of Excellence *liv*MatS University of Freiburg Georges‐Köhler‐Allee 105 D‐79110 Freiburg Germany; ^6^ NETZSCH‐Gerätebau GmbH Wittelsbacherstraße 42 D‐95100 Selb Germany

**Keywords:** Artificial Solid Electrolyte Interphase, Full Cell, Low‐Concentration Electrolyte, Lithium‐Ion Battery, Lithium Metal Anode, NCM Cathode Active Material

## Abstract

0.2 M Low Concentration Electrolytes (LCEs) for lithium‐based batteries formed from lithium salts with very weakly coordinating anions, i.e., the aluminate Li[Al{OC(CF_3_)_3_}_4_] and the gallate Li[Ga(C_2_F_5_)_4_] in *ortho*‐difluorobenzene (*o*‐DFB), showed competitive conductivity to classical electrolytes of up to 5.0 mS cm^−1^ at 25 °C combined with electrochemical stability at least up to 4.5 V vs. Li/Li^+^. Given that a stoichiometric amount of 2 equivalents dimethoxyethane (DME) per lithium ion (as Li^+^ complexing agent) and 2 wt.% fluoroethylene carbonate (as solid electrolyte interphase (SEI) former) were present in the LCEs, half and full‐cell measurements confirmed stable LCE cycling over 300 cycles in Lithium‐Ion‐Batteries. Even at high currents (5C), the discharge retained two thirds of the practical 1C capacities of NMC622. By contrast, a LCE made from 0.2 M LiPF_6_ in EC/EMC 3:7 solution already led at a 2C rate to cell death, while a simple switch of the conducting salt to 0.2 M Li[Al{OC(CF_3_)_3_}_4_] led to stable cycling including rate tests for over 300 cycles and approached closely the values of the standard 1.0 M LiPF_6_ electrolyte in EC/EMC 3:7 – attributed to the anions’ stability. The performance of the aluminate LCE was further evaluated in symmetrical Li‐Li cells and Lithium‐Metal‐Batteries containing 48 µm thin Lithium‐Metal‐Anodes (LMAs): LCEs improved the cell's lifetime by a factor of 3–6 at a current density of 1 mA cm^−2^. Scanning electron microscope/energy‐dispersive X‐ray and potentiostatic electrochemical impedance spectroscopy measurements confirmed the exceptional stabilization of the LMAs by the aluminate LCE throughout the cycling, especially when combined with an artificial, adaptive and self‐healing SEI based on Li[PO_2_(OCH_2_CF_3_)_2_]. The solvation structure of standard and LCEs was investigated by NMR spectroscopic diffusion measurements and quantum chemical calculations. A three‐to‐fourfold increased Li ion mobility was found in LCEs compared to the system with 0.2 M LiPF_6_ in standard carbonate solution. The presence of stable and compact Li(DME)_2_^+^ structures as moving ions was shown and the relevance of Li^+^ ions solvated with fluoro‐ethylene carbonate or *o*‐DFB for SEI‐formation is discussed.

## Graphical abstract

Big is beautiful? The performance of 0.2 M low concentration lithium electrolytes with large weakly coordinating anions [Al{OC(CF_3_)_3_}_4_]^−^ and [Ga(C_2_F_5_)_4_]^−^ in low viscosity ortho‐difluorobenzene solution is tested in comparison to standard 1.0 M as well as diluted 0.2 M carbonate‐based electrolytes with Li[PF_6_] conducting salt. Lithium ion / lithium metal batteries were tested symmetric, half, and full cells.

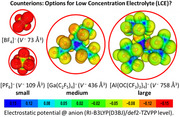

## Introduction

The demanding requirements of automotive applications have driven the search for high performance lithium‐ion batteries (LIBs) with the goal of reducing greenhouse gas emissions in the mobility sector.^[^ ^1^, ^2^, ^3^ ^]^ With the intensive efforts of the research community and industry, their specific energy has been tripled over the last decades.^[^ ^4^, ^5^ ^]^ Hence, the state‐of‐the‐art mainly uses graphite anodes and cathode active materials like LiNi_x_Mn_y_Co_1‐x‐y_O_2_ (NMCs) (*x* > 0.6) and lithium‐iron‐phosphate (LFP).^[^ ^6^, ^7^, ^8^, ^9^ ^]^

Lithium‐ion battery (LIB)‐electrolyte solutions typically consist of about 1 M solutions of lithium hexafluorophosphate (LiPF_6_) as conducting salt in a solvent mixture of cyclic (e.g., ethylene carbonate EC) and linear carbonates (e.g., dimethyl carbonate DMC), including various additives.^[^ ^10^, ^11^ ^]^ The overall composition has not changed much since the commercialization of LIBs in 1991 and typically includes the very polar, but viscous and high‐melting EC as polarity donor and a low dielectric, low viscosity thinner like DMC. In addition, the beneficial electrochemical decomposition of EC at the graphite anode within the initial cycles forms a stable Li‐ion conductive solid electrolyte interphase (SEI) that strongly mitigates the ongoing further electrolyte decomposition.^[^ ^12^, ^13^, ^14^ ^]^ Due to this superior performance of the carbonate mixtures in LIBs, research efforts have been mainly restricted to the development of new additives.^[^ ^15^ ^]^

By contrast, the introduction of secondary lithium‐metal batteries (LMBs) into the mass market has failed since the 1970s due to the highly reactive inherent properties of lithium metal that lead to an unstable electrode/electrolyte interface/interphase at the lithium‐metal anode (LMA) in carbonate‐based electrolytes.^[^ ^16^, ^17^, ^18^ ^]^ Several strategies^[^ ^19^ ^]^ have been investigated to overcome these issues: highly concentrated electrolytes (HCE), localized highly concentrated electrolytes (LHCE), and weakly solvating electrolytes (WSE),^[^ ^20^, ^21^ ^]^ but the ideal stabilization strategy and their corresponding electrolytes^[^ ^18^, ^22^, ^23^ ^]^ have yet to be identified for LMBs.

**From Alternative Conducting Salts to Low Concentration Electrolytes**: Although LiPF_6_ as conducting salt offers the best compromise regarding conductivity, electrochemical stability, and economic issues, its thermal instability and overall sensitivity against moisture can initiate the formation of highly toxic, gaseous, and reactive decomposition products such as hydrogen fluoride (HF), phosphorus pentafluoride (PF_5_), and phosphoryl fluoride (POF_3_), which pose a serious hazard in case of cell failure.^[^ ^12^, ^24^, ^25^ ^]^ Thus, the search for alternative electrolyte systems has gained renewed interest. Yet, practically all well‐functioning alternatives to LiPF_6_ as a conducting salt^[^ ^26^, ^27^, ^28^ ^]^ are considerably more expensive. However, when investigating the ion‐pairing^[^ ^29^, ^30^ ^]^ and ionicity^[^ ^31^ ^]^ of commercial carbonate based LiPF_6_ electrolytes, one realizes that only from 30% to 47% of the Li^+^ ions are active in transport, while the rest is part of ion‐pairs or ion‐tuples contributing minor to charge transfer. Ion‐pairing with Li^+^ is a function of the counter anions’ quality as weakly coordinating anion (WCA): Better WCAs should promote dissociation and hence induce very high Li^+^ ion activity.^[^ ^32^, ^33^ ^]^ Thus, low concentration electrolytes (LCEs)^[^ ^34^, ^35^, ^36^, ^37^, ^38^, ^39^, ^40^ ^]^ with conducting Li salt concentrations considerably lower than the 1.0…1.5 M standard might be accessible and also affordable, given use of suitable LiWCA salts, a topic that gained a lot of recent interest.^[^ ^41^ ^]^

**Scheme 1:**
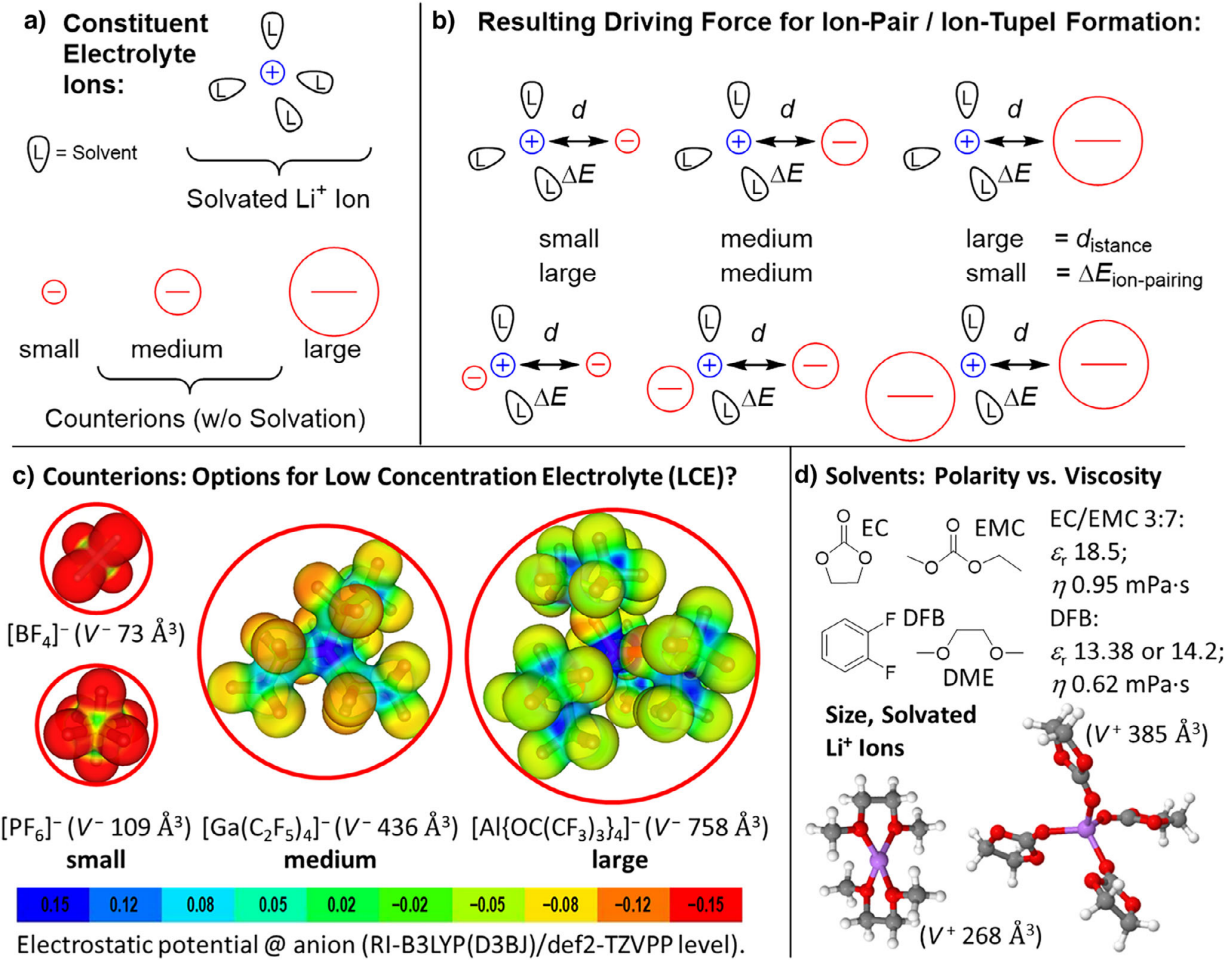
a) Schematically shown constituent electrolyte ions for Li‐based batteries. b) Exemplarily sketched effect of the counter anion size on the tendency to form ion‐pairs or ion‐tuples. c) Molecular structures and sizes *V* ^−^ of [BF_4_]^−^, [PF_6_]^−^, [Ga(C_2_F_5_)_4_]^−^, and [Al{OC(CF_3_)_3_}_4_]^−^ in Å^3^, including the projection of the calculated electrostatic potential onto a 0.025 e^−^ Bohr^−3^ isodensity surface of [BF_4_]^−^, [PF_6_]^−^, [Ga(C_2_F_5_)_4_]^−^, and [Al{OC(CF_3_)_3_}_4_]^−^. Red: accumulation of negative charge; green: neutral; blue: positively charged surface regions (see scale bar). d) Permittivity *ε*_r_ and dynamic viscosity *η* of the pure standard carbonate solvent mixture L57 = EC:EMC 3:7 (in wt.‐%) as well as the pure *o*‐DFB solvent.^[^ ^44^ ^]^ In addition, the DFT calculated structures and cation volumes *V* ^+^ of the probably dominant migrating Li^+^ solvate cations are shown (in Å^3^). All calculations were performed at the RI‐B3LYP/def2‐TZVPP level of theory.

**Scope of this work**: We investigate the air‐ and moisture‐stable LiWCA salts of the aluminate^[^ ^42^ ^]^ [Al{OC(CF_3_)_3_}_4_]^−^ and gallate^[^ ^43^ ^]^ [Ga(C_2_F_5_)_4_]^−^ as conducting salts for 0.2 M LCEs in the low‐viscosity, but polar^[^ ^44^ ^]^ solvent *o*‐difluorobenzene (*o*‐DFB).^[^ ^33^, ^45^, ^46^ ^]^ Scheme 1a–d shows the working hypotheses for this investigation: Li^+^ ion transport functions through mobility of solvated Li^+^ ions, which also is a function of the size and quality of the counter anion. Hence, larger and better WCAs should increase Li^+^ ion activity and mobility by a reduced tendency to form ion‐pairs or ion‐tuples (Scheme 1a and b). The size of the counterions,^[^ ^46^, ^47^ ^]^ given in Å^3^, as well as DFT calculated electrostatic potential plots of [Al{OC(CF_3_)_3_}_4_]^−^ and [Ga(C_2_F_5_)_4_]^−^ in comparison to [PF_6_]^−^ (Scheme 1c), highlight the different charge distribution of the anions and suggest a lower basicity of the gallate and aluminate anions with respect to [PF_6_]^−^ or the even smaller [BF_4_]^−^ included for completion. Hence, the aluminate and gallate should also enable Li^+^ ion mobility in a low viscosity, but less polar solvent such as *o*‐DFB (Scheme 1d), given that a stoichiometric amount of 2 equivalents of dimethoxyethane (DME) per Li^+^ ion is present as complexing agent. Note, that the compact Li(DME)_2_^+^ ion with a calculated cation volume *V* ^+^ of 268 Å^3^ is considerably smaller than the exemplary Li(EC)_4_^+^ ion with *V* ^+^ of 385 Å^3^ shown in Scheme 1d. Hence, the small size of the Li(DME)_2_^+^ ion should enhance its mobility compared to classical carbonate solutions. Note that ethers coordinated to Li^+^ are compatible with battery cycling also at higher potentials.^[^ ^31^ ^]^ Only free DME starts to degrade at potentials higher than 4.2 V vs. Li^+^/Li. Therefore, a stoichiometric amount of 2 DME molecules per Li^+^ cation was used. Since DME does not form stable and protective secondary interphases,^[^ ^48^, ^49^ ^]^ 2 wt.% of fluoroethylene carbonate (FEC) were added as an SEI forming agent.^[^ ^50^, ^51^, ^52^ ^]^ Yet, *o*‐DFB or other fluorobenzenes used as a cosolvent in lithium‐metal batteries (LMBs) were also reported to form a LiF‐rich SEI.^[^ ^53^, ^54^, ^55^ ^]^

Overall, the composition and nomenclature of the electrolytes considered in this study is summarized in Table 1, including some fundamental physicochemical electrolyte data determined in this study.

**Table 1:** Overview of the physicochemical properties and the composition of the electrolyte systems used in this work: Conductivity *σ*, viscosity *η*, and concentration *c*.

			Composition of the electrolyte			
Electrolyte Systems / Acronym used	*σ* / mS cm^−1^ 298 K	*η* / mPa·s 298 K	*c* in mol L^−1^ / conducting salt	Solvent	Co‐Solvent (Eq. per Li^+^)	Additive (wt.%)
**LP57** **^a^** **^)^**	8.15	3.4	**1.0** / LiPF_6_	L57^b)^	−	−
**DFB02_Aluminate**	5.03	0.86	**0.2** / Li[Al{OC(CF_3_)_3_}_4_]	*o*‐DFB	DME (2)	FEC (2)
**DFB02_Gallate**	4.09	0.87	**0.2** / [Li(DEC)_2_][Ga(C_2_F_5_)_4_]	*o*‐DFB	DME (2)	FEC (2)
**LiPF_6_ / L57** **^b)^**	3.79	1.3	**0.2** / LiPF_6_	L57^b)^	−	−
**Li[Al{OC(CF_3_)_3_}_4_] / L57** **^b)^**	2.24	1.7	**0.2** / Li[Al{OC(CF_3_)_3_}_4_]	L57^b)^	−	−

^a)^ LP57 = 1.0 M solution of LiPF_6_ in EC/EMC 3:7 (wt.%)

^b)^ L57 = solvent mixture EC/EMC 3:7 (wt.%).

Hence, the DFB02 LCE electrolytes in Table 1 were evaluated in comparison to the other systems in half‐ and full‐cells with NMC cathodes including long‐term cycling and rate‐performance tests. The performance of the aluminate LCE was investigated in Li‐Li symmetrical cells and realistic LMBs with thin (48 µm) LMAs, in part protected by the artificial LiBFEP‐SEI (BFEP^−^ = [PO_2_(OCH_2_CF_3_)_2_]^−^).^[^ ^56^, ^57^ ^]^ Finally, the solvation structures and electrolyte properties were evaluated by temperature dependent viscosity and conductivity measurements, multinuclear NMR, Pulsed‐Gradient Spin Echo PSTE‐NMR experiments and quantum chemical calculations.

## Results and Discussion

### Physicochemical Characterization of the LCEs

**Figure 1:**
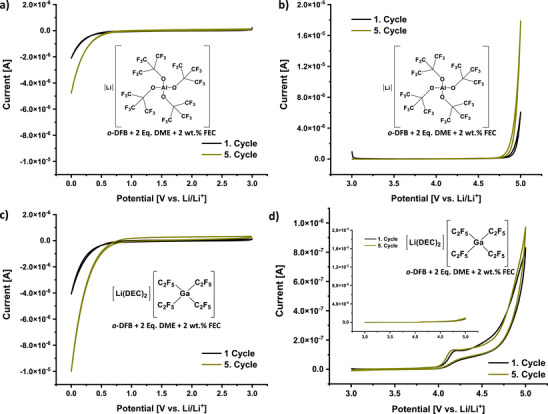
Cyclic voltammetric measurements of the DFB02‐LCEs: a, b) 0.2 M solution of Li[Al{OC(CF_3_)_3_}_4_] in *o*‐DFB + 2 Eq. DME + 2 wt.% FEC (DFB02_Aluminate) and c, d) 0.2 M solution of [Li(DEC)_2_][Ga(C_2_F_5_)_4_] in *o*‐DFB + 2 Eq. DME + 2 wt.% FEC (DFB02_Gallate). For the measurements in a,c) a glassy carbon electrode was used (0 − 3 V), while a platinum electrode was used in (b and d) (3–5 V). The plot in d) contains an inset with identical scaling as in (b). All CV experiments were carried out with a lithium counter and reference electrode. The scan rate was set to 10 mV s^−1^ at 25 °C. With active electrode areas of 0.785 mm^2^, currents below 1x10^−6^ A in the electrolyte window are to our experience typically very well usable for battery work.

Figure 1 shows cyclic voltammetric (CV) measurements of the 0.2 M DFB02‐LCEs containing Li[Al{OC(CF_3_)_3_}_4_],^[^ ^42^ ^]^ also commercially available,^[^ ^58^ ^]^ and [Li(DEC)_2_][Ga(C_2_F_5_)_4_]^[^ ^43^ ^]^ as conducting salts (DEC = diethylcarbonate). The CV measurements in Figure 1a–d show the absence of any relevant oxidative current at potentials <4.5 V vs. Li/Li^+^ for the Li[Al{OC(CF_3_)_3_}_4_] conducting salt. Moderate reductive currents with an onset at potentials <0.9 V vs. Li/Li^+^ are assigned to FEC decomposition.

Only at very low currents, the DFB02_Gallate LCE shows small oxidative signatures at potentials >4.1 V vs. Li/Li^+^. However, when the overall currents are plotted on the same scale as those in Figure 1b (inset in d), the performance of the two electrolyte salts is comparable, as are the CVs at 0–3 V vs. Li/Li^+^. Hence, both 0.2 M DFB02 LCEs enable sufficient stability in the potential range of classical LIBs typically operating between 0.01–4.3 V vs. Li/Li^+^.

**Figure 2:**
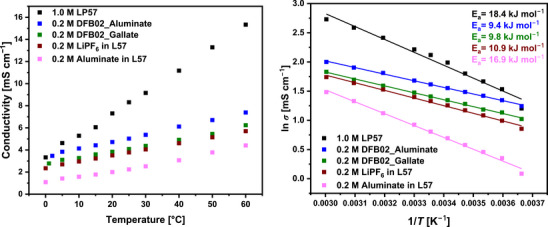
Temperature‐dependent conductivity measurements of the 0.2 M DFB02 and LiPF_6_/L57 LCEs compared to the 1.0 M LP57 reference electrolyte between 0 − 60 °C. On the left as a regular plot and on the right as Arrhenius‐plot including the activation energies for the ionic motion.

**Conductivities**: Figure 2 shows the regular temperature‐dependent conductivities *σ* as well as the Arrhenius plots of the 0.2 M DFB02_Aluminate and DFB02_Gallate LCEs in comparison to the 1 M solution of LiPF_6_ in ethylene carbonate/ethyl methyl carbonate 3:7 (by wt.%; LP57), which is used in commercial applications. In addition, the activation energies for the ionic motion *E*_a_ in the electrolyte solutions determined from the Arrhenius plots are included with Figure 2, right.

The conductivity of the commercially used 1.0 M LP57 electrolyte reaches 8.15 mS cm^−1^ at room temperature (25 °C).^[^ ^59^, ^60^ ^]^ Fortunately, the 0.2 M LCEs with only one‐fifth of the ions still ensure at 25 °C a conductivity of 5.03 mS cm^−1^ (Li[Al{OC(CF_3_)_3_}_4_], 62% of LP57), and 4.09 mS cm^−1^ ([Li(DEC)_2_][Ga(C_2_F_5_)_4_], 50% of LP57). All 0.2 M solutions in the carbonate blend L57 (L57 = ethylene carbonate/ethyl methyl carbonate 3:7 wt.%) are inferior to the *o*‐DFB‐based LCEs (Table 1, Figure 2). This agrees with the derivation of the activation energy for ionic motion *E*_a_ according to Arrhenius theory in Figure 2: The DFB02 electrolytes do have the lowest *E*_a_‐values of below 10 kJ mol^−1^, while that of LP57 is the highest (18.4 kJ mol^−1^). Comparison of the two *E*_a_ values of 0.2 M LiPF_6_ in L57 and 0.2 M Li[Al(OR^F^)_4_] in L57, shows that the much larger [Al{OC(CF_3_)_3_}_4_]^−^ counterion induces higher activation energies at like concentration in the same solvent (10.9 versus 16.9 kJ mol^−1^), but and despite the much larger anion sizes (Scheme 1), the activation energies for ionic motion in the DFB02 electrolyte (9.4 and 9.8 kJ mol^−1^) are still lower than that of 0.2 M LiPF_6_ in L57 (10.9 kJ mol^−1^). Thus, Figure 2 implies an improved mobility of the Li ions in the 0.2 M DFB02‐electrolytes, probably – and apart from viscosity effects investigated in the next section – induced by the less interacting WCAs and the small [Li(DME)_2_]^+[^ ^48^ ^]^ entities, if compared to the larger [Li(carbonate)_4–6_]^+^ ions present in L57^[^ ^61^, ^62^ ^]^ (cf. diffusion‐NMR / quantum chemical calculations in Section 4 / 6).

**Figure 3:**
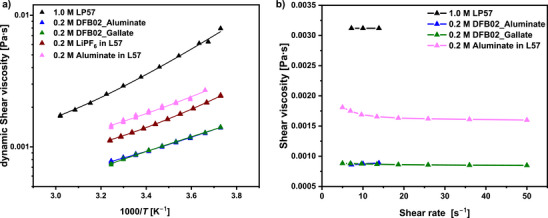
a) Arrhenius plot of the dynamic shear viscosity *η* for the electrolyte solutions measured between 268 and 308 K and showing the Arrhenius behavior. b) Viscosities at different shear rates at 298 K, suggesting Newtonian behavior.

Figure 3a plots the viscosities *η* of the electrolyte systems between 268–308 K. All studied electrolyte systems follow Arrhenius behavior. In addition, the viscosities of the DFB02 LCEs are almost one order of magnitude lower than the reference electrolyte LP57. Since conductivity in concentrated solutions is often limited by viscosity, this is also reflected in the low activation energies *E*_a_ for ionic transport and is favorable for the DFB02 LCEs (see Table 1 and Figure 2).^[^ ^63^, ^64^ ^]^ The viscosity is also constant at different shear rates (Figure 3b), a characteristic of Newtonian fluids.^[^ ^65^ ^]^ The complementary fit data of the temperature‐dependent viscosity measurements of Figure 3a is deposited in Table S1.

### Testing of the Electrolyte Systems in LIB Half Cells and Full Cells

**Figure 4:**
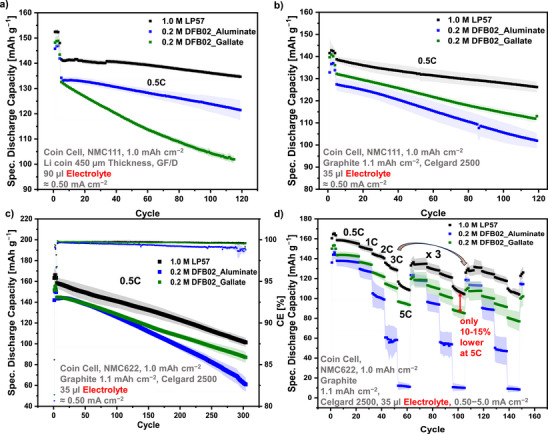
a, b) Cycling data of half and full cells at 25 °C with the specified electrolyte solutions and NMC111 cathodes at 0.5 °C. Quadruplicates were tested for each series of cells; c) Cycling data of full cells at 25 °C with the specified electrolyte solutions and NMC622 cathodes at 0.5 °C; d) full cell rate capability tests with the same electrolyte solutions at 0.5–5 °C (3 rounds with 10 rate‐cycles each). Quadruplicates were tested for each series of cells.

**Half‐Cell Cycling**: The performance of the 0.2 M DFB02_Aluminate and DFB02_Gallate LCEs in Li|NMC111 half cells compared to the LP57 reference electrolyte is shown in Figure 4a. Almost stable cycling was found for cells with 1.0 M LP57 or 0.2 M Li[Al{OC(CF_3_)_3_}_4_] electrolyte at 0.5C and with similar capacity retentions over 115 cycles: 95.2 % (141.5 → 134.7 mAh g^−1^) with LP57 and 90.5 % (134.3 → 121.5 mAh g^−1^) with DFB02_Aluminate LCE. Hence, despite the very different solvent composition and conducting salt concentration, the capacities and their retention are close for both electrolytes. In contrast, the 0.2 M gallate LCE showed an enhanced capacity decay, and after 115 cycles, only 76.7 % of the initial capacity was retained. Possibly, this could be due to an inferior compatibility of the gallate anion with the LMA, a phenomenon further investigated in section 6 / Supporting Information Section 7. To study the influence of FEC, Figure S2 shows half‐cell cycling with 2, 5, or 10 wt.% of FEC, indeed demonstrating a positive effect: Cells with 5 or 10 wt.% FEC show about 15 % better performance than the DFB02_Aluminate cells with 2 wt.% FEC. Nevertheless, in this proof‐of‐concept study, the content of FEC was kept low at 2 wt.% with the aim of achieving (almost) carbonate‐free electrolyte systems.

**NMC111 Full Cells**: Figure 4b shows the cycling data of Graphite|NMC111 full cells (in CR2032 coin cell quadruplicates) under electrolyte‐sparse conditions (35 µL) with the 0.2 M DFB02 LCEs compared to the 1.0 M LP57 reference. The cycling is almost stable for the low‐concentration electrolyte over 115 cycles at 0.5C, maintaining 80.0 % (DFB02_Aluminate) / 85.5 % (DFB02_Gallate) of the initial capacity, demonstrating the compatibility of the novel low‐concentration and low‐viscosity electrolyte systems with commercially used active materials and separators under electrolyte‐scarce conditions. This observation supports the notion of gallate incompatibility with Li metal, here mitigated by the SEI formed on the graphite electrodes. The LP57 reference cells enable capacity retention of 90.9 % under the same cycling conditions. To gain further insight, rate capability tests were conducted by applying currents between 0.5C–5C, shown in Figure S3. Although the 1.0 M LP57 reference cells perform best, cells with the 0.2 M DFB02 LCEs maintain very reasonable capacities even at a 2C rate. At 5C, the dilute gallate even retains almost 80 % of the discharge capacity compared to the 1.0 M LP57 reference.

**NMC622 Full Cells**: The measurements were repeated with NMC622 cathodes. Figure 4c shows the cycling data of full cells over 300 cycles at 0.5C, again under electrolyte sparse conditions (35 µL, CR2032 quadruplicates). The full cells with DFB02 LCEs retain 42.5 % (Aluminate) and 60.2 % (Gallate) of the initial capacity, whereas the 1.0 M LP57 reference retains 64.0 %, only slightly better than the dilute DFB02_Gallate LCE. The drop of the specific discharge capacity accelerates after 100 cycles for the DFB02_Aluminate LCE and coincides with decreasing Coulombic efficiencies (CE). The CEs of the 1.0 M LP57 reference and the DFB02_Gallate LCE are with >99 % almost stable throughout the cycling. To evaluate the capacity drop of the DFB02_Aluminate LCE, full cells with 5 wt.% FEC as additive were tested (Figure S4). This improved the capacity retention after 300 cycles to 50.4 % (cf.: 2 wt.%, 42.5 %) with also slightly improved CEs. Possibly, FEC is depleted during the later stages of the cycling process and a higher FEC content could mitigate this deficiency.

The NMC622 full cells were also investigated at C‐rates up to 5C (Figure 4d). These tests confirm the previous observations for NMC111 cathodes: the performance is inferior compared to the LP57 reference, but not as much as one could anticipate with an ion‐loading of one fifth for the LCEs. Quite in contrast, the 0.2 M DFB02_Gallate LCE offers remarkably competitive discharge capacities that are only 10%–15 % lower than that of the 1.0 M LP57 reference at 5C (Figure 4d; Figure S5; and Table S3). The capacities reached, almost completely recover upon return to slower rates. These findings are remarkable and speak for very high Li^+^ ion activity overcoming in part the low charge carrier concentration and agree with high Li^+^ ion mobilities and little anion degradation in the DFB02‐electrolytes.

**Figure 5:**
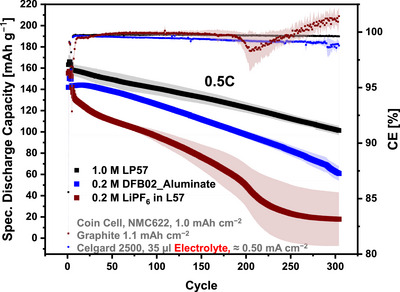
Cycling data of full cells (25 °C) for different electrolyte solutions containing NMC622 cathodes at 0.5C, including a carbonate‐based low‐concentration solution. Quadruplicates were tested for each series of cells.

The performance of the 0.2 M DFB02_Aluminate LCE was further evaluated by comparing the extended full cell cycling data with 1.0 or 0.2 M LiPF_6_‐solutions in L57; Figure 5 reveals significant differences: The capacity of the 0.2 M LiPF_6_‐solution drops rapidly right after the initial formation cycles, indicating sluggish mobility of the charge carriers accompanied by a low conductivity influencing the performance, even at a moderate C‐rate of 0.5C (Figure 5). The erratic behavior of the CEs also confirms the inferior performance of the 0.2 M LiPF_6_‐electrolyte in L57 and suggest increased counter anion participation (degradation?) for [PF_6_]^−^, but not [Al{OC(CF_3_)_3_}_4_]^−^ (cf. Sections 4 and 6).

The superior characteristics of the 0.2 M DFB02 electrolytes become even more evident by comparing the rate performance tests of the different low‐concentration electrolyte systems. Figures S5a and b shows a significant capacity drop of the 0.2 M solution of LiPF_6_ in L57 at slightly increased currents (1C) and, at the latest, a complete breakdown of the capacities at rates ≥2C (cf. DFB02 electrolytes and 1.0 M LP57 reference in Figure 4d). The full cells with 0.2 M LiPF_6_ in L57 maintain only a few mAh g^−1^ at this cycling stage. This supports the assumption of the degradation of the only in low concentration available [PF_6_]^−^, resulting in a further depletion of the charge carriers (see Sections 4, 6).^[^ ^66^, ^67^ ^]^

**Influence of DME**: Since stoichiometric amounts of DME improve the electrochemical performance of the DFB02 LCEs, the addition of 2 equivalents of DME to 0.2 M LiPF_6_ in L57 was also tested at various C‐rates (Figure S5b). Its influence remains very limited and the capacities remain much worse compared to the 0.2 M DFB02 LCEs and 1.0 M LP57. Apparently, the competition between EC / EMC and DME for the coordination sites around Li^+^ is won by the carbonate solvent molecules present in excess. This agrees with the calculated thermodynamics in Section 6.

**Figure 6:**
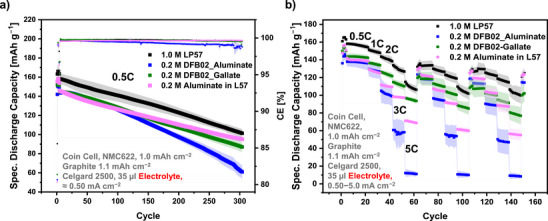
a) Cycling tests of Graphite/NMC622 full cells (25 °C) of different electrolyte solutions at 0.5C, including the 0.2 M carbonate‐based L57 solution with Li[Al{OC(CF_3_)_3_}_4_] as conducting salt; b) rate performance of the same electrolyte solutions at 0.5–5C (3 repetitions) in comparison to the DFB02 analogs. Quadruplicates were tested for each series of cells.

**Influence of Carbonate Solvent**: The performance of the aluminate was also tested in a carbonate‐based solution. Figure 6 shows the electrochemical cycling data of the full cells containing a 0.2 M solution of Li[Al{OC(CF_3_)_3_}_4_] in L57. The full cells maintain the discharge capacities surprisingly well. After 300 cycles, 65.2% of the initial capacity is retained during discharge (Figure 6a).

This exceeds the results of the tested LCEs and is only slightly lower than the LP57 reference. Also, the CEs show a beneficial performance within the 300 cycles, and the 0.2 M Li[Al{OC(CF_3_)_3_}_4_] solution in L57 offers partly superior CEs compared to the 1.0 M LP57 reference, especially at the end of the cycling. The rate capability tests also confirm a decent performance of Li[Al{OC(CF_3_)_3_}_4_] in L57, which is intermediate between the 0.2 M DFB02_Gallate and DFB02_Aluminate LCEs. Note that the 0.2 M solution of Li[Al{OC(CF_3_)_3_}_4_] in L57 was prepared **without** adding any other additives to the solution, such as DME or FEC. Hence, also the 0.2 M solution of Li[Al{OC(CF_3_)_3_}_4_] in L57 is a well‐performing LCE.

### Testing of the DFB02_Aluminate in Lithium‐Metal‐Batteries

The performance of the 0.2 M DFB02 LCEs was further evaluated in LMBs. The focus was set on the DFB02_Aluminate LCE, since DFB02_Gallate, and here presumably the gallate anion, showed an inferior performance in half cells (Figure 4a), i.e., in the presence of Li metal, which is inevitable in LMBs.

**Symmetrical Li–Li Cells**: The initial tests were done in symmetrical Li–Li cells with a 48 µm Li‐metal foil as LMA, with and without HBFEP‐treatment to form an adaptive and self‐healing artificial SEI on the LMA. HBFEP‐treatment was recently shown^[^ ^56^, ^57^ ^]^ to yield an exceptional improvement in cell lifetime. Figure S6 in the SI shows the performance of the HBFEP‐treated Li electrodes in DFB02_Aluminate compared to HBFEP‐treated Li electrodes in 1.0 M LP57 as well as pristine LMAs with LP57 in symmetrical cells. Cells with pristine Li electrodes display a rapidly increasing cell voltage in LP57 until the cutoff voltage (±3 V) is reached after 21 cycles. The HBFEP treatment almost doubles cell‐life to 39 cycles in LP57.^[^ ^57^ ^]^ This is further enhanced to 58 cycles by applying the DFB02_Aluminate LCE, corresponding to a 3‐fold life‐time improvement at a realistic current density of 1 mA cm^−2^. Note that the HBFEP‐treated cells with the DFB02_Aluminate LCE exhibit a voltage drop /short circuit after 58 cycles, hence, a different degradation mechanism as in LP57: short circuits in the aluminate LCE imply dendrite growths, while rapid cell voltage increase in 1.0 M LP57 implies largely electrolyte degradation and depletion.

**Figure 7:**
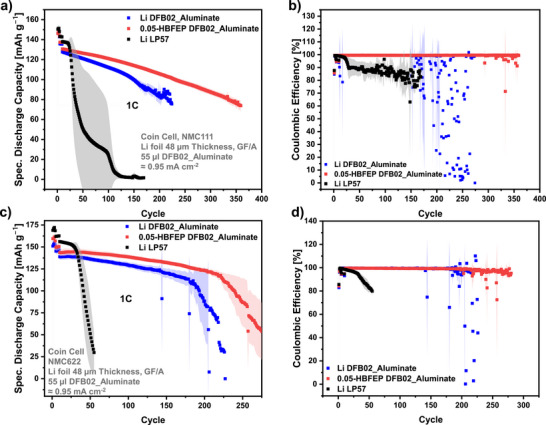
a) Cycling data / b) Coulombic efficiencies of NMC111 full cells (25 °C) with 48 µm thin LMA at 1C as function of the electrolyte and HBFEP‐coating. c) and d) show the data for NMC622 full cells. Quadruplicates were tested for each cell series.

**NMC‐Full Cells**: The DFB02_Aluminate LCE was further tested in LMB full cells with thin 48 µm Li‐metal foil LMAs and NMC111 or NMC622 cathodes at a 1C rate (CR2032 coin cell quadruplicates; Figure 7). While the LP57 cells start to die after 20 to 30 cycles, remarkably, the DFB02_Aluminate LCE enables almost 200 cycles with pristine LMAs and NMC111 cathodes. Even better, the HBFEP‐treated anodes sustain over 350 cycles at 60 % initial discharge capacity retention.

Compared to the 1.0 M LP57 reference, the DFB02_Aluminate LCE cells show an exceptional lifetime increase by one order of magnitude. The CEs of the 1.0 M LP57 reference (Figure 7b) drop sharply right after the start of the cycling and remain at values of 80%–90 %, indicating irreversible processes at the LMAs in combination with the LP57 electrolyte. In contrast, the 0.2 M DFB02 LCE full cells maintain stable CEs over the most significant part of the cycling. Cells with untreated / HBFEP‐treated LMAs show after 175 / 300 cycles an erratic behavior of the CEs (less pronounced for the HBFEP‐LMA), indicating the formation of short circuits (Figure 7b).^[^ ^66^, ^68^, ^69^ ^]^ Notably, the full cells with 0.2 M DFB02 LCE display CEs of >99.5 % throughout the cycling, which is remarkable, since thin 48 µm LMAs are used at charge/discharge currents equal to 1C, besides applying the HBFEP coating. The same conditions were applied to full cells with NMC622 cathodes (Figure 7c). Again, the measurements indicate the superior performance of the 0.2 M DFB02___Aluminate LCE compared to the 1.0 M LP57 reference solution, albeit at slightly lower stability as with NCM111 in Figure 7a. However, the NMC622 full cells (Figures 7c and d) confirm a related situation to the NMC111 pendants: The HBFEP‐treated LMAs perform best and the LP57 reference cells begin to die after 20 cycles. Due to ongoing irreversible processes at the lithium/electrolyte interface, SoH80 is reached after 36 cycles, while the cells with untreated / HBFEP‐treated LMAs in DFB02_Aluminate electrolyte reach SoH80 after 167 / 214 cycles.

### Charge Carrier Mobility and Ionicity in the Electrolyte Systems

To understand the competitive performance of the 0.2 M DFB02 LCEs compared to 0.2 or 1.0 M carbonate‐based electrolytes in NMC full cells, the self‐diffusion coefficients of cations (*D^+^)* and anions (*D* ^−^) were measured in the different electrolyte systems by Pulsed‐Gradient Spin Echo (PGSTE) NMR measurements. Since the solution structures of the solvated Li^+^ cations in DFB02 electrolytes should be different from those of the solvated ions in LP57 (cf. Scheme 1), and the viscosities of the systems vary greatly (cf. Figure 3), one expects differences. Figure 9a and Table 2 gives an overview of the diffusion coefficients measured. But, before we analyze those in detail, the nature of the diffusing cation entity in the DFB02 LCEs needs to be derived.

**Table 2:** Electrolyte systems studied and their corresponding physicochemical properties from this work.

Electrolyte system	Diffusion coefficient *D*_+_ (m^2^ s^−1^)	Diffusion coefficient *D*_−_ (m^2^ s^−1^)	exp. Con‐ductivity (mS cm^−1^) at 298 K	NMR‐Con‐ductivity (mS cm^−1^) at 298 K	Ionicity *I* σ_exp._/ σ_NMR_	Apparent transport number (*t*_+_)	Viscosity (mPa s) at 298 K
**1.0 M** LP57	2.01×10^−10^	2.82×10^−10^	8.15	18.15	0.45	0.42	3.4
**0.2 M** DFB02_Aluminate	7.21×10^−10^	5.99×10^−10^	5.03	9.92	0.51	0.55	0.86
**0.2 M** DFB02_Gallate	7.25×10^−10^	7.40×10^−10^	4.09	9.68	0.37	0.49	0.87
**0.2 M** LiPF_6_ in L57	4.84×10^−10^	6.14×10^−10^	3.78	8.24	0.46	0.44	1.3
**0.2 M** Li[Al{OC(CF_3_)_3_}_4_] in L57	3.85×10^−10^	3.86×10^−10^	2.24	5.80	0.38	0.50	1.7
**0.2 M** LiPF_6_ in L57 + 2 Eq. DME	4.57×10^−10^	5.83×10^−10^	3.79	7.33	0.55	0.44	1.3

**Is Li(DME)_2_^+^ the preferred ion in DFB02_Aluminate?** The 0.2 M DFB02 electrolytes contain, alongside *o*‐DFB, 0.4 M DME and 2 wt.% FEC. To verify whether the small Li(DME)_2_⁺ ion is the major cation responsible for Li⁺ ion transport, the NMR‐based diffusion constants (*D*) of Li⁺, DME, and FEC were measured using PGSTE‐NMR of the ^7^Li, ^1^H, and ^19^F nuclei. If DME is fully complexed as Li(DME)_2_⁺, one would expect *D*(Li^+^), and *D*(DME) to be the same within the method's error margin and different to that of FEC or *o*‐DFB. This is indeed the case: In DFB02_Aluminate, *D*(Li^+^), and *D*(DME) are the same within method error (±0.5) at 7.2 and 8.1 × 10^−10^ m^2^ s^−1^, respectively, and much slower than FEC (11.4 × 10^−10^ m^2^ s^−1^) and o‐DFB (18.1 × 10^−10^ m^2^ s^−1^). Additionally, we measured the diffusion constants of DME, FEC and *o*‐DFB in a solution containing only *o*‐DFB as the solvent, with DME and FEC added but no Li salt present. Here, *D*(DME) is doubled to 15.9 × 10^−10^ m^2^ s^−1^, while the values for FEC and *o*‐DFB have only changed slightly to 13.6 × 10^−10^ and 17.5 × 10^−10^ m^2^ s^−1^. Yet, the by 16% reduced *D*(FEC) in the electrolyte could be due to a small degree of FEC coordination (in agreement with the DFT calculations in Section 6 and Figure 13a). Further, the calibrated ^1^H NMR chemical shifts of the DME molecule in the presence and absence of Li[Al{OC(CF_3_)_3_}_4_] in the same DME, FEC and, *o*‐DFB mixture are 3.49 (CH_2_)/3.35 (CH_3_) and 3.38 (CH_2_)/3.20 (CH_3_) ppm. Hence, they are shifted downfield by 0.11 (CH_2_)/0.15 (CH_3_) ppm through coordination to Li^+^. By contrast, the shifts assigned to FEC and *o*‐DFB are unchanged. This is consistent with the hypothesis that Li(DME)_2_⁺ is the relevant cation for charge transport, with FEC having only a minor role in Li⁺ coordination.

**Figure 8:**
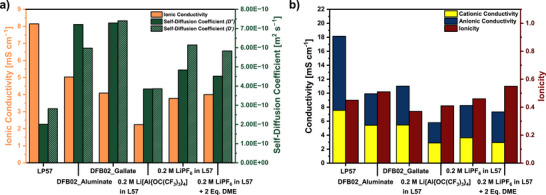
a) Graphical representation of the ionic conductivity and the self‐diffusion coefficients from the data of Table 2; b) theoretical conductivity calculated from the diffusion coefficients and the corresponding ionicity of the electrolyte systems.

**Comparisons between the Electrolytes**: The 1.0 M LP57 reference electrolyte shows sluggish cation mobility at 2.01 × 10^−10^ m^2^ s^−1^ within the electrolyte solution, and the [PF_6_]^−^ anion shows higher mobility (2.82 × 10^−10^ m^2^ s^−1^). This is typical for (smaller) anions, since only the small Li^+^ cation strongly coordinates to the electrolyte components and generates large solvation shells.^[^ ^59^, ^70^ ^]^ The diffusion coefficients of the Li^+^ ions in the 0.2 M DFB02 LCEs are at least increased by a factor of three if compared to the LP57 reference with values of 7.21 × 10^−10^ m^2^ s^−1^ for the 0.2 M DFB02_Aluminate and 7.29 × 10^−10^ m^2^ s^−1^ for the 0.2 M DFB02_Gallate LCEs. In 0.2 M LiPF_6_ in L57, the Li^+^ diffusion coefficient is doubled compared to the 1.0 M LP57 electrolyte, but adding a stoichiometric amount of two DME molecules per Li^+^ cation to this 0.2 M LiPF_6_ electrolyte has almost **no** influence. Hence, in carbonate solution, DME is apparently **not** the preferred ligand for Li^+^. Otherwise, a more significant effect would have been expected. The Li^+^ diffusion coefficient of 0.2 M Li[Al{OC(CF_3_)_3_}_4_] in L57 is also almost doubled compared to the 1.0 M LP57 reference, but is inferior to the 0.2 M LiPF_6_ electrolyte in L57. Yet, the WCA electrolytes are the only ones, where the cation diffusion constant is the same (in L57) or even higher (DFB02) than that of the anion (Figure 8).

However, the motion of the anions and cations in these electrolyte systems is strongly correlated,^[^ ^30^ ^]^ and since the time scale of the NMR method does not allow one to distinguish between single and aggregate ion motion, one cannot clearly separate anion and cation diffusion. Consequently, the diffusion coefficients of the anions tend to follow similar trends to the cation‐behavior.

**Figure 9:**
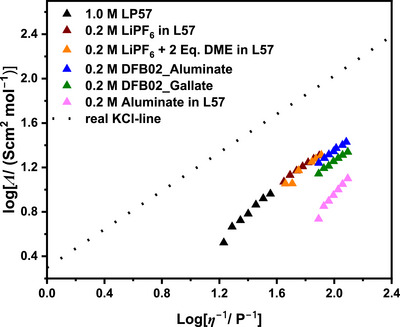
Walden plots of the electrolyte systems, including the real KCl‐line as a visual reference.

**Ionicities**: The knowledge of the diffusion coefficients allow calculation of the theoretically expected conductivity (*σ*_NE_) of the electrolyte solutions according to Equation S2 (Supporting Information, Nernst‐Einstein‐Equation).^[^ ^71^ ^]^ Separation of the cationic and anionic contributions to the total conductivity is also possible‐ within the limitations mentioned above (Figure 9b; Table 2). The ratio of the experimental (*σ*_exp_) (Figure 2) and the theoretically expected conductivity (*σ*_NE_) gives the ionicity (*I*), sometimes referred to as the inverse Haven ratio *H* ^−1^ (Equation (1)):^[^ ^71^ ^]^

(1) $$I=\frac{\sigma_{\exp.}}{\sigma_{\mathrm{NE}}}$$

Since *σ*_NE_ is typically considered as an “ideal” undisturbed conductivity, where all ions contribute fully, and the experimental conductivity *σ*_exp._ already includes all ion‐pairing processes, the ionicity *I* is roughly considered as the amount of fully dissociated ions of the electrolyte salt. A value of *I* = 1 thus corresponds to complete or 100 % dissociation, and a value of *I* = 0 reflects 0 % dissociation. Note that the error bars of ionicity determination are typically within 5%–10 %. Thus, moderate ionicity is associated with a limited number of solvent‐separated ions. However, the presence of freely mobile ions is essential because contact ion pairs (CIP) and aggregate ions (AGG) involve a strong cation‐anion interaction and are either overall charge neutral (CIPs) or have a reduced charge density (AGGs).^[^ ^30^ ^]^ Thus, the generated structures do not move / move slower in an applied electric field, thus limiting the transport properties of the electrolyte solutions (Scheme 1). Since the charge/discharge characteristics of LIBs depend on ion transport between the cathode and anode, undisturbed ions that move the fastest are preferred, but are further leveled by the viscosity of the system (Figure 4).

Of all the electrolyte systems tested electrochemically, the 0.2 M DFB02_Aluminate LCE shows the highest ionicity with *I* = 0.51, highlighting reduced ion‐pairing. Interestingly, the smaller [Ga(C_2_F_5_)_4_]^−^‐based electrolyte DFB02_Gallate displays a lower ionicity of *I* = 0.37. Apparently, the lower polarity of this *o‐*DFB‐based system with the presence of two equivalents of non‐polar DEC from synthesis (*ε*_r_ = 2.8) induce more ion‐pairing with the smaller anion of higher charge density (cf. *ε*_r_ = 13.38 in *o*‐DFB versus 18.5 in L57).^[^ ^44^, ^72^ ^]^ The carbonate‐based 1.0 M LP57 reference solution also exhibits only a moderate ionicity of *I* = 0.45, which is in agreement with the literature.^[^ ^29^, ^30^ ^]^ Figure 9b shows that the ionicity of 0.2 M LiPF_6_ in L57 improves with dilution. The addition of DME to this solution has only a limited influence on the charge carrier mobility and the corresponding ionicity. This again supports the notion that the addition of DME only slightly alters the solvation structure in L57 solutions. Table 2 (Section 7) also gives the self‐diffusion coefficients of the 0.2 M Li[Al{OC(CF_3_)_3_}_4_] solution in L57. The Li ions exhibit a relatively low mobility of 3.35 × 10^−10^ m^2^ s^−1^. Interestingly, an almost identical value was observed for the anion (3.36 × 10^−10^ m^2^ s^−1^), which is consistent with the lowest experimental conductivity within the study (Figure S1). The ionicity reached 0.41, almost equal to the LP57 reference system and slightly better than the DFB02_Gallate LCE.

**Figure 10:**
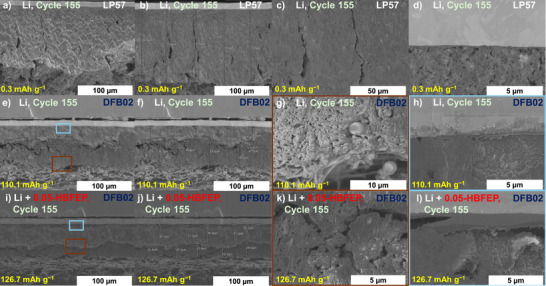
SEM cross section images of cycled Li‐metal anodes in LP57 and DFB02_Aluminate after 155 cycles, but including an artificial SEI in Figure 11i‐l. In all images, the discharge capacity of the last cycle before opening the cells for the SEM measurements are displayed in yellow.

**Walden Plots**: Figure 10 shows the Walden plots of the different electrolyte solutions. The dashed line corresponds to the real KCl line as a visual reference line with high dissociation of the ions over the entire viscosity range.^[^ ^73^ ^]^ Among the DFB02 LCE systems, the aluminate system performs slightly better than the gallate systems. Unexpectedly for its good performance, the 0.2 M Li[Al{OC(CF_3_)_3_}_4_] LCE in L57 carbonate blend is much less ideal. The addition of DME does not seem to affect the ionicity and solvation structure of the L57‐based 0.2 M LiPF_6_ solutions, as the Walden plots display almost identical entries. Interestingly, Figure 10 suggests that the physicochemical difference between the electrolyte systems is less pronounced than anticipated. Apparently, the fluidity (= 1/viscosity) of the electrolytes plays a more critical role in the performance of the cells.

In addition, the calculations of the fraction of the current carried by the Li ions, best called the apparent transport number (*t*_+_), show, according to Equation (2):

(2) $$t_{+}=\frac{D_{+}}{D_{+}+D_{-}}\;$$

a value for DFB02_Aluminate and DFB02_Gallate LCEs between 0.55 and 0.49, respectively, which is higher than that of 0.41 for the LP57 electrolyte solution. The diffusion coefficients are taken from the NMR measurements in Figure 9 and Table 2, and have the same limitations as discussed above (correlated motion of anions and cations^[^ ^30^ ^]^). The low‐concentration 0.2 M carbonate solutions also show an almost identical value of 0.44 for *t*_+_. Since freely moving solvated Li^+^ ions are essential to avoid the formation of concentration gradients at high currents, the high apparent transport numbers of the Li‐ion are also partly responsible for the good performance of the DFB02 LCEs.

### Postmortem Investigations of Li‐Metal Anodes

To evaluate the fate of the LMAs with DFB02_Aluminate LCE, LMB full cells were opened after 55 and 155 cycles and the LMAs were extracted for scanning electron microscopy studies (SEM). Note that the nature of the artificial SEIs in HBFEP‐coated LMAs in comparison to pristine LMAs were intensely studied in the two preceding papers on the HBFEP coating; this includes XPS^[^ ^56^ ^]^ and TOF_SIMS^[^ ^57^ ^]^ measurements in addition to the same SEM‐EDX techniques applied here. Figure 10 shows the LMAs of this work after 155 cycles from cells cycled with the LP57 reference (a‐d), the pure DFB02_Aluminate LCE (e‐h), as well as the ones further modified by the artificial LiBFEP‐SEI (i‐l). The cross‐sections of the LP57 cycled LMAs in Figure 10a–d show severe deterioration of the Li layer with open, needle‐like structures and huge cracks being evident over the entire cutting edge.^[^ ^74^, ^75^ ^]^ The Li structure seems to have pulverized totally and expanded to a thickness of up to 150 µm, corresponding to a threefold increase compared to the initial LMA thickness of 48 µm. Hence, this LP57 cycled cell provided almost no capacity after 155 cycles and its state is close to cell death.

The SEM images of LMAs cycled with DFB02_Aluminate LCE, show significant structural differences compared to those with LP57 electrolyte (Figure 10e–h): Apparently, the overall structure of the electrode is still intact and less deteriorated. After 155 cycles, this cycled LMA is 66 to 79 µm thick (Figure 10f) – a moderate expansion compared to the carbonate‐based pendant and the original 48 µm LMA thickness. Figure 10g displays the magnification of the brown box in Figure 10e and focusses on the electrode / electrolyte interphase. A needle‐like and open lithium morphology, accompanied by large cracks, indicates the deterioration of the upper parts of the Li anode. By contrast, Figure 10h shows a compact layer of dense lithium with a thickness between 24 − 39 µm near the copper current collector with DFB02_Aluminate LCE. Figure 10i–l shows SEM images of a HBFEP‐treated LMA extracted from cells cycled with the DFB02_Aluminate LCE. The artificial LiBFEP‐SEI further supports the stability and performance of LMAs and the degraded needle‐like, open Li structure layer is significantly smaller than in the untreated Li anode (Figure 10j, 19–28 µm). Conversely, the compact Li metal layer in the lower parts of the anode is significantly thicker than in the untreated anode (Figure 10j; 35–41 µm). The overall thickness reaches slightly more than 60 µm, showing that the action of the LiBFEP‐SEI, with 8–10 µm own thickness,^[^ ^56^, ^57^ ^]^ results in much less LMA deterioration during cycling.

**Figure 11:**
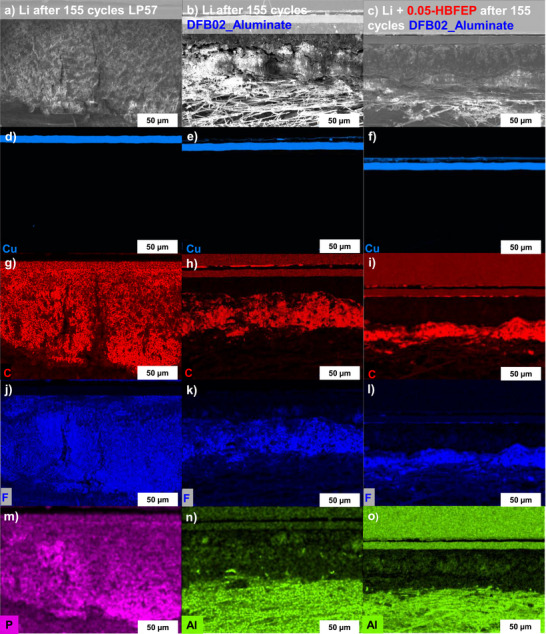
EDX‐Mapping analysis of the cycled Li anodes after 155 cycles and the corresponding elemental distribution of Cu (light blue), C (red), P (purple (just LP57)), F (blue) and Al (green) a) in LP57, b) in DFB02_Aluminate, and c) HBFEP‐treated Li anodes in DFB02_Aluminate.

Energy‐dispersive X‐ray spectroscopical (EDX) mapping on the SEM images in Figure 11 confirms the assignments and conclusions drawn from Figure 10 and the assignment of the lithium metal layer as well as the needle‐like, open Li structure layer as resulting from degradation reactions during the 155 cycles. It is worth to mention, that the results completely agree with those shown in the original two papers,^[^ ^56^, ^57^ ^]^ which in addition were backed up by XPS and TOF‐SIMS work analyses on the artificial HBFEP‐induced SEI. Hence, LiPF_6_ is the only compound containing fluorine and phosphorous in the LP57 reference cells and Figure 11g,j,m indicates that the Li reservoir has completely disappeared during the cycling; open, needle‐like structures can even be seen near the copper current collector.

The presence of a dense lithium metal layer when using the DFB02_Aluminate LCE follows from the EDX mapping in Figure 11h,k,n, since the carbon, fluorine, and aluminum signals are almost absent within this compact layer. By contrast, the carbon, fluorine, and aluminum signals reach maximum intensities in the upper parts of the LMA, with a needle‐like, open Li structure morphology. These superior morphological properties are also reflected by the cell's large discharge capacity of 110.1 mAh g^−1^ before LMA extraction after 155 cycles. The EDX mapping in Figure 11i, l, o indicates a further improvement by the HBFEP‐treatment: The signal intensities of carbon, fluorine, and aluminum overlap in the area, where degradation was assigned and are concentrated in a much smaller area, confirming the minor degree of deterioration. Furthermore, the cell's last discharge capacity was further enhanced by the LiBFEP‐SEI to 126.7 mAh g^−1^ after 155 cycles. Figure S7 shows the state of the LMBs after 55 cycles, already confirming the trends observed after 155 cycles. Overall, the DFB02_Aluminate LCE with the HBFEP‐treated LMA performed the best. By contrast, cycling with the LP57 reference led to a complete breakdown of the Li structure and discharge capacity.^[^ ^76^ ^]^

**Figure 12:**
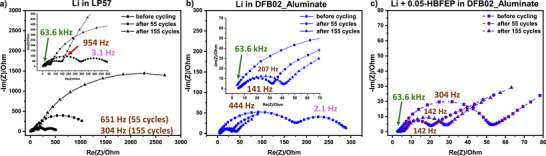
EIS spectra of LMAs in symmetrical Li − Li cells before cycling and after 55 or 155 cycles. The LMAs were extracted from two full cells containing NMC622 cathodes: a) LMBs using commercially applied LP57, b) LMBs cycled with DFB02_Aluminate LCE, and c) LMBs cycled with HBFEP‐treated LMAs and DFB02_Aluminate LCE. The peak frequencies of the different processes are given in the figure. Some peak frequencies happen to be identical for different cells and electrolyte systems.

**Electrochemical Impedance Spectroscopy (EIS)**: The effect of the electrolyte system and the HBFEP treatment on the LMB performance was also investigated by EIS. Figure 12 shows the EIS spectra of symmetrical Li–Li cells containing the extracted LMAs of two LMBs before cycling and after 55 or 155 cycles. Due to the use of this fabrication process in a 2‐electrode setup, we refrain from giving an equivalent circuit diagram and the discussion is only qualitative. Figure 12a demonstrates rapidly increasing resistances throughout the cycling with the LP57 reference electrolyte and indicates severe deterioration of the Li anodes within the 155 cycles. The EIS spectra appear to be qualitatively shifted to higher values over the entire frequency range, suggesting an influence of ion‐related (10^0^–10^3^ Hz) or electron‐related (10^4^–10^5^ Hz) processes. After 155 cycles, the LP57 reference cell shows a huge semicircle at low frequency (several kΩ), which may be related to diffusion processes that lead to the superimposition of the rest of the spectrum.^[^ ^77^ ^]^

The magnification of the high‐frequency parts at 63.6 kHz in Figure S8a, often referred to as electrolyte properties, also reveals a significant shift of the spectra towards higher resistance with LP57 electrolyte. This seems surprising, since the extracted LMAs were tested in symmetrical Li–Li cells, and a new LP57 electrolyte solution was added. The observations may be due to the heavily increased degradation of the LMA, as a highly pulverized layer is visible in Figure 10a, which may prevent sufficient contact between the residual lithium on both sides of the cell and the electrolyte.

The EIS spectra of the LMAs cycled with DFB02___Aluminate LCE (Figure 12b) demonstrate the superior compatibility of the Li[Al{OC(CF_3_)_3_}_4_] solution and the LMAs, with the evolution and shift of the spectra to higher impedances being significantly reduced. Interestingly, the spectrum before cycling exhibits the highest impedance, which drops substantially for the LMAs after 55 cycles and only slightly increases after 155 cycles. Lithium has a native oxide layer with limited Li‐ion conductivity present before cycling.^[^ ^78^, ^79^ ^]^ Hence, cycling in DBF02_Aluminate LCE has to lead to a well ion‐conducting SEI, otherwise, the impedance drop would be impossible. Thereafter, the impedance increases slightly following the progressive degradation of the LMA at 155 cycles. According to Figure S8b, the magnified high‐frequency part is almost unaffected by the number of cycles, confirming the previous observations.

The effect of the artificial LiBFEP‐SEI in Figure 12c shows the further reduced impedances. In contrast to the untreated LMAs in the DFB02_Aluminate LCE, the spectrum exhibits only a minimally higher impedance before cycling, supporting the immediate presence of a beneficial artificial SEI. The symmetrical Li‐Li cells containing cycled LMAs show only very slightly increased impedances after 55 and 155 cycles. It seems that some degradation of the anodes is present, although to a much lesser extent than in the untreated case. The impedance of the extracted HBFEP‐treated LMAs is only a fraction of those cycled in LP57. The high‐frequency part is also largely unaffected by the number of cycles (Figure S8c). Thus, the EIS measurements support the trends drawn from the SEM(‐EDX) measurements in Figure 10 and Figure 11, with the most stable LMA and, simultaneously, the least amount of degradation, present with HBFEP‐treated LMA and DFB02_Aluminate LCE. Note that the exact separation of the different processes according to the time constants is difficult, since the 2‐electrode setup of the coin cell only shows the overall spectrum. Yet, the qualitative trends are evident.

### Quantum Chemical Calculations of the DFB02 Electrolyte Systems

To gain insight into the possible structures present in the electrolyte solutions, quantum chemical calculations at the RI‐B3LYP^[^ ^80^, ^81^, ^82^ ^]^(D3BJ)/def2‐TZVPP^[^ ^83^ ^]^ level of theory with the inclusion of COSMO solvation^[^ ^84^ ^]^ using the relative permittivities *ε*_r_ = 13.38 for *o*‐DFB and 18.5 for L57 were performed to identify and energetically rank the solvates / CIPs present in the different electrolyte solutions according to their relative Gibbs energies *G*°_rel,solv_ (in kJ mol^−1^). Note that through the use of the Li(DEC)_2_[Ga(C_2_F_5_)_4_] conducting salt in the DFB02_Gallate LCE, two equivalents of DEC are introduced to the DFB02 system. These DEC ligands are absent for the DFB02_Aluminate LCE.

**Figure 13:**
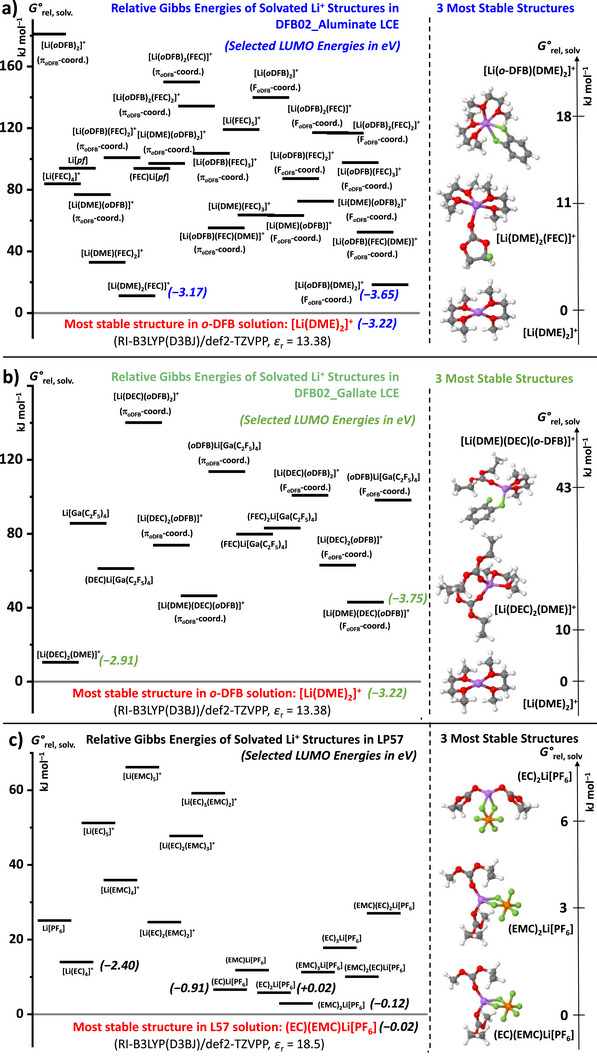
Relative standard Gibbs energy *G*°_rel,solv_ (in kJ mol^−1^) of solvated Li^+^ complexes with the solvent and / or the anion, relative to the most stable [Li(DME)_2_]^+^ structure in *o*‐DFB a, b) or relative to the most stable (EC)(EMC)Li[PF_6_] entity in LP57 c). The three structures calculated to be most stable in the respective electrolyte are given with the explicit *G*°_rel,solv_ values on the right to the schematic representations showing all calculated entities. Selected LUMO energies of the lowest lying relevant entities are included in eV in bold and italics. a, b) represent calculations for the DFB02 LCEs with aluminate (using the short cut [*pf*] for [Al{OC(CF_3_)_3_)}_4_]) and gallate ([Li(DEC)_2_][Ga(C_2_F_5_)_4_]) conducting salts. c) shows the calculations for the LP57 electrolyte with Li[PF_6_] conducting salt. All calculations were performed at the RI‐B3LYP^[^ ^80^, ^81^, ^82^ ^]^(D3BJ)/def2‐TZVPP^[^ ^83^ ^]^ level of theory; solvation energies were calculated with the COSMO method using the indicated relative permittivities *ε*_r_.^[^ ^84^ ^]^ All reactions and additional information are presented in Section 7 in the Supporting Information.

For each of the three electrolyte systems, virtually all thinkable combinations of Li^+^, solvent and anion were optimized and energetically assessed in Figure 13a–c. The baseline for the schematic *G*°_rel,solv_ energy ranking of all assessed entities (left side of the figure) always refers to the most stable particle in this electrolyte, given at the bottom of each Scheme 1a–c in **red**. In addition, the calculated lowest unoccupied molecular orbital (LUMO) energies for the energetically most relevant ions and CIPs are also included in bold and italics. Additional information in Section 7 (Supporting Information) where Table S9 includes the relevant *G*°_rel,solv_ values and LUMO energies of an extended particle set.

**Most Stable Particles in the Electrolyte**: For both DFB02 LCEs, the most stable entity is the [Li(DME)_2_]^+^ ion (cf. working hypothesis in Scheme 1), shown by NMR in Section 4. By contrast, in LP57 the most advantageous entity is the (EC)(EMC)Li[PF_6_] CIP. By contrast, the energetically most favorable CIPs with the large aluminate and gallate WCAs are only found at high *G*°_rel,solv_ of + 94 (aluminate) or + 61 kJ mol^−1^; they are therefore inaccessible. The energetically closest congeners to the [Li(DME)_2_]^+^ complex in the DFB02 LCEs are all cations that contribute favorably to charge transport.

**Relevance to SEI‐Formation**. Of all the assessed structures in Figure 13a b, only the Li(DME)_2_(FEC)^+^ and Li(DME)_2_(*o‐*DFB)^+^ (+11 / +18 kJ mol^−1^; Aluminate LCE) and the Li(DME)(DEC)_2_^+^ (+10 kJ mol^−1^; Gallate) complex cations are thermodynamically accessible in solution and may slightly contribute to charge transport (cf. Section 4), but more importantly to SEI formation. In this respect, the largest difference between the aluminate and gallate LCEs is that, in the absence of DEC, the FEC complex Li(DME)_2_(FEC)^+^ is the second most favorable which may degrade during the formation cycle and be (favorably) incorporated to the SEI.^[^ ^85^, ^86^, ^87^ ^]^ In addition, also the *o*‐DFB complex Li(DME)_2_(*o‐*DFB)^+^ may contribute to SEI formation in aluminate LCE. By contrast, in the gallate LCE, the most favorable FEC complex is residing at relatively high *G*°_rel,solv_ of + 80 kJ mol^−1^. Also, the most favorable *o*‐DFB complex in the gallate LCE, Li(DME)(DEC)(*o‐*DFB)^+^, resides with + 43 kJ mol^−1^ at a high *G*°_rel,solv_ energy. Hence, both fluorinated electrolyte components are **not** expected to be present in relevant quantities in electrolyte solution and are unlikely to contribute to SEI formation.

The calculations for the carbonate‐based LP57 solution in Figure 13c are very different from the DFB02 solutions:^[^ ^72^ ^]^ Alternative structures reside at minimally positive *G*°_rel,solv_ energies with respect to the most favorable CIP (EC)(EMC)Li[PF_6_)]. At least eight different structures are possible within *G*°_rel,solv_ ≤ 20 kJ mol^−1^. But, the majority of alternative complexes formed in carbonate solution are also neutral CIPs, except for the large and thermodynamically accessible [Li(EC)_4_]^+^ ion at *G*°_rel,solv_ + 14 kJ mol^−1^ that is supposed to be relevant for SEI formation (see discussion in Section 7).

**Possible Impact of LUMO Energies**: In Figure 13 and extended also in Table S9 the relevant LUMO energies of the most stable particles in the electrolyte solutions are included. One notices, that the LUMO energies of cations are by at least 1.5 eV lower than those of CIPs. Hence, given they are thermodynamically accessible based on their *G*°_rel,solv_ value, the tendency of cations to be degraded at the negative electrode during the formation cycle is highest, provided that a kinetically viable decomposition channel exists, e.g., for the Li(EC)_4_^+^ ion in the LP57 system (LUMO –2.40 eV, *G*°_rel,solv_ = +14 kJ mol^−1^). Favorably, the LUMO energies of all particles in the DFB02 electrolytes are even lower than that of Li(EC)_4_^+^ and hence, they would be expected to be easier to reduce. Yet, for the Li(DME)_2_^+^ cation (LUMO –3.22 eV), no easy degradation channel for the DME ligand exists, which agrees with the fact that DME does not support SEI formation.^[^ ^48^, ^49^ ^]^ Further, the lowest LUMO energy of all here investigated cations is that of Li(DME)_2_(*o*‐DFB)^+^ (–3.65 eV) at an accessible *G*°_rel,solv_ value of + 18 kJ mol^−1^ (aluminate system). Hence, if this in small quantities available ion is reduced at the negative electrode, it may likely decompose with degradation of the *o*‐DFB molecule and incorporation to the SEI, as suggest by the recent reports on PhF and *o*‐DFB inducing formation of a LiF‐rich SEI.^[^ ^53^, ^54^, ^55^ ^]^

### Short Discussion of Electrolyte Properties and Performance

Overall, the DFB02 LCEs show promising physicochemical properties for battery applications, especially in LMBs. The properties of all investigated electrolytes are collected in Table 2 below.

**Electrolyte Characterization and Speciation**: Conductivity, viscosity, and NMR data show that low‐concentration DFB02 electrolytes exhibit the highest Li⁺ self‐diffusion coefficients – three to four times those of LP57 – indicating superior charge‐carrier mobility (Table 2). This arises from a modified solvation structure dominated by the small, mobile [Li(DME)_2_]⁺ complex, benefiting from reduced viscosity rather than increased ion dissociation. In carbonate electrolytes, two DME molecules have minimal impact on Li⁺ diffusion, confirming their limited solvation role. Anion mobility is less affected, though [Al{OC(CF_3_)_3_}_4_]^−^ and [Ga(C_2_F_5_)_4_]^−^ in DFB02 LCEs move faster than [PF_6_]^−^ in LP57, likely due to lower viscosity. Ionicity differences are small, but relevant. Ionicities are: LP57 44 %, DFB02_Aluminate 51 %, DFB02_Gallate 37 %. Walden plots confirm that enhanced Li⁺ mobility – not higher dissociation – drives DFB02 performance. The low Li⁺ apparent transport number in DFB02_Gallate, despite strong 5C rate performance, suggests additional transport factors in cells beyond NMR observations. DFT calculations indicate and NMR shows that [Li(DME)_2_]⁺ is the most stable species in DFB02, with minimal cation–anion interaction and high ionicity. LP57 contains multiple contact ion pairs, mainly uncharged, limiting mobility. In LP57, bulky [Li(EC)_4_]⁺ complexes and ion‐pairing hinder Li^+^ transport, especially at low 0.2 M concentration. Hence, despite Li[Al{OC(CF_3_)_3_}_4_] in L57 shows sluggish ion mobility from carbonate solvation, it delivers very strong cycling with improved capacity retention and reduced CE fade over 300 cycles. Apparently, the absence of ion‐pairing in the 0.2 M carbonate LCE Li[Al{OC(CF_3_)_3_}_4_] in L57 – induced by the WCA counterion – is the true reason for improved full‐cell capacity retention and reduced CE fade over 300 cycles, that – in contrast to LP57 – leads to reduced impedance built‐up, a point discussed in more detail in the next paragraph.

**Consequences of Ion‐Pairing**: The ample presence of CIPs in the LP57 electrolyte also increases the probability of finding a CIP (e.g., the lowest lying (EC)(EMC)Li[PF_6_]) close to the negative Li electrode rather than the isolated PF_6_^−^ anion itself (electrostatic repulsion of the free anion against the negative electrode). Hence, the consequences are two‐fold:

First, the SEI in LP57 LMB cells contains with higher probability than in the DFB02 cases components of the degraded electrolyte salt LiPF_6_, which may influence SEI‐quality and thickness. Note that the LUMO energy of the isolated PF_6_^−^ ion of + 7.05 eV (Table S9) is greatly reduced by ion‐pairing, e.g., to –0.02 eV in (EC)(EMC)Li[PF_6_] (Figure 13c). While this is still less favorable for reduction than the –2.40 eV LUMO energy of the Li(EC)_4_^+^ ion mentioned before, the first is the mainly occurring and most favored particle in the electrolyte and Li(EC)_4_^+^ at *G*°_rel,solv_ = +14 kJ mol^−1^ has an equilibrium constant *K* for its formation of *K* = 10^−2.45^. Hence, under ideal conditions and in the absence of other effects only one out of 281 CIPs is dissociated to Li(EC)_4_^+^. Yet, under real conditions with the coupling of a manifold of equilibria that all lead to further CIPs (Figure 13c, Tables S4–S9) the ratio is very likely inferior. Thus, the chances are high that one of the CIPs with a kinetically viable degradation channel will decompose at the negative electrode upon formation and/or cycling.

Second, this CIP‐degradation process effectively reduces the amount of available charge carriers. While this is not relevant for the standard 1.0 M LP57 electrolyte with ample of Li^+^ ions, it gets very relevant in the low concentration 0.2 M LiPF_6_ electrolyte in L57. Both consequences of ion‐pairing are absent in the 0.2 M DFB02 electrolyte and also in the 0.2 M Li[Al(OR^F^)_4_] electrolyte in L57 accounting for their longevity and good performance at low concentration.

**NMC Half and Full Cells**: Half‐cell tests confirm DFB02 compatibility with commercial cathodes, though the DFB02_Gallate LCE shows capacity fade with LMAs, likely due to solvation differences: The aluminate allows the formation of thermodynamically accessible [Li(DME)_2_(FEC)]⁺ and [Li(DME)_2_(*o*‐DFB]⁺ solvate ions, aiding SEI formation, while the gallate forms **no** F‐containing Li^+^ complexes with FEC and only an energetically high‐lying one with *o*‐DFB, accounting for the inferior SEI performance in half cells with Li metal anode. In addition, the [Ga(C_2_F_5_)_4_]^−^ anion's more accessible LUMO increases reduction risk, accounting for instability (cf. Supporting Information, Section 7, Figure S9).

Both 0.2 M DFB02 electrolytes enable stable full‐cell cycling with NMC111/NMC622 for 300 cycles (capacity retention: 42.5 % for aluminate, 60.2 % for gallate, versus 64.0 % for LP57). Rate tests show sufficient mobility for up to 2C discharge; DFB02_Gallate reaches 95.5 mAh g^−1^ at 5C, close to 1.0 M LP57 (111.3 mAh g^−1^). Unlike 0.2 M LiPF_6_ in L57, DFB02 LCEs maintain capacity after rate testing.

In carbonate systems, the coordination of added DME to Li⁺ is minimal; bulky [Li(EC)_4_]⁺, and ion‐pairing with [PF_6_]^−^ dominates. Yet, in the DFB02 LCEs, the weak *o*‐DFB coordination allows for the formation of compact, mobile [Li(DME)_2_]⁺, consistent with DFT and NMR. Higher FEC content in DFB02 LCE improves discharge capacity and SEI stability via LiF‐rich SEI, mediated through the thermodynamically accessible Li^+^ solvation with FEC (and *o*‐DFB) as well as the favorable LUMO energies of their Li^+^ complexes.

Also 0.2 M Li[Al{OC(CF_3_)_3_}_4_] in L57 performs very well as LCE with high CEs and capacity retention, despite moderate conductivity and higher viscosity, likely due to greater chemical anion stability – as opposed to LiPF_6_ as conducting salt^[^ ^88^ ^]^ – and the absence of ion‐pairing of the Li^+^ solvates in the presence of the aluminate WCA. This makes the commercially available^[^ ^58^ ^]^ Li[Al{OC(CF_3_)_3_}_4_] in L57 an interesting reference LCE for cases, where counter anion interaction in LIBs should be minimized in long‐term cycling.

**Lithium‐Metal Cells**: The DFB02_Gallate LCE performed worse in half cell measurements^[^ ^89^, ^90^ ^]^ with LMA and was excluded from LMB tests. By contrast, the DFB02_Aluminate LCE shows superior Li‐metal compatibility, enabling 58 / 39 cycles in symmetric Li–Li cells with / without artificial LiBFEP‐SEI^[^ ^56^, ^57^ ^]^ vs. 21 cycles for LP57. In LMB full cells with NMC111, it supports 200 cycles (untreated Li) and 350 cycles (HBFEP‐treated Li) at 1C with 60 % capacity retention; LP57 fails already after ∼30 cycles. CEs remain >99.5 % for 175–300 cycles. The aluminate LCE plus HBFEP‐treatment of the LMA mitigate dendrite growth / SEI instability with a different degradation pathway to the electrolyte degradation / depletion path observed with LP57.^[^ ^91^, ^92^ ^]^

**Morphology and Impedance of the Lithium‐Metal Anode**: SEM/EDX reveals severe Li‐anode degradation in LP57 (LMA thickness tripled to 150 µm, cracks, pulverization, P/F‐rich surface)^[^ ^74^, ^75^, ^93^ ^]^ with final a cell capacity of only 0.3 mAh g^−1^ after 155 cycles. DFB02_Aluminate‐cycled anodes show minimal deterioration after 155 Cycles (70 µm LMA thickness, compact lower Li‐metal layer, final cell capacity: 110.3 mAh g^−1^) and the artificial LiBFEP‐SEI further improves the stability (only 60–65 µm LMA thickness, with ∼40 µm metallic Li layer remaining, final cell capacity: 126.7 mAh g^−1^). EIS confirms the lower impedance growth for the DFB02_Aluminate LCE, with minor changes over 155 cycles. The artificial LiBFEP‐SEI reduces impedance further and from the first cycle on – a consequence of the HBFEP‐treatment that removed the natural lithium oxide layer on the LMA replacing it by an artificial SEI.^[^ ^56^, ^57^ ^]^ By contrast, cells run with LP57 electrolyte show large, diffusion‐dominated low‐frequency features, indicating structural breakdown of the LMA. Data consistently support the superior stability with DFB02_Aluminate LCE, especially with LiBFEP‐SEI.

## Conclusion

Low‐concentration DFB02 electrolytes, particularly the DFB02_Aluminate LCE, achieve superior performance in both Li‐ion and Li‐metal cells due to a favorable solvation structure dominated by compact, mobile [Li(DME)_2_]⁺ complexes. This structure prevails without noticeable ion‐pairing and enhances Li⁺ mobility without relying on increased ion dissociation, as necessary according to the DFT calculation in 1.0 M LP57, and supports stable cycling even at low salt concentrations. In Li‐ion cells, DFB02 blends deliver competitive rate capability and long‐term capacity retention, while in Li‐metal cells, the DFB02_Aluminate LCE – especially supported with an artificial adaptive and self‐healing LiBFEP‐SEI^[^ ^56^, ^57^ ^]^ significantly mitigates dendrite growth, reduces impedance rise, and preserves electrode morphology. These benefits are linked to stable SEI formation, reduced electrolyte reactivity, and sustained high Coulombic efficiencies. In contrast, DFB02_Gallate shows limitations in Li‐metal stability, likely due to less favorable Li^+^ solvation structure without complexed F‐containing entities like FEC or *o*‐DFB and higher anion reducibility.

If one needs to work with a LCE in classical carbonate solution, 0.2 M Li[Al{OC(CF_3_)_3_}_4_] in L57 performs very well with high CEs and capacity retention, a feature we attribute to greater chemical anion stability – as opposed to LiPF_6_ as conducting salt^[^ ^88^ ^]^ and the absence of ion‐pairing of the Li^+^ solvates in the presence of the aluminate WCA. Hence, commercially available^[^ ^58^ ^]^ Li[Al{OC(CF_3_)_3_}_4_] in L57 is an interesting reference LCE for cases, where counteranion interaction in LIBs needs to be minimized, also in long‐term cycling.

Overall, the results highlight the critical role of tailored solvation chemistry in enabling high‐performance, low‐concentration electrolytes for next‐generation rechargeable batteries.

## Electronic Supporting Information

Available (227 pages), eight sections contain the details on Syntheses and Methodologies (1.), Conductivity and viscosity measurements (2.), Cycling data (3.), Determination of the charge carrier mobility and ionicity (4.), Postmortem investigations (5.), Raman Spectra DFB02 solvent mixture and DFB02_Aluminate (6.), Quantum chemical calculations (7.), and Coordinates, Energies etc. Calculated Structures (8.). The supporting information contains additional references.^[^ ^94^, ^95^, ^96^, ^97^, ^98^, ^99^, ^100^, ^101^, ^102^, ^103^, ^104^, ^105^, ^106^, ^107^, ^108^, ^109^, ^110^, ^111^ ^]^

## Conflict of Interests

The authors declare no conflict of interest.

## Supporting information



Supporting Information

## Data Availability

The data that support the findings of this study are available in the Supporting Information of this article.
