# Supplementary material for: Low‐Concentration Electrolytes Based on Weakly Coordinating Anions for Applications in Lithium‐Ion‐Batteries and Lithium‐Metal‐Batteries
Source: Angew Chem Int Ed Engl. 2025 Dec 23;65(6):e23246. doi: 10.1002/anie.202523246 (PMC12865265; doi:10.1002/anie.202523246)
Supplement: Supplementary file 1 — Supporting Information [file ANIE-65-e23246-s001.pdf]

Supporting Information

to

Low-Concentration Electrolytes based on Weakly Coordinating Anions for Applications in Lithium-Ion- and Lithium-Metal-Batteries

Stephan Burger,[a,c] Katharina Tölke,[b] Hendrik Koger,[a,c] Noah Schmidt-Meinzer,[a,c] Antoine Barthélemy,[a] Torsten Remmler,[f] Berthold Hoge,[b] and Ingo Krossing*[a,c,d,e]

[a] S. Burger, Hendrik Koger, Noah Schmidt-Meinzer, Antoine Barthelemy, Prof. Dr. I. Krossing, Institute for Inorganic and Analytical Chemistry, University of Freiburg, Albertstr. 21, 79104 Freiburg im Breisgau, Germany.

[b] K. Tölke, Prof. Dr. B. Hoge, Inorganic Chemistry II, University of Bielefeld, Universitätsstraße 25, 33615 Bielefeld, Germany.

[c] S. Burger, Hendrik Koger, Noah Schmidt-Meinzer, Prof. Dr. I. Krossing, Freiburg Materials Research Center (FMF), University of Freiburg, Stefan-Meier-Str. 21, 79104 Freiburg im Breisgau, Germany.

[d] Prof. Dr. I. Krossing, Freiburg Center for Interactive Materials and Bioinspired Technologies (FIT), University of Freiburg, Georges-Koehler-Allee 105, 79110 Freiburg im Breisgau, Germany.

[e] Prof. Dr. I. Krossing Cluster of Excellence *liv*MatS, University of Freiburg, Georges-Köhler-Allee 105, D-79110 Freiburg, Germany

[f] Torsten Remmler, NETZSCH-Gerätebau GmbH, Wittelsbacherstraße 42, D-95100 Selb, Germany

* Correspondence to krossing@uni-freiburg.de

Table of Contents to Supporting Information

_Toc214722430
1 Syntheses and Methodologies 3
2 Conductivity and viscosity measurements 6
3 Cycling data 7
4 Determination of the charge carrier mobility and ionicity 11
5 Post-mortem investigations 12
6 Quantum chemical calculations 15
7 Coordinates, Energies etc. of Calculated Structures 22
Li^+ 22
FEC 23
DME 24
$[Li(\mathit{o}DFB)_2]^+$ 30
$[Li(FEC)_4]^+$ 32
$[Li(DME)_2]^+$ 35
Li[*pf*] 38
$[Li(DME)(FEC)_2]^+$ 42
$[Li(DME)(\mathit{o}DFB)]^+$ 45
$[Li(\mathit{o}DFB)(FEC)_2]^+$ 47
$[Li(DME)_2(FEC)]^+$ 50
Li(FEC)[*pf*] 53
$[Li(DME)(\mathit{o}DFB)_2]^+$ 58

$[Li(oDFB)_2(FEC)]^+$ 61
$[Li(oDFB)_2(FEC)_2]^+$ 64
$[Li(oDFB)(FEC)_3]^+$ 67
$[Li(oDFB)(FEC)(DME)]^+$ 71
$[Li(FEC)_5]^+$ 74
$[Li(DME)(FEC)_3]^+$ 78
DEC 81
$[Ga(C_2F_5)_4]^-$ 83
$[Li(DME)(DEC)_2]^+$ 85
$Li[Ga(C_2F_5)_4]$ 90
$(DEC)Li[Ga(C_2F_5)_4]$ 92
$[Li(DEC)(oDFB)_2]^+$ 96
$[Li(DEC)_2(oDFB)]^+$ 99
$Li(DME)(DEC)(oDFB)]^+$ 103
$(oDFB)Li[Ga(C_2F_5)_4]$ 106
$(FEC)Li[Ga(C_2F_5)_4]$ 110
$(FEC)_2Li[Ga(C_2F_5)_4]$ 113
EC 117
EMC 118
$[PF_6]^-$ 119
$Li[PF_6]$ 120
$[Li(EC)_4]^+$ 121
$[Li(EC)_5]^+$ 124
$[Li(EMC)_4]^+$ 128
$[Li(EMC)_5]^+$ 132
$[Li(EC)_2(EMC)_2]^+$ 137
$[Li(EC)_2(EMC)_3]^+$ 141
$[Li(EC)_3(EMC)_2]^+$ 146
$[(EC)Li(PF_6)]$ 150
$[(EMC)Li(PF_6)]$ 152
$(EC)_2Li[PF_6]$ 154
$(EMC)_2Li[PF_6]$ 156
$(EC)(EMC)Li[PF_6]$ 159
$(EC)_3Li[PF_6]$ 162
$(EMC)_3Li[PF_6]$ 165
$(EC)_2(EMC)Li[PF_6]$ 169
$(EC)(EMC)_2Li[PF_6]$ 172
$[pf]^{\cdot 2-}$ 176
$[Ga(C_2F_5)_4]^{\cdot 2-}$ 180
$[(DEC)Li]^+$ 182
$[(FEC)Li]^+$ 184

1 Syntheses and Methodologies

All manipulations were carried out in an argon-filled (99.999 %) glovebox (GS MEGA Line 3), where the oxygen content was <3 ppm, and the water content was <1 ppm.

Conductivity measurement: Temperature-dependent ionic conductivities were performed outside the glovebox with the Mettler Toledo SevenMulti conductometer and a 4-pin platinum electrode (Mettler Toledo InLab@710) in an inert home-built cell (3 mL volume). A cryostat connected to the cell regulated the temperature (0-60 °C).

Rheological measurements: The shear viscosity of the electrolyte solutions was measured using a Kinexus Prime Ultra+ rotational rheometer (NETZSCH-Gerätebau GmbH, Germany) equipped with a cylinder peltier temperature control and passive heat exchanger. A coaxial double gap DG25/27, which, according to ISO 3219-2, allows the determination of absolute dynamic shear viscosity functions, was used as measuring geometry. A sample volume of about 5 mL was loaded into the outer ring shear slot of the double gap geometry under an inert gas atmosphere (nitrogen gas) before the inner bob was immersed into the sample during the target gap approach. A covered solvent trap was attached immediately after finishing loading to avoid solvent evaporation. Isothermal pretests were performed to determine the optimum shear rate range for the shear viscosity measurements. The minimum torque of the rotational rheometer limits the viscosity measurements towards lower shear rates, and the onset of Taylor vortex flow acts as an upper shear rate limit[94]. Before the test, a thermal equilibrium of 5 min within a temperature tolerance of +/- 0.1 K was established. As a pre-test, a table of shear rates between 1 s^{-1} and 1.000 s^{-1} was performed, and the Newtonian flow behavior of the electrolyte solutions was measured, which turned into the turbulent flow at higher shear rates. The optimum shear rate for the subsequent temperature-dependent tests was derived to be 10 s−1 with a steady-state tolerance of 2 % to ensure time-independent flow conditions. To follow up on the flow behavior for all temperatures, three shear rates of 7.197 s^{-1}, 10 s^{-1}, and 13.9 s^{-1} were set for the comparison tests. An average shear viscosity across all three shear rates was calculated, each measured with a steady-state tolerance limit of 2 %. The measurements were performed in isothermal steps from -5 °C to 35 °C with a 5 K increment. Shear Viscosity data for each temperature were plotted against the reciprocal absolute temperature multiplied by a factor of 1000 to calculate the Arrhenius activation energy for the temperature-dependent shear viscosity.

Cyclic voltammetry measurements: Cyclic voltammetry was performed under inert conditions with a potentiostat AUTOLAB PGSTAT101 (METROHM) and a three-electrode setup (glass casing) using lithium metal ribbons as a counter electrode (CE) and reference electrode (RE) and a platinum-disc electrode (d = 1 mm, METROHM, 3 to 5 V) or a glassy carbon-disc electrode (d = 1 mm, METROHM, 0 to 3 V) as working electrode (WE) respectively. CE and RE were prepared freshly for each measurement using a fixture connected to the potentiostat. Each measurement was conducted from 0 to 3 V with GC as WE and from 3 to 5 V with Pt as WE, using the same pair of prepared RE and CE. Glass measurement cell, WEs, CE, and RE fixtures were cleaned and dried before each measurement, working electrodes were additionally polished with polishing paste grain sizes of (1, 0.25) µm.

Syntheses: $Li[Al\{OC(CF_3)_3\}_4]$: Dried $Li[AlH_4]$ (1.91 g, 51.1 mmol) was suspended in dried heptane (200 mL). The flask was equipped with an intensive condenser and cooled to −30 °C. Dried 1,1,1,3,3,3-

hexafluoro-2-(trifluoromethyl)propane-2-ol (= Perfluoro-*t*-BuOH, 61.0 g, 36.0 mL, 258 mmol, 5.00 Eq.) was added within 1.5 h under stirring. Afterwards, the mixture was refluxed for 4.5 h. The reaction was stored overnight at −40 °C for product precipitation. The solvent was decanted, the volatiles removed in vacuo, and the obtained yellow solid was sublimed (dynamic vacuum, 120 °C oil bath temperature, −30 °C cooling finger temperature). The $Li[Al\{OC(CF_3)_3\}_4]$ was obtained as a colorless solid (45.3 g, 46.5 mmol, 91.0 %). However, $Li[Al\{OC(CF_3)_3\}_4]$ is now commercially available,[58] albeit sublimation is recommended prior to use to reach battery grade. Full detailed synthesis procedures are given as video tutorial at: https://www.krossing-group.de/video-tutorials/ under "#1 - Perfluoroalkoxyaluminates".

$[Li(DEC)_2][Ga(C_2F_5)_4]$: A solution of *n*-butyllithium (95.0 mL, 152 mmol, 1.6 M in *n*-hexane) in diethyl ether (200 mL) was degassed at −80 °C. Pentafluoroethane (1502 mbar, 188 mmol) was condensed into the solution. After stirring for 30 min at −80 °C a solution of gallium trichloride (3.75 g, 21.6 mmol) and dimethylaminopyridine (DMAP) (2.66 g, 21.8 mmol) in dichloromethane (30 mL) was added to the in situ generated pentafluoroethyllithium at −80 °C. The mixture was allowed to reach room temperature within 21 h. Aqueous hydrochloric acid (1 M, 100 mL) was added to the crude product suspension, and the aqueous layer was separated. The organic layer was washed with aqueous hydrochloric acid (1 M, 3 x 100 mL), and the organic layer was evaporated to half its volume. An aqueous lithium hydroxide solution (1 M, 1 x 100 mL) was added to the crude product solution and stirred for 22 h. The organic layer was separated, and the aqueous layer was extracted with diethyl ether (2 x 30 mL). The combined organic phases were washed with water (2 x 40 mL), and all volatile compounds were removed in a high vacuum over 3 days to obtain a brown liquid (10.5 g). Water (40 mL) was added to the brown liquid, and a brown oil was separated. This mixture was filtered over celite and the filter cake was washed with water (30 mL). Again, diethyl ether (160 mL) and lithium hydroxide (2.72 g, 113 mmol) were added, and the mixture was stirred for 16 h. The organic layer was separated and washed with aqueous lithium hydroxide (1 M, 2 x 80 mL). The organic layer was concentrated under reduced pressure, and the residue was dissolved in diethyl carbonate (40 mL). At 130 °C, half of the solution was distilled off, and all residual volatile compounds were removed in a high vacuum for over 4 days. $[Li(DEC)_2][Ga(C_2F_5)_4]$ was obtained as a yellow, highly fluid liquid (12.3 g, 15.7 mmol, 73.0 %).

Electrochemical measurements: Electrochemical measurements were conducted at 25 °C under controlled temperature conditions (Binder KB53) in a coin cell setup with CR2032 cell format (Gelon, SS316). Half cells (NMC111 1.0 mAh cm^{-2}, Customcells) were built in combination with thick lithium electrodes (450 µm) and 90 µL of electrolyte. The cells were subjected to three formation cycles at C/10 after a rest time of 10 h, followed by one cycle at C/5 and five cycles at C/2. Afterward, the cells were tested at 0.5C for 115 cycles. Full cell measurements were conducted with graphite electrodes (1.2 mAh cm^{-2}, Ø 15 mm, Customcells) and NMC622 cathodes (1.0 mAh cm^{-2}, Ø 14 mm, Customcells). 35 µl of electrolyte were added to the cells using a polymer separator (Celgard 2500, Ø 16 mm). Afterward, the cells were cycled at 0.5C for 300 cycles in a constant current-constant voltage (CC-CV) mode with cut-off voltages at 4.2 V and 2.8 V. The current limit during the constant voltage step was set to C/20. Li-Li symmetrical cells were tested in a coin cell setup with CR2032 cell format (Gelon, SS316). 55 µL of electrolyte was added to the cells using a glass fiber separator (Whatman GF/A, Ø 16 mm). Lithium electrodes (Ø 15 mm) were punched out of a thin Li-metal foil (48 µm thickness on 12 µm copper, China Energy Lithium Co., Ltd.) for the cycling tests. The cycling parameters were kept

as follows: after 10 h initial rest time, the cells were charged and discharged for 1 h with a constant current density of 1 mA cm^{-2} (1 mAh cm^{-2} areal capacity) with a rest time of 3 min in between; the symmetric cell cycling was stopped when a predefined cell voltage was reached (±3 V), or sudden voltage drop was visible in the later stages of the cycling. Thin 48 µm lithium electrodes (Ø 15 mm) were used as anodes and combined with NMC-based cathodes (Ø 14 mm) (NMC111 and NMC622) with an areal loading of 1.0 mAh cm^{-2} (CustomCells). The amount of electrolyte and the separator were identical to the symmetrical cells (Whatman GF/A, Ø 16 mm). The cells were subjected to three formation cycles at C/10 after a rest time of 10 h, followed by one cycle at C/5 and five cycles at C/2. Afterward, the cells were cycled at 1C for 300 cycles in a constant current-constant voltage (CC-CV) mode with cut-off voltages at 4.3 V and 3.0 V. The current limit during the constant voltage step was set to C/20. The coin cells were tested with a Landt CT3001A or CT3002A multi-channel battery tester. The cells for the PEIS measurements were stopped after 55 and 155 cycles. Afterward, identically cycled Li anodes were implemented in Li-Li symmetrical cells using a new separator (Whatman GF/A, Ø 16 mm), and fresh electrolyte (55 µL). The impedance measurements were performed under potential control (PEIS) with a perturbation of 10 mV at OCV in the frequency range between 200 kHz-0.1 Hz. Before the measurements, the cells were rested at OCV for 1 h.

Nuclear magnetic resonance (NMR) spectroscopy: NMR spectra were recorded in pulsed field gradient stimulated spin echo experiments on a BRUKER *Avance II+ WB 400 MHz* and a BRUKER *Avance III HD 300 MHz* at 298 K. The samples were prepared in an inert atmosphere, and NMR tubes (3 mm) were subsequently flame-sealed to prevent the samples from contact with moisture, oxygen, and other atmospheric contamination.

SEM/EDX measurements: SEM-EDX images were obtained with a FEG-HRSEM SU8220 (Hitachi) at 25 °C using an accelerating voltage of 5 kV. An Everhart-Thornley detector was used in the secondary electron (SE) mode. The EDX-measurements were done with a XFlash 6-30-detector (Bruker) and the QUANTAX FlatQUAD-detector (Bruker). The accelerating voltage was also set to 5 kV. The samples were prepared and mounted inside the glovebox. The inert transfer of the samples to the SEM was ensured by a specialized sample holder (Hitachi). For the post-mortem investigations, the cells were opened, the Li-metal electrodes extracted from the battery stack, washed in 1 ml ethylmethyl carbonate (EMC) for 1 min, and dried. The cycled Li anodes were prepared with the Hitachi Ion Milling System ArBlade 5000 to conduct cross-section images. An Ar^{+}-ion beam is used to ablate the lithium with a pulsed sequence, 4 s ablation, and 9 s pause between the laser pulses (accelerating voltage 6 kV, discharge voltage 2 kV), resulting in a total ablation time of 14 h. The Li anodes were transferred under inert conditions to the electron microscope.

2 Conductivity and viscosity measurements

Figure S1 shows the overview of the temperature-dependent conductivity measurements of all low-concentration electrolytes. The comparison demonstrates the limited influence of the addition of DME on the conductivity of the low-concentration $LiPF_6$ solutions in L57.

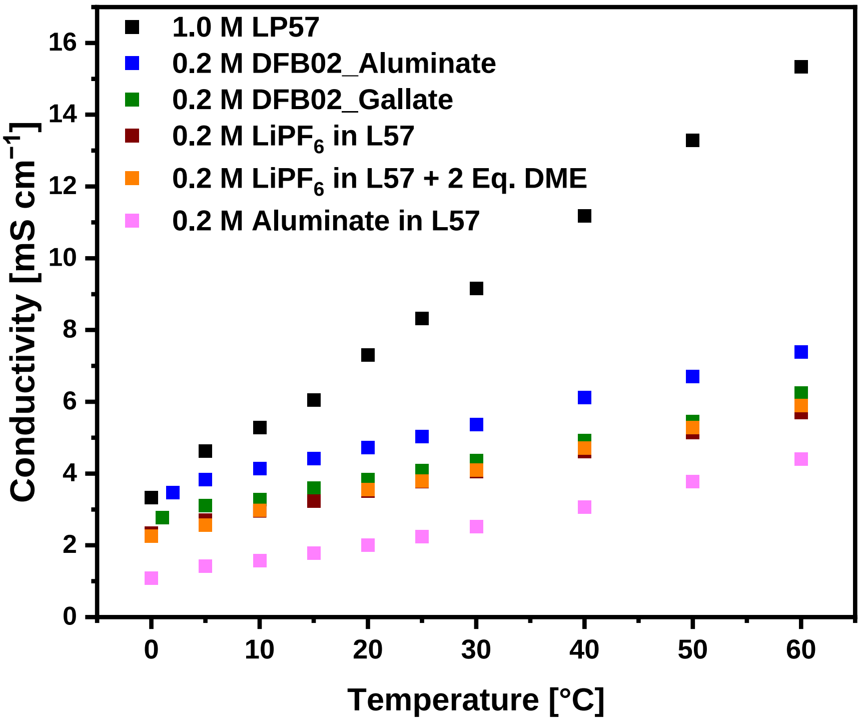

Figure S1. Temperature-dependent conductivity measurement of all low-concentration electrolytes compared to the LP57 reference electrolyte.

Table S1. Additional viscosity data to Figure 4

Electrolyte solution	Fit parameter describing the Arrhenius behavior of the temperature-dependent measurements
1.0 MLP57	y = 6.96E-4 + 3.51E-7*exp(2.65x)
0.2 M DFB02_Aluminate	y = 2.83E-4 + 1.93E-6*exp(1.71x)
0.2 M DFB02_Gallate	y = -2.11E-4 + 2.86E-5*exp(1.08x)
0.2 M $LiPF_6$ in L57	y = 5.49E-4 + 2.31E-7*exp(2.42x)
0.2 M $LiPF_6$ in L57 + 2 Eq. DME	y = 4.23E-4 + 6.69E-7*exp(2.15x)
0.2 M Aluminate in L57	y = 5.75E-4 + 1.13E-6*exp(2.05x)

3 Cycling data

The additional cycling data (Figure S2) show the influence of FEC on the performance of half cells and lithium-metal-based cells. The addition of 5 wt.% or 10 wt.% FEC improves the discharge capacity and overall performance.

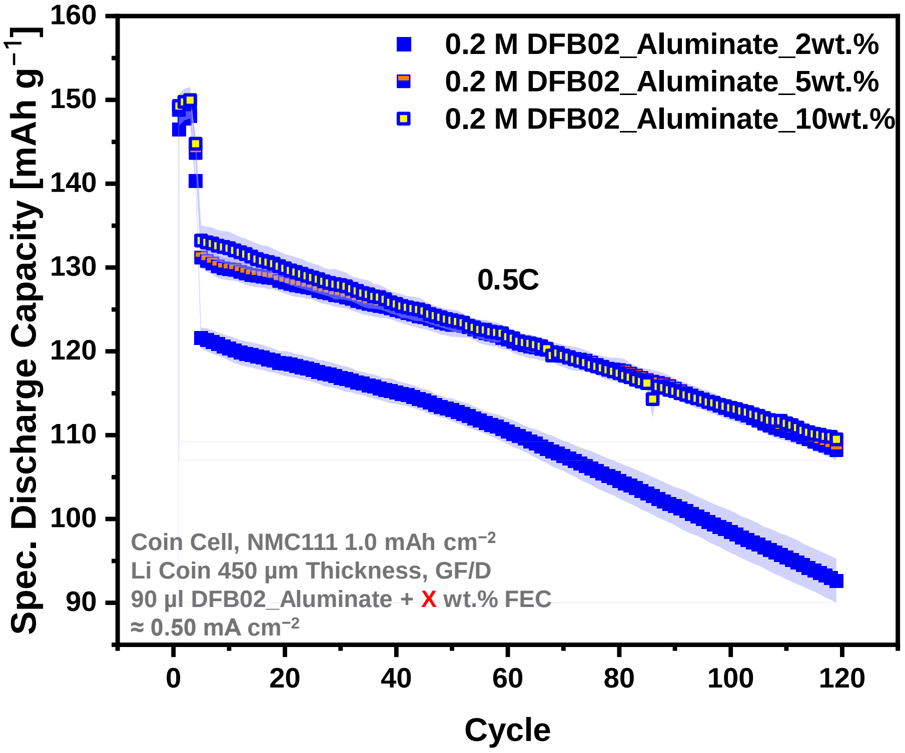

Figure S2. Cycling data of half cells (25 °C) containing different amounts of FEC at 0.5C. Quadruplicates were tested for each series of cells.

Figure S3 depicts the rate capability performance of the DFB02_Aluminate in NMC111 full cells compared to the LP57 electrolyte solution. Table S2 gives an overview of the discharge capacities for each C-rate. The rate capability test confirms the competitive performance of the DFB02 solutions at higher current density conditions. Particularly, the DFB02_Gallate only shows a 20 % lower discharge capacity than the LP57. Note that the DFB02_Gallate is a 0.2 M solution.

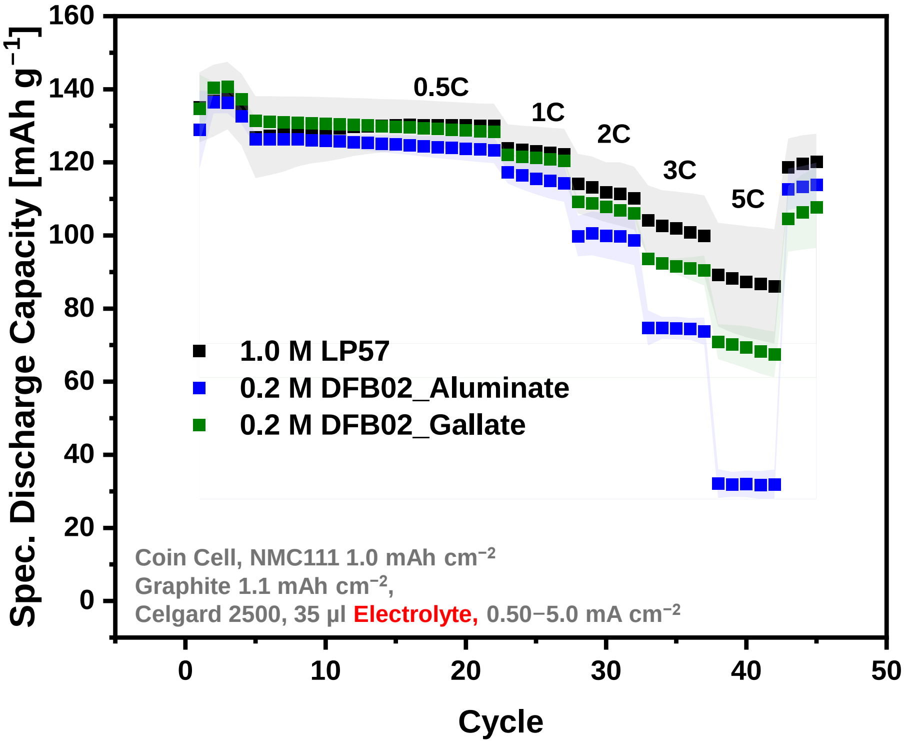

Figure S3. Rate capability tests of NMC111 full cells of the DFB02 electrolytes compared to LP57 at various C-rates (0.5–5C).

Table S2. The specific discharge capacity of the different electrolyte systems during the rate capability test is separated into various C-rates (LIB; NMC111).

Electrolyte system	Specific discharge capacity for each C-rate (mAh g^{-1})				
	0.5C	1C	2C	3C	5C
**LP57**	129.9	122.6	111.3	100.8	86.7
**DFB02_Aluminate**	125.3	115.0	99.7	74.4	31.7
**DFB02_Gallate**	130.1	120.8	106.9	90.9	68.3

Figure S4 shows the influence of higher FEC amounts in the 0.2 M DFB02_Aluminate on the performance of NMC622 full cells. The higher FEC amount seems to lead to improved capacity retention after 300 cycles. The standard solution (2 wt.%) exhibits a kink after 280 cycles, indicating the consumption of FEC within the solution, which may be related to the ongoing degradation at the electrode/electrolyte interface.

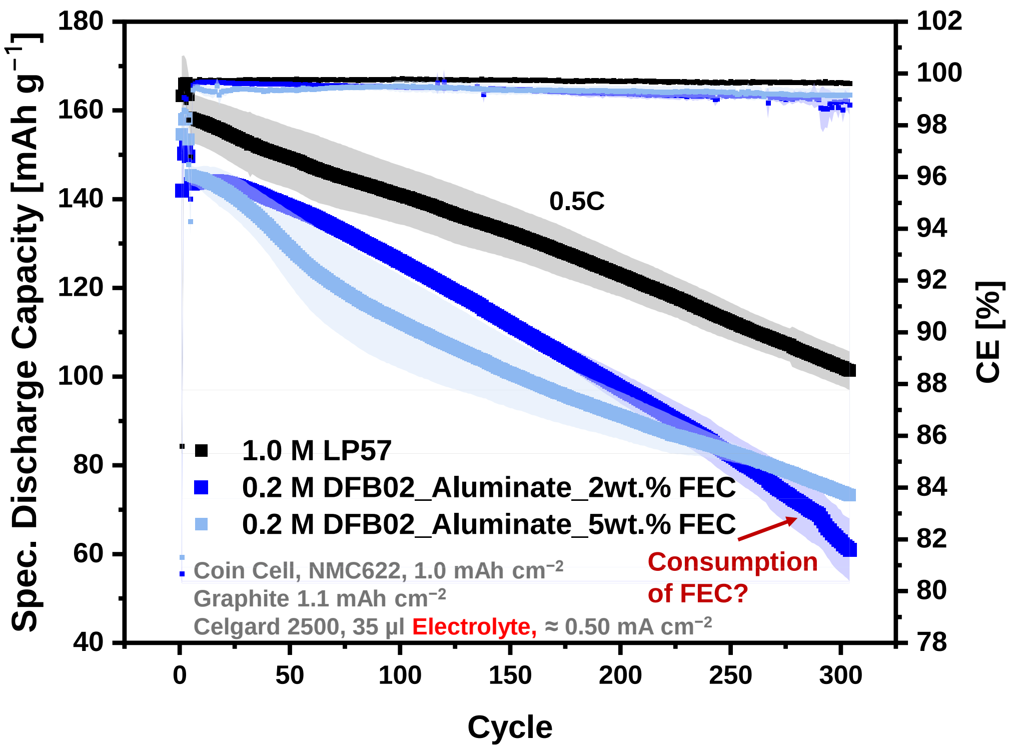

Figure S4. Comparison of the cycling data of full cells with DFB02 solutions containing different amounts of FEC. Quadruplicates were tested for each series of cells at 25 °C.

Table S3 illustrates the additional data to Figure 5b, showing the exact values for each C-rate of the NMC622 full cells at a C-rate between 0.5 and 5C.

Table S3. The specific discharge capacity of the different electrolyte systems during the rate capability test is separated into various C-rates (LIB; NMC622).

Electrolyte system	Specific discharge capacity for each C-rate (mAh g^{-1})				
	0.5C	1C	2C	3C	5C
**LP57**	157.6	150.6	142.9	131.0	111.3
**DFB02_Aluminate**	137.6	128.0	101.5	58.3	12.0
**DFB02_Gallate**	143.6	135.6	124.2	112.7	95.5

The comparison of the rate capability tests (Figure S5a) of the low-concentration (0.2 M) carbonate solutions shows that the retention of the discharge capacity breaks down at higher C-rates. A minor improvement can be seen at 1C through the DME addition, although an increase in the current density leads to almost no obtainable capacity. Figure 5b demonstrates the interior performance compared to the DFB02 solutions, which perform much better at C-rates >1C.

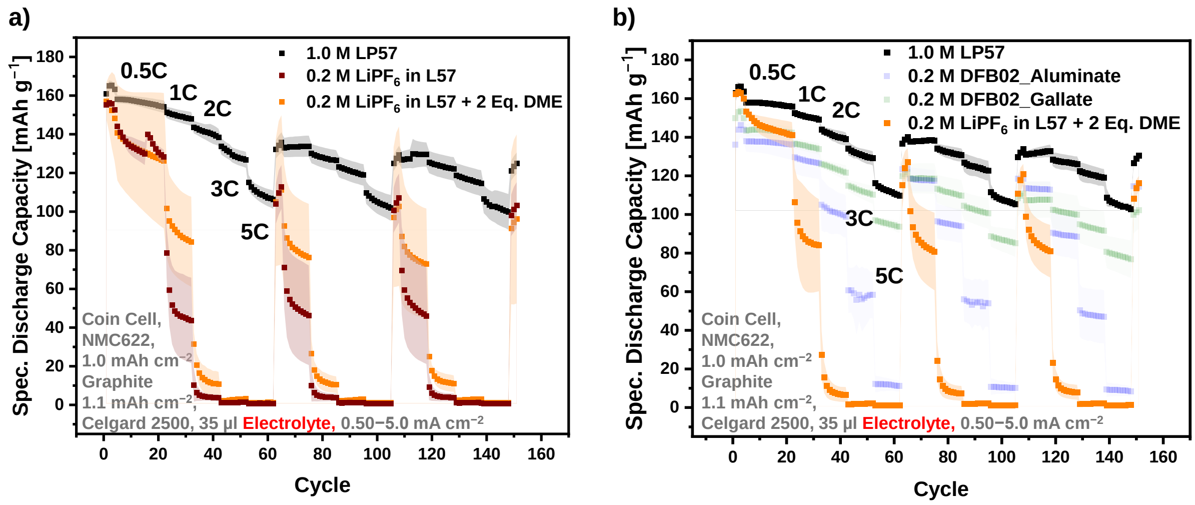

Figure S5. a) Rate capability tests of full cells (25 °C) of different electrolyte solutions using NMC622 cathodes at 0.5C, including low-concentration carbonate-based solutions; b) rate performance of the low-concentration carbonate-based electrolyte solution at 0.5–5C (3 cycles) in comparison to the DFB02 analogs. Quadruplicates were tested for each series of cells.

The performance of the DFB02_Aluminate is evaluated in Li-Li symmetrical cells. Figure S6 shows the cycling data of these cells, which contain HBFEP-treated Li anodes in LP57 and DFB02_Aluminate. The HBFEP-treated Li anodes enable stable cycling at 1 mA cm^{-2} over 39 cycles in LP57. In contrast, HBFEP-treated Li anodes with DFB02_Aluminate allow cycling over 58 cycles, corresponding to a 3-fold increase in the cell's lifetime compared to the untreated Li anodes in LP57. The DFB02_Aluminate outperforms the LP57 cells significantly in combination with an artificial SEI.

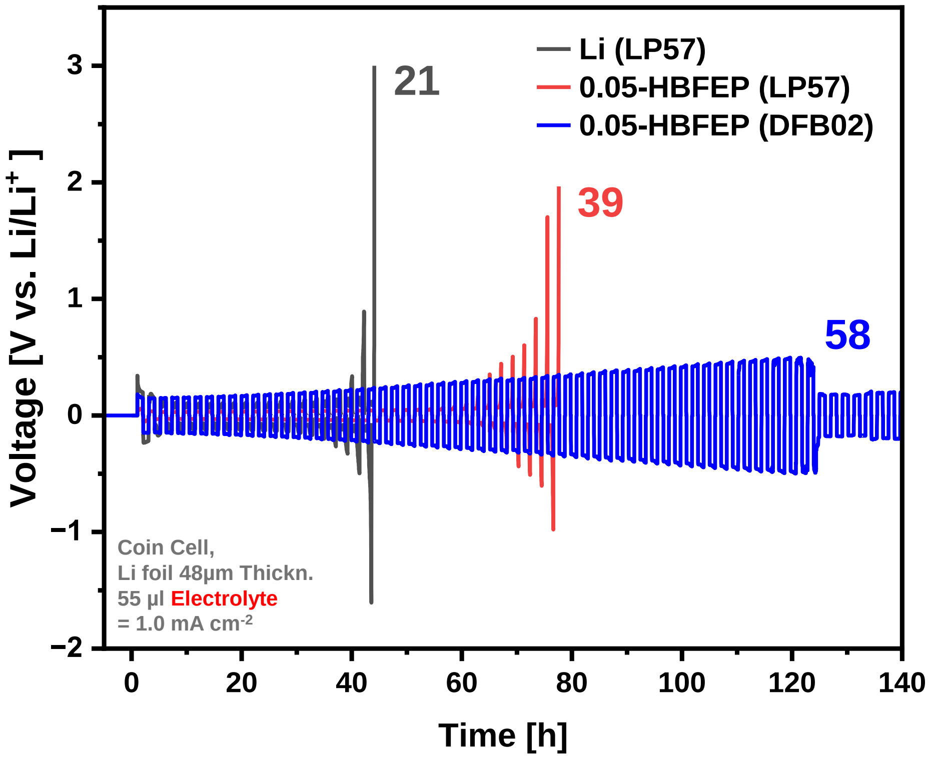

Figure S6. The overall course of Li–Li symmetrical cells at 1 mA cm^{-2} in LP57 and DFB02_Aluminate (25 °C) containing thin Li-metal anodes; c) additional HBFEP-treatment of the Li anodes. Quadruplicates were tested for each series of cells.

4 Determination of the charge carrier mobility and ionicity

The mobility of the charge carriers and their ionicity (ion dissociation) within the electrolyte solutions were determined by pulsed-field gradient stimulated echo (PGSTE) NMR measurements. The self-diffusion coefficients were obtained from ^{7}Li (D^{+}) and ^{19}F (D^{-}) spectra using the NMR signals' integrals. The integrals were fitted to the Stejskal-Tanner equation (equation 1)[1].

$$\frac{I(\Delta,\delta,g)}{I(\delta,\Delta,g=0)}=exp\left[-\gamma^2\delta^2g^2D\left(\Delta-\frac{\delta}{3}\right)\right] \quad (1)$$

with I = signal intensity, δ = gradient pulse width, γ = gyromagnetic ratio, D = self-diffusion coefficient, Δ = diffusion time, and g = gradient magnitude.

The theoretical conductivity σ_{NE} of an electrolyte solution was determined by the NERNST-EINSTEIN equation[2] (equation 2) using the self-diffusion coefficients obtained by the NMR experiments.

$$\sigma_{NE}=\frac{F^2}{RT}\left(c_+z_+^2D_++c_-z_-^2D_-\right) \quad (2)$$

with F = FARADAY Constant, 96485 C mol^{-1}, R = ideal gas constant, 8.314 J K^{-1}mol^{-1}, T =temperature, $D_{\pm}$ = self-diffusion coefficients of cations or anions, $c_{\pm}$ = concentration of cations or anions, $z_{\pm}$ = charge of cations or anions.

To obtain a quantitative assumption of the degree of ion dissociation, the ionicity is calculated by the ratio of the experimental conductivity σ_{exp} and the theoretical conductivity σ_{NE}.

5 Post-mortem investigations

The SEM images (Figure S7) show the conditions of the cycled Li anodes after 55 cycles. The Li anode in LP57 exhibits a severe degree of deterioration (Figure S7a-d), including huge cracks and the presence of thin, needle-like structures. Apparently, the thickness has significantly increased within the 55 cycles, indicating the Li anode's degradation. The almost non-existent discharge capacity supports the previously made assumption. The unprotected Li anode in DFB02_Aluminate shows a significantly improved structure throughout the identical cycling duration. Interestingly, the overall thickness remains slightly lower compared to the initial thickness. The cross-section images (Figure S7e-h) illustrate the absence of any needle-like structures after 55 cycles over the investigated surface. According to the contrast, the lower parts (near the Cu current collector) of the Li anode consist of a decent lithium reservoir. The cell can maintain 127.5 mAh g^{-1} of the initial discharge capacity, corresponding to 93 % of the initial capacity. The protection of the Li anode further improves the condition of the cycled Li anode after 55 cycles. Figure S7i-l shows no indication of significant degradation of the anode structure. The images also demonstrate the existence of a lithium reservoir, which is completely lacking in the SEM images of the cycled Li anode in L57. The LiBFEP-SEI further enhances the preservation of the discharge capacity with 140.4 mAh g^{-1} of the initial capacity. Hence, the SEM images prove the beneficial effect of the LiBFEP-SEI on the performance of LMBs after 55 cycles. The magnification of the high-frequency part of the EIS spectra from the Li-Li symmetrical cells containing cycled Li anodes shows that the spectra of the LP57 cycled anodes are shifting towards higher impedances (Figure S8a). Since the high-frequency part displays electronic processes or electrolyte resistance, the increased deterioration of the anodes within the LP57 is also visible. The cells in the DFB02_Aluminate solutions exhibit a much smaller extent of shifting (Figure 8b,c), indicating the superior structure of the cycled Li anodes. It may be possible that the pulverized Li structure and its corresponding highly increased surface area results in an inferior wetting of the degradation layer and depletion of the electrolyte. The contact between the electrode and the electrolyte is generally inhibited.[3]

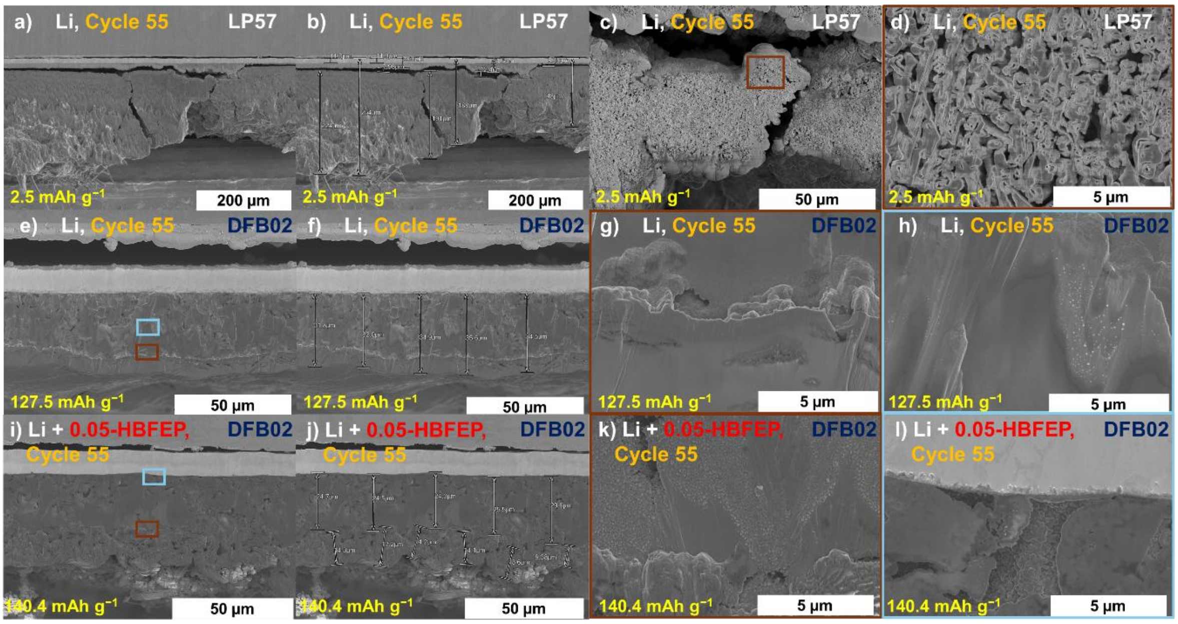

Figure S7. SEM *cross-section* images of cycled Li-metal anodes in LP57 and DFB02_Aluminate after 55 cycles, including an artificial SEI in Figure S7i-l. Figure S7 also shows the discharge capacity of the last cycle before opening the cells for the SEM measurements**.**

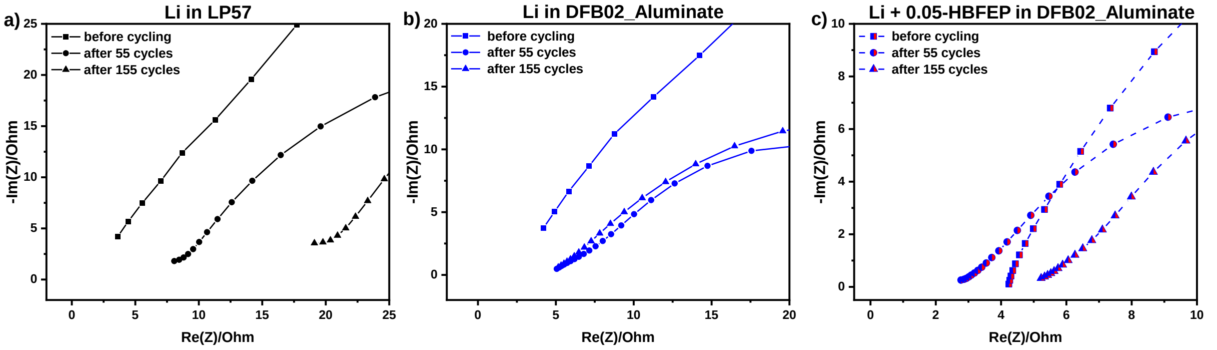

Figure S8. Magnification of the high-frequency part of the EIS spectra of LMBs after different cycling conditions: a) LMBs using commercially applied LP57; b) LMBs using DFB02_Aluminate; and c) LMBs with HBFEP-treated Li anodes and DFB02_Aluminate as electrolyte solution.

6 Raman Spectra of DFB02 Solvent Mixture and DFB02_Aluminate

We have recorded a series of Raman Spectra, collecting 10,000 scans (each spectrum 12 hours), but the analysis remained rather inconclusive, as the 0.2 M concentration of the Li salt is rather low and one cannot see real differences between the blank solvent without Li Salt and the spectrum with 0.2 M Li salt.

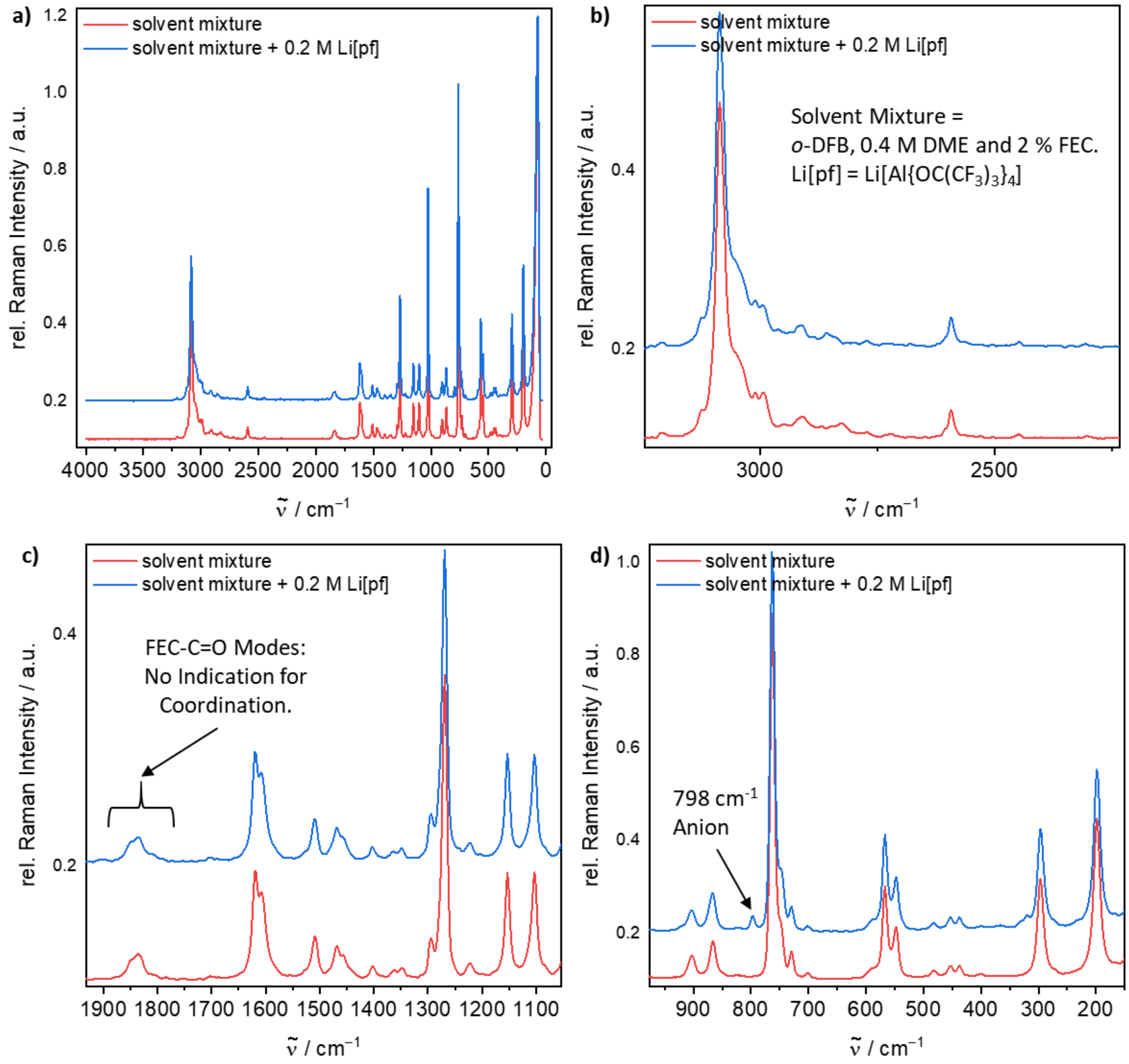

7 Quantum chemical calculations

The quantum chemical calculations reveal that the thermodynamic properties for the reduction of the $[Al\{OC(CF_3)_3\}_4]^-$ and $[Ga(C_2F_5)_4]^-$ go along with almost identical values. The differences (Figure S9) of the reaction energies ($\Delta_rG°$) of the $[Al\{OC(CF_3)_3\}_4]^-$ (−86 kJ mol^{-1}) and the $[Ga(C_2F_5)_4]^-$ (−71 kJ mol^{-1}) are within the margin of error. Therefore, the inferior performance of the $[Li(DEC)_2][Ga(C_2F_5)_4]$ solution in half-cells is probably related to kinetics instead of thermodynamics. Although the energetic levels of the LUMO orbital indicate fast kinetics for the reduction of the anion $[Al\{OC(CF_3)_3\}_4]^-$, its Al-centering prevents the uptake of electrons. On the contrary, the LUMO and the LUMO+1/2 of the anion $[Ga(C_2F_5)_4]^-$ are energetically and structurally more preferred for the uptake of electrons than the $[Al\{OC(CF_3)_3\}_4]^-$.

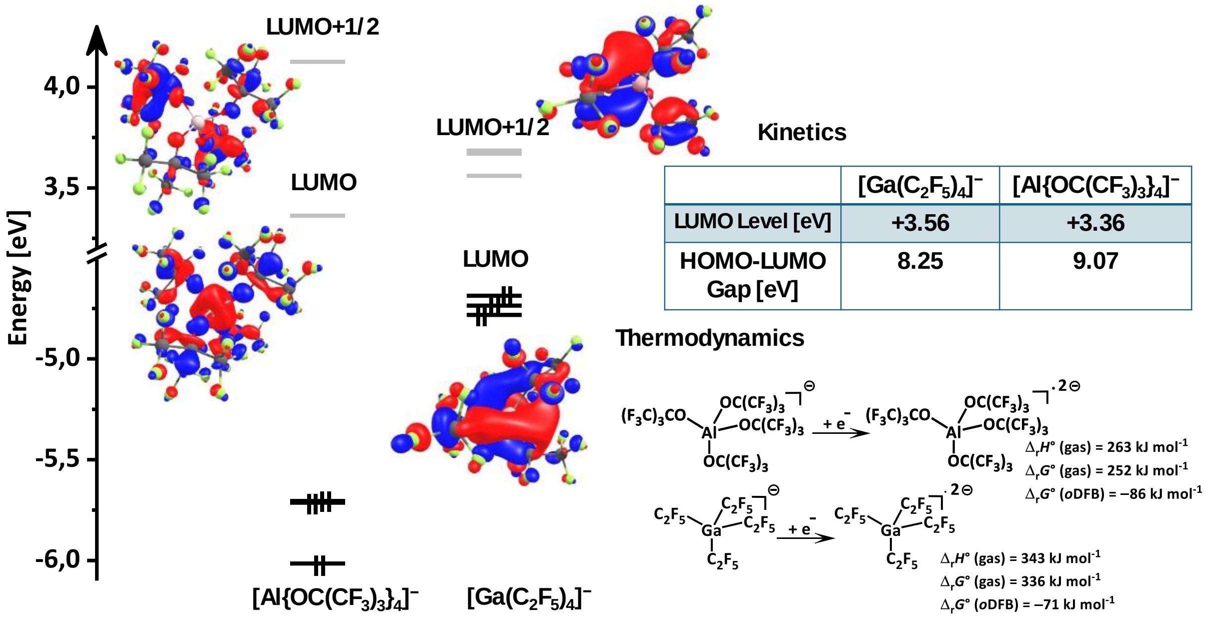

Figure S9. Calculated thermodynamic values for the one-electron reduction of the anions $[Al\{OC(CF_3)_3\}_4]^-$ and $[Ga(C_2F_5)_4]^-$, as well as the energy and shape of their respective frontier orbitals. The latter values are relevant for the kinetics of the one-electron reduction. Structures are optimized at RI-B3LYP(D3BJ)/def2-TZVPP level (isosurface values: 0.035 au).

Note that the following section represents the full data set obtained from the quantum chemical calculations of Figure 14 and Figure S9.

Quantum chemical calculations were performed with *Turbomole* (version 7.5).[4–8],[9],[10],[11],[12],[13] Structures were optimized using density functional theory (DFT),[9] internal coordinates, resolution of identity-approximation (RI),[14–16] D3(BJ)-dispersion correction[17,18] and a fine integration gridsize (gridsize = m5). Calculations were performed at the RI-B3LYP[19–21](D3BJ)/def2-TZVPP[22] level of theory. Thermal and entropic contributions to the Gibbs energy were calculated without scaling factor at standard conditions (298.15 K, 0.1 MPa) with the FREEH module. Every species presented herein was checked in terms of reasonable geometry and electronic occupation with the EIGER module. Vibrational analyses were performed with the AOFORCE module, in order to detect imaginary frequencies.[13]

For each molecular species, the standard enthalpy $H°$ at 298.15 K and 0.1 MPa was calculated from the electronic SCF energy E_{SCF} and the sum of translational, rotational, and vibrational energy including zero-point energy E_{vrt} (FREEH energy) using the following equation (3):

$$H^\circ = E_{SCF} + E_{vrt} + \mathrm{R} \cdot T \quad (3)$$

E_{SCF}: electronic SCF energy

E_{vrt}: sum of translational, rotational, and vibrational energy including zero-point energy

R: universal gas constant (ca. 8.314 J K^{-1} mol^{-1})

T: temperature in Kelvin (298.15 K)

The Gibbs free energy G° follows from the standard enthalpy H° and the standard entropy S°:

$$G^\circ = H^\circ - S^\circ \cdot T \quad (4)$$

Solvation effects were incorporated using the conductor like screening model (COSMO)[23] for *o*-DFB and an EC:EMC 3:7 wt.% mixture (LP57) as the solvent. A dielectric constant of 13.38[24] and 18.5[25] at 298 K was assumed for these solvents, respectively. Single point calculations were performed on the optimized gas phase structures and the results from the vibrational analysis were taken from the respective gas phase calculations.

Table S4: Calculated reaction enthalpies and free reaction enthalpies in the gas phase and in an *o*-DFB solution (RI-B3LYP(D3BJ)/def2-TZVPP; ε_r = 13.38).

Reaction	$\Delta_rH°$ (gas) [kJ mol^{-1}]	$\Delta_rG°$ (gas) [kJ mol^{-1}]	$\Delta_rG°$ (*o*-DFB) [kJ mol^{-1}]
$[Li(DME)_2]^+$ + 2 *o*-DFB → $[Li(o\text{-DFB})_2]^+$ ($\pi_{o\text{-DFB}}$-coord.) + 2 DME	251	233	181
$[Li(DME)_2]^+$+ 4 FEC → $[Li(FEC)_4]^+$ + 2 DME	−52	17	84
$[Li(DME)_2]^+$ + $[pf]^-$ → Li[*pf*] + 2 DME	−82	−117	94
$[Li(DME)_2]^+$ + *o*-DFB → $[Li(DME)(o\text{DFB})]^+$ ($\pi_{o\text{-DFB}}$-coord.) + DME	114	104	77
$[Li(DME)_2]^+$ + 2 FEC → $[Li(DME)(FEC)_2]^+$ + DME	−31	−4	33
$[Li(DME)_2]^+$ + 2 FEC + *o*-DFB → $[Li(o\text{-DFB})(FEC)_2]^+$ ($\pi_{o\text{-DFB}}$-coord.) + 2 DME	69	86	101
$[Li(DME)_2]^+$ + FEC → $[Li(DME)_2(FEC)]^+$	−67	−24	11
$[Li(DME)_2]^+$ + $[pf]^-$ + FEC → Li(FEC)[*pf*] + 2 DME	−142	−133	94
$[Li(DME)_2]^+$ + 2 *o*-DFB → $[Li(DME)(o\text{-DFB})_2]^+$ ($\pi_{o\text{-DFB}}$-coord.) + DME	68	102	97
$[Li(DME)_2]^+$ + 2 *o*-DFB + FEC → $[Li(o\text{-DFB})_2(FEC)]^+$ ($\pi_{o\text{-DFB}}$-coord.) + 2 DME	131	154	150
$[Li(DME)_2]^+$ + 2 *o*-DFB + 2 FEC → $[Li(o\text{-DFB})_2(FEC)_2]^+$ ($\pi_{o\text{-DFB}}$-coord.) + 2 DME	24	96	134
$[Li(DME)_2]^+$ + *o*-DFB + 3 FEC → $[Li(o\text{-DFB})(FEC)_3]^+$ ($\pi_{o\text{-DFB}}$-coord.) + 2 DME	−28	44	104
$[Li(DME)_2]^+$ + *o*-DFB + FEC → $[Li(o\text{-DFB})(FEC)(DME)]^+$ ($\pi_{o\text{-DFB}}$-coord.) + DME	13	42	55
$[Li(DME)_2]^+$ + 5 FEC → $[Li(FEC)_5]^+$ + 2 DME	−92	27	119
$[Li(DME)_2]^+$ + 3 FEC → $[Li(DME)(FEC)_3]^+$ + DME	−86	−3	64
$[Li(DME)_2]^+$ + 2 *o*-DFB → $[Li(o\text{-DFB})_2]^+$ ($F_{o\text{-DFB}}$-coord.)+ 2 DME	178	165	140
$[Li(DME)_2]^+$ + *o*-DFB → $[Li(DME)(o\text{-DFB})]^+$ ($F_{o\text{-DFB}}$-coord.) + DME	82	72	63

$[Li(DME)_2]^+$ + 2 FEC + *o*-DFB → $[Li(\textit{o}\text{-DFB})(FEC)_2]^+$ ($F_{o\text{-DFB}}$-coord.) + 2 DME	44	60	87
$[Li(DME)_2]^+$ + 2 *o*-DFB → $[Li(DME)(\textit{o}\text{-DFB})_2]^+$ ($F_{o\text{-DFB}}$-coord.) + DME	18	56	73
$[Li(DME)_2]^+$ + 2 *o*-DFB + FEC → $[Li(\textit{o}\text{-DFB})_2(FEC)]^+$ ($F_{o\text{-DFB}}$-coord.) + 2 DME	73	96	117
$[Li(DME)_2]^+$ + 2 *o*-DFB + 2 FEC → $[Li(\textit{o}\text{-DFB})_2(FEC)_2]^+$ ($F_{o\text{-DFB}}$-coord.) + 2 DME	−1	66	117
$[Li(DME)_2]^+$ + *o*-DFB + 3 FEC → $[Li(\textit{o}\text{-DFB})(FEC)_3]^+$ ($F_{o\text{-DFB}}$-coord.) + 2 DME	−27	40	98
$[Li(DME)_2]^+$ + *o*-DFB + FEC → $[Li(\textit{o}\text{-DFB})(FEC)(DME)]^+$ ($F_{o\text{-DFB}}$-coord.) + DME	−4	26	53
$[Li(DME)_2]^+$ + *o*-DFB → $[Li(\textit{o}\text{-DFB})(DME)_2]^+$ ($F_{o\text{-DFB}}$-coord.)	−50	−4	18

Table S5: Calculated reaction enthalpies and free reaction enthalpies in the gas phase and in an *o*-DFB solution (RI-B3LYP(D3BJ)/def2-TZVPP; ε_r = 13.38).

**Reaction**	**$\Delta_rH°$ (gas) [kJ mol^{-1}]**	**$\Delta_rG°$ (gas) [kJ mol^{-1}]**	**$\Delta_rG°$ (*o*-DFB)**
$[Li(DME)_2]^+$ + 2 DEC → $[Li(DEC)_2(DME)]^+$ + DME	−43	−18	10
$[Li(DME)_2]^+$ + $[Ga(C_2F_5)_4]^-$ → $Li[Ga(C_2F_5)_4]$ + 2 DME	−62	−106	86
$[Li(DME)_2]^+$ + $[Ga(C_2F_5)_4]^-$ + DEC → $(DEC)Li[Ga(C_2F_5)_4]$ + 2 DME	−174	−179	61
$[Li(DME)_2]^+$ + DEC + 2 *o*-DFB → $[Li(DEC)(\textit{o}\text{-DFB})_2]^+$ ($\pi_{o\text{-DFB}}$-coord.)+ 2 DME	116	141	140
$[Li(DME)_2]^+$ + 2 DEC + *o*-DFB → $[Li(DEC)_2(\textit{o}\text{-DFB})]^+$ ($\pi_{o\text{-DFB}}$-coord.)+ 2 DME	50	63	74
$[Li(DME)_2]^+$ + DEC + *o*-DFB → $[Li(DME)(DEC)(\textit{o}\text{-DFB})]^+$ ($\pi_{o\text{-DFB}}$-coord.)+ DME	4	35	46
$[Li(DME)_2]^+$ + $[Ga(C_2F_5)_4]^-$ + *o*-DFB → $(\textit{o}\text{-DFB})Li[Ga(C_2F_5)_4]$ ($\pi_{o\text{-DFB}}$-coord.)+ 2 DME	−111	−119	114
$[Li(DME)_2]^+$ + $[Ga(C_2F_5)_4]^-$ + FEC → $(FEC)Li[Ga(C_2F_5)_4]$ + 2 DME	−155	−155	80
$[Li(DME)_2]^+$ + $[Ga(C_2F_5)_4]^-$ + 2 FEC → $(FEC)_2Li[Ga(C_2F_5)_4]$ + 2 DME	−231	−185	83

$[Li(DME)_2]^+$ + DEC + 2 *o*-DFB → $[Li(DEC)(o\text{-}DFB)_2]^+$ ($F_{o\text{-}DFB}$-coord.) + 2 DME	64	81	101
$[Li(DME)_2]^+$ + 2 DEC + *o*-DFB → $[Li(DEC)_2(o\text{-}DFB)]^+$ ($F_{o\text{-}DFB}$-coord.) + 2 DME	24	36	63
$[Li(DME)_2]^+$ + DEC + *o*-DFB → $[Li(DME)(DEC)(o\text{-}DFB)]^+$ ($F_{o\text{-}DFB}$-coord.) + DME	−17	21	43
$[Li(DME)_2]^+$ + $[Ga(C_2F_5)_4]^-$ + *o*-DFB → (*o*-DFB)Li$[Ga(C_2F_5)_4]$ ($F_{o\text{-}DFB}$-coord.) + 2 DME	−123	−129	98

Table S6: Calculated reaction enthalpies and free reaction enthalpies in the gas phase and in an *o*-DFB solution (RI-B3LYP(D3BJ)/def2-TZVPP; ε_r = 13.38).

**Reaction**[a)]	$\Delta_r H°$ **(gas) [kJ mol^{-1}]**	$\Delta_r G°$ **(gas) [kJ mol^{-1}]**	$\Delta_r G°$ **(*o*-DFB) [kJ mol^{-1}]**
$[Li(DME)_2]^+$ → Li^+ + 2 DME	456	375	96
$[Li(DME)_2(FEC)]^+$ → Li^+ + 2 DME + FEC	522	399	85
$[Li(DEC)_2(DME)]^+$ → Li^+ + 2 DEC + DME	499	393	86

a) The sum of the translational, rotational, and vibrational energy, including the zero-point energy of a single Li+ ion, equals 3/2 RT ≈ 3.718 kJ mol^{-1}. The standard entropy of Li^+ was calculated with the Sackur-Tetrode equation.[26]

Table S7: Calculated reaction enthalpies and free reaction enthalpies in the gas phase and in an *o*-DFB solution (RI-B3LYP(D3BJ)/def2-TZVPP; ε_r = 13.38).

**Reaction**	$\Delta_r H°$ **(gas) [kJ mol^{-1}]**	$\Delta_r G°$ **(gas) [kJ mol^{-1}]**	$\Delta_r G°$ **(*o*-DFB) [kJ mol^{-1}]**
Li[*pf*] → Li^+ + $[pf]^-$	537	492	2
(FEC)Li[*pf*] → $[(FEC)Li]^+$ + $[pf]^-$	401	340	-13
(FEC)Li[*pf*] → FEC + Li^+ + $[pf]^-$	597	508	3
(DEC)Li$[Ga(C_2F_5)_4]$ → $[(DEC)Li]^+$ + $[Ga(C_2F_5)_4]^-$	421	374	13
(DEC)Li$[Ga(C_2F_5)_4]$ → DEC + Li^+ + $[Ga(C_2F_5)_4]^-$	629	554	35
(FEC)Li$[Ga(C_2F_5)_4]$ → $[(FEC)Li]^+$ + $[Ga(C_2F_5)_4]^-$	414	362	1
(FEC)Li$[Ga(C_2F_5)_4]$ → FEC + Li^+ + $[Ga(C_2F_5)_4]^-$	610	530	17

Table S8: Calculated reaction enthalpies and free reaction enthalpies in the gas phase and in an EC:EMC 3:7 wt.% (LP57) solution (RI-B3LYP(D3BJ)/def2-TZVPP, ε_r = 18.5).

Reaction	$\Delta_r H°$ (gas) [kJ mol^{-1}]	$\Delta_r G°$ (gas) [kJ mol^{-1}]	$\Delta_r G°$ (LP57) [kJ mol^{-1}]
(EC)(EMC)Li[PF_6] → Li[PF_6] + EC + EMC	193	113	25
(EC)(EMC)Li[PF_6] + 3 EC → [Li(EC)$_4$]$^+$ + EMC + [PF_6]$^-$	243	263	14
(EC)(EMC)Li[PF_6] + 4 EC → [Li(EC)$_5$]$^+$ + EMC + [PF_6]$^-$	182	257	51
(EC)(EMC)Li[PF_6] + 3 EMC → [Li(EMC)$_4$]$^+$ + EC + [PF_6]$^-$	254	300	36
(EC)(EMC)Li[PF_6] + 4 EMC → [Li(EMC)$_5$]$^+$ + EC + [PF_6]$^-$	220	315	66
(EC)(EMC)Li[PF_6] + EC + EMC → [Li(EC)$_2$(EMC)$_2$]$^+$ + [PF_6]$^-$	241	285	25
(EC)(EMC)Li[PF_6] + EC + 2 EMC → [Li(EC)$_2$(EMC)$_3$]$^+$ + [PF_6]$^-$	187	278	48
(EC)(EMC)Li[PF_6] + 2 EC + EMC → [Li(EC)$_3$(EMC)$_2$]$^+$ + [PF_6]$^-$	197	282	59
(EC)(EMC)Li[PF_6] → (EC)Li[PF_6] + EMC	96	44	7
(EC)(EMC)Li[PF_6] → (EMC)Li[PF_6] + EC	83	44	12
(EC)(EMC)Li[PF_6] + EC → (EC)$_2$Li[PF_6] + EMC	1	0	6
(EC)(EMC)Li[PF_6] + EMC → (EMC)$_2$Li[PF_6] + EC	-2	7	3
(EC)(EMC)Li[PF_6] + 2 EMC → (EMC)$_3$Li[PF_6] + EC	-53	5	11
(EC)(EMC)Li[PF_6] + 2 EC → (EC)$_3$Li[PF_6] + EMC	-62	-16	18
(EC)(EMC)Li[PF_6] + EMC → (EC)(EMC)$_2$Li[PF_6]	-64	-11	10
(EC)(EMC)Li[PF_6] + EC → (EC)$_2$(EMC)Li[PF_6]	-32	13	27

Table S9: Collection of the relative Gibbs energies of the energetically most relevant solvated particles $G°_{rel}$ in kJ mol^{-1} and their respective LUMO energies in eV calculated at the RI-B3LYP(D3BJ)/def2-TZVPP level of theory with the inclusion of COSMO solvation energies using the relative permittivities ε_r = 13.38 for *o*-DFB and 18.5 for L57.

DFB02_Aluminate System			DFB02_Gallate System			LP57 / $LiPF_6$ in EC:EMC = 3:7		
Species	$G°_{rel}$ / kJ mol^{-1}	LUMO / eV	Species	$G°_{rel}$ / kJ mol^{-1}	LUMO / eV	Species	$G°_{rel}$ / kJ mol^{-1}	LUMO / eV
$[Al\{OC(CF_3)_3\}_4]^-$	-	+3.36	$[Ga(C_2F_5)_4]^-$	-	+3.54	$[PF_6]^-$ (-)	-	+7.05
$[Li(DME)(FEC)_2]^+$	+33	–3.15	$[Li(DEC)_2(DME)]^+$	+10	–2.91	$(EMC)_2(EC)Li[PF_6]$	+10	+0.08
$[Li(DME)_2(FEC)]^+$	+11	–3.17	$[Li(DME)_2]^+$	0	–3.22	$(EC)_2Li[PF_6]$	+6	+0.02
$[Li(DME)_2]^+$	0	–3.22	$[Li(DME)(DEC)\text{-}(oDFB)]^+$	+43	–3.75	$(EC)(EMC)Li[PF_6]$	0	–0.02
$[Li(oDFB)(DME)_2]^+$	+18	–3.65				$(EMC)_3Li[PF_6]$	+11	–0.02
						$(EMC)_2Li[PF_6]$	+3	–0.12
						$(EMC)Li[PF_6]$	12	–0.52
						$(EC)Li[PF_6]$	+7	–0.91
						$[Li(EC)_4]^+$	+14	–2.40

For comparison, the LUMO energies of the relevant neutral solvent mixture constituents calculated at the same level as above in Table S9 are:

FEC: +0.29 eV

oDFB: -0.78 eV

EC: +0.49 eV

EMC: +0.93 eV

DEC: +0.97 eV

DME: +1.14 eV

8 Coordinates, Energies etc. of Calculated Structures

Li^+

```
Method: (RI-)B3LYP(D3BJ)/def2-TZVPP
Symmetry: c1

Cartesian coordinates in Ångström:
Li   -8.4196200    0.8351200    0.0000000

SCF energy GEOOPT =    -7.2785595771 H

Total COSMO energy + OC corr. = -7.428626078 H
```

o-DFB

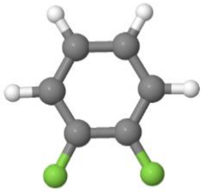

```
Method: (RI-)B3LYP(D3BJ)/def2-TZVPP
Symmetry: c2v

Cartesian coordinates in Ångström:
F    -1.3493503    0.0000000    2.3225501
C    -0.6943849    0.0000000    1.1502851
C    -1.3954274    0.0000000   -0.0412338
H    -2.4758725    0.0000000   -0.0114715
C    -0.6948489    0.0000000   -1.2431476
H    -1.2389980    0.0000000   -2.1769823
C     0.6948489    0.0000000   -1.2431476
H     1.2389980    0.0000000   -2.1769823
C     1.3954274    0.0000000   -0.0412338
H     2.4758725    0.0000000   -0.0114715
C     0.6943849    0.0000000    1.1502851
F     1.3493503    0.0000000    2.3225501

SCF energy GEOOPT =  -430.6996733142 H
ZPE =   220.7 kJ/mol
FREEH energy =   236.38 kJ/mol
FREEH entropy =   0.32053 kJ/mol/K

$vibrational spectrum
#  mode     symmetry     wave number   IR intensity    selection rules
#                         cm**(-1)        km/mol          IR     RAMAN
     1                       0.00         0.00000        -        -
     2                       0.00         0.00000        -        -
     3                       0.00         0.00000        -        -
     4                       0.00         0.00000        -        -
     5                       0.00         0.00000        -        -
     6                       0.00         0.00000        -        -
     7         a2          188.67         0.00000       NO       YES
     8         a1          288.64         0.22913       YES      YES
     9         b2          293.25         0.01868       YES      YES
    10         b1          444.97         0.07558       YES      YES
    11         b2          463.90         3.69507       YES      YES
    12         b1          553.02         3.67952       YES      YES
```

13	a2	565.59	0.00000	NO	YES
14	a1	585.07	4.84279	YES	YES
15	a2	704.63	0.00000	NO	YES
16	b2	767.15	80.35915	YES	YES
17	a1	778.60	33.73975	YES	YES
18	a2	857.89	0.00000	NO	YES
19	b1	865.30	19.61020	YES	YES
20	b2	948.94	4.76774	YES	YES
21	a2	972.95	0.00000	NO	YES
22	a1	1050.20	6.55799	YES	YES
23	b1	1125.98	20.93863	YES	YES
24	a1	1178.68	1.18709	YES	YES
25	b1	1223.23	40.66770	YES	YES
26	b1	1292.53	3.75864	YES	YES
27	a1	1297.27	142.31527	YES	YES
28	a1	1336.05	0.05004	YES	YES
29	b1	1494.80	11.17872	YES	YES
30	a1	1545.01	182.34382	YES	YES
31	b1	1643.79	8.42522	YES	YES
32	a1	1648.30	21.87682	YES	YES
33	b1	3183.84	1.01663	YES	YES
34	a1	3195.00	7.95116	YES	YES
35	b1	3203.19	3.01930	YES	YES
36	a1	3208.79	1.92483	YES	YES

$end

Total COSMO energy + OC corr. = -430.7042974563 H

FEC

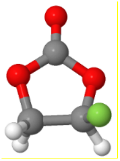

Method: (RI-)B3LYP(D3BJ)/def2-TZVPP
Symmetry: c1

Cartesian coordinates in Ångström:

O	0.5785664	0.5153183	1.0656930
C	-0.5601051	-0.1828804	1.4015083
O	-0.9442397	-0.3431656	2.5080071
O	-1.1648321	-0.6296079	0.2777944
C	-0.4639777	-0.1532439	-0.8743414
C	0.8463990	0.3727613	-0.2971538
H	-1.0484177	0.6330904	-1.3515132
H	-0.2983204	-0.9769089	-1.5633380
H	1.2016118	1.3177881	-0.6999045
F	1.8533156	-0.5531513	-0.4667519

SCF energy GEOOPT = -441.6458172085 H
ZPE = 175.9 kJ/mol
FREEH energy = 190.16 kJ/mol
FREEH entropy = 0.31861 kJ/mol/K

$vibrational spectrum

# mode	symmetry	wave number	IR intensity	selection rules	
#		cm**(-1)	km/mol	IR	RAMAN

1		-0.00	0.00000	-	-
2		-0.00	0.00000	-	-
3		-0.00	0.00000	-	-
4		0.00	0.00000	-	-
5		0.00	0.00000	-	-
6		0.00	0.00000	-	-
7	a	114.22	2.67107	YES	YES
8	a	174.18	1.42100	YES	YES
9	a	394.14	5.47923	YES	YES
10	a	478.66	2.99476	YES	YES
11	a	550.93	2.82832	YES	YES
12	a	735.25	9.16122	YES	YES
13	a	763.67	12.62708	YES	YES
14	a	824.39	14.49530	YES	YES
15	a	866.35	28.95941	YES	YES
16	a	900.01	9.72105	YES	YES
17	a	1011.27	176.36672	YES	YES
18	a	1076.47	43.88157	YES	YES
19	a	1080.70	195.48302	YES	YES
20	a	1123.81	55.33382	YES	YES
21	a	1150.12	252.87538	YES	YES
22	a	1239.76	25.40572	YES	YES
23	a	1365.19	22.23513	YES	YES
24	a	1381.06	30.15671	YES	YES
25	a	1408.99	16.35175	YES	YES
26	a	1508.42	8.90040	YES	YES
27	a	1920.14	621.43411	YES	YES
28	a	3071.37	17.15924	YES	YES
29	a	3124.85	19.88355	YES	YES
30	a	3141.69	5.69841	YES	YES

$end

Total COSMO energy + OC corr. = -441.6598276763 H

DME

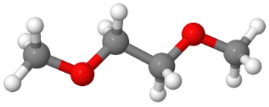

Method: (RI-)B3LYP(D3BJ)/def2-TZVPP
Symmetry: c2h

Cartesian coordinates in Ångström:

C	-0.6129658	-0.4447301	0.0000000
O	-0.1733562	-1.7867083	0.0000000
O	0.1733562	1.7867083	0.0000000
C	0.6129658	0.4447301	0.0000000
C	-1.2378354	-2.7113275	0.0000000
H	-1.2252917	-0.2346100	0.8864595
H	-1.2252917	-0.2346100	-0.8864595
C	1.2378354	2.7113275	0.0000000
H	1.2252917	0.2346100	0.8864595
H	1.2252917	0.2346100	-0.8864595
H	0.8032211	3.7090119	0.0000000
H	1.8686790	2.5952292	-0.8899749
H	1.8686790	2.5952292	0.8899749
H	-0.8032211	-3.7090119	0.0000000
H	-1.8686790	-2.5952292	-0.8899749

```
H    -1.8686790   -2.5952292    0.8899749

SCF energy GEOOPT =  -308.8247293747 H
ZPE =   369.7 kJ/mol
FREEH energy =   390.81 kJ/mol
FREEH entropy =   0.35901 kJ/mol/K

$vibrational spectrum
#  mode     symmetry     wave number   IR intensity    selection rules
#                         cm**(-1)        km/mol         IR     RAMAN
     1                      -0.00          0.00000         -       -
     2                      -0.00          0.00000         -       -
     3                      -0.00          0.00000         -       -
     4                       0.00          0.00000         -       -
     5                       0.00          0.00000         -       -
     6                       0.00          0.00000         -       -
     7        au            70.44          3.74262        YES     NO
     8        au            98.61          6.56529        YES     NO
     9        bg           124.23          0.00000        NO      YES
    10        bu           148.22          3.55386        YES     NO
    11        bg           222.15          0.00000        NO      YES
    12        au           235.34          7.38437        YES     NO
    13        ag           331.71          0.00000        NO      YES
    14        ag           396.31          0.00000        NO      YES
    15        bu           506.26          3.75871        YES     NO
    16        au           840.56          0.31966        YES     NO
    17        bu           959.21         62.89096        YES     NO
    18        ag          1013.77          0.00000        NO      YES
    19        ag          1080.82          0.00000        NO      YES
    20        bg          1153.02          0.00000        NO      YES
    21        bu          1157.00        298.94569        YES     NO
    22        ag          1158.33          0.00000        NO      YES
    23        au          1177.49          3.62455        YES     NO
    24        bg          1191.59          0.00000        NO      YES
    25        bu          1217.32        102.85925        YES     NO
    26        au          1237.07         12.41457        YES     NO
    27        ag          1238.26          0.00000        NO      YES
    28        bg          1299.47          0.00000        NO      YES
    29        bu          1369.31         22.76144        YES     NO
    30        ag          1443.78          0.00000        NO      YES
    31        bu          1477.46          1.75901        YES     NO
    32        ag          1483.54          0.00000        NO      YES
    33        au          1487.18         13.69368        YES     NO
    34        bg          1487.24          0.00000        NO      YES
    35        ag          1504.17          0.00000        NO      YES
    36        bu          1504.79         19.42549        YES     NO
    37        ag          1524.67          0.00000        NO      YES
    38        bu          1533.18          3.53410        YES     NO
    39        ag          2965.63          0.00000        NO      YES
    40        bu          2967.73         77.82758        YES     NO
    41        ag          2976.12          0.00000        NO      YES
    42        bu          2978.52        150.51578        YES     NO
    43        bg          2988.51          0.00000        NO      YES
    44        au          3012.28        209.43952        YES     NO
    45        bg          3013.25          0.00000        NO      YES
    46        au          3017.19         17.44019        YES     NO
    47        bu          3112.42         58.20076        YES     NO
    48        ag          3112.54          0.00000        NO      YES
$end


Total COSMO energy + OC corr. = -308.8313777376 H
```

[*pf*]$^-$

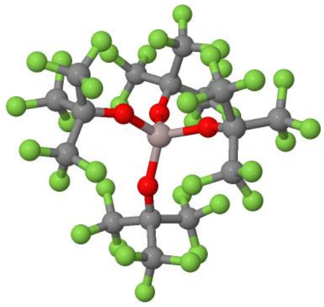

```
Method: (RI-)B3LYP(D3BJ)/def2-TZVPP
Symmetry: c1

Cartesian coordinates in Ångström:
Al   -0.0002412   -0.0059233    0.0063119
O    -0.0205545   -1.5733443   -0.7441850
O     1.6479720    0.3212398    0.4493780
O    -0.5403649    1.2632199   -1.0478665
O    -1.0822857   -0.0708914    1.3655046
C     2.8179225   -0.1437478    0.9134364
F     2.5285057   -2.5308925    0.8296943
C     2.6475249   -1.5063518    1.6844190
C    -0.2795487    2.1394778   -2.0290639
F    -1.7129348    1.2085411   -3.7140684
C    -1.7895933    0.6120067    2.2785665
F    -2.8377107    0.1553146    4.4251147
C    -0.7483399   -2.6207168   -1.1593264
F     2.0920851    1.8934005   -2.3374794
C     0.9153868    1.6621792   -2.9360817
F     0.9398468    2.2836878   -4.1301484
F     0.8291773    0.3490779   -3.1606800
C    -1.5712856    2.2785330   -2.9162191
F    -1.5434816    3.3709626   -3.7046697
F    -2.6616556    2.3578988   -2.1530868
C     0.0777337    3.5412828   -1.4099028
F     0.6370305    4.3742543   -2.3099585
F    -1.0207213    4.1467776   -0.9325301
F     0.9325214    3.4000275   -0.3973690
F     4.8820383   -1.1060418    0.0614233
F     1.5437843   -1.4754170    2.4353941
F     3.6888614   -1.7751717    2.4949033
C     3.8153499   -0.3582206   -0.2843171
F     4.2809906    0.8167585   -0.7357276
F     3.2015621   -0.9662931   -1.2996615
F     3.2749581    2.1571823    1.3961811
C     3.4154978    0.9291548    1.8964266
F     2.7751686    0.8968642    3.0754577
F     4.7264360    0.7352810    2.1416085
F    -3.5301710    0.6861037    0.6383923
C    -2.9589093    1.4170350    1.5961097
F    -3.9206805    1.7686912    2.4735791
F    -2.5001659    2.5428783    1.0332860
F    -2.8323251   -3.1154132   -2.3127813
C    -1.9056508   -2.1584192   -2.1179268
F    -2.5250355   -1.0885829   -1.6116410
F    -1.4211647   -1.8148074   -3.3198657
F    -0.4596681   -4.5010693   -2.6755019
C     0.2110853   -3.5915819   -1.9409733
F     1.0009492   -4.2649010   -1.0903414
```

```
F     1.0018537   -2.9073794   -2.7684362
F    -0.4932244   -3.4680967    1.0645874
C    -1.3728328   -3.3904549    0.0653119
F    -2.4591050   -2.7556353    0.5262624
F    -1.7450797   -4.6468612   -0.2523127
F    -1.5692560    2.5254827    3.7657884
C    -2.4045881   -0.4255942    3.2885805
F    -1.5020789   -1.3480379    3.6242136
F    -3.4502750   -1.0624377    2.7396049
C    -0.8689362    1.6124205    3.0676295
F    -0.0717502    2.2711654    2.2211056
F    -0.0846267    0.9553847    3.9338511

SCF energy GEOOPT =  -4748.105344067 H
ZPE =    580.9 kJ/mol
FREEH energy =    725.22 kJ/mol
FREEH entropy =    1.34095 kJ/mol/K

$vibrational spectrum
#  mode     symmetry     wave number   IR intensity    selection rules
#                         cm**(-1)        km/mol          IR     RAMAN
     1                     -0.00          0.00000         -        -
     2                      0.00          0.00000         -        -
     3                      0.00          0.00000         -        -
     4                      0.00          0.00000         -        -
     5                      0.00          0.00000         -        -
     6                      0.00          0.00000         -        -
     7         a           11.44          0.00093        YES      YES
     8         a           13.99          0.00084        YES      YES
     9         a           14.50          0.00164        YES      YES
    10         a           17.16          0.00190        YES      YES
    11         a           27.03          0.10291        YES      YES
    12         a           28.09          0.00253        YES      YES
    13         a           32.83          0.01298        YES      YES
    14         a           34.21          0.01674        YES      YES
    15         a           35.87          0.05531        YES      YES
    16         a           40.96          0.06259        YES      YES
    17         a           44.11          0.06184        YES      YES
    18         a           58.62          0.00437        YES      YES
    19         a           59.59          0.00696        YES      YES
    20         a           68.85          0.10271        YES      YES
    21         a           70.79          0.15686        YES      YES
    22         a           72.15          0.17162        YES      YES
    23         a           73.56          0.04052        YES      YES
    24         a           75.43          0.03588        YES      YES
    25         a           76.15          0.13635        YES      YES
    26         a           76.64          0.07167        YES      YES
    27         a           78.67          0.00885        YES      YES
    28         a           79.90          0.03712        YES      YES
    29         a           85.14          0.03036        YES      YES
    30         a           85.67          0.06128        YES      YES
    31         a           89.01          0.00352        YES      YES
    32         a           90.60          0.05030        YES      YES
    33         a           97.30          0.77612        YES      YES
    34         a           98.19          0.65178        YES      YES
    35         a           99.38          0.95374        YES      YES
    36         a          112.71          0.00785        YES      YES
    37         a          160.56          0.06813        YES      YES
    38         a          160.90          0.09658        YES      YES
    39         a          164.77          0.32794        YES      YES
    40         a          165.85          0.02443        YES      YES
    41         a          167.47          0.68967        YES      YES
    42         a          167.79          0.71645        YES      YES
```

43	a	168.87	0.05822	YES	YES
44	a	170.66	0.59605	YES	YES
45	a	198.86	3.90970	YES	YES
46	a	200.22	3.28813	YES	YES
47	a	200.45	3.08030	YES	YES
48	a	223.91	0.00806	YES	YES
49	a	270.47	0.77605	YES	YES
50	a	270.77	0.65708	YES	YES
51	a	279.18	3.50369	YES	YES
52	a	280.97	0.00381	YES	YES
53	a	284.63	3.18769	YES	YES
54	a	284.94	3.08925	YES	YES
55	a	287.92	2.93235	YES	YES
56	a	288.59	0.22346	YES	YES
57	a	290.94	0.02506	YES	YES
58	a	291.35	0.02248	YES	YES
59	a	291.48	0.01699	YES	YES
60	a	291.84	0.00712	YES	YES
61	a	308.65	9.09793	YES	YES
62	a	309.22	7.96005	YES	YES
63	a	309.45	7.87667	YES	YES
64	a	314.33	0.00600	YES	YES
65	a	318.82	0.02078	YES	YES
66	a	319.13	0.02090	YES	YES
67	a	323.91	0.15353	YES	YES
68	a	325.02	0.02589	YES	YES
69	a	327.20	1.58930	YES	YES
70	a	327.58	1.67660	YES	YES
71	a	329.34	1.95876	YES	YES
72	a	331.94	0.08963	YES	YES
73	a	351.58	0.85457	YES	YES
74	a	351.74	0.84769	YES	YES
75	a	357.95	2.91950	YES	YES
76	a	366.42	9.63425	YES	YES
77	a	366.58	10.62537	YES	YES
78	a	369.13	0.85009	YES	YES
79	a	386.06	33.06489	YES	YES
80	a	392.49	0.48819	YES	YES
81	a	442.88	56.33493	YES	YES
82	a	454.03	65.96722	YES	YES
83	a	455.29	63.38707	YES	YES
84	a	524.94	0.08292	YES	YES
85	a	529.32	1.61856	YES	YES
86	a	529.51	4.21030	YES	YES
87	a	529.57	1.64545	YES	YES
88	a	530.09	2.23335	YES	YES
89	a	530.54	2.40793	YES	YES
90	a	530.82	7.98361	YES	YES
91	a	531.07	9.54851	YES	YES
92	a	531.11	12.39195	YES	YES
93	a	535.42	2.67229	YES	YES
94	a	536.01	2.04066	YES	YES
95	a	536.13	1.84506	YES	YES
96	a	539.05	0.01514	YES	YES
97	a	555.29	19.62564	YES	YES
98	a	560.38	19.63464	YES	YES
99	a	560.53	19.22963	YES	YES
100	a	565.68	0.39118	YES	YES
101	a	565.92	0.38177	YES	YES
102	a	566.11	0.36964	YES	YES
103	a	566.34	0.65227	YES	YES
104	a	567.18	0.37153	YES	YES
105	a	568.16	3.03901	YES	YES

106	a	569.49	12.42447	YES	YES
107	a	569.81	12.98016	YES	YES
108	a	722.06	1.62684	YES	YES
109	a	722.22	1.39875	YES	YES
110	a	722.72	0.40560	YES	YES
111	a	723.07	0.68075	YES	YES
112	a	723.82	9.18073	YES	YES
113	a	724.63	83.34360	YES	YES
114	a	724.92	81.38658	YES	YES
115	a	725.15	80.68333	YES	YES
116	a	740.39	0.01506	YES	YES
117	a	750.32	2.59178	YES	YES
118	a	751.49	4.59249	YES	YES
119	a	751.72	4.45335	YES	YES
120	a	793.15	0.02903	YES	YES
121	a	828.19	12.87184	YES	YES
122	a	836.54	20.47220	YES	YES
123	a	837.94	20.99571	YES	YES
124	a	964.66	15.35067	YES	YES
125	a	964.89	11.84399	YES	YES
126	a	968.30	5.25094	YES	YES
127	a	969.88	1.99966	YES	YES
128	a	972.85	87.65403	YES	YES
129	a	973.61	323.94270	YES	YES
130	a	976.11	358.79335	YES	YES
131	a	976.33	349.25422	YES	YES
132	a	1110.81	11.43449	YES	YES
133	a	1111.94	10.78304	YES	YES
134	a	1113.39	9.44349	YES	YES
135	a	1120.11	0.14617	YES	YES
136	a	1131.92	2.69700	YES	YES
137	a	1132.44	0.66798	YES	YES
138	a	1133.82	3.99164	YES	YES
139	a	1137.07	25.99133	YES	YES
140	a	1137.48	4.94593	YES	YES
141	a	1141.95	33.42923	YES	YES
142	a	1142.36	27.91099	YES	YES
143	a	1143.51	27.91366	YES	YES
144	a	1197.27	10.47425	YES	YES
145	a	1199.47	14.77023	YES	YES
146	a	1200.34	13.03504	YES	YES
147	a	1206.07	9.57891	YES	YES
148	a	1208.26	23.22060	YES	YES
149	a	1213.31	28.17678	YES	YES
150	a	1214.45	3.56632	YES	YES
151	a	1216.60	98.52853	YES	YES
152	a	1223.10	1455.62233	YES	YES
153	a	1223.24	1442.93445	YES	YES
154	a	1224.64	1363.00669	YES	YES
155	a	1233.54	6.14403	YES	YES
156	a	1235.64	95.42343	YES	YES
157	a	1236.28	75.59486	YES	YES
158	a	1240.16	1.25719	YES	YES
159	a	1248.90	26.95697	YES	YES
160	a	1249.79	44.08151	YES	YES
161	a	1251.48	22.82246	YES	YES
162	a	1262.20	117.72498	YES	YES
163	a	1264.22	111.48028	YES	YES
164	a	1267.88	160.72706	YES	YES
165	a	1268.81	906.50565	YES	YES
166	a	1270.75	925.66695	YES	YES
167	a	1271.65	686.55011	YES	YES
168	a	1344.95	204.70439	YES	YES

169	a	1345.61	230.06301	YES	YES
170	a	1347.72	242.84185	YES	YES
171	a	1370.87	1.97210	YES	YES

$end

Total COSMO energy + OC corr. = -4748.1482361576 H

[Li(*o*DFB)$_2$]$^+$

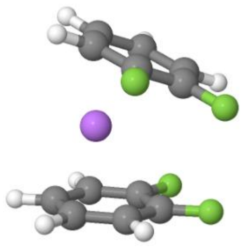

Method: (RI-)B3LYP(D3BJ)/def2-TZVPP
Symmetry: c1

Cartesian coordinates in Ångström:

C	-0.2680201	0.7779976	1.4126118
C	0.7959567	1.0690666	2.2721051
C	1.6373302	0.0566529	2.7154389
H	2.4459959	0.3065254	3.3883605
C	1.4039319	-1.2605339	2.3063428
H	2.0376186	-2.0541624	2.6762152
C	0.3406318	-1.5501609	1.4478285
H	0.1480980	-2.5694060	1.1443515
C	-0.4971083	-0.5265041	0.9949439
H	-1.3278014	-0.7242755	0.3315227
F	-1.0361943	1.7691563	0.9802720
F	1.0063662	2.3279148	2.6360113
C	2.7913713	-0.5796889	-2.0703202
H	3.1211038	-1.5488236	-2.4189511
C	3.6293487	0.2562394	-1.3235096
H	4.6342243	-0.0665571	-1.0902744
C	3.1776302	1.5113687	-0.9086387
H	3.8277467	2.1637645	-0.3433649
C	1.8911167	1.9427530	-1.2428660
H	1.5183524	2.9095893	-0.9339250
C	1.0671374	1.1150003	-1.9936166
C	1.5159492	-0.1431928	-2.4082876
F	-0.1640136	1.4945712	-2.3080111
F	0.7026161	-0.9208282	-3.1116280
Li	1.9781018	-0.2764766	0.2431488

SCF energy GEOOPT = -868.7592107939 H
ZPE = 449.5 kJ/mol
FREEH energy = 490.73 kJ/mol
FREEH entropy = 0.56617 kJ/mol/K

$vibrational spectrum

# mode	symmetry	wave number	IR intensity	selection rules	
#		cm**(-1)	km/mol	IR	RAMAN
1		-0.00	0.00000	-	-
2		0.00	0.00000	-	-
3		0.00	0.00000	-	-

4		0.00	0.00000	-	-
5		0.00	0.00000	-	-
6		0.00	0.00000	-	-
7	a	9.25	1.13040	YES	YES
8	a	20.85	5.66554	YES	YES
9	a	25.91	0.68455	YES	YES
10	a	35.11	3.32779	YES	YES
11	a	44.58	2.17431	YES	YES
12	a	102.12	2.49270	YES	YES
13	a	140.60	9.74832	YES	YES
14	a	151.71	9.59290	YES	YES
15	a	190.83	0.34419	YES	YES
16	a	194.59	0.41822	YES	YES
17	a	290.25	0.70028	YES	YES
18	a	290.47	0.32770	YES	YES
19	a	302.14	13.65366	YES	YES
20	a	312.87	0.35734	YES	YES
21	a	364.99	127.25389	YES	YES
22	a	446.19	0.20347	YES	YES
23	a	446.66	0.16768	YES	YES
24	a	473.43	0.62125	YES	YES
25	a	477.74	24.99381	YES	YES
26	a	555.83	4.28100	YES	YES
27	a	555.93	4.29158	YES	YES
28	a	579.92	0.95033	YES	YES
29	a	581.40	0.92001	YES	YES
30	a	583.82	3.66201	YES	YES
31	a	585.03	3.21500	YES	YES
32	a	732.86	0.14302	YES	YES
33	a	735.36	0.02014	YES	YES
34	a	777.49	21.89238	YES	YES
35	a	778.77	19.59731	YES	YES
36	a	811.06	208.12834	YES	YES
37	a	814.69	6.59610	YES	YES
38	a	874.09	17.01264	YES	YES
39	a	874.76	12.21360	YES	YES
40	a	892.07	0.48781	YES	YES
41	a	893.21	1.14670	YES	YES
42	a	966.34	2.67107	YES	YES
43	a	968.55	9.31473	YES	YES
44	a	1012.16	0.10976	YES	YES
45	a	1013.99	0.24622	YES	YES
46	a	1041.78	4.97181	YES	YES
47	a	1042.32	5.88797	YES	YES
48	a	1125.41	10.60135	YES	YES
49	a	1126.04	12.42654	YES	YES
50	a	1182.56	0.68556	YES	YES
51	a	1182.95	0.75335	YES	YES
52	a	1242.89	30.24072	YES	YES
53	a	1248.37	14.58558	YES	YES
54	a	1301.15	18.73817	YES	YES
55	a	1301.29	7.95300	YES	YES
56	a	1311.12	75.02770	YES	YES
57	a	1313.04	104.10008	YES	YES
58	a	1324.73	13.50642	YES	YES
59	a	1325.36	10.66164	YES	YES
60	a	1486.54	15.25300	YES	YES
61	a	1486.59	21.42125	YES	YES
62	a	1541.47	180.69512	YES	YES
63	a	1544.25	210.76035	YES	YES
64	a	1620.93	23.24550	YES	YES
65	a	1621.63	16.27874	YES	YES
66	a	1624.03	29.36031	YES	YES

67	a	1626.87	16.55566	YES	YES
68	a	3194.34	1.63773	YES	YES
69	a	3194.92	1.39673	YES	YES
70	a	3199.95	0.49731	YES	YES
71	a	3200.75	0.32921	YES	YES
72	a	3205.35	9.46112	YES	YES
73	a	3206.00	10.03159	YES	YES
74	a	3212.34	3.01578	YES	YES
75	a	3212.66	2.49047	YES	YES

$end

Total COSMO energy + OC corr. = -868.8321669343 H

$[Li(FEC)_4]^+$

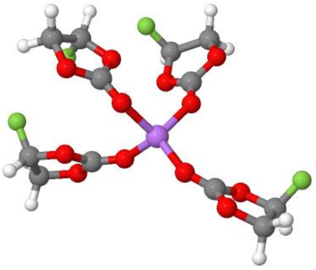

Method: (RI-)B3LYP(D3BJ)/def2-TZVPP
Symmetry: c1

Cartesian coordinates in Ångström:

O	1.1657649	2.1695478	-1.7824453
C	0.7383179	0.9447673	-2.1841966
O	-0.1066903	0.3177825	-1.6131370
O	1.3708398	0.5532578	-3.2861093
C	2.2575897	1.5972656	-3.7350695
C	2.2660835	2.5788390	-2.5652691
H	1.8504923	2.0331507	-4.6448355
H	3.2408927	1.1734262	-3.9158232
H	2.1544740	3.6294350	-2.8168919
F	3.4047293	2.4412785	-1.8142809
F	2.8904224	-2.6294366	1.6439719
H	1.4368253	-4.1112721	2.9762578
O	-0.1310308	-3.0584945	2.1338544
C	-0.0662344	-2.1596325	1.1535870
O	-0.8735542	-1.2983346	0.9606687
C	0.9623952	-3.9879342	2.0075364
C	1.8773428	-3.3012202	0.9999905
O	1.0353817	-2.3545484	0.3848481
H	2.3250944	-3.9336084	0.2395410
H	0.5766500	-4.9375410	1.6417156
F	-5.9158281	-1.6265693	1.0284107
H	-6.9649894	0.5826829	0.7602658
O	-5.1118423	1.2404759	0.1199496
C	-4.0340335	0.4934393	-0.0970903
O	-2.9059054	0.8969959	-0.0628399
C	-6.2979721	0.4473957	-0.0857238
C	-5.7537458	-0.9765443	-0.1621117
O	-4.3690855	-0.7856201	-0.3832893

```
H    -6.1552792   -1.6043541   -0.9523140
H    -6.7746341    0.7656876   -1.0105573
F     3.7760989    0.3073903    0.2815802
H     4.0766047    2.6219667    1.0628056
O     2.0466475    2.6352924    1.4484156
C     1.2146490    1.6072846    1.3218854
O     0.0407580    1.6826705    1.1000013
C     3.3733867    2.1538760    1.7447765
C     3.2628847    0.6497030    1.5043701
O     1.8698361    0.4261963    1.4786572
H     3.7142634    0.0047673    2.2518490
H     3.6103054    2.3972869    2.7782242
Li   -1.0779051    0.4132484    0.0888221

SCF energy GEOOPT =  -1774.063746570 H
ZPE =   721.0 kJ/mol
FREEH energy =   796.75 kJ/mol
FREEH entropy =   0.90600 kJ/mol/K

$vibrational spectrum
#  mode     symmetry     wave number   IR intensity    selection rules
#                         cm**(-1)        km/mol          IR     RAMAN
     1                      -0.00         0.00000         -        -
     2                       0.00         0.00000         -        -
     3                       0.00         0.00000         -        -
     4                       0.00         0.00000         -        -
     5                       0.00         0.00000         -        -
     6                       0.00         0.00000         -        -
     7         a             6.87         0.03388        YES      YES
     8         a            10.37         0.65140        YES      YES
     9         a            11.48         1.47955        YES      YES
    10         a            14.06         0.22629        YES      YES
    11         a            18.35         4.12454        YES      YES
    12         a            25.08         3.19146        YES      YES
    13         a            27.93         1.02178        YES      YES
    14         a            33.66         0.84241        YES      YES
    15         a            41.06         0.85252        YES      YES
    16         a            43.73         4.42894        YES      YES
    17         a            50.75         0.81336        YES      YES
    18         a            53.21         0.34209        YES      YES
    19         a            60.47         1.04161        YES      YES
    20         a            70.30         0.89709        YES      YES
    21         a            87.83         6.41591        YES      YES
    22         a            94.27         5.50544        YES      YES
    23         a            98.00         2.28906        YES      YES
    24         a           115.26         4.51455        YES      YES
    25         a           119.09         1.40633        YES      YES
    26         a           124.03         9.69376        YES      YES
    27         a           134.83         2.40874        YES      YES
    28         a           146.90         1.59012        YES      YES
    29         a           194.73         0.57089        YES      YES
    30         a           198.57         1.15717        YES      YES
    31         a           201.11         1.54508        YES      YES
    32         a           211.92         0.31999        YES      YES
    33         a           362.04        56.89407        YES      YES
    34         a           392.34         9.56790        YES      YES
    35         a           395.70         7.78743        YES      YES
    36         a           395.87         1.87796        YES      YES
    37         a           397.98        27.11647        YES      YES
    38         a           408.93       141.92309        YES      YES
    39         a           474.51       226.50900        YES      YES
    40         a           481.82         1.50080        YES      YES
    41         a           482.67        11.65282        YES      YES
```

42	a	483.67	3.52611	YES	YES
43	a	485.77	23.86940	YES	YES
44	a	561.27	8.88855	YES	YES
45	a	564.96	9.27137	YES	YES
46	a	565.84	3.93134	YES	YES
47	a	568.97	11.21065	YES	YES
48	a	744.81	20.53350	YES	YES
49	a	749.11	36.81880	YES	YES
50	a	752.16	28.16206	YES	YES
51	a	755.88	95.21902	YES	YES
52	a	773.28	24.96751	YES	YES
53	a	775.23	14.02876	YES	YES
54	a	776.93	26.35742	YES	YES
55	a	777.21	9.57358	YES	YES
56	a	821.51	21.25791	YES	YES
57	a	827.64	15.36555	YES	YES
58	a	828.60	17.12614	YES	YES
59	a	833.78	14.05401	YES	YES
60	a	870.33	22.04037	YES	YES
61	a	872.51	39.49236	YES	YES
62	a	874.08	5.69008	YES	YES
63	a	875.76	18.50647	YES	YES
64	a	927.28	5.96296	YES	YES
65	a	928.49	9.71655	YES	YES
66	a	929.12	9.88116	YES	YES
67	a	933.59	20.19367	YES	YES
68	a	1004.97	36.23639	YES	YES
69	a	1015.31	92.54393	YES	YES
70	a	1019.47	328.26569	YES	YES
71	a	1023.15	124.29871	YES	YES
72	a	1049.13	10.54367	YES	YES
73	a	1053.13	5.21063	YES	YES
74	a	1054.66	6.43854	YES	YES
75	a	1056.71	11.98759	YES	YES
76	a	1089.46	20.45067	YES	YES
77	a	1092.21	106.29966	YES	YES
78	a	1095.81	475.82818	YES	YES
79	a	1097.56	88.75688	YES	YES
80	a	1127.14	99.93379	YES	YES
81	a	1132.50	123.14000	YES	YES
82	a	1135.17	23.81610	YES	YES
83	a	1139.10	64.93245	YES	YES
84	a	1184.30	358.74024	YES	YES
85	a	1191.35	97.66204	YES	YES
86	a	1191.69	205.69316	YES	YES
87	a	1194.25	222.22959	YES	YES
88	a	1241.56	29.57513	YES	YES
89	a	1242.93	19.14674	YES	YES
90	a	1244.72	87.25664	YES	YES
91	a	1244.92	6.77261	YES	YES
92	a	1364.50	31.74221	YES	YES
93	a	1365.60	23.77220	YES	YES
94	a	1365.87	16.99558	YES	YES
95	a	1366.56	9.19798	YES	YES
96	a	1383.66	36.86838	YES	YES
97	a	1385.76	25.56303	YES	YES
98	a	1389.37	25.79184	YES	YES
99	a	1389.68	67.41945	YES	YES
100	a	1419.55	48.64421	YES	YES
101	a	1422.83	20.57187	YES	YES
102	a	1423.32	17.98641	YES	YES
103	a	1424.19	53.86856	YES	YES
104	a	1503.75	16.83453	YES	YES

105	a	1505.68	13.22796	YES	YES
106	a	1506.14	18.41399	YES	YES
107	a	1506.16	6.50209	YES	YES
108	a	1850.67	140.01876	YES	YES
109	a	1856.08	1825.22792	YES	YES
110	a	1859.70	856.72382	YES	YES
111	a	1897.90	393.65474	YES	YES
112	a	3091.36	6.63357	YES	YES
113	a	3092.53	7.03669	YES	YES
114	a	3094.11	6.23214	YES	YES
115	a	3094.38	7.13803	YES	YES
116	a	3139.90	9.34000	YES	YES
117	a	3141.16	8.06351	YES	YES
118	a	3146.51	7.80326	YES	YES
119	a	3147.57	7.44589	YES	YES
120	a	3160.40	1.02887	YES	YES
121	a	3160.76	1.04796	YES	YES
122	a	3160.94	0.91489	YES	YES
123	a	3162.97	1.04059	YES	YES

$end

Total COSMO energy + OC corr. = -1774.1382414109 H

[Li(DME)$_2$]$^+$

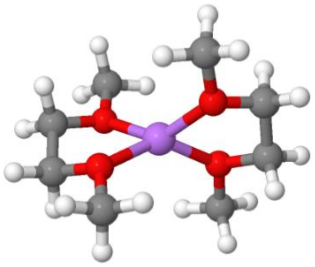

Method: (RI-)B3LYP(D3BJ)/def2-TZVPP
Symmetry: d2

Cartesian coordinates in Ångström:

C	0.4121983	2.6558917	0.6327126
O	1.1285751	1.4196574	0.6769104
Li	0.0000000	0.0000000	0.0000000
O	-1.1285751	-1.4196574	0.6769104
C	-0.4121983	-2.6558917	0.6327126
O	-1.1285751	1.4196574	-0.6769104
O	1.1285751	-1.4196574	-0.6769104
C	-0.4121983	2.6558917	-0.6327126
C	0.4121983	-2.6558917	-0.6327126
C	2.0883541	1.3446976	1.7327938
H	1.1080076	3.4987145	0.6240173
H	-0.2273151	2.7379497	1.5172047
C	-2.0883541	1.3446976	-1.7327938
H	-1.1080076	3.4987145	-0.6240173
H	0.2273151	2.7379497	-1.5172047
C	-2.0883541	-1.3446976	1.7327938
H	-1.1080076	-3.4987145	0.6240173
H	0.2273151	-2.7379497	1.5172047
C	2.0883541	-1.3446976	-1.7327938
H	1.1080076	-3.4987145	-0.6240173
H	-0.2273151	-2.7379497	-1.5172047

```
H    -2.5537323    0.3645688   -1.6708713
H    -1.6037443    1.4623912   -2.7049309
H    -2.8481906    2.1182054   -1.6093597
H     2.5537323    0.3645688    1.6708713
H     1.6037443    1.4623912    2.7049309
H     2.8481906    2.1182054    1.6093597
H     2.5537323   -0.3645688   -1.6708713
H     1.6037443   -1.4623912   -2.7049309
H     2.8481906   -2.1182054   -1.6093597
H    -2.5537323   -0.3645688    1.6708713
H    -1.6037443   -1.4623912    2.7049309
H    -2.8481906   -2.1182054    1.6093597

SCF energy GEOOPT =  -625.1071665289 H
ZPE =   757.3 kJ/mol
FREEH energy =   805.07 kJ/mol
FREEH entropy =   0.58077 kJ/mol/K

$vibrational spectrum
#  mode     symmetry     wave number   IR intensity    selection rules
#                         cm**(-1)        km/mol          IR     RAMAN
     1                       0.00         0.00000         -        -
     2                       0.00         0.00000         -        -
     3                       0.00         0.00000         -        -
     4                       0.00         0.00000         -        -
     5                       0.00         0.00000         -        -
     6                       0.00         0.00000         -        -
     7        a             16.78         0.00000        NO       YES
     8        b1            26.52         0.01599        YES      YES
     9        b3            46.94         0.04609        YES      YES
    10        b1            59.44         0.00042        YES      YES
    11        b3            64.89         0.24085        YES      YES
    12        a            100.62         0.00000        NO       YES
    13        b3           108.90         8.84584        YES      YES
    14        b2           115.88         2.34381        YES      YES
    15        b1           137.28         4.86583        YES      YES
    16        a            141.28         0.00000        NO       YES
    17        b2           166.69         0.03193        YES      YES
    18        a            167.47         0.00000        NO       YES
    19        b2           208.79         0.23263        YES      YES
    20        a            216.45         0.00000        NO       YES
    21        b3           216.71         3.56615        YES      YES
    22        b1           223.26         4.07485        YES      YES
    23        b2           277.54         2.50605        YES      YES
    24        a            283.97         0.00000        NO       YES
    25        b1           326.20        10.16984        YES      YES
    26        b3           341.78         6.72351        YES      YES
    27        b2           349.14         1.44548        YES      YES
    28        a            376.74         0.00000        NO       YES
    29        b1           390.61        33.54719        YES      YES
    30        b3           451.44        49.90294        YES      YES
    31        b1           573.59         1.97513        YES      YES
    32        b3           577.12         5.15513        YES      YES
    33        b2           588.79       147.95719        YES      YES
    34        b1           841.49        15.09643        YES      YES
    35        b3           844.16         0.25206        YES      YES
    36        b2           876.68       106.07798        YES      YES
    37        a            885.58         0.00000        NO       YES
    38        b2          1024.12        26.63053        YES      YES
    39        a           1029.05         0.00000        NO       YES
    40        b1          1039.27        15.21006        YES      YES
    41        b3          1040.60         0.40556        YES      YES
    42        b1          1096.10       254.89801        YES      YES
```

43	b3	1097.46	352.36872	YES	YES
44	a	1128.24	0.00000	NO	YES
45	b2	1128.48	7.44708	YES	YES
46	a	1131.68	0.00000	NO	YES
47	b2	1132.25	107.78418	YES	YES
48	b3	1181.00	9.51570	YES	YES
49	b1	1181.10	0.51917	YES	YES
50	a	1187.14	0.00000	NO	YES
51	b2	1187.25	3.27978	YES	YES
52	a	1213.10	0.00000	NO	YES
53	b2	1213.47	38.42017	YES	YES
54	b1	1233.79	0.41470	YES	YES
55	b3	1233.98	0.98301	YES	YES
56	b1	1267.49	4.02820	YES	YES
57	b3	1267.92	27.77089	YES	YES
58	b2	1301.49	20.78350	YES	YES
59	a	1301.71	0.00000	NO	YES
60	b1	1402.24	11.80532	YES	YES
61	b3	1404.24	16.10882	YES	YES
62	b2	1437.49	3.34488	YES	YES
63	a	1438.13	0.00000	NO	YES
64	a	1483.39	0.00000	NO	YES
65	b1	1483.63	0.00788	YES	YES
66	b3	1483.75	0.20254	YES	YES
67	b2	1484.59	1.50852	YES	YES
68	b1	1491.65	15.51361	YES	YES
69	b2	1492.72	6.10342	YES	YES
70	a	1494.36	0.00000	NO	YES
71	b3	1494.77	17.89756	YES	YES
72	b3	1501.65	20.28259	YES	YES
73	b1	1502.82	3.81830	YES	YES
74	b2	1503.96	17.78286	YES	YES
75	a	1504.97	0.00000	NO	YES
76	b1	1517.30	14.24727	YES	YES
77	b3	1517.36	0.04103	YES	YES
78	b2	1518.98	11.49638	YES	YES
79	a	1520.66	0.00000	NO	YES
80	b2	3013.23	32.91256	YES	YES
81	a	3013.25	0.00000	NO	YES
82	b1	3016.03	2.43043	YES	YES
83	b3	3016.27	27.80454	YES	YES
84	b2	3021.97	4.06252	YES	YES
85	b3	3022.05	16.96015	YES	YES
86	b1	3022.25	109.26659	YES	YES
87	a	3022.87	0.00000	NO	YES
88	b2	3060.70	38.90436	YES	YES
89	a	3060.90	0.00000	NO	YES
90	b3	3070.19	40.05560	YES	YES
91	b1	3070.23	22.68346	YES	YES
92	b2	3086.77	6.64854	YES	YES
93	b3	3087.09	37.82061	YES	YES
94	a	3087.28	0.00000	NO	YES
95	b1	3087.31	16.78122	YES	YES
96	b1	3140.64	0.88369	YES	YES
97	b2	3140.87	38.28963	YES	YES
98	a	3140.93	0.00000	NO	YES
99	b3	3140.97	3.03036	YES	YES

$end

Total COSMO energy + OC corr. = -625.1644483427 H

Li[*pf*]

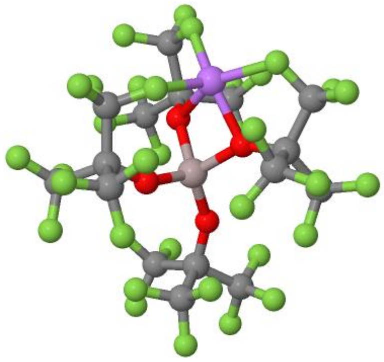

```
Method: (RI-)B3LYP(D3BJ)/def2-TZVPP
Symmetry: c1

Cartesian coordinates in Ångström:
Al     0.0995580   -0.0186840    0.0335387
O     -0.2176598   -1.6818217   -0.2392774
O      1.6196174    0.3371449    0.6994663
O     -0.4431415    0.8080160   -1.4510111
O     -1.2733391    0.6777739    0.9362805
C      2.8473478   -0.1261954    1.0458058
F      2.5506650   -2.4991328    0.8607497
C      2.7355427   -1.5229614    1.7599102
C     -0.2123203    1.8616238   -2.2838386
F     -1.4939942    1.0736387   -4.1605208
C     -1.8291069    0.9188619    2.1576865
F     -2.6917640   -0.2854167    4.0664114
C     -0.8327406   -2.6665208   -0.9189325
F      2.1676126    1.8251830   -2.4673930
C      1.0546772    1.6119973   -3.1736866
F      1.0671384    2.4284142   -4.2370169
F      1.0709197    0.3529212   -3.6052650
C     -1.4733546    1.9971490   -3.2103322
F     -1.6066802    3.1934255   -3.7623519
F     -2.6026563    1.7847635   -2.4420166
C     -0.0398895    3.1794320   -1.4484804
F      0.3870636    4.2076138   -2.1784805
F     -1.2300111    3.5138124   -0.9014505
F      0.8125014    2.9794048   -0.4459132
F      4.8185370   -1.0322412   -0.0233064
F      1.6814070   -1.5312640    2.5834744
F      3.8273900   -1.8171915    2.4732972
C      3.7424249   -0.2676750   -0.2363890
F      4.1613135    0.9302123   -0.6609189
F      3.0272467   -0.8190725   -1.2277242
F      3.2827056    2.1591633    1.5837158
C      3.4920377    0.9176013    2.0252671
F      2.9395335    0.8209455    3.2423866
F      4.8130189    0.7348928    2.1526628
F     -3.6570806    1.5141009    0.7289985
C     -3.0453907    1.8819318    1.9100085
F     -3.9687592    1.8406830    2.8561812
F     -2.6518047    3.1399657    1.7388264
F     -2.9379700   -3.0468801   -2.0792379
C     -2.0401523   -2.1227878   -1.7704180
F     -2.7106797   -1.1515663   -1.0502291
F     -1.6356763   -1.5293442   -2.8905885
F     -0.4206257   -4.1184980   -2.8082631
```

```
C     0.1944950   -3.3513784   -1.8944494
F     1.0498907   -4.1156803   -1.2126672
F     0.8984158   -2.4189346   -2.5407401
F    -0.4651826   -3.9542711    1.0563704
C    -1.3718294   -3.7258696    0.1120838
F    -2.4814878   -3.2579395    0.7095268
F    -1.6798107   -4.8909187   -0.4723113
F    -1.4418730    2.2129675    4.1435798
C    -2.3376587   -0.4233942    2.7865920
F    -1.3860457   -1.3517583    2.7050168
F    -3.4050989   -0.8646977    2.0993290
C    -0.8085903    1.6176096    3.1229841
F    -0.1108963    2.5411299    2.4613426
F     0.0492771    0.7268349    3.6224080
Li   -2.3432370    0.8195112   -0.6921205

SCF energy GEOOPT =  -4755.590505453 H
ZPE =   590.8 kJ/mol
FREEH energy =   736.40 kJ/mol
FREEH entropy =   1.32105 kJ/mol/K

$vibrational spectrum
#  mode     symmetry     wave number   IR intensity    selection rules
#                         cm**(-1)        km/mol          IR     RAMAN
     1                      -0.00          0.00000         -        -
     2                      -0.00          0.00000         -        -
     3                       0.00          0.00000         -        -
     4                       0.00          0.00000         -        -
     5                       0.00          0.00000         -        -
     6                       0.00          0.00000         -        -
     7         a            17.51          0.00426        YES      YES
     8         a            17.63          0.03313        YES      YES
     9         a            20.91          0.03580        YES      YES
    10         a            24.85          0.00658        YES      YES
    11         a            30.79          0.05072        YES      YES
    12         a            34.22          0.18983        YES      YES
    13         a            35.89          0.10071        YES      YES
    14         a            38.13          0.03152        YES      YES
    15         a            39.60          0.07289        YES      YES
    16         a            46.15          0.14718        YES      YES
    17         a            55.98          0.14349        YES      YES
    18         a            66.33          0.26961        YES      YES
    19         a            67.17          0.12514        YES      YES
    20         a            68.70          0.24710        YES      YES
    21         a            72.96          0.07480        YES      YES
    22         a            74.56          0.08652        YES      YES
    23         a            77.35          0.28649        YES      YES
    24         a            78.61          0.08676        YES      YES
    25         a            80.90          0.18221        YES      YES
    26         a            81.82          0.17978        YES      YES
    27         a            85.20          0.04066        YES      YES
    28         a            87.27          0.00513        YES      YES
    29         a            88.94          0.18651        YES      YES
    30         a            92.12          0.33228        YES      YES
    31         a            95.82          0.17059        YES      YES
    32         a            98.45          0.76321        YES      YES
    33         a           102.33          0.87926        YES      YES
    34         a           109.94          1.28759        YES      YES
    35         a           112.61          0.86466        YES      YES
    36         a           121.49          0.36626        YES      YES
    37         a           161.24          0.27390        YES      YES
    38         a           161.81          0.65218        YES      YES
    39         a           165.49          0.44083        YES      YES
```

40	a	165.85	0.17782	YES	YES
41	a	167.01	2.81517	YES	YES
42	a	169.82	0.18918	YES	YES
43	a	172.44	1.04645	YES	YES
44	a	175.49	0.11610	YES	YES
45	a	195.42	6.42375	YES	YES
46	a	197.80	2.20556	YES	YES
47	a	208.85	5.22573	YES	YES
48	a	223.70	1.78141	YES	YES
49	a	252.98	35.20218	YES	YES
50	a	271.19	0.37107	YES	YES
51	a	279.07	1.67817	YES	YES
52	a	281.81	0.80017	YES	YES
53	a	284.88	5.60298	YES	YES
54	a	287.27	1.90882	YES	YES
55	a	291.43	0.22937	YES	YES
56	a	291.98	0.85243	YES	YES
57	a	292.66	2.72834	YES	YES
58	a	293.91	0.04866	YES	YES
59	a	297.95	9.53780	YES	YES
60	a	299.32	2.08596	YES	YES
61	a	300.81	5.97661	YES	YES
62	a	309.24	4.86837	YES	YES
63	a	313.41	10.54177	YES	YES
64	a	314.26	6.44076	YES	YES
65	a	316.71	0.33435	YES	YES
66	a	321.80	1.14273	YES	YES
67	a	322.33	0.65322	YES	YES
68	a	323.35	7.64419	YES	YES
69	a	326.18	3.62687	YES	YES
70	a	329.20	0.64031	YES	YES
71	a	332.40	0.68886	YES	YES
72	a	336.58	5.01785	YES	YES
73	a	336.77	2.48803	YES	YES
74	a	343.83	14.44414	YES	YES
75	a	351.21	4.83040	YES	YES
76	a	359.46	13.77841	YES	YES
77	a	360.81	5.15440	YES	YES
78	a	368.01	19.99305	YES	YES
79	a	368.74	9.86418	YES	YES
80	a	387.25	37.81845	YES	YES
81	a	393.79	20.84794	YES	YES
82	a	419.52	66.34575	YES	YES
83	a	437.09	69.72439	YES	YES
84	a	454.41	65.70709	YES	YES
85	a	465.92	15.00743	YES	YES
86	a	488.86	59.97519	YES	YES
87	a	525.75	3.84583	YES	YES
88	a	526.25	4.03057	YES	YES
89	a	527.72	1.58638	YES	YES
90	a	529.57	9.98867	YES	YES
91	a	530.97	4.96131	YES	YES
92	a	531.83	1.33697	YES	YES
93	a	532.53	2.55738	YES	YES
94	a	532.94	5.16877	YES	YES
95	a	533.65	9.49699	YES	YES
96	a	536.74	4.32827	YES	YES
97	a	537.73	8.53043	YES	YES
98	a	537.88	5.53367	YES	YES
99	a	539.47	1.56898	YES	YES
100	a	543.16	19.91469	YES	YES
101	a	559.70	21.21187	YES	YES
102	a	565.91	0.26203	YES	YES

103	a	565.93	0.84463	YES	YES
104	a	566.31	2.34667	YES	YES
105	a	566.96	0.80818	YES	YES
106	a	567.60	3.90643	YES	YES
107	a	568.71	1.69185	YES	YES
108	a	569.97	1.53653	YES	YES
109	a	571.83	11.45183	YES	YES
110	a	585.59	32.28461	YES	YES
111	a	720.30	5.94332	YES	YES
112	a	720.91	24.29921	YES	YES
113	a	721.78	38.05348	YES	YES
114	a	724.18	1.46742	YES	YES
115	a	725.11	44.34555	YES	YES
116	a	725.68	12.63321	YES	YES
117	a	727.38	60.60307	YES	YES
118	a	727.67	60.03339	YES	YES
119	a	740.52	20.28537	YES	YES
120	a	742.26	6.09221	YES	YES
121	a	748.32	4.10797	YES	YES
122	a	756.57	1.80863	YES	YES
123	a	797.24	37.10019	YES	YES
124	a	802.76	7.28777	YES	YES
125	a	844.96	18.83931	YES	YES
126	a	890.46	16.61998	YES	YES
127	a	940.84	94.77692	YES	YES
128	a	953.84	25.85134	YES	YES
129	a	967.95	381.63343	YES	YES
130	a	972.85	22.40599	YES	YES
131	a	974.74	76.62505	YES	YES
132	a	977.90	36.35636	YES	YES
133	a	980.75	295.23325	YES	YES
134	a	984.49	318.61997	YES	YES
135	a	1071.64	10.40122	YES	YES
136	a	1080.60	45.37378	YES	YES
137	a	1090.23	4.74750	YES	YES
138	a	1116.50	1.46504	YES	YES
139	a	1144.60	4.71785	YES	YES
140	a	1147.02	22.90281	YES	YES
141	a	1156.96	64.51614	YES	YES
142	a	1162.22	45.92972	YES	YES
143	a	1166.05	22.62312	YES	YES
144	a	1170.20	17.25509	YES	YES
145	a	1177.90	9.63160	YES	YES
146	a	1182.42	33.78194	YES	YES
147	a	1190.05	87.69227	YES	YES
148	a	1195.58	195.99704	YES	YES
149	a	1198.00	64.61960	YES	YES
150	a	1200.93	81.31676	YES	YES
151	a	1208.33	33.77208	YES	YES
152	a	1209.82	39.52147	YES	YES
153	a	1211.35	100.08348	YES	YES
154	a	1220.49	20.36231	YES	YES
155	a	1221.44	77.22594	YES	YES
156	a	1226.13	459.30271	YES	YES
157	a	1228.97	59.70378	YES	YES
158	a	1233.48	538.72155	YES	YES
159	a	1237.39	287.81374	YES	YES
160	a	1239.20	49.46281	YES	YES
161	a	1247.30	948.34892	YES	YES
162	a	1258.13	171.70685	YES	YES
163	a	1259.81	870.58306	YES	YES
164	a	1264.72	967.05840	YES	YES
165	a	1267.62	842.82079	YES	YES

166	a	1272.90	73.30741	YES	YES
167	a	1277.37	430.17897	YES	YES
168	a	1281.52	536.87022	YES	YES
169	a	1287.38	497.32953	YES	YES
170	a	1291.01	333.37653	YES	YES
171	a	1323.93	2.10676	YES	YES
172	a	1329.23	33.52307	YES	YES
173	a	1345.94	130.89152	YES	YES
174	a	1361.76	103.60771	YES	YES

$end

Total COSMO energy + OC corr. = -4755.5970736175 H

[Li(DME)(FEC)$_2$]$^+$

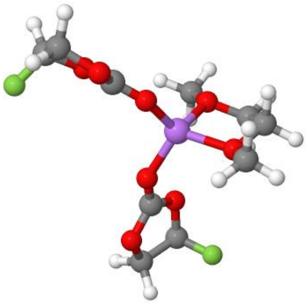

Method: (RI-)B3LYP(D3BJ)/def2-TZVPP
Symmetry: c1

Cartesian coordinates in Ångström:

O	1.6153700	2.0517937	-2.5091509
C	0.2682488	1.9771552	-2.4479318
O	-0.3313419	1.4999514	-1.5236863
O	-0.2999697	2.5101598	-3.5219986
C	0.7193622	3.0595854	-4.3842541
C	2.0093298	2.4907871	-3.7994129
H	0.6737772	4.1450534	-4.3285481
H	0.5459101	2.7152039	-5.3991420
H	2.8400149	3.1830880	-3.6982213
F	2.4326327	1.4060789	-4.5102392
F	-0.3370417	-4.2135657	2.4435569
H	0.2390638	-5.4621670	0.3971748
O	0.5698457	-3.6122688	-0.4678624
C	0.5529371	-2.4333787	0.1392848
O	0.3349343	-1.3843771	-0.3993851
C	0.9429700	-4.6392418	0.4751747
C	0.8432391	-3.9256752	1.8203551
O	0.8309093	-2.5540571	1.4611225
H	1.6543440	-4.1017540	2.5208618
H	1.9541526	-4.9698296	0.2478757
Li	-0.3319285	0.3674198	-0.0113299
H	-0.6429313	-0.3496194	2.9687148
H	1.8020643	-0.1169870	2.5734959
C	-0.7297538	0.7040621	2.6847567
C	1.6073708	0.9056503	2.2418790
H	-0.6143428	1.3216972	3.5796601
H	-2.8737808	0.6489058	2.6992557

```
O     0.2822584    1.0238940    1.7285984
O    -2.1033238    0.2559862    0.8096539
H     1.7545885    1.5951707    3.0755443
C    -2.0657183    0.9776772    2.0396437
H    -4.1639839    0.0285491    0.6933072
C    -3.3311758    0.4057364    0.0965995
H    -3.2442917   -0.1767603   -0.8164520
H    -2.1872109    2.0475897    1.8410875
H    -3.5050523    1.4545424   -0.1551483
H     2.2885236    1.1639439    1.4351598

SCF energy GEOOPT =  -1199.587411490 H
ZPE =   739.1 kJ/mol
FREEH energy =   801.10 kJ/mol
FREEH entropy =   0.76989 kJ/mol/K

$vibrational spectrum
#  mode     symmetry     wave number   IR intensity    selection rules
#                         cm**(-1)        km/mol          IR     RAMAN
     1                      -0.00          0.00000         -        -
     2                      -0.00          0.00000         -        -
     3                      -0.00          0.00000         -        -
     4                       0.00          0.00000         -        -
     5                       0.00          0.00000         -        -
     6                       0.00          0.00000         -        -
     7         a             7.87          0.30350        YES      YES
     8         a            10.26          0.56853        YES      YES
     9         a            13.63          0.13199        YES      YES
    10         a            18.27          0.98958        YES      YES
    11         a            21.04          4.43562        YES      YES
    12         a            33.59          1.00132        YES      YES
    13         a            37.80          0.44467        YES      YES
    14         a            47.65          0.71764        YES      YES
    15         a            65.58          3.64802        YES      YES
    16         a            70.34          6.11112        YES      YES
    17         a            80.70          4.41700        YES      YES
    18         a           112.95          2.47979        YES      YES
    19         a           120.74          1.57024        YES      YES
    20         a           122.48          3.64836        YES      YES
    21         a           134.65          5.78012        YES      YES
    22         a           147.56          1.35219        YES      YES
    23         a           162.51          0.36780        YES      YES
    24         a           199.83          0.11657        YES      YES
    25         a           201.49          2.02406        YES      YES
    26         a           220.78          4.77869        YES      YES
    27         a           222.41          0.37375        YES      YES
    28         a           283.56          1.26190        YES      YES
    29         a           331.08         12.34340        YES      YES
    30         a           358.06          4.87126        YES      YES
    31         a           392.64         25.98523        YES      YES
    32         a           396.06          5.79301        YES      YES
    33         a           397.87         20.64137        YES      YES
    34         a           457.58        162.09171        YES      YES
    35         a           481.36          6.22539        YES      YES
    36         a           482.97          0.21356        YES      YES
    37         a           508.40        130.78069        YES      YES
    38         a           566.40         21.20283        YES      YES
    39         a           566.91          9.17417        YES      YES
    40         a           578.12          3.91045        YES      YES
    41         a           751.63         43.46533        YES      YES
    42         a           757.24         76.97521        YES      YES
    43         a           777.20         18.72188        YES      YES
    44         a           777.78         14.77063        YES      YES
```

45	a	829.12	20.55657	YES	YES
46	a	829.37	9.74525	YES	YES
47	a	846.28	8.82698	YES	YES
48	a	875.95	15.12941	YES	YES
49	a	876.55	17.51240	YES	YES
50	a	879.24	45.13859	YES	YES
51	a	930.89	16.22398	YES	YES
52	a	935.15	24.01617	YES	YES
53	a	1016.63	216.50342	YES	YES
54	a	1019.59	157.08226	YES	YES
55	a	1028.30	11.24923	YES	YES
56	a	1043.10	4.31337	YES	YES
57	a	1044.38	2.88081	YES	YES
58	a	1044.67	16.36688	YES	YES
59	a	1092.25	180.89900	YES	YES
60	a	1096.63	116.51907	YES	YES
61	a	1101.84	323.59765	YES	YES
62	a	1125.64	13.74829	YES	YES
63	a	1137.96	30.45772	YES	YES
64	a	1139.36	87.63054	YES	YES
65	a	1141.04	63.63660	YES	YES
66	a	1181.66	3.74960	YES	YES
67	a	1188.24	4.72324	YES	YES
68	a	1195.86	212.28376	YES	YES
69	a	1199.00	196.76331	YES	YES
70	a	1215.46	19.06247	YES	YES
71	a	1233.26	0.68815	YES	YES
72	a	1245.24	33.27173	YES	YES
73	a	1245.94	57.63943	YES	YES
74	a	1267.36	15.35896	YES	YES
75	a	1302.43	9.11685	YES	YES
76	a	1365.54	17.21983	YES	YES
77	a	1365.92	24.10142	YES	YES
78	a	1389.77	40.48548	YES	YES
79	a	1390.56	47.26760	YES	YES
80	a	1402.37	15.93244	YES	YES
81	a	1424.05	39.04513	YES	YES
82	a	1426.24	44.49642	YES	YES
83	a	1438.37	2.18232	YES	YES
84	a	1484.00	0.37496	YES	YES
85	a	1485.06	0.44371	YES	YES
86	a	1491.37	14.71105	YES	YES
87	a	1492.94	10.92481	YES	YES
88	a	1500.71	9.77131	YES	YES
89	a	1503.17	9.50851	YES	YES
90	a	1505.56	13.81959	YES	YES
91	a	1505.69	15.34857	YES	YES
92	a	1516.34	5.86382	YES	YES
93	a	1517.83	5.37319	YES	YES
94	a	1847.75	1487.99557	YES	YES
95	a	1867.81	356.99068	YES	YES
96	a	3009.88	18.09152	YES	YES
97	a	3012.74	13.32531	YES	YES
98	a	3020.73	69.48099	YES	YES
99	a	3021.24	4.42072	YES	YES
100	a	3053.85	25.67841	YES	YES
101	a	3063.86	38.26622	YES	YES
102	a	3082.65	20.36534	YES	YES
103	a	3083.76	23.73653	YES	YES
104	a	3095.14	5.70439	YES	YES
105	a	3095.28	4.75556	YES	YES
106	a	3136.75	11.12627	YES	YES
107	a	3142.00	7.24964	YES	YES

108	a	3142.41	9.86093	YES	YES
109	a	3142.55	8.21215	YES	YES
110	a	3163.63	0.50088	YES	YES
111	a	3163.78	0.58930	YES	YES

$end

Total COSMO energy + OC corr. = -1199.6518418024 H

[Li(DME)(*o*DFB)]$^+$

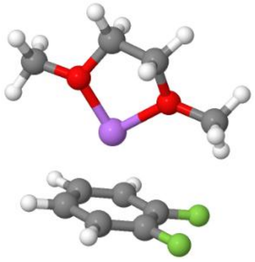

Method: (RI-)B3LYP(D3BJ)/def2-TZVPP
Symmetry: c1

Cartesian coordinates in Ångström:

C	-0.4707697	2.4104010	0.4756356
O	0.5238840	1.3974764	0.6841635
Li	-0.0383201	-0.1390172	-0.2664482
O	-1.2570135	0.9328980	-1.2151203
C	-0.9381886	2.3088310	-0.9566522
C	1.2270307	1.5345941	1.9286069
H	-0.0424330	3.3988967	0.6546133
H	-1.2980210	2.2532411	1.1738833
C	-1.9174487	0.7224536	-2.4694962
H	-1.8229401	2.9321275	-1.1029782
H	-0.1577590	2.6325977	-1.6509403
H	-2.0982729	-0.3449266	-2.5627928
H	-1.2899349	1.0646130	-3.2946497
H	-2.8687635	1.2550966	-2.4846716
H	1.9815898	0.7549064	1.9635411
H	0.5402946	1.4237522	2.7696129
H	1.7125429	2.5096090	1.9734780
H	2.6567724	-2.0066519	-1.1225638
F	3.0657026	-1.5935793	1.4063861
C	1.7776824	-2.1132869	-0.5022875
C	1.8947376	-1.9288455	0.8676962
H	0.4432554	-2.6205409	-2.1053005
C	0.5334361	-2.4500115	-1.0419055
C	0.7833308	-2.0719594	1.6996363
F	0.9249540	-1.8689052	3.0073731
C	-0.5778248	-2.5950326	-0.2095312
C	-0.4548821	-2.4030797	1.1685377
H	-1.5353063	-2.8765849	-0.6239019
H	-1.2973351	-2.5190727	1.8360761

SCF energy GEOOPT = -746.9379631741 H
ZPE = 604.2 kJ/mol
FREEH energy = 648.59 kJ/mol
FREEH entropy = 0.57455 kJ/mol/K

$vibrational spectrum

# mode	symmetry	wave number cm**(-1)	IR intensity km/mol	selection rules IR	RAMAN
1		-0.00	0.00000	-	-
2		-0.00	0.00000	-	-
3		-0.00	0.00000	-	-
4		0.00	0.00000	-	-
5		0.00	0.00000	-	-
6		0.00	0.00000	-	-
7	a	15.99	0.76495	YES	YES
8	a	28.73	0.10549	YES	YES
9	a	44.28	1.52068	YES	YES
10	a	55.51	0.03624	YES	YES
11	a	57.03	1.37583	YES	YES
12	a	80.09	2.26476	YES	YES
13	a	88.55	1.78927	YES	YES
14	a	121.77	0.28589	YES	YES
15	a	169.68	3.76101	YES	YES
16	a	177.09	7.06068	YES	YES
17	a	192.24	0.02573	YES	YES
18	a	208.30	0.64559	YES	YES
19	a	229.99	21.15023	YES	YES
20	a	282.43	0.45271	YES	YES
21	a	291.38	0.43996	YES	YES
22	a	305.09	0.73358	YES	YES
23	a	332.86	3.50249	YES	YES
24	a	340.88	15.71947	YES	YES
25	a	447.14	0.09325	YES	YES
26	a	454.13	25.51698	YES	YES
27	a	474.96	0.09867	YES	YES
28	a	515.96	164.10424	YES	YES
29	a	555.68	4.54025	YES	YES
30	a	573.91	3.29299	YES	YES
31	a	579.34	0.07929	YES	YES
32	a	584.86	5.56935	YES	YES
33	a	739.42	0.06488	YES	YES
34	a	780.79	24.42244	YES	YES
35	a	806.61	111.86938	YES	YES
36	a	837.95	6.89975	YES	YES
37	a	872.00	15.57149	YES	YES
38	a	876.61	57.88767	YES	YES
39	a	895.57	0.64689	YES	YES
40	a	975.66	4.64865	YES	YES
41	a	1013.70	0.02028	YES	YES
42	a	1022.94	11.82229	YES	YES
43	a	1035.38	4.79679	YES	YES
44	a	1042.92	4.60939	YES	YES
45	a	1080.98	295.85900	YES	YES
46	a	1119.23	63.78163	YES	YES
47	a	1127.12	12.70651	YES	YES
48	a	1127.54	0.22839	YES	YES
49	a	1180.30	3.82748	YES	YES
50	a	1181.95	1.03346	YES	YES
51	a	1186.79	0.90626	YES	YES
52	a	1210.87	19.53724	YES	YES
53	a	1233.56	0.97557	YES	YES
54	a	1241.22	27.94810	YES	YES
55	a	1268.08	12.93329	YES	YES
56	a	1299.03	9.20116	YES	YES
57	a	1303.36	11.22643	YES	YES
58	a	1308.67	116.15414	YES	YES
59	a	1325.83	4.08245	YES	YES

60	a	1404.03	11.65304	YES	YES
61	a	1438.62	1.54456	YES	YES
62	a	1485.84	0.12102	YES	YES
63	a	1487.20	3.09859	YES	YES
64	a	1489.43	15.07255	YES	YES
65	a	1490.38	9.32525	YES	YES
66	a	1494.21	14.79162	YES	YES
67	a	1503.08	15.41129	YES	YES
68	a	1505.10	7.66598	YES	YES
69	a	1516.81	12.13777	YES	YES
70	a	1519.61	7.36925	YES	YES
71	a	1543.05	190.77440	YES	YES
72	a	1627.97	27.17088	YES	YES
73	a	1629.13	12.73629	YES	YES
74	a	3023.01	14.30275	YES	YES
75	a	3026.74	12.35411	YES	YES
76	a	3031.22	27.39752	YES	YES
77	a	3034.40	19.50645	YES	YES
78	a	3073.09	11.90901	YES	YES
79	a	3082.86	20.00948	YES	YES
80	a	3100.77	10.38395	YES	YES
81	a	3103.87	12.16593	YES	YES
82	a	3144.01	11.45269	YES	YES
83	a	3159.56	5.17833	YES	YES
84	a	3192.26	1.18504	YES	YES
85	a	3198.95	0.51213	YES	YES
86	a	3204.37	5.16886	YES	YES
87	a	3211.23	0.56726	YES	YES

$end

Total COSMO energy + OC corr. = -747.0036434326 H

[Li(*o*DFB)(FEC)$_2$]$^+$

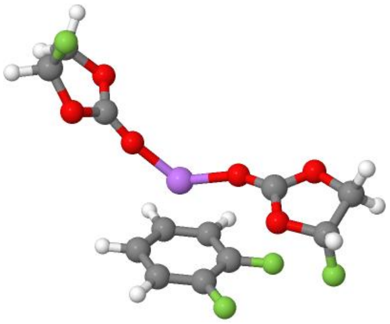

Method: (RI-)B3LYP(D3BJ)/def2-TZVPP
Symmetry: c1

Cartesian coordinates in Ångström:

O	1.0197043	1.4308814	-0.7115923
C	-0.1506159	0.7821248	-0.6212656
O	-0.5090143	0.1979963	0.3698561
O	-0.8614253	0.8775269	-1.7339431
C	-0.1593403	1.7189029	-2.6792719
C	1.2284296	1.8489701	-2.0566606
H	-0.6830781	2.6693767	-2.7504628
H	-0.1297676	1.2167872	-3.6412383

```
H     1.6656875    2.8429365   -2.0494302
F     2.1071790    0.9868002   -2.6365549
F    -0.4443035   -5.2094511    4.9844764
H    -1.4414275   -6.5981011    3.2164395
O    -1.2292827   -4.9600249    1.9691895
C    -0.6455591   -3.8162656    2.2789661
O    -0.8933200   -2.7595872    1.7511779
C    -0.6435940   -6.0255749    2.7536304
C     0.1948877   -5.2759855    3.7879017
O     0.2718575   -3.9594477    3.2469472
H     1.2039194   -5.6437878    3.9496223
H    -0.0459617   -6.6497859    2.0936490
Li   -1.0591678   -0.9355688    1.6824529
F    -0.5402708   -1.8814280    5.5497601
C    -1.3414279   -1.1680539    4.7586179
C    -1.0106134    0.1328507    4.4121206
C    -2.5058588   -1.7510318    4.2669486
C    -1.8636439    0.8569756    3.5787130
F    -2.7722721   -3.0257012    4.5737854
C    -3.3576253   -1.0395599    3.4389935
C    -3.0356742    0.2743044    3.0958887
H    -0.1027335    0.5645501    4.8094448
H    -1.6168930    1.8769277    3.3212049
H    -4.2571473   -1.5176572    3.0775061
H    -3.7058866    0.8403310    2.4647374

SCF energy GEOOPT =  -1321.423372655 H
ZPE =   585.7 kJ/mol
FREEH energy =   644.65 kJ/mol
FREEH entropy =   0.76627 kJ/mol/K

$vibrational spectrum
#  mode     symmetry     wave number   IR intensity    selection rules
#                         cm**(-1)        km/mol         IR      RAMAN
     1                      -0.00         0.00000         -        -
     2                       0.00         0.00000         -        -
     3                       0.00         0.00000         -        -
     4                       0.00         0.00000         -        -
     5                       0.00         0.00000         -        -
     6                       0.00         0.00000         -        -
     7        a              6.14         3.12231        YES      YES
     8        a              8.05         0.11797        YES      YES
     9        a             13.38         0.54868        YES      YES
    10        a             17.31         4.35239        YES      YES
    11        a             20.65         0.12906        YES      YES
    12        a             21.71         0.17893        YES      YES
    13        a             26.52         1.30327        YES      YES
    14        a             37.07         2.07724        YES      YES
    15        a             51.09         0.37037        YES      YES
    16        a             61.27         3.78639        YES      YES
    17        a             78.78         9.38608        YES      YES
    18        a            109.89         4.73391        YES      YES
    19        a            120.29         2.40364        YES      YES
    20        a            123.98         6.04475        YES      YES
    21        a            132.62        29.14489        YES      YES
    22        a            194.58         0.17515        YES      YES
    23        a            201.20         1.70110        YES      YES
    24        a            206.48         0.42357        YES      YES
    25        a            290.57         0.40137        YES      YES
    26        a            305.03         1.47764        YES      YES
    27        a            347.39       147.77826        YES      YES
    28        a            395.21         8.47763        YES      YES
    29        a            396.17         9.56666        YES      YES
```

30	a	446.61	0.07088	YES	YES
31	a	473.58	3.99167	YES	YES
32	a	475.35	27.78361	YES	YES
33	a	481.96	0.47211	YES	YES
34	a	529.36	89.95989	YES	YES
35	a	549.54	2.26658	YES	YES
36	a	555.54	4.26478	YES	YES
37	a	575.09	44.15049	YES	YES
38	a	578.23	0.93117	YES	YES
39	a	586.92	10.01043	YES	YES
40	a	735.23	0.01035	YES	YES
41	a	761.50	21.94086	YES	YES
42	a	772.67	164.39040	YES	YES
43	a	778.81	23.80743	YES	YES
44	a	779.70	30.79863	YES	YES
45	a	782.72	42.26743	YES	YES
46	a	796.04	108.85800	YES	YES
47	a	826.40	20.28735	YES	YES
48	a	831.49	24.85223	YES	YES
49	a	872.98	15.35371	YES	YES
50	a	877.98	8.77225	YES	YES
51	a	878.15	25.80856	YES	YES
52	a	886.32	0.87407	YES	YES
53	a	941.50	29.82272	YES	YES
54	a	943.00	81.94539	YES	YES
55	a	969.62	4.53966	YES	YES
56	a	1005.60	0.06684	YES	YES
57	a	1021.67	212.35270	YES	YES
58	a	1022.57	62.83165	YES	YES
59	a	1034.45	1.03254	YES	YES
60	a	1035.97	6.32177	YES	YES
61	a	1047.51	7.55361	YES	YES
62	a	1101.52	145.56867	YES	YES
63	a	1103.12	177.66910	YES	YES
64	a	1127.92	12.61704	YES	YES
65	a	1146.25	60.23889	YES	YES
66	a	1149.34	79.16971	YES	YES
67	a	1180.69	0.74218	YES	YES
68	a	1207.78	138.52534	YES	YES
69	a	1212.41	158.98048	YES	YES
70	a	1236.01	35.32060	YES	YES
71	a	1247.26	53.74393	YES	YES
72	a	1249.17	55.73673	YES	YES
73	a	1296.88	12.51374	YES	YES
74	a	1306.16	116.84167	YES	YES
75	a	1328.46	0.50930	YES	YES
76	a	1365.71	4.67907	YES	YES
77	a	1365.93	31.14159	YES	YES
78	a	1392.75	47.40974	YES	YES
79	a	1394.33	48.00082	YES	YES
80	a	1431.54	39.01022	YES	YES
81	a	1434.38	51.64842	YES	YES
82	a	1491.10	15.92270	YES	YES
83	a	1504.69	7.42418	YES	YES
84	a	1504.92	28.33828	YES	YES
85	a	1543.80	169.63250	YES	YES
86	a	1632.87	14.15476	YES	YES
87	a	1636.25	23.52884	YES	YES
88	a	1826.18	1709.73014	YES	YES
89	a	1851.73	421.55747	YES	YES
90	a	3098.64	4.22770	YES	YES
91	a	3099.61	4.15044	YES	YES
92	a	3143.33	6.89732	YES	YES

93	a	3146.43	5.39722	YES	YES
94	a	3167.58	0.64416	YES	YES
95	a	3167.62	0.42756	YES	YES
96	a	3189.31	0.64309	YES	YES
97	a	3198.65	1.83038	YES	YES
98	a	3205.20	1.64427	YES	YES
99	a	3210.21	0.05785	YES	YES

$end

Total COSMO energy + OC corr. = -1321.4941559204 H

[Li(DME)$_2$(FEC)]$^+$

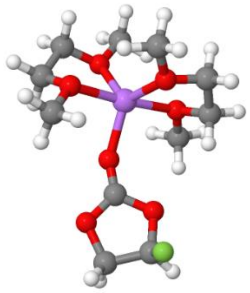

Method: (RI-)B3LYP(D3BJ)/def2-TZVPP
Symmetry: c1

Cartesian coordinates in Ångström:

C	1.2496747	2.7903671	1.4338435
O	1.7665867	1.7003803	0.6742255
Li	0.4211194	0.2736118	0.3569368
O	0.1990312	-1.2369574	1.6381125
C	1.0964211	-2.3120223	1.3718656
O	-0.8106505	1.6334633	1.3902332
O	1.7391016	-1.0719196	-0.5330006
C	-0.2225958	2.9048031	1.1344199
C	1.3217489	-2.3684164	-0.1173336
C	3.1673104	1.5076145	0.8573731
H	1.7600025	3.7194195	1.1631550
H	1.4117667	2.5998903	2.4999128
C	-2.2277345	1.6367926	1.2526231
H	-0.6715186	3.6719633	1.7724920
H	-0.3871209	3.1807545	0.0871308
C	-0.1122657	-1.1024739	3.0235821
H	0.6734551	-3.2580537	1.7225419
H	2.0407415	-2.1355477	1.8975127
C	2.1008184	-1.0074967	-1.9080411
H	2.0904235	-3.1124125	-0.3472901
H	0.4004979	-2.6431782	-0.6414628
H	-2.5707534	0.6204457	1.4315389
H	-2.5201215	1.9469431	0.2468632
H	-2.6809396	2.3069189	1.9868218
H	3.4454334	0.6240129	0.2921506
H	3.3982368	1.3502176	1.9144975
H	3.7221535	2.3750839	0.4934207
H	2.3468487	0.0283240	-2.1276714
H	1.2716064	-1.3263685	-2.5437851
H	2.9701688	-1.6393548	-2.1046133

```
H    -0.7345001   -0.2197423    3.1281380
H     0.8006956   -0.9729485    3.6111058
H    -0.6481631   -1.9844961    3.3806938
F    -3.4832524   -2.4999668   -2.2362660
H    -4.3607547   -0.4210026   -3.2296984
O    -2.6275264    0.4620862   -2.5235252
C    -1.5805272   -0.1464246   -1.9721993
O    -0.7995302    0.3709500   -1.2277170
C    -3.2937058   -0.4438361   -3.4284870
C    -2.6746754   -1.7939982   -3.0801729
O    -1.4983022   -1.4394978   -2.3730916
H    -2.4070614   -2.4327317   -3.9167480
H    -3.0821438   -0.1351963   -4.4500873

SCF energy GEOOPT =  -1066.780361997 H
ZPE =   936.4 kJ/mol
FREEH energy =  1002.99 kJ/mol
FREEH entropy =   0.75742 kJ/mol/K

$vibrational spectrum
#  mode     symmetry     wave number   IR intensity    selection rules
#                         cm**(-1)        km/mol          IR     RAMAN
     1                       -0.00         0.00000         -       -
     2                        0.00         0.00000         -       -
     3                        0.00         0.00000         -       -
     4                        0.00         0.00000         -       -
     5                        0.00         0.00000         -       -
     6                        0.00         0.00000         -       -
     7         a             13.07         1.21594        YES     YES
     8         a             15.23         0.41314        YES     YES
     9         a             27.05         2.27502        YES     YES
    10         a             30.34         0.76339        YES     YES
    11         a             39.74         1.12661        YES     YES
    12         a             49.88         0.34692        YES     YES
    13         a             58.79         0.45825        YES     YES
    14         a             86.53         1.10688        YES     YES
    15         a             94.34         0.25151        YES     YES
    16         a             98.01         1.49041        YES     YES
    17         a            108.92         3.37336        YES     YES
    18         a            112.50         3.29990        YES     YES
    19         a            116.78         0.76504        YES     YES
    20         a            128.73         6.81343        YES     YES
    21         a            135.32         2.57637        YES     YES
    22         a            157.32         0.25728        YES     YES
    23         a            164.56         0.68333        YES     YES
    24         a            169.90         3.01167        YES     YES
    25         a            179.79         4.17584        YES     YES
    26         a            199.42         0.86006        YES     YES
    27         a            221.47         1.43579        YES     YES
    28         a            227.28         3.91647        YES     YES
    29         a            232.86         2.12192        YES     YES
    30         a            235.42         1.46405        YES     YES
    31         a            282.47         2.43774        YES     YES
    32         a            288.37         0.68620        YES     YES
    33         a            340.36        29.43076        YES     YES
    34         a            345.21        26.68158        YES     YES
    35         a            350.43        12.51280        YES     YES
    36         a            357.14        14.27169        YES     YES
    37         a            363.88        21.08800        YES     YES
    38         a            396.89         3.53472        YES     YES
    39         a            411.58       125.54781        YES     YES
    40         a            467.85       111.09409        YES     YES
    41         a            483.92         2.09040        YES     YES
```

42	a	556.08	2.22715	YES	YES
43	a	579.20	3.47670	YES	YES
44	a	581.34	3.65673	YES	YES
45	a	748.47	37.96140	YES	YES
46	a	776.30	13.69104	YES	YES
47	a	830.74	12.52141	YES	YES
48	a	848.53	15.00873	YES	YES
49	a	851.73	0.85530	YES	YES
50	a	875.96	16.22450	YES	YES
51	a	877.94	84.45670	YES	YES
52	a	884.70	8.55413	YES	YES
53	a	928.07	13.09325	YES	YES
54	a	1014.54	199.66910	YES	YES
55	a	1030.15	22.38161	YES	YES
56	a	1033.92	1.88267	YES	YES
57	a	1043.34	5.30851	YES	YES
58	a	1045.25	26.13144	YES	YES
59	a	1046.09	8.06802	YES	YES
60	a	1091.36	203.63649	YES	YES
61	a	1102.52	124.46832	YES	YES
62	a	1105.84	352.93151	YES	YES
63	a	1126.77	11.82276	YES	YES
64	a	1128.47	12.04658	YES	YES
65	a	1138.11	68.88739	YES	YES
66	a	1142.07	13.27377	YES	YES
67	a	1142.56	97.63084	YES	YES
68	a	1181.05	6.29649	YES	YES
69	a	1181.93	4.32927	YES	YES
70	a	1186.44	67.42086	YES	YES
71	a	1187.08	31.04425	YES	YES
72	a	1187.32	125.16578	YES	YES
73	a	1217.56	33.17839	YES	YES
74	a	1218.24	1.67270	YES	YES
75	a	1233.17	0.36759	YES	YES
76	a	1233.69	1.80556	YES	YES
77	a	1244.90	42.22326	YES	YES
78	a	1269.35	10.48519	YES	YES
79	a	1270.82	20.20439	YES	YES
80	a	1303.76	15.18464	YES	YES
81	a	1304.20	1.48652	YES	YES
82	a	1365.42	21.15357	YES	YES
83	a	1388.47	40.86652	YES	YES
84	a	1402.02	9.43452	YES	YES
85	a	1405.50	18.35918	YES	YES
86	a	1420.92	36.79507	YES	YES
87	a	1439.63	6.09483	YES	YES
88	a	1440.85	0.61886	YES	YES
89	a	1480.03	0.35959	YES	YES
90	a	1480.84	2.09330	YES	YES
91	a	1482.75	0.06153	YES	YES
92	a	1483.79	0.32788	YES	YES
93	a	1491.50	9.48270	YES	YES
94	a	1492.90	8.56104	YES	YES
95	a	1496.82	28.73010	YES	YES
96	a	1499.10	0.43622	YES	YES
97	a	1500.58	4.31799	YES	YES
98	a	1503.72	22.09947	YES	YES
99	a	1504.83	2.51522	YES	YES
100	a	1505.58	2.23114	YES	YES
101	a	1505.92	11.83589	YES	YES
102	a	1515.90	0.81811	YES	YES
103	a	1516.42	6.63683	YES	YES
104	a	1518.32	9.18848	YES	YES

105	a	1520.37	2.38286	YES	YES
106	a	1867.20	856.46500	YES	YES
107	a	3004.79	20.04673	YES	YES
108	a	3006.42	11.51115	YES	YES
109	a	3006.99	15.12400	YES	YES
110	a	3009.02	15.80435	YES	YES
111	a	3015.20	27.11189	YES	YES
112	a	3016.13	81.46853	YES	YES
113	a	3017.92	37.23482	YES	YES
114	a	3018.85	22.00665	YES	YES
115	a	3046.76	46.58439	YES	YES
116	a	3047.23	14.33864	YES	YES
117	a	3056.53	40.96012	YES	YES
118	a	3057.99	42.80202	YES	YES
119	a	3073.26	26.31529	YES	YES
120	a	3074.31	13.42733	YES	YES
121	a	3076.71	25.59022	YES	YES
122	a	3078.25	26.20733	YES	YES
123	a	3094.34	5.53074	YES	YES
124	a	3129.23	15.86706	YES	YES
125	a	3134.81	12.07535	YES	YES
126	a	3143.26	7.00077	YES	YES
127	a	3157.93	6.98584	YES	YES
128	a	3159.76	7.20125	YES	YES
129	a	3163.50	0.46715	YES	YES

$end

Total COSMO energy + OC corr. = -1066.8381513011 H

Li(FEC)[*pf*]

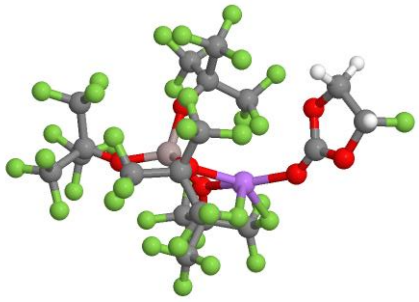

Method: (RI-)B3LYP(D3BJ)/def2-TZVPP
Symmetry: c1

Cartesian coordinates in Ångström:

Al	-0.0739993	0.2318296	-0.2185444
O	-0.5674678	-1.3508625	-0.6673445
O	1.4135605	0.2403144	0.6139777
O	-0.2231321	1.2776516	-1.6516911
O	-1.4430318	1.0701555	0.5762018
C	2.4648106	-0.4333564	1.1422126
F	1.9747158	-2.7160803	0.5853472
C	2.0432391	-1.8713462	1.6214060
C	0.3972193	2.2561871	-2.3698182
F	-0.5625863	1.7149674	-4.5005186
C	-2.0134749	1.4408189	1.7592040

F	-3.6870769	0.6055700	3.3083160
C	-1.1458688	-2.2479379	-1.4875400
F	2.7265959	1.8174667	-2.0504567
C	1.7605234	1.7635119	-2.9707662
F	2.1367114	2.5211632	-4.0112797
F	1.6489532	0.5023420	-3.3845570
C	-0.5687895	2.6401815	-3.5447013
F	-0.3001585	3.8233020	-4.0870759
F	-1.8443922	2.6970031	-3.0638295
C	0.6349302	3.5105375	-1.4571401
F	1.4246461	4.4231865	-2.0227440
F	-0.5503444	4.0933444	-1.1786803
F	1.1720599	3.1387448	-0.2950032
F	4.5113573	-1.4935531	0.4071836
F	0.8320468	-1.8308938	2.1878589
F	2.9024850	-2.3814937	2.5118371
C	3.6044854	-0.5677169	0.0685952
F	4.2457145	0.5965092	-0.0920905
F	3.0813358	-0.9116866	-1.1130194
F	3.0349339	1.6869742	2.0869589
C	3.0100992	0.3840281	2.3697325
F	2.2185127	0.2123000	3.4392050
F	4.2496874	0.0081660	2.7138502
F	-3.0550052	3.0695516	0.3519897
C	-2.5543081	2.9112643	1.5990205
F	-3.5284896	3.2017389	2.4620306
F	-1.5809278	3.8094038	1.7399479
F	-2.6969153	-2.3072222	-3.3568821
C	-1.8049107	-1.5419488	-2.7247565
F	-2.4608024	-0.4265130	-2.3175260
F	-0.8848898	-1.1492941	-3.6058772
F	-0.5000546	-3.9449698	-3.0862528
C	-0.0644869	-3.2663762	-2.0117427
F	0.2488513	-4.1561923	-1.0651425
F	1.0469813	-2.6156026	-2.3546809
F	-1.8050352	-3.3523737	0.5255585
C	-2.2495575	-3.0287717	-0.6857678
F	-3.3418230	-2.2558290	-0.5245759
F	-2.6375975	-4.1507806	-1.3073302
F	-1.4882473	2.0616585	4.0204303
C	-3.2014084	0.4653616	2.0734626
F	-2.8181482	-0.7969889	1.9106362
F	-4.2201050	0.6910579	1.2081402
C	-0.9961885	1.4195278	2.9514397
F	0.1415557	2.0105687	2.5951489
F	-0.7245130	0.1614708	3.3090589
Li	-2.2582110	1.6468278	-1.2669935
F	-8.0783640	0.8092937	-2.3783644
H	-7.0091288	-1.3258506	-1.7647001
O	-5.2843256	-0.2200490	-1.5014655
C	-5.1569055	1.1034018	-1.5117302
O	-4.1340377	1.6979775	-1.7011496
C	-6.6171571	-0.5702789	-1.0909525
C	-7.3698024	0.7519742	-1.2092541
O	-6.3363154	1.7139935	-1.2605204
H	-8.0414326	0.9988412	-0.3920839
H	-6.5787449	-0.9410105	-0.0685314

SCF energy GEOOPT = -5197.261360701 H
ZPE = 769.4 kJ/mol
FREEH energy = 934.88 kJ/mol
FREEH entropy = 1.49130 kJ/mol/K

```
$vibrational spectrum
#  mode     symmetry     wave number   IR intensity    selection rules
#                         cm**(-1)        km/mol         IR     RAMAN
     1                      -0.00         0.00000         -       -
     2                      -0.00         0.00000         -       -
     3                      -0.00         0.00000         -       -
     4                       0.00         0.00000         -       -
     5                       0.00         0.00000         -       -
     6                       0.00         0.00000         -       -
     7         a             8.99         0.88267        YES     YES
     8         a            17.57         0.38137        YES     YES
     9         a            18.97         0.22620        YES     YES
    10         a            22.12         0.42842        YES     YES
    11         a            23.26         0.02999        YES     YES
    12         a            28.69         0.48414        YES     YES
    13         a            30.75         1.53895        YES     YES
    14         a            32.18         0.46800        YES     YES
    15         a            34.13         1.27119        YES     YES
    16         a            37.42         0.93988        YES     YES
    17         a            42.79         0.49866        YES     YES
    18         a            43.15         0.43868        YES     YES
    19         a            51.40         0.17627        YES     YES
    20         a            53.19         0.07547        YES     YES
    21         a            60.71         1.73212        YES     YES
    22         a            63.37         0.40826        YES     YES
    23         a            67.98         0.05559        YES     YES
    24         a            69.55         0.61893        YES     YES
    25         a            74.76         0.12701        YES     YES
    26         a            75.59         0.12425        YES     YES
    27         a            78.24         0.14225        YES     YES
    28         a            79.07         0.72265        YES     YES
    29         a            82.45         0.03095        YES     YES
    30         a            83.68         0.20323        YES     YES
    31         a            85.79         0.05113        YES     YES
    32         a            87.59         0.65607        YES     YES
    33         a            88.20         0.18037        YES     YES
    34         a            91.48         0.18119        YES     YES
    35         a            94.59         0.05184        YES     YES
    36         a            96.39         0.17123        YES     YES
    37         a            99.92         0.65315        YES     YES
    38         a           101.36         0.17333        YES     YES
    39         a           105.31         0.38888        YES     YES
    40         a           107.81         0.98979        YES     YES
    41         a           110.42         0.67530        YES     YES
    42         a           120.70         0.43356        YES     YES
    43         a           140.99         1.65417        YES     YES
    44         a           161.36        27.04983        YES     YES
    45         a           163.12        10.04464        YES     YES
    46         a           164.48         0.97251        YES     YES
    47         a           165.18         1.28508        YES     YES
    48         a           168.58         5.65002        YES     YES
    49         a           169.94         1.10551        YES     YES
    50         a           171.52        12.38401        YES     YES
    51         a           175.01         0.71968        YES     YES
    52         a           180.52         2.49873        YES     YES
    53         a           197.48         6.71966        YES     YES
    54         a           200.51         8.05257        YES     YES
    55         a           208.11         4.80575        YES     YES
    56         a           217.55         1.57855        YES     YES
    57         a           228.49         0.78059        YES     YES
    58         a           271.80         5.10792        YES     YES
    59         a           276.09        19.23292        YES     YES
    60         a           278.03        19.96197        YES     YES
```

61	a	281.66	7.08826	YES	YES
62	a	284.07	2.89942	YES	YES
63	a	288.05	3.97113	YES	YES
64	a	291.37	0.67307	YES	YES
65	a	291.77	2.92354	YES	YES
66	a	292.36	0.32421	YES	YES
67	a	293.33	0.20560	YES	YES
68	a	295.83	3.53408	YES	YES
69	a	299.30	1.35472	YES	YES
70	a	301.47	7.35725	YES	YES
71	a	310.92	7.40489	YES	YES
72	a	313.64	5.36714	YES	YES
73	a	315.33	11.90703	YES	YES
74	a	318.49	0.55448	YES	YES
75	a	321.75	0.37589	YES	YES
76	a	322.65	0.67638	YES	YES
77	a	326.37	0.52359	YES	YES
78	a	327.76	1.64548	YES	YES
79	a	331.00	1.00640	YES	YES
80	a	332.25	1.09357	YES	YES
81	a	333.85	0.22651	YES	YES
82	a	337.41	5.00505	YES	YES
83	a	353.93	2.93994	YES	YES
84	a	355.23	2.83089	YES	YES
85	a	360.33	2.62722	YES	YES
86	a	362.77	2.69521	YES	YES
87	a	366.49	21.07489	YES	YES
88	a	386.91	12.01565	YES	YES
89	a	392.53	45.36476	YES	YES
90	a	397.44	44.23342	YES	YES
91	a	406.48	35.33972	YES	YES
92	a	437.42	27.76116	YES	YES
93	a	441.28	95.93707	YES	YES
94	a	454.25	103.79336	YES	YES
95	a	478.82	50.48122	YES	YES
96	a	494.49	19.40938	YES	YES
97	a	525.81	0.57721	YES	YES
98	a	529.02	3.43323	YES	YES
99	a	529.87	4.88473	YES	YES
100	a	530.81	3.64966	YES	YES
101	a	531.84	8.43898	YES	YES
102	a	532.18	4.79687	YES	YES
103	a	532.88	2.59760	YES	YES
104	a	533.34	8.11199	YES	YES
105	a	534.97	5.32494	YES	YES
106	a	537.94	8.52152	YES	YES
107	a	538.42	1.82090	YES	YES
108	a	539.13	2.78593	YES	YES
109	a	540.71	0.10253	YES	YES
110	a	544.71	13.58218	YES	YES
111	a	556.00	8.47650	YES	YES
112	a	559.25	22.59862	YES	YES
113	a	566.70	0.71444	YES	YES
114	a	566.90	0.60585	YES	YES
115	a	567.21	1.37719	YES	YES
116	a	567.78	1.27632	YES	YES
117	a	568.77	0.59880	YES	YES
118	a	569.80	2.11736	YES	YES
119	a	570.80	4.37983	YES	YES
120	a	571.26	0.08828	YES	YES
121	a	580.95	24.08557	YES	YES
122	a	722.79	4.02047	YES	YES
123	a	724.05	5.90739	YES	YES

124	a	724.78	19.88156	YES	YES
125	a	725.20	38.07054	YES	YES
126	a	725.68	36.45597	YES	YES
127	a	726.59	54.32750	YES	YES
128	a	728.11	37.40804	YES	YES
129	a	728.46	58.86860	YES	YES
130	a	741.09	25.10333	YES	YES
131	a	743.39	3.20134	YES	YES
132	a	751.94	14.66933	YES	YES
133	a	754.00	32.44624	YES	YES
134	a	756.51	0.78302	YES	YES
135	a	780.85	28.82866	YES	YES
136	a	794.44	33.39842	YES	YES
137	a	799.42	6.51176	YES	YES
138	a	831.31	14.02607	YES	YES
139	a	844.84	19.41912	YES	YES
140	a	874.29	19.98729	YES	YES
141	a	876.27	16.99162	YES	YES
142	a	936.49	24.94921	YES	YES
143	a	962.62	92.82619	YES	YES
144	a	969.15	15.30335	YES	YES
145	a	970.47	2.05639	YES	YES
146	a	975.60	16.21450	YES	YES
147	a	976.52	77.45458	YES	YES
148	a	978.40	323.27930	YES	YES
149	a	980.61	300.69438	YES	YES
150	a	982.48	476.60161	YES	YES
151	a	1019.42	158.21560	YES	YES
152	a	1055.80	11.08171	YES	YES
153	a	1096.56	33.12191	YES	YES
154	a	1097.25	209.93078	YES	YES
155	a	1103.79	21.09353	YES	YES
156	a	1105.50	35.76667	YES	YES
157	a	1117.91	1.87159	YES	YES
158	a	1137.76	51.25748	YES	YES
159	a	1144.81	10.46189	YES	YES
160	a	1146.12	7.89316	YES	YES
161	a	1156.30	94.53502	YES	YES
162	a	1158.06	32.76012	YES	YES
163	a	1160.59	7.42127	YES	YES
164	a	1165.60	12.30521	YES	YES
165	a	1177.03	15.65580	YES	YES
166	a	1182.16	13.75350	YES	YES
167	a	1189.19	196.63419	YES	YES
168	a	1191.50	38.67356	YES	YES
169	a	1195.53	25.76770	YES	YES
170	a	1197.86	48.08923	YES	YES
171	a	1201.87	242.44326	YES	YES
172	a	1203.64	108.95153	YES	YES
173	a	1208.00	17.91272	YES	YES
174	a	1213.47	71.38567	YES	YES
175	a	1215.54	7.53440	YES	YES
176	a	1218.47	199.85865	YES	YES
177	a	1223.34	129.43895	YES	YES
178	a	1227.59	124.37937	YES	YES
179	a	1232.22	743.84663	YES	YES
180	a	1235.25	359.67483	YES	YES
181	a	1240.35	228.42883	YES	YES
182	a	1244.00	887.08330	YES	YES
183	a	1244.41	171.58327	YES	YES
184	a	1254.62	919.49269	YES	YES
185	a	1256.45	422.61453	YES	YES
186	a	1260.33	659.15730	YES	YES

187	a	1264.65	572.47255	YES	YES
188	a	1266.77	189.95444	YES	YES
189	a	1269.58	59.31136	YES	YES
190	a	1277.42	725.57347	YES	YES
191	a	1281.83	224.51362	YES	YES
192	a	1283.17	653.84054	YES	YES
193	a	1320.18	12.35919	YES	YES
194	a	1326.51	36.27459	YES	YES
195	a	1345.36	152.86529	YES	YES
196	a	1361.99	96.79126	YES	YES
197	a	1365.94	26.46970	YES	YES
198	a	1388.56	49.11873	YES	YES
199	a	1422.92	37.96816	YES	YES
200	a	1505.14	15.68779	YES	YES
201	a	1865.20	801.28804	YES	YES
202	a	3091.34	6.68642	YES	YES
203	a	3138.85	10.41740	YES	YES
204	a	3161.16	1.13197	YES	YES

$end

Total COSMO energy + OC corr. = -5197.2760180850 H

[Li(DME)(*o*DFB)$_2$]$^+$

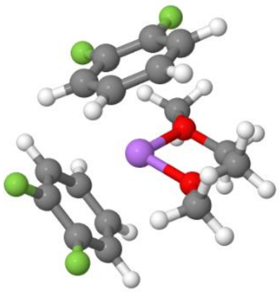

Method: (RI-)B3LYP(D3BJ)/def2-TZVPP
Symmetry: c1

Cartesian coordinates in Ångström:

C	0.1054560	0.9692867	1.7462109
C	0.8911483	0.9950277	2.8966531
C	1.2262480	-0.1837471	3.5443724
H	1.8304418	-0.1339370	4.4389298
C	0.7730887	-1.3969489	3.0316135
H	1.0210552	-2.3162590	3.5424388
C	-0.0057276	-1.4250062	1.8766303
H	-0.3668089	-2.3646848	1.4844284
C	-0.3422352	-0.2379508	1.2269256
H	-0.9568659	-0.2339684	0.3380044
F	-0.2035347	2.1213604	1.1422201
F	1.3293583	2.1702174	3.3593990
C	4.2664674	0.2708055	-2.0968603
H	5.2372372	-0.0848843	-2.4126361
C	4.0891762	1.5465498	-1.5695645
H	4.9397105	2.2066525	-1.4765635
C	2.8244398	1.9795994	-1.1862338
H	2.6817664	2.9766404	-0.7949418
C	1.7201265	1.1411080	-1.3398876
H	0.7224005	1.4761451	-1.0971941

```
C     1.9051113   -0.1307079   -1.8615563
C     3.1732876   -0.5655771   -2.2332929
F     0.8603752   -0.9645816   -1.9994895
F     3.3216145   -1.8054850   -2.7096071
Li    2.4552304   -0.3848089    0.8862444
H     3.3215003   -3.9199708   -0.4251941
H     1.7623179   -3.0737484   -0.4843830
C     2.6154658   -3.3141819    0.1429142
H     2.2861750   -3.8609032    1.0289270
O     3.2266125   -2.0748881    0.5180123
H     5.0650670   -2.9571755    0.9186560
C     4.3808049   -2.2235906    1.3508075
H     4.0698955   -2.5716208    2.3404832
C     5.0604748   -0.8768847    1.4357317
H     5.5200844   -0.6073180    0.4813355
O     4.0609489    0.0948447    1.7657147
H     5.8324775   -0.8991266    2.2081939
C     4.5950786    1.3811585    2.0978877
H     5.1710490    1.7816960    1.2624739
H     3.7543713    2.0349636    2.3112766
H     5.2303394    1.3035900    2.9810398

SCF energy GEOOPT =  -1177.657583690 H
ZPE =   829.2 kJ/mol
FREEH energy =   893.89 kJ/mol
FREEH entropy =   0.74699 kJ/mol/K

$vibrational spectrum
#  mode     symmetry     wave number   IR intensity    selection rules
#                         cm**(-1)        km/mol          IR     RAMAN
     1                      -0.00         0.00000        -        -
     2                       0.00         0.00000        -        -
     3                       0.00         0.00000        -        -
     4                       0.00         0.00000        -        -
     5                       0.00         0.00000        -        -
     6                       0.00         0.00000        -        -
     7         a            10.11         0.76056       YES      YES
     8         a            19.02         1.66429       YES      YES
     9         a            20.64         0.93167       YES      YES
    10         a            33.33         0.36939       YES      YES
    11         a            40.23         0.01739       YES      YES
    12         a            45.71         0.74265       YES      YES
    13         a            62.54         0.73969       YES      YES
    14         a            65.05         3.48878       YES      YES
    15         a            70.62         0.17681       YES      YES
    16         a            75.74         1.45847       YES      YES
    17         a            88.51         0.23092       YES      YES
    18         a           104.16         0.39050       YES      YES
    19         a           132.38         1.53676       YES      YES
    20         a           138.45         0.72041       YES      YES
    21         a           168.26         0.02612       YES      YES
    22         a           197.85         0.73223       YES      YES
    23         a           200.54         0.34124       YES      YES
    24         a           210.51         9.60231       YES      YES
    25         a           216.70         2.11097       YES      YES
    26         a           248.08       111.31150       YES      YES
    27         a           282.63         0.92098       YES      YES
    28         a           290.02         0.10335       YES      YES
    29         a           290.34         0.59960       YES      YES
    30         a           304.91         2.01605       YES      YES
    31         a           306.10         4.84039       YES      YES
    32         a           331.18         5.38293       YES      YES
    33         a           344.41         5.43264       YES      YES
```

34	a	445.73	0.23509	YES	YES
35	a	447.45	2.12816	YES	YES
36	a	448.68	15.38543	YES	YES
37	a	475.12	4.81018	YES	YES
38	a	479.21	6.66445	YES	YES
39	a	511.94	69.07822	YES	YES
40	a	554.96	4.36185	YES	YES
41	a	555.93	3.89896	YES	YES
42	a	572.26	0.86211	YES	YES
43	a	573.70	0.38757	YES	YES
44	a	575.72	0.15479	YES	YES
45	a	586.36	6.04852	YES	YES
46	a	587.96	3.93540	YES	YES
47	a	732.07	0.05163	YES	YES
48	a	733.73	0.21326	YES	YES
49	a	779.10	13.34917	YES	YES
50	a	782.01	43.88170	YES	YES
51	a	789.71	140.25460	YES	YES
52	a	792.26	72.57067	YES	YES
53	a	840.32	7.22157	YES	YES
54	a	867.68	14.95129	YES	YES
55	a	872.61	18.21167	YES	YES
56	a	880.22	48.44085	YES	YES
57	a	885.42	4.34496	YES	YES
58	a	890.93	2.22471	YES	YES
59	a	972.96	10.92266	YES	YES
60	a	976.26	9.35320	YES	YES
61	a	1012.22	0.15447	YES	YES
62	a	1013.58	0.47458	YES	YES
63	a	1024.55	14.97806	YES	YES
64	a	1039.84	7.98955	YES	YES
65	a	1050.30	4.14919	YES	YES
66	a	1051.98	0.97054	YES	YES
67	a	1087.69	232.79109	YES	YES
68	a	1124.85	39.96370	YES	YES
69	a	1128.50	12.09663	YES	YES
70	a	1129.19	4.83999	YES	YES
71	a	1131.53	26.34981	YES	YES
72	a	1180.53	4.54595	YES	YES
73	a	1182.34	1.88990	YES	YES
74	a	1183.01	0.07980	YES	YES
75	a	1186.62	1.16601	YES	YES
76	a	1215.48	13.14991	YES	YES
77	a	1227.15	37.43446	YES	YES
78	a	1235.56	29.56878	YES	YES
79	a	1238.23	2.35079	YES	YES
80	a	1268.67	10.62333	YES	YES
81	a	1293.47	28.25257	YES	YES
82	a	1298.86	14.45406	YES	YES
83	a	1302.30	50.66958	YES	YES
84	a	1304.06	12.57284	YES	YES
85	a	1306.22	122.39578	YES	YES
86	a	1323.95	0.04414	YES	YES
87	a	1330.44	0.26215	YES	YES
88	a	1404.22	6.80092	YES	YES
89	a	1438.53	1.13042	YES	YES
90	a	1487.39	1.58535	YES	YES
91	a	1488.27	0.15920	YES	YES
92	a	1491.50	4.39693	YES	YES
93	a	1492.56	2.23369	YES	YES
94	a	1493.36	21.25609	YES	YES
95	a	1493.47	25.75674	YES	YES
96	a	1501.49	5.72844	YES	YES

97	a	1503.23	9.29048	YES	YES
98	a	1514.66	16.61188	YES	YES
99	a	1517.56	17.13733	YES	YES
100	a	1541.60	17.07205	YES	YES
101	a	1544.35	281.77470	YES	YES
102	a	1633.48	8.90271	YES	YES
103	a	1635.34	20.42311	YES	YES
104	a	1636.92	4.86565	YES	YES
105	a	1641.52	19.97877	YES	YES
106	a	3019.42	14.70631	YES	YES
107	a	3028.65	12.94026	YES	YES
108	a	3029.80	27.43527	YES	YES
109	a	3034.65	26.77193	YES	YES
110	a	3069.44	13.81450	YES	YES
111	a	3080.91	25.13853	YES	YES
112	a	3100.40	10.30379	YES	YES
113	a	3100.96	19.32454	YES	YES
114	a	3152.03	4.04364	YES	YES
115	a	3153.95	5.21968	YES	YES
116	a	3191.39	0.85509	YES	YES
117	a	3191.94	0.75380	YES	YES
118	a	3200.67	2.39260	YES	YES
119	a	3201.23	0.11923	YES	YES
120	a	3207.19	2.17733	YES	YES
121	a	3208.02	0.96277	YES	YES
122	a	3212.40	0.44178	YES	YES
123	a	3214.86	1.36125	YES	YES

$end

Total COSMO energy + OC corr. = -1177.7195160757 H

[Li(*o*DFB)$_2$(FEC)]$^+$

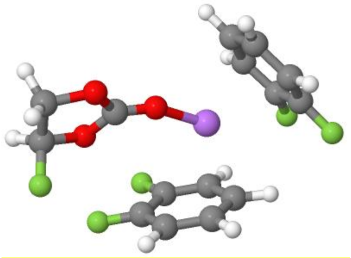

Method: (RI-)B3LYP(D3BJ)/def2-TZVPP
Symmetry: c1

Cartesian coordinates in Ångström:

C	1.0347003	0.3449702	2.5488674
C	1.3325539	-0.8881238	3.1259121
C	0.9296606	-2.0640674	2.5185706
H	1.1749490	-3.0049942	2.9906088
C	0.2052462	-2.0084187	1.3295412
H	-0.1349512	-2.9239058	0.8668021
C	-0.1147987	-0.7753756	0.7606002
H	-0.7334851	-0.7286590	-0.1248361
C	0.3058967	0.4117898	1.3714336
H	0.0405334	1.3802827	0.9703704
F	1.4491920	1.4604605	3.1488707

```
F      2.0143697   -0.9172261    4.2694123
C      1.6640668   -0.7811007   -2.9367653
H      1.3984933   -1.7261557   -3.3898191
C      2.9947307   -0.3947965   -2.7701091
H      3.7856935   -1.0557116   -3.0931159
C      3.3007638    0.8369477   -2.1929526
H      4.3307720    1.1388786   -2.0703464
C      2.2789435    1.6921942   -1.7767506
H      2.4884711    2.6561378   -1.3341622
C      0.9576274    1.3073435   -1.9533289
C      0.6514301    0.0769795   -2.5314532
F     -0.0379973    2.0994046   -1.5477039
F     -0.6288667   -0.2753338   -2.6670807
Li     2.1521552   -0.6155502   -0.1608494
F      4.5009218   -3.1134288    3.9893712
H      6.2975720   -3.8931376    2.4850556
O      5.4189721   -2.6480649    1.0817152
C      4.4457194   -1.7809660    1.2898663
O      3.6872124   -1.3878391    0.4361139
C      6.1661340   -2.8299268    2.3109835
C      5.2627212   -2.1802373    3.3553843
O      4.3955771   -1.3704748    2.5652042
H      5.7457209   -1.5536962    4.0987099
H      7.1253289   -2.3279685    2.2080800

SCF energy GEOOPT =  -1310.453599091 H
ZPE =   630.2 kJ/mol
FREEH energy =   690.39 kJ/mol
FREEH entropy =   0.74479 kJ/mol/K

$vibrational spectrum
#  mode     symmetry     wave number   IR intensity    selection rules
#                         cm**(-1)        km/mol          IR     RAMAN
     1                      -0.00         0.00000         -        -
     2                      -0.00         0.00000         -        -
     3                       0.00         0.00000         -        -
     4                       0.00         0.00000         -        -
     5                       0.00         0.00000         -        -
     6                       0.00         0.00000         -        -
     7         a             8.18         0.51589        YES      YES
     8         a             9.80         1.57224        YES      YES
     9         a            17.76         0.10504        YES      YES
    10         a            25.53         2.04980        YES      YES
    11         a            29.94         1.33972        YES      YES
    12         a            36.85         1.04037        YES      YES
    13         a            40.92         0.31290        YES      YES
    14         a            52.75         2.17806        YES      YES
    15         a            59.78         2.54288        YES      YES
    16         a            63.98         9.00010        YES      YES
    17         a            84.79         2.87363        YES      YES
    18         a           102.75         2.37027        YES      YES
    19         a           125.00         3.22666        YES      YES
    20         a           137.11        17.54069        YES      YES
    21         a           197.64         0.99322        YES      YES
    22         a           198.19         0.30025        YES      YES
    23         a           207.40         1.55501        YES      YES
    24         a           253.75       131.00053        YES      YES
    25         a           290.71         0.16131        YES      YES
    26         a           290.90         0.35240        YES      YES
    27         a           305.65         2.72952        YES      YES
    28         a           309.39         3.95516        YES      YES
    29         a           397.71         7.05848        YES      YES
    30         a           446.87         0.21304        YES      YES
```

31	a	447.19	0.47167	YES	YES
32	a	464.47	84.43872	YES	YES
33	a	473.06	4.02961	YES	YES
34	a	477.07	15.87974	YES	YES
35	a	485.92	6.16047	YES	YES
36	a	555.31	3.88674	YES	YES
37	a	556.26	4.15596	YES	YES
38	a	563.70	5.85337	YES	YES
39	a	576.01	0.01336	YES	YES
40	a	581.42	0.97692	YES	YES
41	a	585.64	3.42763	YES	YES
42	a	587.57	6.22372	YES	YES
43	a	735.00	0.03339	YES	YES
44	a	736.63	0.18198	YES	YES
45	a	767.09	82.28198	YES	YES
46	a	780.93	21.70150	YES	YES
47	a	781.26	4.40823	YES	YES
48	a	782.80	58.92700	YES	YES
49	a	796.10	124.11996	YES	YES
50	a	810.57	96.45198	YES	YES
51	a	834.95	14.23598	YES	YES
52	a	871.01	9.62246	YES	YES
53	a	873.31	19.15951	YES	YES
54	a	877.84	17.19693	YES	YES
55	a	890.08	2.52494	YES	YES
56	a	893.64	5.69431	YES	YES
57	a	940.49	54.97480	YES	YES
58	a	973.09	5.79469	YES	YES
59	a	974.95	4.41346	YES	YES
60	a	1009.46	0.11387	YES	YES
61	a	1014.69	74.22299	YES	YES
62	a	1015.38	48.18574	YES	YES
63	a	1031.78	1.93780	YES	YES
64	a	1046.19	8.49769	YES	YES
65	a	1048.31	5.19707	YES	YES
66	a	1099.55	141.43239	YES	YES
67	a	1126.67	14.93291	YES	YES
68	a	1128.51	14.88278	YES	YES
69	a	1144.26	68.88150	YES	YES
70	a	1182.30	1.28985	YES	YES
71	a	1182.85	0.13246	YES	YES
72	a	1211.22	163.19190	YES	YES
73	a	1236.10	38.43825	YES	YES
74	a	1240.23	15.54048	YES	YES
75	a	1247.57	58.80834	YES	YES
76	a	1296.86	1.60028	YES	YES
77	a	1298.71	20.61907	YES	YES
78	a	1306.53	123.39856	YES	YES
79	a	1308.27	97.51377	YES	YES
80	a	1322.88	0.55504	YES	YES
81	a	1334.78	2.35523	YES	YES
82	a	1364.70	19.90930	YES	YES
83	a	1390.18	41.06862	YES	YES
84	a	1433.19	53.68024	YES	YES
85	a	1490.42	13.30662	YES	YES
86	a	1491.21	17.49900	YES	YES
87	a	1503.72	16.61778	YES	YES
88	a	1541.76	156.63310	YES	YES
89	a	1545.56	210.58384	YES	YES
90	a	1630.08	16.02335	YES	YES
91	a	1632.30	14.89118	YES	YES
92	a	1632.86	28.57580	YES	YES
93	a	1633.94	11.59520	YES	YES

94	a	1830.51	970.05389	YES	YES
95	a	3100.82	3.10669	YES	YES
96	a	3152.91	3.25125	YES	YES
97	a	3170.89	0.53231	YES	YES
98	a	3189.76	0.48864	YES	YES
99	a	3193.10	1.59704	YES	YES
100	a	3197.97	1.08093	YES	YES
101	a	3198.64	0.89601	YES	YES
102	a	3204.09	3.02618	YES	YES
103	a	3204.60	2.77952	YES	YES
104	a	3210.06	0.78481	YES	YES
105	a	3212.98	0.21911	YES	YES

$end

Total COSMO energy + OC corr. = -1310.5223947005 H

[Li(*o*DFB)$_2$(FEC)$_2$]$^+$

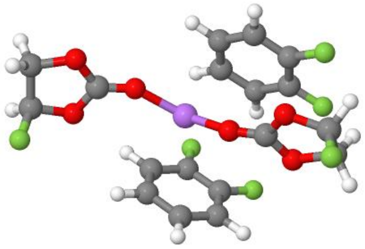

Method: (RI-)B3LYP(D3BJ)/def2-TZVPP
Symmetry: c1

Cartesian coordinates in Ångström:

C	-0.1928810	0.0604771	1.3587621
C	1.0613064	0.5228112	1.7442997
C	1.8763032	-0.2468771	2.5584894
H	2.8373618	0.1432860	2.8619893
C	1.4210089	-1.4930516	2.9924225
H	2.0471529	-2.0907550	3.6391814
C	0.1620443	-1.9481414	2.6134360
H	-0.1930233	-2.9089135	2.9569475
C	-0.6490507	-1.1698609	1.7936604
H	-1.6275204	-1.5031823	1.4800412
F	-0.9462558	0.8210318	0.5567831
F	1.4752005	1.7264531	1.3196204
C	3.0325942	-1.2936213	-3.0482305
H	3.5828606	-2.0599580	-3.5749565
C	3.6088999	-0.5486787	-2.0217984
H	4.6394134	-0.7276049	-1.7504995
C	2.8682726	0.4321868	-1.3639675
H	3.3218824	1.0338074	-0.5883063
C	1.5440112	0.6772885	-1.7258052
H	0.9515418	1.4364608	-1.2372385
C	0.9812943	-0.0640344	-2.7462111
C	1.7215905	-1.0410904	-3.4013402
F	-0.2930830	0.1339827	-3.1112733
F	1.1256608	-1.7588451	-4.3710525
Li	2.5171668	-1.7566958	0.3512441
F	5.4328173	-3.2960994	4.6401789
H	7.2813362	-3.9128895	3.1284063
O	6.1856183	-2.9761382	1.6427608

C	5.0677073	-2.2880836	1.7991042
O	4.2429046	-2.1172911	0.9392122
C	6.9687040	-2.9092621	2.8573027
C	5.9871662	-2.3210260	3.8673237
O	4.9619984	-1.7776673	3.0429177
H	6.3695986	-1.5374822	4.5146962
H	7.8283200	-2.2672999	2.6791433
O	-0.7577215	-2.4554396	-1.3876849
H	-2.4735225	-3.2155012	-2.2653764
C	-1.4645099	-2.9406435	-2.5555896
H	-1.4673238	-2.1515778	-3.3023756
O	1.2773012	-2.7747147	-0.5077926
C	0.4260773	-3.0472812	-1.3155977
C	-0.6391221	-4.1544773	-2.9745875
O	0.5817262	-3.9818821	-2.2616669
F	-1.2167862	-5.3120872	-2.5498000
H	-0.4131718	-4.2402713	-4.0327928

SCF energy GEOOPT = -1752.142636249 H
ZPE = 810.6 kJ/mol
FREEH energy = 889.64 kJ/mol
FREEH entropy = 0.89885 kJ/mol/K

$vibrational spectrum

# mode	symmetry	wave number	IR intensity	selection rules	
#		cm**(-1)	km/mol	IR	RAMAN
1		-0.00	0.00000	-	-
2		0.00	0.00000	-	-
3		0.00	0.00000	-	-
4		0.00	0.00000	-	-
5		0.00	0.00000	-	-
6		0.00	0.00000	-	-
7	a	9.49	0.28350	YES	YES
8	a	13.73	1.09966	YES	YES
9	a	19.33	0.44491	YES	YES
10	a	21.04	0.22558	YES	YES
11	a	24.45	1.76084	YES	YES
12	a	31.80	0.15673	YES	YES
13	a	35.15	1.18785	YES	YES
14	a	39.67	0.20181	YES	YES
15	a	43.34	0.17391	YES	YES
16	a	44.49	1.24535	YES	YES
17	a	52.82	1.98711	YES	YES
18	a	58.97	1.58910	YES	YES
19	a	67.95	5.61708	YES	YES
20	a	70.33	3.13795	YES	YES
21	a	76.92	2.97839	YES	YES
22	a	82.20	5.47313	YES	YES
23	a	102.39	2.47453	YES	YES
24	a	105.00	1.78446	YES	YES
25	a	121.40	3.89619	YES	YES
26	a	138.37	6.84203	YES	YES
27	a	190.28	32.83869	YES	YES
28	a	202.02	0.24087	YES	YES
29	a	202.77	1.12640	YES	YES
30	a	210.50	10.06576	YES	YES
31	a	220.30	60.48490	YES	YES
32	a	290.92	1.21115	YES	YES
33	a	292.86	0.54200	YES	YES
34	a	298.63	40.76260	YES	YES
35	a	303.93	5.24408	YES	YES
36	a	312.07	20.20264	YES	YES
37	a	396.06	4.03510	YES	YES

38	a	396.86	14.37047	YES	YES
39	a	445.47	0.03489	YES	YES
40	a	446.55	0.06526	YES	YES
41	a	470.36	3.52773	YES	YES
42	a	472.31	9.15374	YES	YES
43	a	475.17	19.68163	YES	YES
44	a	482.41	1.18156	YES	YES
45	a	534.86	69.86243	YES	YES
46	a	549.97	0.20535	YES	YES
47	a	555.03	3.71647	YES	YES
48	a	555.37	4.02558	YES	YES
49	a	573.80	0.32829	YES	YES
50	a	576.22	43.93170	YES	YES
51	a	579.15	3.25178	YES	YES
52	a	584.29	9.45821	YES	YES
53	a	586.48	4.30857	YES	YES
54	a	729.41	0.39115	YES	YES
55	a	730.94	0.14472	YES	YES
56	a	757.41	11.67925	YES	YES
57	a	765.08	118.52221	YES	YES
58	a	778.81	9.90995	YES	YES
59	a	779.61	64.87381	YES	YES
60	a	780.89	31.22526	YES	YES
61	a	783.59	71.81094	YES	YES
62	a	787.26	102.84843	YES	YES
63	a	793.84	95.66625	YES	YES
64	a	826.01	19.24521	YES	YES
65	a	829.84	16.80002	YES	YES
66	a	867.29	15.01002	YES	YES
67	a	868.82	12.83066	YES	YES
68	a	877.57	18.88056	YES	YES
69	a	879.26	13.59008	YES	YES
70	a	882.39	2.88569	YES	YES
71	a	887.10	4.77478	YES	YES
72	a	937.08	36.01264	YES	YES
73	a	942.55	78.02299	YES	YES
74	a	969.15	7.80881	YES	YES
75	a	974.42	5.32447	YES	YES
76	a	1010.58	0.24723	YES	YES
77	a	1012.03	4.43584	YES	YES
78	a	1018.16	192.28799	YES	YES
79	a	1024.26	146.63643	YES	YES
80	a	1033.67	8.47985	YES	YES
81	a	1036.75	0.49293	YES	YES
82	a	1047.80	6.57854	YES	YES
83	a	1049.64	4.66007	YES	YES
84	a	1095.67	164.93903	YES	YES
85	a	1103.58	191.25333	YES	YES
86	a	1125.75	21.55875	YES	YES
87	a	1127.90	14.06895	YES	YES
88	a	1143.44	62.54617	YES	YES
89	a	1144.91	59.54266	YES	YES
90	a	1179.53	2.07698	YES	YES
91	a	1181.33	3.12478	YES	YES
92	a	1204.03	24.94279	YES	YES
93	a	1206.25	254.46552	YES	YES
94	a	1220.92	53.66221	YES	YES
95	a	1230.00	20.80020	YES	YES
96	a	1246.95	57.81653	YES	YES
97	a	1250.48	44.25252	YES	YES
98	a	1293.16	27.89191	YES	YES
99	a	1295.24	56.15160	YES	YES
100	a	1299.30	70.82236	YES	YES

101	a	1302.83	82.51187	YES	YES
102	a	1332.68	0.13917	YES	YES
103	a	1336.90	1.45742	YES	YES
104	a	1365.39	20.29759	YES	YES
105	a	1366.57	10.31888	YES	YES
106	a	1390.69	52.00033	YES	YES
107	a	1391.93	46.23923	YES	YES
108	a	1429.07	35.45668	YES	YES
109	a	1430.78	51.32731	YES	YES
110	a	1493.39	10.58263	YES	YES
111	a	1494.60	10.74243	YES	YES
112	a	1504.65	16.03579	YES	YES
113	a	1505.67	22.13279	YES	YES
114	a	1542.27	166.69002	YES	YES
115	a	1543.21	145.14738	YES	YES
116	a	1637.08	5.13438	YES	YES
117	a	1639.88	8.18725	YES	YES
118	a	1641.40	11.30490	YES	YES
119	a	1641.69	24.28434	YES	YES
120	a	1832.69	1701.53253	YES	YES
121	a	1851.20	188.03030	YES	YES
122	a	3098.84	3.92372	YES	YES
123	a	3109.43	4.15020	YES	YES
124	a	3145.76	5.27281	YES	YES
125	a	3152.16	3.43090	YES	YES
126	a	3167.45	0.46344	YES	YES
127	a	3174.70	0.04603	YES	YES
128	a	3185.10	0.90955	YES	YES
129	a	3191.65	0.91094	YES	YES
130	a	3199.60	2.75391	YES	YES
131	a	3201.13	1.39159	YES	YES
132	a	3207.37	0.44575	YES	YES
133	a	3211.74	0.72512	YES	YES
134	a	3215.66	0.06455	YES	YES
135	a	3220.33	3.54437	YES	YES

$end

Total COSMO energy + OC corr. = -1752.2094088080 H

[Li(*o*DFB)(FEC)$_3$]$^+$

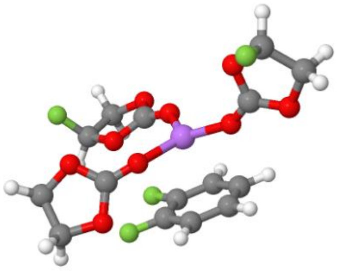

Method: (RI-)B3LYP(D3BJ)/def2-TZVPP
Symmetry: c1

Cartesian coordinates in Ångström:

O	1.5595913	-1.4758676	2.2912431
C	1.1103544	-0.5678419	1.4008964

```
O     0.0683508   -0.6812676    0.8094671
O     1.9413615    0.4554836    1.2831221
C     3.0376039    0.2981008    2.2141365
C     2.8723146   -1.1369605    2.7102043
H     2.9209113    1.0307267    3.0076127
H     3.9725013    0.4375518    1.6803539
H     2.9461991   -1.2894116    3.7819965
F     3.7486425   -1.9756738    2.0855418
F     2.2764429   -4.0691200    4.0928819
H     0.3425717   -4.9113650    5.3494655
O    -0.7723074   -4.5690881    3.6428133
C    -0.2985547   -4.1466783    2.4763154
O    -0.9337078   -3.5220024    1.6708688
C     0.2520848   -5.3068562    4.3424190
C     1.5020438   -5.0350010    3.5088657
O     0.9835036   -4.5178078    2.2976822
H     2.1338191   -5.8898742    3.2879412
H    -0.0254579   -6.3582764    4.3571107
Li   -1.3934279   -1.8749064    0.8935231
F     0.8492678    0.6048916    4.5555373
C    -0.3812437    0.2871222    4.1013445
C    -1.1275808    1.2162538    3.4030848
C    -0.8628691   -0.9954768    4.3175488
C    -2.3747111    0.8466028    2.9108972
F    -0.0930817   -1.8782648    4.9793994
C    -2.1056267   -1.3672924    3.8389034
C    -2.8624263   -0.4377912    3.1284517
H    -0.7254176    2.2072986    3.2479713
H    -2.9648392    1.5658247    2.3619578
H    -2.4640165   -2.3686634    4.0277471
H    -3.8355630   -0.7196505    2.7529141
H    -3.4919799   -2.3037087   -4.2484679
F    -4.1994216   -3.9069154   -3.2926022
C    -3.9036643   -2.5762723   -3.2811993
O    -2.9205801   -2.3473792   -2.2822104
C    -3.5044827   -1.8285426   -1.1847042
C    -5.0940123   -1.7549889   -2.7927655
O    -2.9350423   -1.6692524   -0.1375800
H    -5.1864165   -0.7997305   -3.3047952
H    -6.0230156   -2.3144908   -2.8423463
O    -4.7708879   -1.5050677   -1.4068274

SCF energy GEOOPT =  -1763.108794803 H
ZPE =   766.4 kJ/mol
FREEH energy =   843.64 kJ/mol
FREEH entropy =   0.89499 kJ/mol/K

$vibrational spectrum
#  mode     symmetry     wave number   IR intensity    selection rules
#                         cm**(-1)        km/mol         IR     RAMAN
     1                       -0.00        0.00000         -       -
     2                       -0.00        0.00000         -       -
     3                        0.00        0.00000         -       -
     4                        0.00        0.00000         -       -
     5                        0.00        0.00000         -       -
     6                        0.00        0.00000         -       -
     7        a               8.83        0.69020        YES     YES
     8        a              11.18        0.34593        YES     YES
     9        a              16.54        2.23725        YES     YES
    10        a              23.15        1.18884        YES     YES
    11        a              27.33        0.93623        YES     YES
    12        a              29.23        0.68700        YES     YES
    13        a              36.37        0.89405        YES     YES
```

14	a	38.34	0.82184	YES	YES
15	a	40.84	1.01624	YES	YES
16	a	44.10	0.61340	YES	YES
17	a	52.62	1.68575	YES	YES
18	a	55.66	3.27882	YES	YES
19	a	68.67	3.82253	YES	YES
20	a	72.55	1.85341	YES	YES
21	a	77.65	2.90134	YES	YES
22	a	85.21	2.67105	YES	YES
23	a	91.59	1.93569	YES	YES
24	a	120.10	4.69574	YES	YES
25	a	124.01	0.55397	YES	YES
26	a	131.98	2.76948	YES	YES
27	a	140.80	14.84941	YES	YES
28	a	181.67	53.55800	YES	YES
29	a	200.43	4.12568	YES	YES
30	a	200.68	0.92320	YES	YES
31	a	212.02	3.02700	YES	YES
32	a	222.67	30.67093	YES	YES
33	a	292.89	0.19460	YES	YES
34	a	302.92	0.26030	YES	YES
35	a	395.36	8.88010	YES	YES
36	a	396.50	5.68440	YES	YES
37	a	398.33	1.30662	YES	YES
38	a	444.56	0.12808	YES	YES
39	a	449.54	81.19711	YES	YES
40	a	472.05	4.13164	YES	YES
41	a	481.35	9.19977	YES	YES
42	a	483.59	0.89671	YES	YES
43	a	488.24	27.59371	YES	YES
44	a	509.12	155.95904	YES	YES
45	a	550.30	1.20754	YES	YES
46	a	553.25	4.84003	YES	YES
47	a	568.46	21.06728	YES	YES
48	a	570.19	0.78224	YES	YES
49	a	581.48	21.26451	YES	YES
50	a	584.48	14.69253	YES	YES
51	a	728.40	0.24707	YES	YES
52	a	753.38	24.93798	YES	YES
53	a	755.55	16.69257	YES	YES
54	a	762.47	161.83919	YES	YES
55	a	774.27	23.19707	YES	YES
56	a	777.12	44.61413	YES	YES
57	a	778.03	9.31813	YES	YES
58	a	779.25	66.20798	YES	YES
59	a	781.84	59.55238	YES	YES
60	a	823.74	20.82175	YES	YES
61	a	829.11	19.68969	YES	YES
62	a	829.72	22.51846	YES	YES
63	a	863.72	17.56952	YES	YES
64	a	874.74	18.16672	YES	YES
65	a	877.09	17.98170	YES	YES
66	a	878.64	12.03278	YES	YES
67	a	879.86	3.82775	YES	YES
68	a	937.05	12.95285	YES	YES
69	a	937.30	12.51349	YES	YES
70	a	938.70	63.24392	YES	YES
71	a	969.28	6.49837	YES	YES
72	a	1008.78	7.99416	YES	YES
73	a	1013.30	14.85945	YES	YES
74	a	1020.47	209.77803	YES	YES
75	a	1027.33	201.61684	YES	YES
76	a	1040.76	2.67619	YES	YES

77	a	1041.83	4.65045	YES	YES
78	a	1046.04	14.34103	YES	YES
79	a	1051.00	6.71840	YES	YES
80	a	1094.94	32.40162	YES	YES
81	a	1098.82	253.16556	YES	YES
82	a	1100.90	224.51240	YES	YES
83	a	1127.88	18.70711	YES	YES
84	a	1135.74	102.64264	YES	YES
85	a	1140.09	49.05317	YES	YES
86	a	1142.62	66.88105	YES	YES
87	a	1183.57	1.88398	YES	YES
88	a	1195.38	208.68738	YES	YES
89	a	1202.81	177.84727	YES	YES
90	a	1205.96	67.66567	YES	YES
91	a	1217.47	107.60946	YES	YES
92	a	1244.07	34.25617	YES	YES
93	a	1246.45	55.10203	YES	YES
94	a	1248.55	53.45314	YES	YES
95	a	1292.00	87.53828	YES	YES
96	a	1296.11	15.41729	YES	YES
97	a	1335.54	0.35256	YES	YES
98	a	1365.84	13.46418	YES	YES
99	a	1366.19	28.95995	YES	YES
100	a	1367.98	11.00955	YES	YES
101	a	1387.21	35.24131	YES	YES
102	a	1388.03	41.48826	YES	YES
103	a	1391.19	49.60882	YES	YES
104	a	1426.16	45.20965	YES	YES
105	a	1428.29	50.29873	YES	YES
106	a	1430.60	20.33010	YES	YES
107	a	1495.73	19.12025	YES	YES
108	a	1502.94	12.99550	YES	YES
109	a	1503.28	14.29704	YES	YES
110	a	1505.45	15.16132	YES	YES
111	a	1542.93	139.11002	YES	YES
112	a	1640.00	5.31302	YES	YES
113	a	1649.24	13.94323	YES	YES
114	a	1830.18	415.99606	YES	YES
115	a	1842.19	1929.01392	YES	YES
116	a	1870.89	233.95744	YES	YES
117	a	3096.18	5.41192	YES	YES
118	a	3097.41	5.97964	YES	YES
119	a	3107.86	3.80071	YES	YES
120	a	3144.27	6.68465	YES	YES
121	a	3147.03	7.43515	YES	YES
122	a	3155.77	3.01674	YES	YES
123	a	3165.02	0.49335	YES	YES
124	a	3165.16	0.89896	YES	YES
125	a	3172.13	0.37378	YES	YES
126	a	3193.51	0.76689	YES	YES
127	a	3202.37	2.93104	YES	YES
128	a	3210.42	0.34821	YES	YES
129	a	3215.49	0.07580	YES	YES

$end

Total COSMO energy + OC corr. = -1763.1769102975 H

[Li(*o*DFB)(FEC)(DME)]$^+$

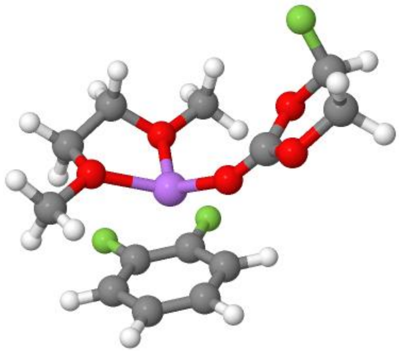

```
Method: (RI-)B3LYP(D3BJ)/def2-TZVPP
Symmetry: c1

Cartesian coordinates in Ångström:
C     1.0913562    2.1164626   -0.7113123
O     0.7520688    1.2175286    0.3470138
Li   -0.6473191    0.0370235   -0.2579819
O    -0.2765154    0.6917339   -2.0260046
C     0.9927458    1.3527469   -2.0100036
C     0.9293397    1.7767742    1.6505309
H     2.1091674    2.4909530   -0.5767539
H     0.3987218    2.9637525   -0.7028877
C    -0.6147125    0.1263578   -3.2928715
H     1.0686184    2.0446243   -2.8522468
H     1.7912683    0.6099345   -2.0913122
H    -1.5759248   -0.3673389   -3.1784714
H     0.1373804   -0.6018297   -3.6034011
H    -0.6950372    0.9118119   -4.0459390
H     0.6473379    1.0127100    2.3688320
H     0.2945381    2.6560731    1.7788424
H     1.9748858    2.0468129    1.8038294
H     1.6774074   -2.4363860   -2.3183029
F     3.2438385   -1.3552670   -0.5614649
C     1.2531709   -2.3669988   -1.3265745
C     2.0186524   -1.8282298   -0.3089610
H    -0.6353150   -3.2435511   -1.8398929
C    -0.0373192   -2.8137146   -1.0491920
C     1.5143830   -1.7452837    0.9846566
F     2.2709974   -1.2055196    1.9459106
C    -0.5461806   -2.7259719    0.2433577
C     0.2350214   -2.1912406    1.2692059
H    -1.5421597   -3.0831057    0.4616985
H    -0.1278034   -2.1282134    2.2856154
F    -3.8188779    3.5666978    2.1378764
H    -5.7913910    2.1391591    1.7493016
O    -4.4456835    0.7023282    1.1094147
C    -3.1256278    0.7737110    1.1631622
O    -2.3764062    0.1660459    0.4441639
C    -5.0165732    1.5046412    2.1683446
C    -3.8224749    2.3121070    2.6701807
O    -2.7078658    1.6053861    2.1405578
H    -3.7100831    2.3908156    3.7474551
H    -5.4250696    0.8387793    2.9251136

SCF energy GEOOPT =  -1188.624509100 H
ZPE =   784.1 kJ/mol
FREEH energy =   847.58 kJ/mol
```

FREEH entropy =   0.76616 kJ/mol/K

$vibrational spectrum

# mode	symmetry	wave number cm**(-1)	IR intensity km/mol	selection rules IR	RAMAN
1		-0.00	0.00000	-	-
2		-0.00	0.00000	-	-
3		-0.00	0.00000	-	-
4		0.00	0.00000	-	-
5		0.00	0.00000	-	-
6		0.00	0.00000	-	-
7	a	6.93	1.16580	YES	YES
8	a	10.07	0.47202	YES	YES
9	a	17.27	0.25669	YES	YES
10	a	22.85	0.88659	YES	YES
11	a	25.50	1.40582	YES	YES
12	a	34.22	0.33669	YES	YES
13	a	50.00	2.21516	YES	YES
14	a	52.23	1.29417	YES	YES
15	a	66.99	5.46912	YES	YES
16	a	77.50	1.89916	YES	YES
17	a	85.34	0.28571	YES	YES
18	a	105.84	1.44430	YES	YES
19	a	119.27	3.02768	YES	YES
20	a	136.07	7.32529	YES	YES
21	a	138.34	1.23362	YES	YES
22	a	163.91	0.31100	YES	YES
23	a	196.51	0.55963	YES	YES
24	a	201.96	0.98174	YES	YES
25	a	211.24	0.74589	YES	YES
26	a	222.47	2.51735	YES	YES
27	a	282.19	4.44672	YES	YES
28	a	290.12	0.28769	YES	YES
29	a	306.06	2.11392	YES	YES
30	a	320.05	86.67746	YES	YES
31	a	330.62	9.84238	YES	YES
32	a	374.16	51.73338	YES	YES
33	a	396.49	5.22248	YES	YES
34	a	418.76	27.20676	YES	YES
35	a	446.87	0.07540	YES	YES
36	a	474.20	3.75830	YES	YES
37	a	482.45	3.00890	YES	YES
38	a	525.24	70.74348	YES	YES
39	a	554.93	3.59540	YES	YES
40	a	573.98	19.42813	YES	YES
41	a	576.15	1.89732	YES	YES
42	a	577.87	14.20782	YES	YES
43	a	587.44	5.21991	YES	YES
44	a	733.31	0.04708	YES	YES
45	a	758.27	74.94182	YES	YES
46	a	778.81	14.43484	YES	YES
47	a	782.50	29.35756	YES	YES
48	a	788.25	111.23055	YES	YES
49	a	829.44	14.83616	YES	YES
50	a	844.25	7.87798	YES	YES
51	a	871.10	12.49073	YES	YES
52	a	877.03	17.61658	YES	YES
53	a	880.33	24.63655	YES	YES
54	a	882.70	22.62926	YES	YES
55	a	935.78	30.11184	YES	YES
56	a	966.48	6.62620	YES	YES
57	a	1006.30	1.37967	YES	YES
58	a	1017.88	165.88038	YES	YES

59	a	1028.58	23.44226	YES	YES
60	a	1038.08	0.40258	YES	YES
61	a	1040.90	9.70298	YES	YES
62	a	1049.79	5.13920	YES	YES
63	a	1094.01	82.37857	YES	YES
64	a	1096.27	339.51895	YES	YES
65	a	1127.63	19.20558	YES	YES
66	a	1129.33	14.72802	YES	YES
67	a	1131.20	35.96186	YES	YES
68	a	1142.52	63.86131	YES	YES
69	a	1181.08	3.50722	YES	YES
70	a	1182.04	0.89020	YES	YES
71	a	1187.75	1.91245	YES	YES
72	a	1204.45	186.10984	YES	YES
73	a	1214.47	12.90718	YES	YES
74	a	1233.26	33.32468	YES	YES
75	a	1236.27	2.49238	YES	YES
76	a	1246.58	51.60139	YES	YES
77	a	1268.68	12.15125	YES	YES
78	a	1296.21	6.04062	YES	YES
79	a	1302.72	95.40296	YES	YES
80	a	1304.79	32.74059	YES	YES
81	a	1332.42	0.80431	YES	YES
82	a	1365.40	19.93894	YES	YES
83	a	1391.58	42.51712	YES	YES
84	a	1404.01	10.79988	YES	YES
85	a	1428.31	45.98668	YES	YES
86	a	1439.41	1.22167	YES	YES
87	a	1485.38	0.21157	YES	YES
88	a	1487.90	3.67367	YES	YES
89	a	1488.49	8.18956	YES	YES
90	a	1491.18	13.56535	YES	YES
91	a	1493.75	6.99087	YES	YES
92	a	1500.88	9.42384	YES	YES
93	a	1503.32	9.68389	YES	YES
94	a	1504.99	12.97890	YES	YES
95	a	1516.63	9.97706	YES	YES
96	a	1519.90	11.92362	YES	YES
97	a	1546.30	179.75463	YES	YES
98	a	1638.07	8.92629	YES	YES
99	a	1641.44	23.27303	YES	YES
100	a	1845.94	1003.31202	YES	YES
101	a	3016.21	17.72636	YES	YES
102	a	3023.48	14.47850	YES	YES
103	a	3027.46	36.64705	YES	YES
104	a	3028.41	22.01877	YES	YES
105	a	3063.95	16.51998	YES	YES
106	a	3075.69	31.78046	YES	YES
107	a	3090.88	19.60335	YES	YES
108	a	3094.95	17.11960	YES	YES
109	a	3098.03	4.00263	YES	YES
110	a	3142.41	7.90956	YES	YES
111	a	3144.91	5.65492	YES	YES
112	a	3149.60	10.91219	YES	YES
113	a	3166.54	0.40767	YES	YES
114	a	3189.78	0.44973	YES	YES
115	a	3196.73	1.70698	YES	YES
116	a	3203.48	0.11109	YES	YES
117	a	3210.83	0.27787	YES	YES

$end

Total COSMO energy + OC corr. = -1188.6885414523 H

[Li(FEC)$_5$]$^+$

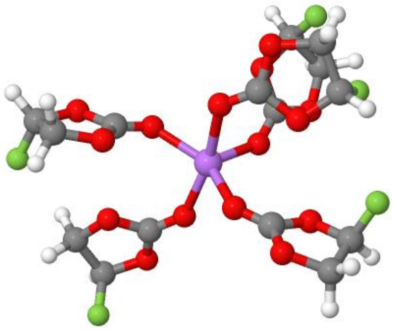

Method: (RI-)B3LYP(D3BJ)/def2-TZVPP
Symmetry: c1

Cartesian coordinates in Ångström:

O	1.7642486	1.6147362	-2.1031969
C	1.5866319	0.2694096	-2.2055917
O	0.8350351	-0.3534498	-1.5179030
O	2.3470560	-0.2406050	-3.1720520
C	3.0733137	0.8111990	-3.8313747
C	2.7927838	2.0395368	-2.9657437
H	2.6922539	0.9165769	-4.8447366
H	4.1303633	0.5613936	-3.8407522
H	2.4721446	2.9338945	-3.4924660
F	3.8898447	2.3749808	-2.2120541
F	3.8905923	-2.9253233	1.2612380
H	2.8811110	-3.9579253	3.2592211
O	1.1515245	-2.9800426	2.6877090
C	0.9251951	-2.3278426	1.5429469
O	0.1077748	-1.4718894	1.3945660
C	2.1375745	-4.0043430	2.4693235
C	2.7170282	-3.6338368	1.1093291
O	1.7470389	-2.7813779	0.5590162
H	2.9114344	-4.4527911	0.4236118
H	1.6445300	-4.9748377	2.4602154
F	-5.6144128	-3.0708757	-0.3106093
H	-5.9450884	-0.7318556	0.3832516
O	-3.9419949	-0.4846098	-0.0682479
C	-3.1262817	-1.3556476	-0.6843876
O	-1.9361250	-1.3582107	-0.6066391
C	-5.2996958	-0.7158341	-0.4891817
C	-5.2175345	-2.0793758	-1.1668331
O	-3.8396370	-2.2352597	-1.4282009
H	-5.7700506	-2.1899831	-2.0953823
H	-5.5953925	0.0777504	-1.1720628
F	4.3883687	0.0005605	-0.5364648
H	4.8816941	2.2863914	0.2470257
O	2.9697033	2.2960696	1.0257824
C	2.1114896	1.2731553	1.0435575
O	0.9230093	1.3552030	1.0546076
C	4.3208012	1.8008883	1.0393786
C	4.1482688	0.3061261	0.7845040
O	2.7877748	0.0839712	1.0478561
H	4.7489913	-0.3637420	1.3912648
H	4.7621106	2.0069915	2.0126169
Li	-0.3956366	-0.1269768	0.0134182
O	-2.4923229	1.8376309	1.6490756
H	-4.1827744	2.0661767	2.8161336
C	-3.5579744	2.6590645	2.1548793

```
H    -3.1290729    3.5067040    2.6860906
O    -1.5880286    1.4636177   -0.3759168
C    -2.4045287    1.9801743    0.3245954
C    -4.2987160    3.0781969    0.8895231
O    -3.3784701    2.8097165   -0.1385494
F    -5.4124042    2.2906738    0.6945901
H    -4.6135489    4.1158455    0.8330181

SCF energy GEOOPT =  -2215.726399604 H
ZPE =   898.8 kJ/mol
FREEH energy =   994.05 kJ/mol
FREEH entropy =   1.05860 kJ/mol/K

$vibrational spectrum
#  mode     symmetry     wave number   IR intensity    selection rules
#                         cm**(-1)        km/mol          IR     RAMAN
     1                      -0.00         0.00000         -        -
     2                      -0.00         0.00000         -        -
     3                      -0.00         0.00000         -        -
     4                       0.00         0.00000         -        -
     5                       0.00         0.00000         -        -
     6                       0.00         0.00000         -        -
     7         a             5.89         1.05258        YES      YES
     8         a            11.31         0.31838        YES      YES
     9         a            13.24         2.83535        YES      YES
    10         a            16.07         0.75923        YES      YES
    11         a            21.31         1.63526        YES      YES
    12         a            23.11         2.20361        YES      YES
    13         a            26.13         1.21364        YES      YES
    14         a            30.37         2.04966        YES      YES
    15         a            32.90         1.48424        YES      YES
    16         a            40.77         1.66389        YES      YES
    17         a            45.30         2.33819        YES      YES
    18         a            46.92         0.41527        YES      YES
    19         a            50.20         0.84656        YES      YES
    20         a            53.09         3.17040        YES      YES
    21         a            58.27         1.13673        YES      YES
    22         a            62.30         2.17079        YES      YES
    23         a            64.89         1.45901        YES      YES
    24         a            70.33         5.14673        YES      YES
    25         a            83.61         0.33619        YES      YES
    26         a            89.88         0.69216        YES      YES
    27         a           100.65         2.79004        YES      YES
    28         a           105.26         2.95681        YES      YES
    29         a           109.54         1.77055        YES      YES
    30         a           113.80         3.67337        YES      YES
    31         a           123.14         8.10901        YES      YES
    32         a           132.33         2.67127        YES      YES
    33         a           140.50         3.71796        YES      YES
    34         a           142.45         2.62859        YES      YES
    35         a           152.80         2.22285        YES      YES
    36         a           192.04         0.46209        YES      YES
    37         a           198.18         8.32774        YES      YES
    38         a           199.00         2.07044        YES      YES
    39         a           205.48         2.60273        YES      YES
    40         a           217.63         0.97719        YES      YES
    41         a           277.15       149.02161        YES      YES
    42         a           382.96        88.48875        YES      YES
    43         a           390.67        17.04701        YES      YES
    44         a           394.10        25.82671        YES      YES
    45         a           396.60         8.03516        YES      YES
    46         a           398.62        14.55349        YES      YES
    47         a           399.72        58.78853        YES      YES
```

48	a	414.81	235.94312	YES	YES
49	a	479.26	1.43773	YES	YES
50	a	483.39	2.58460	YES	YES
51	a	485.68	0.57644	YES	YES
52	a	486.28	0.34967	YES	YES
53	a	492.64	4.36037	YES	YES
54	a	555.73	1.74369	YES	YES
55	a	557.79	2.92371	YES	YES
56	a	559.60	12.72308	YES	YES
57	a	561.32	3.25822	YES	YES
58	a	565.98	2.08837	YES	YES
59	a	740.72	20.39496	YES	YES
60	a	744.42	22.43866	YES	YES
61	a	745.88	51.93557	YES	YES
62	a	749.21	30.09005	YES	YES
63	a	750.00	27.51122	YES	YES
64	a	768.15	26.33370	YES	YES
65	a	769.61	14.09313	YES	YES
66	a	771.44	25.25165	YES	YES
67	a	773.30	10.18607	YES	YES
68	a	773.66	15.25836	YES	YES
69	a	809.84	24.72141	YES	YES
70	a	829.33	16.87361	YES	YES
71	a	830.90	14.72975	YES	YES
72	a	831.75	14.78775	YES	YES
73	a	834.07	16.89638	YES	YES
74	a	867.56	23.57205	YES	YES
75	a	868.45	6.68853	YES	YES
76	a	868.80	65.13163	YES	YES
77	a	873.87	15.09030	YES	YES
78	a	874.12	13.69643	YES	YES
79	a	917.48	5.03906	YES	YES
80	a	923.00	0.43071	YES	YES
81	a	923.79	7.05349	YES	YES
82	a	925.33	14.14283	YES	YES
83	a	929.03	5.69574	YES	YES
84	a	1000.08	28.86716	YES	YES
85	a	1008.53	95.91170	YES	YES
86	a	1013.29	345.59489	YES	YES
87	a	1016.25	211.89070	YES	YES
88	a	1024.27	109.75623	YES	YES
89	a	1053.12	12.78249	YES	YES
90	a	1062.26	4.04721	YES	YES
91	a	1062.60	18.24870	YES	YES
92	a	1062.75	1.45170	YES	YES
93	a	1063.69	6.28138	YES	YES
94	a	1086.12	78.34861	YES	YES
95	a	1091.53	142.87517	YES	YES
96	a	1092.42	158.03977	YES	YES
97	a	1094.06	308.66611	YES	YES
98	a	1099.79	199.27932	YES	YES
99	a	1124.45	169.63098	YES	YES
100	a	1126.04	51.45067	YES	YES
101	a	1126.71	95.54529	YES	YES
102	a	1132.98	39.68879	YES	YES
103	a	1135.02	108.95239	YES	YES
104	a	1169.43	208.09556	YES	YES
105	a	1171.65	294.07701	YES	YES
106	a	1179.21	168.58903	YES	YES
107	a	1180.48	259.85174	YES	YES
108	a	1188.66	206.40298	YES	YES
109	a	1240.16	24.67660	YES	YES
110	a	1240.32	27.71711	YES	YES

111	a	1241.78	21.29362	YES	YES
112	a	1243.05	44.68970	YES	YES
113	a	1243.42	33.12271	YES	YES
114	a	1364.15	26.00113	YES	YES
115	a	1365.95	10.72467	YES	YES
116	a	1366.33	2.54352	YES	YES
117	a	1366.43	43.58897	YES	YES
118	a	1366.85	6.61890	YES	YES
119	a	1381.56	35.05851	YES	YES
120	a	1382.52	13.71870	YES	YES
121	a	1383.78	34.45187	YES	YES
122	a	1386.25	51.39094	YES	YES
123	a	1390.02	53.99131	YES	YES
124	a	1415.75	23.09390	YES	YES
125	a	1415.97	34.73189	YES	YES
126	a	1418.12	30.85748	YES	YES
127	a	1418.27	18.84150	YES	YES
128	a	1423.11	27.38930	YES	YES
129	a	1503.43	16.23444	YES	YES
130	a	1506.16	14.71599	YES	YES
131	a	1506.24	13.85523	YES	YES
132	a	1506.64	17.60027	YES	YES
133	a	1506.79	4.83344	YES	YES
134	a	1857.64	106.02459	YES	YES
135	a	1870.41	481.03887	YES	YES
136	a	1874.02	850.87933	YES	YES
137	a	1883.94	2305.28943	YES	YES
138	a	1915.25	43.15945	YES	YES
139	a	3087.67	8.92461	YES	YES
140	a	3088.81	9.05279	YES	YES
141	a	3090.14	10.73124	YES	YES
142	a	3092.61	7.93580	YES	YES
143	a	3094.52	6.52836	YES	YES
144	a	3135.09	10.94233	YES	YES
145	a	3140.26	10.18143	YES	YES
146	a	3143.51	10.02044	YES	YES
147	a	3145.50	8.83322	YES	YES
148	a	3150.67	8.66742	YES	YES
149	a	3156.38	1.86043	YES	YES
150	a	3156.76	2.03815	YES	YES
151	a	3157.27	1.91476	YES	YES
152	a	3162.97	1.10273	YES	YES
153	a	3163.34	1.05662	YES	YES

$end

Total COSMO energy + OC corr. = -2215.8052799258 H

[Li(DME)(FEC)$_3$]$^+$

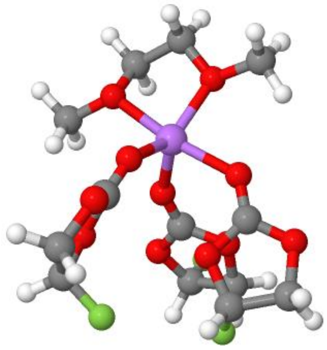

Method: (RI-)B3LYP(D3BJ)/def2-TZVPP
Symmetry: c1

Cartesian coordinates in Ångström:

O	2.0436756	1.6575114	-1.2204956
C	0.8054727	2.1839431	-1.4345477
O	-0.1194900	2.0352026	-0.6967954
O	0.7821291	2.8938757	-2.5646036
C	2.1053910	2.9430257	-3.1292937
C	2.8403986	1.8506104	-2.3629713
H	2.5284566	3.9305775	-2.9542743
H	2.0471402	2.7334516	-4.1929289
H	3.8604519	2.0688554	-2.0622386
F	2.8561610	0.6777471	-3.0877980
F	1.5632098	-4.0125005	2.9503200
H	0.3604706	-4.6412064	0.8997586
O	0.2302843	-2.6104132	0.5290202
C	0.8241493	-1.6272190	1.2140510
O	0.4496311	-0.4926497	1.2585920
C	1.0154026	-3.8152686	0.6396279
C	1.9917748	-3.4866565	1.7646216
O	1.9283034	-2.0763339	1.8510176
H	3.0242370	-3.7857899	1.6094356
H	1.5144006	-3.9915525	-0.3101834
Li	-0.9353914	0.8211433	0.6662015
H	-0.9934710	0.8219293	3.7470201
H	1.2944433	1.4689589	3.0767615
C	-1.3299909	1.7669390	3.3054307
C	0.8618572	2.3554001	2.6067524
H	-1.3053438	2.5410947	4.0788668
H	-3.4004111	1.2623374	3.5380294
O	-0.4847326	2.1161736	2.2181407
O	-2.6801757	0.7148065	1.6694894
H	0.9154048	3.2011592	3.2967062
C	-2.7276928	1.6323925	2.7583952
H	-4.6014490	-0.0326986	1.9023151
C	-3.9690494	0.4197041	1.1348267
H	-3.8238887	-0.2773698	0.3167378
H	-3.0986700	2.6004734	2.4057780
H	-4.4481772	1.3294264	0.7627541
H	1.4188695	2.5871302	1.7031379
H	-1.0498889	-2.0502902	-4.4438983
O	-1.6673271	-0.4353235	-0.8652783
H	1.1933181	-1.2564742	-3.8334907
C	-1.1074144	-1.0241993	-1.7374569
O	0.1662517	-0.7433451	-2.1453421
C	-0.6880381	-2.4106461	-3.4826993

```
O    -1.6209390   -2.0221242   -2.4575973
C     0.6030530   -1.7238022   -3.0521583
H    -0.5963033   -3.4924780   -3.4890400
F     1.4235065   -2.6055274   -2.3806965

SCF energy GEOOPT =  -1641.255863642 H
ZPE =   918.0 kJ/mol
FREEH energy =   998.60 kJ/mol
FREEH entropy =   0.90152 kJ/mol/K

$vibrational spectrum
#  mode     symmetry     wave number   IR intensity    selection rules
#                         cm**(-1)        km/mol          IR     RAMAN
     1                        0.00         0.00000         -       -
     2                        0.00         0.00000         -       -
     3                        0.00         0.00000         -       -
     4                        0.00         0.00000         -       -
     5                        0.00         0.00000         -       -
     6                        0.00         0.00000         -       -
     7         a              8.79         0.01991        YES     YES
     8         a             14.64         0.61702        YES     YES
     9         a             16.87         0.49439        YES     YES
    10         a             23.07         0.08413        YES     YES
    11         a             27.42         3.66376        YES     YES
    12         a             29.83         2.53630        YES     YES
    13         a             34.84         2.24827        YES     YES
    14         a             45.37         2.32720        YES     YES
    15         a             51.13         1.77718        YES     YES
    16         a             54.40         1.14432        YES     YES
    17         a             65.61         2.05920        YES     YES
    18         a             70.37         1.93774        YES     YES
    19         a             75.72         0.39563        YES     YES
    20         a             85.89         0.01533        YES     YES
    21         a             98.34         0.17728        YES     YES
    22         a            107.18         5.95773        YES     YES
    23         a            115.64         4.53208        YES     YES
    24         a            116.56         0.39467        YES     YES
    25         a            124.15         3.96924        YES     YES
    26         a            133.78         4.63464        YES     YES
    27         a            139.45         2.70748        YES     YES
    28         a            145.63         1.81290        YES     YES
    29         a            161.41         3.88091        YES     YES
    30         a            172.32         0.40466        YES     YES
    31         a            196.75         2.94200        YES     YES
    32         a            204.24         0.50073        YES     YES
    33         a            207.81         0.48422        YES     YES
    34         a            222.94         0.74049        YES     YES
    35         a            227.11         2.52415        YES     YES
    36         a            288.79         1.53149        YES     YES
    37         a            344.84        72.94840        YES     YES
    38         a            351.93        24.20148        YES     YES
    39         a            361.91        18.47186        YES     YES
    40         a            370.26        98.29407        YES     YES
    41         a            396.37        13.07432        YES     YES
    42         a            396.61        10.82287        YES     YES
    43         a            402.71        28.34052        YES     YES
    44         a            443.59       198.65296        YES     YES
    45         a            482.66         0.79665        YES     YES
    46         a            484.05         0.25506        YES     YES
    47         a            490.74         1.40911        YES     YES
    48         a            555.89         0.59559        YES     YES
    49         a            556.24         5.21755        YES     YES
    50         a            561.62         4.03622        YES     YES
```

51	a	580.55	3.88238	YES	YES
52	a	745.84	14.29506	YES	YES
53	a	748.81	39.60907	YES	YES
54	a	749.64	38.39767	YES	YES
55	a	769.47	20.94985	YES	YES
56	a	772.61	17.73522	YES	YES
57	a	774.89	14.83675	YES	YES
58	a	830.35	18.96256	YES	YES
59	a	832.38	12.11915	YES	YES
60	a	834.69	15.02955	YES	YES
61	a	852.02	9.39781	YES	YES
62	a	866.88	41.47297	YES	YES
63	a	868.21	26.87587	YES	YES
64	a	878.09	13.81346	YES	YES
65	a	884.39	59.60693	YES	YES
66	a	919.28	4.76934	YES	YES
67	a	922.25	8.40332	YES	YES
68	a	930.64	16.99288	YES	YES
69	a	998.16	7.20993	YES	YES
70	a	1011.73	204.11301	YES	YES
71	a	1018.52	232.50730	YES	YES
72	a	1033.02	15.75327	YES	YES
73	a	1047.55	5.92566	YES	YES
74	a	1047.77	22.40095	YES	YES
75	a	1058.61	5.96463	YES	YES
76	a	1060.99	5.22933	YES	YES
77	a	1085.70	15.55972	YES	YES
78	a	1091.40	218.54807	YES	YES
79	a	1099.85	276.02473	YES	YES
80	a	1107.34	267.90417	YES	YES
81	a	1121.89	122.59099	YES	YES
82	a	1123.77	69.26711	YES	YES
83	a	1128.65	3.93500	YES	YES
84	a	1138.55	69.08643	YES	YES
85	a	1144.61	71.63122	YES	YES
86	a	1174.58	264.09938	YES	YES
87	a	1179.54	16.38681	YES	YES
88	a	1182.00	117.91714	YES	YES
89	a	1182.28	263.78670	YES	YES
90	a	1188.67	4.32416	YES	YES
91	a	1217.81	18.29712	YES	YES
92	a	1234.79	1.05539	YES	YES
93	a	1240.30	36.08030	YES	YES
94	a	1240.82	22.73057	YES	YES
95	a	1245.72	40.37578	YES	YES
96	a	1268.15	16.46468	YES	YES
97	a	1302.62	9.10121	YES	YES
98	a	1365.00	16.75586	YES	YES
99	a	1366.28	7.33378	YES	YES
100	a	1367.20	43.72156	YES	YES
101	a	1381.86	34.39668	YES	YES
102	a	1384.17	11.90032	YES	YES
103	a	1388.34	48.17018	YES	YES
104	a	1402.41	17.87147	YES	YES
105	a	1415.68	29.98138	YES	YES
106	a	1418.23	36.37371	YES	YES
107	a	1420.95	21.32635	YES	YES
108	a	1438.93	5.24443	YES	YES
109	a	1481.87	0.42066	YES	YES
110	a	1482.68	1.06207	YES	YES
111	a	1492.19	11.54142	YES	YES
112	a	1498.41	11.10687	YES	YES
113	a	1499.39	4.86870	YES	YES

114	a	1501.59	10.32219	YES	YES
115	a	1506.19	14.81642	YES	YES
116	a	1506.64	18.17833	YES	YES
117	a	1506.80	2.43491	YES	YES
118	a	1515.16	6.55352	YES	YES
119	a	1517.49	4.08157	YES	YES
120	a	1861.56	289.68367	YES	YES
121	a	1870.75	607.14815	YES	YES
122	a	1905.20	1287.76295	YES	YES
123	a	2998.02	21.83446	YES	YES
124	a	3005.45	13.31892	YES	YES
125	a	3014.14	65.42634	YES	YES
126	a	3015.85	30.29902	YES	YES
127	a	3040.86	42.69596	YES	YES
128	a	3052.73	45.10207	YES	YES
129	a	3070.09	23.40782	YES	YES
130	a	3074.21	29.77596	YES	YES
131	a	3089.69	6.77754	YES	YES
132	a	3089.92	7.25954	YES	YES
133	a	3099.86	6.50329	YES	YES
134	a	3139.12	19.43825	YES	YES
135	a	3141.85	7.87021	YES	YES
136	a	3147.22	7.69217	YES	YES
137	a	3153.90	4.08179	YES	YES
138	a	3159.55	1.21079	YES	YES
139	a	3160.05	3.51919	YES	YES
140	a	3164.83	0.58440	YES	YES
141	a	3165.37	4.43049	YES	YES

$end

Total COSMO energy + OC corr. = -1641.3230615398 H

DEC

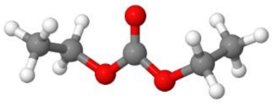

Method: (RI-)B3LYP(D3BJ)/def2-TZVPP
Symmetry: c2

Cartesian coordinates in Ångström:

H	3.4403771	0.4138379	-0.7355177
H	2.7145819	0.5825102	0.8751416
C	3.1063576	-0.1284655	0.1492261
H	3.9698562	-0.6277751	0.5921124
H	-1.6918294	1.6828099	0.6648007
O	0.0000000	0.0000000	1.1014995
C	-2.0553318	1.1563136	-0.2158678
C	0.0000000	-0.0000000	-0.1051084
O	-0.9316979	0.5460570	-0.8913228
C	2.0553318	-1.1563136	-0.2158678
H	-2.4353958	1.8773824	-0.9364943
O	0.9316979	-0.5460570	-0.8913228
H	2.4353958	-1.8773824	-0.9364943
H	1.6918294	-1.6828099	0.6648007
C	-3.1063576	0.1284655	0.1492261
H	-3.9698562	0.6277751	0.5921124

```
H    -2.7145819   -0.5825102    0.8751416
H    -3.4403771   -0.4138379   -0.7355177

SCF energy GEOOPT =  -422.2129862775 H
ZPE =   399.0 kJ/mol
FREEH energy =   423.37 kJ/mol
FREEH entropy =   0.39104 kJ/mol/K

$vibrational spectrum
#  mode     symmetry     wave number   IR intensity    selection rules
#                         cm**(-1)        km/mol          IR     RAMAN
     1                      0.00         0.00000         -        -
     2                      0.00         0.00000         -        -
     3                      0.00         0.00000         -        -
     4                      0.00         0.00000         -        -
     5                      0.00         0.00000         -        -
     6                      0.00         0.00000         -        -
     7        a            59.70         0.28626        YES      YES
     8        b            68.95         3.67149        YES      YES
     9        b           102.29         0.90988        YES      YES
    10        a           124.13         0.01384        YES      YES
    11        a           194.57         2.80469        YES      YES
    12        b           227.48         1.61275        YES      YES
    13        a           288.04         3.28072        YES      YES
    14        b           359.64        22.83776        YES      YES
    15        a           376.58         1.28786        YES      YES
    16        b           429.94         7.15549        YES      YES
    17        a           543.50         0.25917        YES      YES
    18        b           686.69         0.57788        YES      YES
    19        a           801.91         5.94673        YES      YES
    20        b           802.64        21.78232        YES      YES
    21        b           822.63         0.59246        YES      YES
    22        b           866.29        25.78005        YES      YES
    23        a           907.58         6.36732        YES      YES
    24        a           973.87         9.56408        YES      YES
    25        b          1029.95       126.23832        YES      YES
    26        a          1102.87         0.62489        YES      YES
    27        b          1117.21        53.56624        YES      YES
    28        a          1141.38         1.21780        YES      YES
    29        b          1195.71         9.55392        YES      YES
    30        a          1209.55         4.76817        YES      YES
    31        b          1284.94       863.30682        YES      YES
    32        b          1333.78        63.35694        YES      YES
    33        a          1337.24         2.96011        YES      YES
    34        a          1399.45         4.47465        YES      YES
    35        b          1408.36        44.45182        YES      YES
    36        a          1419.35         0.19104        YES      YES
    37        b          1430.63        31.55648        YES      YES
    38        a          1486.88        17.05309        YES      YES
    39        b          1487.04         0.70063        YES      YES
    40        a          1493.61         0.59457        YES      YES
    41        b          1496.26        14.13393        YES      YES
    42        a          1507.55         7.37020        YES      YES
    43        b          1510.22        27.83737        YES      YES
    44        a          1778.14       273.92930        YES      YES
    45        b          3038.88        31.04284        YES      YES
    46        a          3039.00         0.19603        YES      YES
    47        b          3071.65        43.88710        YES      YES
    48        a          3071.94         0.78286        YES      YES
    49        b          3101.18        37.42993        YES      YES
    50        a          3101.24         8.66119        YES      YES
    51        b          3109.72        14.02061        YES      YES
    52        a          3109.74         1.08796        YES      YES
```

53	b	3131.72	13.84067	YES	YES
54	a	3132.04	35.89477	YES	YES

$end

Total COSMO energy + OC corr. = -422.2215175474 H

$[Ga(C_2F_5)_4]^-$

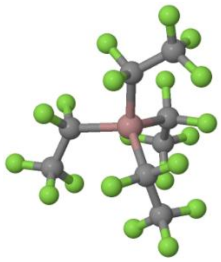

Method: (RI-)B3LYP(D3BJ)/def2-TZVPP
Symmetry: c1

Cartesian coordinates in Ångström:

F	1.7517772	3.0957360	-2.4487997
F	3.7635142	3.0506157	-1.6379426
F	-2.5514863	3.9083254	-0.9787172
C	2.4588052	3.1490791	-1.3068779
F	2.4243607	0.8570814	-0.9269985
F	2.2753114	4.3607005	-0.7619781
C	2.0165301	2.0396863	-0.3396883
C	-1.4880747	4.5492672	-0.4692690
F	-1.9142604	5.7548209	-0.0384260
F	-1.8285911	3.5817308	1.6061097
F	0.2834136	0.6806960	-3.0678756
C	-0.8417483	3.7221370	0.6517776
Ga	0.0176904	1.8801940	0.2107216
F	-0.6139890	4.7670773	-1.4661578
F	2.8087543	2.2013666	0.7790647
F	-0.7408482	-0.6218311	-0.9509117
C	-0.9554818	0.9520469	-2.6323933
C	-1.0668070	0.7092184	-1.1185388
F	0.1132098	4.5455761	1.2153896
F	-1.8078930	0.1784695	-3.3370134
F	-1.2329498	2.2309238	-2.9357373
F	-2.4144587	0.8122578	-0.8254112
F	-2.1079264	1.0076356	2.7695265
F	0.5479709	1.7540947	3.0085196
C	-1.0853800	0.1543501	2.6065267
F	-0.8270996	-0.3979152	3.8122215
C	0.1455936	0.8701944	2.0248066
F	-1.4953767	-0.8330480	1.7960404
F	1.1266795	-0.1004169	1.9597019

SCF energy GEOOPT = -4226.798511957 H
ZPE = 267.2 kJ/mol
FREEH energy = 346.18 kJ/mol
FREEH entropy = 0.84009 kJ/mol/K

$vibrational spectrum

# mode	symmetry	wave number	IR intensity	selection rules

#		cm**(-1)	km/mol	IR	RAMAN
1		-0.00	0.00000	-	-
2		-0.00	0.00000	-	-
3		-0.00	0.00000	-	-
4		-0.00	0.00000	-	-
5		0.00	0.00000	-	-
6		0.00	0.00000	-	-
7	a	21.69	0.05238	YES	YES
8	a	32.39	0.16942	YES	YES
9	a	35.73	0.04071	YES	YES
10	a	43.45	0.08854	YES	YES
11	a	47.21	0.00540	YES	YES
12	a	53.34	0.04825	YES	YES
13	a	58.83	0.12211	YES	YES
14	a	60.23	0.10999	YES	YES
15	a	64.51	0.20652	YES	YES
16	a	68.42	0.07032	YES	YES
17	a	77.99	0.57510	YES	YES
18	a	78.94	0.34318	YES	YES
19	a	85.37	0.39949	YES	YES
20	a	99.20	0.64846	YES	YES
21	a	102.03	0.73595	YES	YES
22	a	110.96	0.12992	YES	YES
23	a	137.57	2.53844	YES	YES
24	a	178.73	0.01741	YES	YES
25	a	189.91	0.25397	YES	YES
26	a	192.64	1.09567	YES	YES
27	a	209.62	1.44509	YES	YES
28	a	210.67	0.65599	YES	YES
29	a	223.27	0.30774	YES	YES
30	a	225.31	0.11459	YES	YES
31	a	227.61	0.33439	YES	YES
32	a	230.73	0.11035	YES	YES
33	a	253.86	22.56897	YES	YES
34	a	272.65	5.69973	YES	YES
35	a	280.26	1.73092	YES	YES
36	a	281.47	0.25878	YES	YES
37	a	298.47	18.55151	YES	YES
38	a	304.13	37.07229	YES	YES
39	a	306.47	33.89836	YES	YES
40	a	361.20	0.51523	YES	YES
41	a	363.73	0.35138	YES	YES
42	a	364.20	1.64359	YES	YES
43	a	364.62	1.67195	YES	YES
44	a	424.56	0.11573	YES	YES
45	a	426.68	0.10922	YES	YES
46	a	430.78	0.55665	YES	YES
47	a	432.43	0.74279	YES	YES
48	a	525.17	1.46054	YES	YES
49	a	526.75	0.98063	YES	YES
50	a	526.88	0.76849	YES	YES
51	a	527.05	0.69899	YES	YES
52	a	580.49	1.18515	YES	YES
53	a	581.08	0.62637	YES	YES
54	a	581.94	3.89494	YES	YES
55	a	582.45	4.54899	YES	YES
56	a	588.90	1.68113	YES	YES
57	a	594.20	2.38703	YES	YES
58	a	597.95	0.92417	YES	YES
59	a	599.00	1.96981	YES	YES
60	a	730.07	23.13237	YES	YES
61	a	730.33	9.78063	YES	YES
62	a	730.84	20.82568	YES	YES

63	a	731.56	3.44842	YES	YES
64	a	917.82	88.25692	YES	YES
65	a	925.04	68.85575	YES	YES
66	a	927.96	78.89906	YES	YES
67	a	932.75	28.01944	YES	YES
68	a	1011.32	36.73199	YES	YES
69	a	1016.89	3.62579	YES	YES
70	a	1026.27	111.68569	YES	YES
71	a	1030.97	156.99073	YES	YES
72	a	1086.88	6.03766	YES	YES
73	a	1094.17	124.45320	YES	YES
74	a	1096.33	113.44609	YES	YES
75	a	1105.28	223.61662	YES	YES
76	a	1150.80	347.87524	YES	YES
77	a	1151.29	390.69894	YES	YES
78	a	1159.45	298.06327	YES	YES
79	a	1162.26	8.40726	YES	YES
80	a	1184.68	98.14807	YES	YES
81	a	1186.16	262.35225	YES	YES
82	a	1187.57	137.48540	YES	YES
83	a	1191.19	420.09275	YES	YES
84	a	1281.02	375.23033	YES	YES
85	a	1288.46	144.28070	YES	YES
86	a	1290.09	139.79383	YES	YES
87	a	1306.63	66.74596	YES	YES

$end

Total COSMO energy + OC corr. = -4226.8510063646 H

$[Li(DME)(DEC)_2]^+$

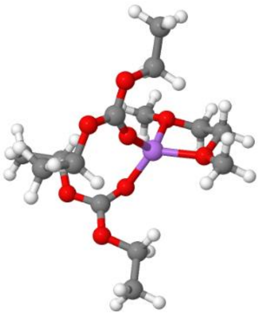

Method: (RI-)B3LYP(D3BJ)/def2-TZVPP
Symmetry: c1

Cartesian coordinates in Ångström:

C	-0.5152140	2.7460788	0.1385343
O	0.1739969	1.7111033	0.8375840
Li	-0.1147482	0.0052146	-0.0870707
O	-0.9733829	1.0928677	-1.4967358
C	-0.4601174	2.4160097	-1.3319892
C	0.2405960	1.9184374	2.2455625
H	-0.0392888	3.7133536	0.3221024
H	-1.5524970	2.7954684	0.4866442
C	-0.9980453	0.6657622	-2.8586264

```
H    -1.0626230    3.1309784   -1.8989396
H     0.5707479    2.4566910   -1.6984363
H    -1.4270852   -0.3325838   -2.8731451
H     0.0127591    0.6351190   -3.2716595
H    -1.6193810    1.3355415   -3.4561420
H     0.7575305    1.0634710    2.6732544
H    -0.7620207    1.9883153    2.6759897
H     0.7953073    2.8311168    2.4719559
O     1.3250049   -0.9780162   -0.8107649
H    -0.4486011   -5.1433091   -1.5460152
H     5.3551189   -2.2192379    0.9423057
H     3.7631853   -0.4995603    0.0327596
H    -0.6392023   -2.6422896   -1.4462652
H     5.1562996   -0.8848272    2.0827433
H    -1.0223180   -4.5012046   -3.0886365
C    -0.1740670   -4.5310291   -2.4044443
C     4.6454991   -1.7173621    1.5984660
C     1.7843529   -2.0893390   -0.5839443
C     3.4666931   -1.1937568    0.8174644
C     0.1781924   -3.1271042   -1.9802417
O     1.3154472   -3.2184271   -1.0788764
O     2.8174544   -2.3376445    0.1911071
H     0.6623879   -5.0053175   -2.9154143
H     4.3264725   -2.4191467    2.3681517
H     2.7351016   -0.6993268    1.4571196
H     0.4639076   -2.5005902   -2.8244145
O    -1.0197564   -1.2505589    0.9584658
H    -1.4029351   -5.5978809    2.3734357
H    -5.1681442    0.2252359    2.3065836
H    -2.8173908    0.6659541    1.5440742
H    -0.2475263   -3.8034160    1.0486078
H    -5.1602641    1.1863211    0.8229549
H     0.2968476   -5.4626874    2.8375816
C    -0.6642433   -4.9481377    2.8408412
C    -4.9159858    0.2120831    1.2472396
C    -1.9400553   -1.8288573    1.5239514
C    -3.4484876   -0.0695005    1.0437821
C    -0.5333972   -3.6488509    2.0876584
O    -1.8389053   -2.9983736    2.1112006
O    -3.1699530   -1.3713308    1.6279705
H    -0.9580802   -4.7738848    3.8749436
H    -5.5287899   -0.5404037    0.7529890
H    -3.1748879   -0.0962128   -0.0105425
H     0.1883017   -2.9750347    2.5481597

SCF energy GEOOPT =  -1160.725973061 H
ZPE =   1183. kJ/mol
FREEH energy =  1266.10 kJ/mol
FREEH entropy =   0.91987 kJ/mol/K

$vibrational spectrum
#  mode     symmetry     wave number   IR intensity    selection rules
#                         cm**(-1)        km/mol          IR     RAMAN
     1                      -0.00         0.00000         -        -
     2                      -0.00         0.00000         -        -
     3                      -0.00         0.00000         -        -
     4                      -0.00         0.00000         -        -
     5                      -0.00         0.00000         -        -
     6                      -0.00         0.00000         -        -
     7         a             6.29         0.10187        YES      YES
     8         a            10.61         0.04465        YES      YES
     9         a            18.06         0.05163        YES      YES
    10         a            21.00         0.08682        YES      YES
```

11	a	26.57	0.03641	YES	YES
12	a	31.48	0.09930	YES	YES
13	a	35.44	0.18729	YES	YES
14	a	40.06	0.05407	YES	YES
15	a	53.23	0.43624	YES	YES
16	a	57.02	1.24488	YES	YES
17	a	62.78	0.22075	YES	YES
18	a	65.67	0.10500	YES	YES
19	a	69.26	0.64505	YES	YES
20	a	78.94	0.68525	YES	YES
21	a	82.51	1.06480	YES	YES
22	a	101.62	1.16171	YES	YES
23	a	112.67	5.12657	YES	YES
24	a	121.43	7.15723	YES	YES
25	a	125.01	3.75314	YES	YES
26	a	137.47	0.07939	YES	YES
27	a	143.95	0.80279	YES	YES
28	a	161.52	0.00021	YES	YES
29	a	162.67	0.23831	YES	YES
30	a	163.87	0.11148	YES	YES
31	a	190.06	0.86534	YES	YES
32	a	217.27	2.75667	YES	YES
33	a	226.62	1.86277	YES	YES
34	a	255.24	1.96992	YES	YES
35	a	256.35	2.65408	YES	YES
36	a	258.83	1.32602	YES	YES
37	a	259.27	4.36163	YES	YES
38	a	265.17	0.00603	YES	YES
39	a	266.52	0.09583	YES	YES
40	a	283.18	2.01971	YES	YES
41	a	330.78	10.92697	YES	YES
42	a	347.44	0.23180	YES	YES
43	a	348.58	0.04970	YES	YES
44	a	362.72	2.61228	YES	YES
45	a	379.58	21.69925	YES	YES
46	a	389.81	42.01197	YES	YES
47	a	390.36	20.20956	YES	YES
48	a	452.86	71.90531	YES	YES
49	a	495.01	40.04027	YES	YES
50	a	536.93	40.84520	YES	YES
51	a	559.79	127.97464	YES	YES
52	a	578.30	3.48161	YES	YES
53	a	741.74	1.30684	YES	YES
54	a	742.53	0.68001	YES	YES
55	a	805.41	18.05304	YES	YES
56	a	808.66	34.24934	YES	YES
57	a	821.07	2.10264	YES	YES
58	a	822.03	0.19544	YES	YES
59	a	828.74	1.95309	YES	YES
60	a	830.97	6.58028	YES	YES
61	a	845.14	7.91360	YES	YES
62	a	858.58	53.87253	YES	YES
63	a	860.39	55.31101	YES	YES
64	a	878.45	46.80041	YES	YES
65	a	918.99	1.02790	YES	YES
66	a	922.81	0.24306	YES	YES
67	a	1019.60	5.25519	YES	YES
68	a	1021.76	18.57999	YES	YES
69	a	1026.61	111.69583	YES	YES
70	a	1027.88	114.40881	YES	YES
71	a	1028.37	31.53113	YES	YES
72	a	1043.46	13.41869	YES	YES
73	a	1101.42	259.38119	YES	YES

74	a	1123.35	15.43389	YES	YES
75	a	1133.62	0.94281	YES	YES
76	a	1134.09	0.82094	YES	YES
77	a	1137.19	6.01992	YES	YES
78	a	1137.90	5.48006	YES	YES
79	a	1139.98	40.01477	YES	YES
80	a	1161.38	4.70686	YES	YES
81	a	1166.86	5.37577	YES	YES
82	a	1177.27	0.17575	YES	YES
83	a	1179.58	1.06967	YES	YES
84	a	1180.22	4.80737	YES	YES
85	a	1180.75	5.22567	YES	YES
86	a	1184.35	6.00243	YES	YES
87	a	1186.67	4.53699	YES	YES
88	a	1214.79	16.11284	YES	YES
89	a	1231.48	0.14295	YES	YES
90	a	1267.18	10.10128	YES	YES
91	a	1301.08	2.67381	YES	YES
92	a	1302.14	11.10007	YES	YES
93	a	1303.04	2.13272	YES	YES
94	a	1303.35	1.88083	YES	YES
95	a	1305.24	10.09706	YES	YES
96	a	1331.63	801.90679	YES	YES
97	a	1340.52	666.13231	YES	YES
98	a	1399.06	13.11727	YES	YES
99	a	1400.64	14.88936	YES	YES
100	a	1402.54	15.16428	YES	YES
101	a	1423.43	81.86213	YES	YES
102	a	1424.00	82.47312	YES	YES
103	a	1430.26	13.99805	YES	YES
104	a	1430.84	17.47058	YES	YES
105	a	1438.62	2.88973	YES	YES
106	a	1455.50	142.90404	YES	YES
107	a	1461.86	165.49450	YES	YES
108	a	1483.90	0.91662	YES	YES
109	a	1484.56	0.35882	YES	YES
110	a	1485.93	6.07071	YES	YES
111	a	1486.07	3.04480	YES	YES
112	a	1486.53	8.72448	YES	YES
113	a	1486.78	12.78432	YES	YES
114	a	1488.95	15.87644	YES	YES
115	a	1491.25	18.22118	YES	YES
116	a	1498.45	4.77177	YES	YES
117	a	1498.79	4.48465	YES	YES
118	a	1499.17	6.49395	YES	YES
119	a	1500.74	8.32297	YES	YES
120	a	1501.03	3.64543	YES	YES
121	a	1502.85	4.32968	YES	YES
122	a	1515.57	4.49156	YES	YES
123	a	1517.88	7.57444	YES	YES
124	a	1518.49	17.66096	YES	YES
125	a	1519.52	37.77287	YES	YES
126	a	1523.58	22.22904	YES	YES
127	a	1524.81	3.48929	YES	YES
128	a	1704.35	424.12540	YES	YES
129	a	1732.58	526.40231	YES	YES
130	a	3006.67	17.20092	YES	YES
131	a	3012.60	14.24126	YES	YES
132	a	3016.16	40.00535	YES	YES
133	a	3022.14	30.82023	YES	YES
134	a	3048.44	6.13961	YES	YES
135	a	3048.93	6.36327	YES	YES
136	a	3049.42	7.08000	YES	YES

137	a	3049.55	5.64304	YES	YES
138	a	3050.91	30.04208	YES	YES
139	a	3054.41	25.58751	YES	YES
140	a	3054.82	5.72151	YES	YES
141	a	3055.71	35.93449	YES	YES
142	a	3063.21	30.18635	YES	YES
143	a	3064.29	17.42851	YES	YES
144	a	3078.21	22.35345	YES	YES
145	a	3085.75	19.43999	YES	YES
146	a	3094.33	6.94525	YES	YES
147	a	3096.72	4.37566	YES	YES
148	a	3099.61	4.10426	YES	YES
149	a	3105.86	1.30159	YES	YES
150	a	3116.41	12.85076	YES	YES
151	a	3116.83	12.02655	YES	YES
152	a	3117.32	13.38398	YES	YES
153	a	3118.25	11.00054	YES	YES
154	a	3127.99	15.38901	YES	YES
155	a	3128.64	29.79189	YES	YES
156	a	3129.32	15.02434	YES	YES
157	a	3131.60	22.52540	YES	YES
158	a	3137.50	12.82419	YES	YES
159	a	3138.45	17.24238	YES	YES

$end

Total COSMO energy + OC corr. = -1160.7826481910 H

Li[Ga(C_2F_5)$_4$]

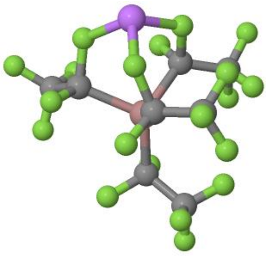

Method: (RI-)B3LYP(D3BJ)/def2-TZVPP
Symmetry: c1

Cartesian coordinates in Ångström:

F	1.8228942	2.9596950	-2.5063276
F	3.7529261	3.0475105	-1.5182602
F	-2.5495536	4.0598033	-0.9562698
C	2.4316021	3.1344529	-1.3310241
F	2.5373099	0.9211509	-0.6014498
F	2.1581772	4.3730101	-0.8865512
C	1.9240218	2.0886729	-0.3200771
C	-1.4484588	4.5882125	-0.4129680
F	-1.7622802	5.7776485	0.1150159
F	-1.8518266	3.4967915	1.5993097
F	0.2723459	0.5928238	-3.0756518
C	-0.9029781	3.6126217	0.6414415
Ga	-0.1072719	1.7735045	0.0690236
F	-0.5441789	4.7830743	-1.3809125
F	2.5341085	2.5306695	0.9279564
F	-0.9302553	-0.6933426	-1.0274546
C	-0.9876053	0.9003800	-2.7300549
C	-1.1941072	0.6360635	-1.2299429
F	0.1683145	4.3424311	1.2958661
F	-1.8188877	0.1860243	-3.4922164
F	-1.1878316	2.2038712	-2.9932335
F	-2.5292846	0.8228464	-0.9716584
F	-2.0853536	0.9496362	2.7268041
F	0.5147964	1.8854451	2.8758536
C	-1.0334029	0.1294576	2.6196716
F	-0.7049832	-0.2950332	3.8473317
C	0.1378682	0.8471508	1.9293441
F	-1.3884896	-0.9293839	1.8860175
F	1.1895531	-0.0064656	1.9393120
Li	1.4615014	3.3487068	2.2119749

SCF energy GEOOPT = -4234.275591700 H
ZPE = 274.7 kJ/mol
FREEH energy = 355.94 kJ/mol
FREEH entropy = 0.84860 kJ/mol/K

$vibrational spectrum

# mode	symmetry	wave number	IR intensity	selection rules	
#		cm**(-1)	km/mol	IR	RAMAN
1		-0.00	0.00000	-	-
2		-0.00	0.00000	-	-
3		-0.00	0.00000	-	-
4		0.00	0.00000	-	-
5		0.00	0.00000	-	-
6		0.00	0.00000	-	-
7	a	25.49	0.07395	YES	YES

8	a	27.39	0.01761	YES	YES
9	a	34.61	0.09696	YES	YES
10	a	45.80	0.02407	YES	YES
11	a	48.64	0.04967	YES	YES
12	a	51.63	0.11798	YES	YES
13	a	58.23	0.32317	YES	YES
14	a	59.47	0.55962	YES	YES
15	a	67.11	0.11196	YES	YES
16	a	71.29	0.33126	YES	YES
17	a	81.74	0.37867	YES	YES
18	a	85.86	1.76918	YES	YES
19	a	93.41	0.82071	YES	YES
20	a	104.24	0.59351	YES	YES
21	a	111.56	1.21753	YES	YES
22	a	123.48	1.77227	YES	YES
23	a	144.74	2.32174	YES	YES
24	a	180.13	0.07027	YES	YES
25	a	189.85	2.64581	YES	YES
26	a	193.15	0.24646	YES	YES
27	a	206.42	0.29667	YES	YES
28	a	209.17	3.31810	YES	YES
29	a	215.09	1.81541	YES	YES
30	a	222.48	0.29975	YES	YES
31	a	228.15	4.31065	YES	YES
32	a	240.19	10.05577	YES	YES
33	a	247.56	12.20936	YES	YES
34	a	274.01	1.09272	YES	YES
35	a	276.78	7.36275	YES	YES
36	a	281.61	8.82780	YES	YES
37	a	293.75	12.05617	YES	YES
38	a	297.79	21.51002	YES	YES
39	a	307.55	95.55165	YES	YES
40	a	354.73	2.67922	YES	YES
41	a	355.96	2.48412	YES	YES
42	a	362.27	7.81609	YES	YES
43	a	364.57	0.52324	YES	YES
44	a	383.64	17.02984	YES	YES
45	a	405.73	27.74712	YES	YES
46	a	407.07	26.71715	YES	YES
47	a	428.53	1.22098	YES	YES
48	a	436.81	23.76165	YES	YES
49	a	449.75	51.84181	YES	YES
50	a	461.59	55.38526	YES	YES
51	a	520.82	4.33437	YES	YES
52	a	523.26	1.92182	YES	YES
53	a	525.61	16.42810	YES	YES
54	a	529.78	2.07427	YES	YES
55	a	573.54	6.73129	YES	YES
56	a	574.57	6.95490	YES	YES
57	a	579.73	31.25193	YES	YES
58	a	582.52	1.75698	YES	YES
59	a	583.09	2.96801	YES	YES
60	a	588.86	1.11074	YES	YES
61	a	590.97	14.71597	YES	YES
62	a	600.61	1.76442	YES	YES
63	a	723.95	44.96476	YES	YES
64	a	725.58	14.69454	YES	YES
65	a	730.77	16.31539	YES	YES
66	a	735.32	15.49892	YES	YES
67	a	854.26	18.86253	YES	YES
68	a	863.66	33.18081	YES	YES
69	a	891.82	231.68424	YES	YES
70	a	934.16	83.13967	YES	YES

71	a	945.49	44.58608	YES	YES
72	a	947.61	23.68647	YES	YES
73	a	950.08	69.18797	YES	YES
74	a	1057.39	70.98152	YES	YES
75	a	1110.17	120.73992	YES	YES
76	a	1132.39	52.13110	YES	YES
77	a	1140.54	131.34132	YES	YES
78	a	1153.90	136.63497	YES	YES
79	a	1172.45	141.66273	YES	YES
80	a	1177.27	419.57953	YES	YES
81	a	1187.94	326.80368	YES	YES
82	a	1194.17	180.56793	YES	YES
83	a	1199.35	115.46868	YES	YES
84	a	1204.06	383.44062	YES	YES
85	a	1205.93	188.09263	YES	YES
86	a	1215.87	98.71014	YES	YES
87	a	1283.48	389.24009	YES	YES
88	a	1294.67	198.05920	YES	YES
89	a	1298.77	159.56525	YES	YES
90	a	1312.17	55.24077	YES	YES

$end

Total COSMO energy + OC corr. = -4234.2992526272 H

(DEC)Li[Ga(C$_2$F$_5$)$_4$]

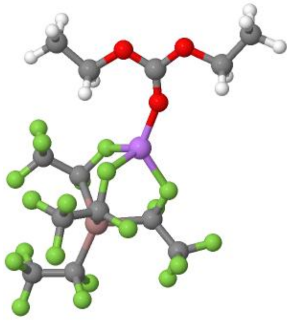

Method: (RI-)B3LYP(D3BJ)/def2-TZVPP
Symmetry: c1

Cartesian coordinates in Ångström:

F	1.8975171	3.5292249	-2.2343227
F	3.6691373	3.6422613	-0.9854386
F	-2.7667596	4.0469482	-0.7594871
C	2.3293568	3.5610874	-0.9732192
F	2.5704905	1.2715569	-0.6537939
F	1.8652244	4.6835150	-0.3924462
C	1.8372668	2.3202160	-0.2076978
C	-1.7430680	4.5339173	-0.0513184
F	-2.1890332	5.5625964	0.6849405
F	-2.1391171	3.0270050	1.6696307
F	0.4713512	1.3221272	-3.3576362
C	-1.1534171	3.4134778	0.8179226
Ga	-0.1775213	1.7952782	-0.0512579
F	-0.8203859	5.0033995	-0.9029902
F	2.3063363	2.5580715	1.1316756
F	-0.7399225	-0.4306654	-1.7087344
C	-0.8216133	1.4831124	-3.0355839
C	-1.0908071	0.8934921	-1.6421564
F	-0.1911797	4.0648765	1.6598422

```
F    -1.5640394    0.9081009   -3.9864146
F    -1.0905193    2.8014449   -3.0329358
F    -2.4511256    0.9352066   -1.4516002
F    -2.2581219    0.2649392    2.1683154
F     0.2310392    1.3397350    2.7454797
C    -1.1236338   -0.4249267    1.9951405
F    -0.8531991   -1.0759609    3.1367837
C     0.0211663    0.5155900    1.5863119
F    -1.3185722   -1.3306569    1.0313603
F     1.1439471   -0.2466054    1.5039371
Li    1.1437238    3.0205146    2.6263100
H     2.0530486    5.4680091    8.2899583
H     3.4222288    7.8584962    2.1414072
H     1.0387497    4.0667142    6.4669037
C     2.4409067    4.4735777    8.0720943
C     3.8389568    6.9151250    1.7907661
H     2.0155842    5.7861017    2.0760303
H     1.9870404    3.7672404    8.7676907
O     1.9224074    3.9309118    3.9904607
C     2.1108401    4.0620784    6.6591457
C     2.5625982    4.8543288    4.4790786
C     3.0696738    5.7439098    2.3457351
H     3.7789694    6.8934781    0.7027881
O     2.7355505    5.0327106    5.7698737
O     3.1708931    5.8002475    3.8000684
H     3.5177081    4.4750916    8.2372405
H     4.8880720    6.8657294    2.0797934
H     2.5015559    3.0755595    6.4133219
H     3.4814955    4.7927908    2.0128964

SCF energy GEOOPT =  -4656.533464630 H
ZPE =    676.5 kJ/mol
FREEH energy =    788.16 kJ/mol
FREEH entropy =    1.11302 kJ/mol/K

$vibrational spectrum
#  mode     symmetry     wave number   IR intensity    selection rules
#                         cm**(-1)        km/mol          IR     RAMAN
     1                      -0.00         0.00000          -       -
     2                      -0.00         0.00000          -       -
     3                       0.00         0.00000          -       -
     4                       0.00         0.00000          -       -
     5                       0.00         0.00000          -       -
     6                       0.00         0.00000          -       -
     7         a             9.08         0.01148         YES     YES
     8         a            12.62         0.01323         YES     YES
     9         a            20.25         0.04466         YES     YES
    10         a            24.75         0.03661         YES     YES
    11         a            30.69         0.07270         YES     YES
    12         a            38.46         0.01210         YES     YES
    13         a            41.16         0.11914         YES     YES
    14         a            44.72         0.04715         YES     YES
    15         a            49.03         0.06513         YES     YES
    16         a            53.47         0.03558         YES     YES
    17         a            53.70         0.15591         YES     YES
    18         a            59.37         0.75669         YES     YES
    19         a            61.33         0.32904         YES     YES
    20         a            66.59         1.88479         YES     YES
    21         a            69.84         0.76516         YES     YES
    22         a            72.29         0.90446         YES     YES
    23         a            74.61         0.58731         YES     YES
    24         a            83.82         1.26627         YES     YES
    25         a            85.89         1.36257         YES     YES
```

26	a	90.98	0.04438	YES	YES
27	a	94.65	0.51009	YES	YES
28	a	107.45	0.49921	YES	YES
29	a	112.31	2.43461	YES	YES
30	a	122.19	1.52828	YES	YES
31	a	127.65	4.12776	YES	YES
32	a	139.89	0.25026	YES	YES
33	a	147.19	3.22837	YES	YES
34	a	167.45	0.00784	YES	YES
35	a	181.59	0.03912	YES	YES
36	a	192.72	0.69406	YES	YES
37	a	194.46	0.07288	YES	YES
38	a	207.63	0.18282	YES	YES
39	a	210.60	2.84853	YES	YES
40	a	220.37	1.45485	YES	YES
41	a	223.55	1.47227	YES	YES
42	a	225.36	5.38279	YES	YES
43	a	240.29	6.96847	YES	YES
44	a	250.75	1.96130	YES	YES
45	a	257.93	0.81230	YES	YES
46	a	260.72	1.40185	YES	YES
47	a	267.12	0.02333	YES	YES
48	a	272.52	2.18430	YES	YES
49	a	275.74	3.67990	YES	YES
50	a	282.98	6.21354	YES	YES
51	a	293.69	7.22493	YES	YES
52	a	297.61	9.72716	YES	YES
53	a	328.82	53.26602	YES	YES
54	a	336.83	58.22088	YES	YES
55	a	344.67	27.51743	YES	YES
56	a	349.15	2.22281	YES	YES
57	a	362.49	0.44710	YES	YES
58	a	364.63	0.44126	YES	YES
59	a	369.05	9.81498	YES	YES
60	a	377.91	20.43409	YES	YES
61	a	391.39	34.68127	YES	YES
62	a	425.25	1.69910	YES	YES
63	a	429.01	5.02918	YES	YES
64	a	432.18	1.33468	YES	YES
65	a	436.52	9.20195	YES	YES
66	a	490.87	41.72325	YES	YES
67	a	522.74	1.56177	YES	YES
68	a	523.34	2.20949	YES	YES
69	a	525.09	2.07009	YES	YES
70	a	529.34	1.35598	YES	YES
71	a	576.77	9.50635	YES	YES
72	a	577.42	3.37416	YES	YES
73	a	580.69	0.85513	YES	YES
74	a	582.60	3.46310	YES	YES
75	a	583.89	1.18233	YES	YES
76	a	587.61	54.94974	YES	YES
77	a	591.12	0.62404	YES	YES
78	a	600.36	20.47712	YES	YES
79	a	601.28	198.30342	YES	YES
80	a	727.25	36.47413	YES	YES
81	a	728.38	16.61898	YES	YES
82	a	732.01	16.23755	YES	YES
83	a	734.57	14.07014	YES	YES
84	a	743.90	2.69995	YES	YES
85	a	805.45	22.90181	YES	YES
86	a	823.44	1.16787	YES	YES
87	a	836.28	0.36078	YES	YES
88	a	856.88	63.56419	YES	YES

89	a	888.92	24.33739	YES	YES
90	a	897.99	48.66180	YES	YES
91	a	923.24	192.37495	YES	YES
92	a	925.84	19.25781	YES	YES
93	a	934.53	166.30738	YES	YES
94	a	945.23	11.42811	YES	YES
95	a	946.16	47.06436	YES	YES
96	a	948.60	74.43010	YES	YES
97	a	1024.73	3.07670	YES	YES
98	a	1026.93	160.50429	YES	YES
99	a	1052.46	70.47026	YES	YES
100	a	1108.84	119.38231	YES	YES
101	a	1120.70	52.64452	YES	YES
102	a	1128.95	119.41722	YES	YES
103	a	1134.55	1.85995	YES	YES
104	a	1136.23	15.92478	YES	YES
105	a	1141.35	155.88469	YES	YES
106	a	1162.73	140.31678	YES	YES
107	a	1168.92	17.41808	YES	YES
108	a	1169.74	351.61778	YES	YES
109	a	1179.39	24.72767	YES	YES
110	a	1181.80	73.51801	YES	YES
111	a	1185.02	191.00932	YES	YES
112	a	1188.54	232.69504	YES	YES
113	a	1194.62	68.00507	YES	YES
114	a	1201.17	427.74636	YES	YES
115	a	1203.25	227.66802	YES	YES
116	a	1211.30	136.97973	YES	YES
117	a	1284.01	337.70400	YES	YES
118	a	1294.85	174.28685	YES	YES
119	a	1298.09	160.89091	YES	YES
120	a	1305.19	1.02819	YES	YES
121	a	1311.97	69.76253	YES	YES
122	a	1312.65	2.82857	YES	YES
123	a	1344.79	790.42548	YES	YES
124	a	1403.06	11.89823	YES	YES
125	a	1421.44	82.73061	YES	YES
126	a	1429.58	20.87985	YES	YES
127	a	1466.11	219.88150	YES	YES
128	a	1486.77	6.75980	YES	YES
129	a	1488.11	8.02408	YES	YES
130	a	1499.82	3.54101	YES	YES
131	a	1501.27	5.91484	YES	YES
132	a	1520.39	25.48096	YES	YES
133	a	1525.00	20.96738	YES	YES
134	a	1723.34	534.36313	YES	YES
135	a	3046.64	10.05093	YES	YES
136	a	3048.53	8.54537	YES	YES
137	a	3063.50	10.75631	YES	YES
138	a	3068.35	15.85531	YES	YES
139	a	3101.62	0.57189	YES	YES
140	a	3108.05	0.47650	YES	YES
141	a	3114.78	14.17433	YES	YES
142	a	3119.79	13.53465	YES	YES
143	a	3127.48	23.43068	YES	YES
144	a	3132.72	21.00027	YES	YES

$end

Total COSMO energy + OC corr. = -4656.5468875227 H

[Li(DEC)(oDFB)$_2$]$^+$

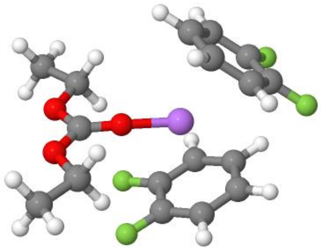

Method: (RI-)B3LYP(D3BJ)/def2-TZVPP
Symmetry: c1

Cartesian coordinates in Ångström:

C	1.5621388	-0.7873707	2.6353165
C	1.6098766	-2.1235367	2.2481656
C	0.8035768	-2.5874643	1.2211799
H	0.8535108	-3.6318846	0.9463719
C	-0.0640516	-1.7012986	0.5802472
H	-0.7089184	-2.0588328	-0.2094171
C	-0.1144448	-0.3631427	0.9703080
H	-0.7974666	0.3190018	0.4856987
C	0.7058345	0.0988229	2.0010493
H	0.6781207	1.1288547	2.3283734
F	2.3790386	-0.3631888	3.6038233
F	2.4709367	-2.9444562	2.8557098
C	1.5286804	-0.7721216	-2.8767154
H	1.1789011	-1.7055830	-3.2952342
C	2.8812210	-0.4255969	-2.8830674
H	3.6031186	-1.1037815	-3.3147597
C	3.2952832	0.7948608	-2.3512960
H	4.3408671	1.0661724	-2.3648986
C	2.3614930	1.6764758	-1.8057480
H	2.6533100	2.6336557	-1.3965943
C	1.0177412	1.3310285	-1.8079736
C	0.6030690	0.1129046	-2.3435710
F	0.1072617	2.1540208	-1.2844853
F	-0.6943931	-0.1986883	-2.3199495
Li	2.3977562	-0.6582049	-0.1926928
H	4.1773483	-3.5972835	1.0329151
H	6.1995502	-4.7161396	1.9961810
H	4.0084849	1.0931300	2.2538818
C	5.1714838	-3.4082863	0.6315824
O	3.9878386	-0.9704667	0.5236196
H	5.7633634	-5.4477060	0.4479061
C	6.1266696	-4.5370283	0.9246101
C	5.0297557	-1.0990967	1.1707968
O	5.6978155	-2.2150812	1.2880306
C	5.0024958	1.1860351	1.8188162
H	5.9763069	1.7484749	3.6524716
O	5.6205434	-0.1337964	1.8273950
H	5.0940883	-3.1977960	-0.4347709
C	5.8882069	2.1009113	2.6260497
H	5.4539797	3.1008119	2.6422948
H	7.1206748	-4.3193458	0.5367943
H	4.9116674	1.5089185	0.7815953
H	6.8842654	2.1652784	2.1907297

SCF energy GEOOPT = -1291.026281410 H
ZPE = 852.9 kJ/mol
FREEH energy = 923.45 kJ/mol

FREEH entropy = 0.81120 kJ/mol/K

$vibrational spectrum

# mode	symmetry	wave number cm**(-1)	IR intensity km/mol	selection rules IR	RAMAN
1		-0.00	0.00000	-	-
2		-0.00	0.00000	-	-
3		0.00	0.00000	-	-
4		0.00	0.00000	-	-
5		0.00	0.00000	-	-
6		0.00	0.00000	-	-
7	a	8.89	0.31490	YES	YES
8	a	13.91	0.07292	YES	YES
9	a	20.07	2.24691	YES	YES
10	a	24.90	0.52831	YES	YES
11	a	32.28	0.01483	YES	YES
12	a	38.94	0.97852	YES	YES
13	a	44.03	1.02162	YES	YES
14	a	52.37	0.55068	YES	YES
15	a	55.97	1.84060	YES	YES
16	a	58.03	0.62173	YES	YES
17	a	67.86	4.30090	YES	YES
18	a	74.99	0.66065	YES	YES
19	a	96.02	1.13138	YES	YES
20	a	104.27	0.56940	YES	YES
21	a	132.82	3.40264	YES	YES
22	a	140.45	6.55604	YES	YES
23	a	141.04	9.84575	YES	YES
24	a	161.16	0.01482	YES	YES
25	a	198.43	0.06551	YES	YES
26	a	200.61	0.07550	YES	YES
27	a	253.63	4.78712	YES	YES
28	a	257.82	0.64555	YES	YES
29	a	265.04	0.03085	YES	YES
30	a	281.46	131.79592	YES	YES
31	a	291.12	0.38628	YES	YES
32	a	291.56	0.33392	YES	YES
33	a	305.98	1.13687	YES	YES
34	a	313.10	1.13931	YES	YES
35	a	346.08	0.21361	YES	YES
36	a	385.58	21.81464	YES	YES
37	a	447.13	0.12554	YES	YES
38	a	447.41	0.04026	YES	YES
39	a	468.85	31.07714	YES	YES
40	a	475.85	8.33363	YES	YES
41	a	477.83	6.35864	YES	YES
42	a	556.02	2.77322	YES	YES
43	a	556.31	4.89256	YES	YES
44	a	576.57	0.04823	YES	YES
45	a	580.69	0.13001	YES	YES
46	a	584.60	48.00870	YES	YES
47	a	588.18	7.97833	YES	YES
48	a	588.81	34.04955	YES	YES
49	a	734.86	0.01422	YES	YES
50	a	737.95	0.98277	YES	YES
51	a	738.01	0.32905	YES	YES
52	a	781.94	12.43396	YES	YES
53	a	783.43	49.67897	YES	YES
54	a	795.78	131.99206	YES	YES
55	a	804.70	14.54809	YES	YES
56	a	809.46	115.86612	YES	YES
57	a	824.43	0.05347	YES	YES
58	a	830.05	0.13811	YES	YES

59	a	854.64	53.75457	YES	YES
60	a	872.86	29.03684	YES	YES
61	a	873.56	1.03056	YES	YES
62	a	888.76	0.13956	YES	YES
63	a	893.11	0.85066	YES	YES
64	a	927.50	0.84306	YES	YES
65	a	973.87	6.71274	YES	YES
66	a	976.97	2.22655	YES	YES
67	a	1012.00	0.20015	YES	YES
68	a	1020.42	22.32351	YES	YES
69	a	1021.24	83.15294	YES	YES
70	a	1023.50	18.20065	YES	YES
71	a	1048.50	2.81134	YES	YES
72	a	1048.64	8.33998	YES	YES
73	a	1127.91	1.46304	YES	YES
74	a	1129.01	19.66552	YES	YES
75	a	1134.84	0.04619	YES	YES
76	a	1138.56	1.43015	YES	YES
77	a	1179.89	0.53635	YES	YES
78	a	1180.72	2.17686	YES	YES
79	a	1182.04	2.11773	YES	YES
80	a	1182.47	0.47547	YES	YES
81	a	1183.74	4.78924	YES	YES
82	a	1234.50	17.34597	YES	YES
83	a	1238.11	21.52164	YES	YES
84	a	1296.55	0.93561	YES	YES
85	a	1298.12	8.33741	YES	YES
86	a	1303.81	49.22924	YES	YES
87	a	1304.24	16.47933	YES	YES
88	a	1306.63	132.61623	YES	YES
89	a	1307.60	22.37780	YES	YES
90	a	1323.32	1.34540	YES	YES
91	a	1327.17	0.35756	YES	YES
92	a	1350.04	569.74231	YES	YES
93	a	1398.98	20.02040	YES	YES
94	a	1426.05	67.89994	YES	YES
95	a	1430.74	15.53027	YES	YES
96	a	1473.71	236.74813	YES	YES
97	a	1485.81	2.58726	YES	YES
98	a	1486.01	13.89273	YES	YES
99	a	1490.42	1.51364	YES	YES
100	a	1491.76	50.31846	YES	YES
101	a	1499.23	4.33467	YES	YES
102	a	1499.44	5.18520	YES	YES
103	a	1515.60	12.67493	YES	YES
104	a	1524.75	51.41791	YES	YES
105	a	1541.14	76.07163	YES	YES
106	a	1545.12	262.59766	YES	YES
107	a	1630.89	15.30451	YES	YES
108	a	1632.05	15.82251	YES	YES
109	a	1633.49	20.41540	YES	YES
110	a	1635.73	16.81071	YES	YES
111	a	1693.13	666.28083	YES	YES
112	a	3050.97	5.66933	YES	YES
113	a	3051.13	4.21412	YES	YES
114	a	3056.50	19.87823	YES	YES
115	a	3061.81	18.88486	YES	YES
116	a	3103.53	3.26413	YES	YES
117	a	3107.64	2.03763	YES	YES
118	a	3119.37	4.87986	YES	YES
119	a	3119.44	13.86506	YES	YES
120	a	3133.46	10.34792	YES	YES
121	a	3134.24	21.26263	YES	YES

```
   122         a            3191.68          1.12802       YES      YES
   123         a            3193.73          0.49011       YES      YES
   124         a            3198.11          0.86655       YES      YES
   125         a            3198.49          0.73675       YES      YES
   126         a            3203.86          1.69993       YES      YES
   127         a            3204.99          2.36644       YES      YES
   128         a            3211.28          0.30362       YES      YES
   129         a            3213.49          1.25824       YES      YES
$end
```

```
Total COSMO energy + OC corr. = -1291.0884603836 H
```

[Li(DEC)$_2$(*o*DFB)]$^+$

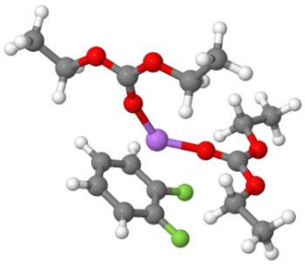

```
Method: (RI-)B3LYP(D3BJ)/def2-TZVPP
Symmetry: c1

Cartesian coordinates in Ångström:
Li   -0.2342117   -0.2975720   -0.2259379
O     1.4148622   -1.0068150   -0.1525216
H     3.0784869   -4.9337665   -1.8303890
H     5.3981919    0.8326321    1.2122452
H     2.9546269    1.0218327    0.7025998
H     1.1629674   -3.4620158   -1.1405571
H     4.5129078    1.4237899    2.6228454
H     1.9335359   -4.7292458   -3.1615638
C     2.6532711   -4.2122383   -2.5265124
C     4.6560922    0.5673318    1.9636673
C     2.5700314   -1.4266291   -0.2183995
C     3.3409286    0.2113361    1.3181984
C     1.9569295   -3.0951730   -1.7912319
O     2.9548937   -2.4361297   -0.9592990
O     3.5824269   -0.9347596    0.4487985
H     3.4506732   -3.8230363   -3.1576431
H     5.0385423   -0.2623095    2.5564463
H     2.5853221   -0.0735991    2.0499916
H     1.5446652   -2.3524337   -2.4722372
O    -1.6956717   -0.6635357    0.7573092
H    -1.6493737   -4.9588065    2.4077399
H    -5.3500128    0.8629385    3.0640859
H    -3.3206702    1.2490143    1.6356187
H    -0.8862649   -3.2265530    0.7607753
H    -5.7842999    1.7181828    1.5800536
H     0.1111743   -4.8036123    2.4334489
C    -0.8255102   -4.2889801    2.6493020
C    -5.4102489    0.7755687    1.9802774
C    -2.4317789   -1.2327358    1.5616324
C    -4.0559351    0.4849097    1.3852923
C    -0.9004365   -3.0238787    1.8321584
O    -2.1627016   -2.3739455    2.1512337
```

```
O    -3.5976029   -0.7810708    1.9478380
H    -0.8571005   -4.0666498    3.7148388
H    -6.1227108   -0.0095959    1.7315950
H    -4.0938306    0.3760175    0.3018971
H    -0.0911596   -2.3329805    2.0674053
C     0.2863672    2.3874386   -1.1899057
C     1.2548454    1.6842148   -1.8876056
C    -1.0602984    2.1043407   -1.4190518
C    -0.4437099    0.4163641   -3.0357312
C     0.8917665    0.7039033   -2.8052960
C    -1.4240629    1.1226872   -2.3383574
F     2.5524329    1.9154505   -1.6635703
F     1.8503650    0.0186028   -3.4372748
H    -1.8189701    2.6580820   -0.8854550
H    -2.4668171    0.9093838   -2.5236328
H    -0.6991429   -0.3433406   -3.7612763
H     0.5953253    3.1500464   -0.4888448

SCF energy GEOOPT =  -1282.564822801 H
ZPE =   1030. kJ/mol
FREEH energy =  1109.95 kJ/mol
FREEH entropy =   0.92142 kJ/mol/K

$vibrational spectrum
#  mode     symmetry     wave number   IR intensity    selection rules
#                         cm**(-1)        km/mol          IR     RAMAN
     1                      -0.00         0.00000         -        -
     2                       0.00         0.00000         -        -
     3                       0.00         0.00000         -        -
     4                       0.00         0.00000         -        -
     5                       0.00         0.00000         -        -
     6                       0.00         0.00000         -        -
     7         a             2.63         0.00638        YES      YES
     8         a             8.70         1.09533        YES      YES
     9         a            11.18         0.11392        YES      YES
    10         a            15.22         0.48464        YES      YES
    11         a            23.93         0.19981        YES      YES
    12         a            29.95         0.00796        YES      YES
    13         a            31.40         0.42229        YES      YES
    14         a            37.73         1.09776        YES      YES
    15         a            48.11         0.33333        YES      YES
    16         a            52.92         2.81648        YES      YES
    17         a            56.83         0.29008        YES      YES
    18         a            61.95         0.26839        YES      YES
    19         a            66.89         2.25428        YES      YES
    20         a            73.57         0.03428        YES      YES
    21         a            80.75         0.17258        YES      YES
    22         a            98.60         1.60715        YES      YES
    23         a           111.59         1.11656        YES      YES
    24         a           124.59         1.97664        YES      YES
    25         a           134.78         7.39238        YES      YES
    26         a           148.11         1.87476        YES      YES
    27         a           159.92         3.79719        YES      YES
    28         a           160.68         0.02525        YES      YES
    29         a           167.74        16.71603        YES      YES
    30         a           197.36         0.01251        YES      YES
    31         a           253.99         5.40583        YES      YES
    32         a           256.56         2.66828        YES      YES
    33         a           257.49         1.24060        YES      YES
    34         a           257.96         6.39659        YES      YES
    35         a           264.43         0.02315        YES      YES
    36         a           264.90         0.02892        YES      YES
    37         a           290.83         0.20480        YES      YES
```

38	a	306.41	0.08466	YES	YES
39	a	340.64	30.73041	YES	YES
40	a	346.29	0.68374	YES	YES
41	a	371.97	103.85857	YES	YES
42	a	386.15	21.87344	YES	YES
43	a	394.66	14.10998	YES	YES
44	a	446.91	0.06094	YES	YES
45	a	476.03	3.84805	YES	YES
46	a	486.81	22.03606	YES	YES
47	a	531.53	16.29024	YES	YES
48	a	555.83	3.86639	YES	YES
49	a	576.83	0.00927	YES	YES
50	a	587.54	3.91180	YES	YES
51	a	630.44	157.23843	YES	YES
52	a	736.95	0.02588	YES	YES
53	a	738.72	1.23394	YES	YES
54	a	740.60	1.12912	YES	YES
55	a	782.39	32.17313	YES	YES
56	a	791.53	111.78539	YES	YES
57	a	804.62	29.90491	YES	YES
58	a	805.25	21.00203	YES	YES
59	a	821.41	0.15594	YES	YES
60	a	823.61	0.07026	YES	YES
61	a	828.75	0.70399	YES	YES
62	a	829.43	0.83070	YES	YES
63	a	856.33	46.17472	YES	YES
64	a	857.06	67.53817	YES	YES
65	a	872.85	16.20908	YES	YES
66	a	887.22	0.60114	YES	YES
67	a	923.68	0.20174	YES	YES
68	a	928.38	1.85137	YES	YES
69	a	971.10	5.29164	YES	YES
70	a	1008.17	0.04054	YES	YES
71	a	1021.59	41.95222	YES	YES
72	a	1022.25	71.13890	YES	YES
73	a	1023.85	50.24523	YES	YES
74	a	1024.57	106.17670	YES	YES
75	a	1049.59	6.39377	YES	YES
76	a	1128.31	12.60005	YES	YES
77	a	1134.31	0.19864	YES	YES
78	a	1134.84	0.07032	YES	YES
79	a	1138.41	4.27185	YES	YES
80	a	1138.73	0.81612	YES	YES
81	a	1173.92	2.36468	YES	YES
82	a	1177.85	0.66318	YES	YES
83	a	1178.57	0.50926	YES	YES
84	a	1179.99	0.58194	YES	YES
85	a	1181.07	2.39730	YES	YES
86	a	1182.78	2.74528	YES	YES
87	a	1183.43	6.95225	YES	YES
88	a	1233.27	13.41251	YES	YES
89	a	1296.38	0.86897	YES	YES
90	a	1301.82	1.84716	YES	YES
91	a	1303.03	68.94972	YES	YES
92	a	1304.20	14.65480	YES	YES
93	a	1305.94	2.06921	YES	YES
94	a	1306.93	23.60221	YES	YES
95	a	1327.89	1.20289	YES	YES
96	a	1345.83	545.84376	YES	YES
97	a	1347.96	777.96165	YES	YES
98	a	1399.14	25.78062	YES	YES
99	a	1399.50	11.44128	YES	YES
100	a	1425.33	66.64649	YES	YES

101	a	1425.44	86.06696	YES	YES
102	a	1430.51	18.81638	YES	YES
103	a	1430.93	13.41784	YES	YES
104	a	1467.72	204.21875	YES	YES
105	a	1469.98	272.05589	YES	YES
106	a	1485.64	8.20316	YES	YES
107	a	1486.05	3.19056	YES	YES
108	a	1486.07	11.10473	YES	YES
109	a	1486.26	12.31262	YES	YES
110	a	1491.79	29.17496	YES	YES
111	a	1498.44	1.18541	YES	YES
112	a	1498.88	1.28348	YES	YES
113	a	1499.12	4.28686	YES	YES
114	a	1499.52	4.26953	YES	YES
115	a	1515.24	44.99331	YES	YES
116	a	1516.64	12.36859	YES	YES
117	a	1522.92	33.79662	YES	YES
118	a	1523.89	39.55366	YES	YES
119	a	1544.54	183.57357	YES	YES
120	a	1634.17	12.97721	YES	YES
121	a	1638.92	23.72496	YES	YES
122	a	1691.09	903.54987	YES	YES
123	a	1714.35	292.03232	YES	YES
124	a	3049.86	25.38608	YES	YES
125	a	3050.32	8.52244	YES	YES
126	a	3050.46	3.38902	YES	YES
127	a	3050.52	7.91263	YES	YES
128	a	3050.59	3.62014	YES	YES
129	a	3057.37	15.64634	YES	YES
130	a	3057.81	24.64652	YES	YES
131	a	3060.89	20.23209	YES	YES
132	a	3093.32	7.48184	YES	YES
133	a	3099.46	5.72111	YES	YES
134	a	3104.51	2.69497	YES	YES
135	a	3106.86	2.09696	YES	YES
136	a	3117.83	11.30030	YES	YES
137	a	3118.51	11.21834	YES	YES
138	a	3118.91	7.67335	YES	YES
139	a	3119.06	12.62240	YES	YES
140	a	3130.87	8.04153	YES	YES
141	a	3131.28	24.50094	YES	YES
142	a	3132.63	11.59740	YES	YES
143	a	3133.37	24.20239	YES	YES
144	a	3191.44	0.77003	YES	YES
145	a	3198.33	2.54379	YES	YES
146	a	3204.32	0.71181	YES	YES
147	a	3211.82	0.25090	YES	YES

$end

Total COSMO energy + OC corr. = -1282.6262448872 H

Li(DME)(DEC)(*o*DFB)]$^+$

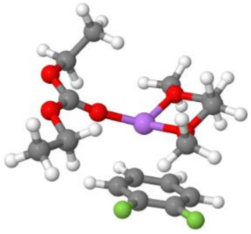

```
Method: (RI-)B3LYP(D3BJ)/def2-TZVPP
Symmetry: c1

Cartesian coordinates in Ångström:
C     1.0244738    1.9571326   -1.4839758
O     0.5582779    1.4420281   -0.2354932
Li   -0.9709232    0.3248183   -0.5690715
O    -0.4625119    0.3584958   -2.4193992
C     0.8740423    0.8625851   -2.5135548
C     0.8201824    2.2983778    0.8793173
H     2.0749359    2.2482930   -1.4051253
H     0.4353384    2.8379023   -1.7577936
C    -0.7990962   -0.5645702   -3.4551447
H     1.0561108    1.2648487   -3.5130868
H     1.5782789    0.0476574   -2.3246812
H    -1.8164019   -0.8985948   -3.2696337
H    -0.1246131   -1.4227859   -3.4396996
H    -0.7488462   -0.0743290   -4.4286846
H     0.4366068    1.7979047    1.7634682
H     0.3221868    3.2618516    0.7482144
H     1.8942995    2.4510529    0.9906387
H     2.7793297   -1.9782025   -0.4413743
F     2.2828955   -0.5822140    1.6861369
C     1.7510125   -2.0202404   -0.1108274
C     1.3867904   -1.3247547    1.0276056
H     1.0759877   -3.3181217   -1.6743725
C     0.7909574   -2.7609815   -0.7932966
C     0.0777358   -1.3748032    1.4931547
F    -0.2496677   -0.6817487    2.5976795
C    -0.5215042   -2.8037794   -0.3342766
C    -0.8835825   -2.1113097    0.8202705
H    -1.2620595   -3.3939095   -0.8549408
H    -1.8894634   -2.1463521    1.2133327
O    -2.6330829    0.5937971    0.0732551
H    -3.1843773    1.6340916   -2.4398350
H    -4.2933590    1.5543333    4.2106879
H    -2.4807823    0.7142755    2.6882372
H    -4.0463400   -0.5739296   -1.6047367
H    -2.7708653    2.3932151    4.5251158
H    -4.2180907    0.8861795   -3.6614098
C    -4.2038863    1.3202742   -2.6606763
C    -3.5604272    2.2405809    3.7889965
C    -3.7552878    0.9602738    0.4115511
C    -2.9596573    1.6782331    2.5259455
C    -4.7000421    0.2949822   -1.6648312
O    -4.8332887    0.8625929   -0.3316685
O    -4.0477636    1.4942166    1.5713096
H    -4.8510452    2.1962999   -2.6653389
H    -4.0451727    3.1973789    3.6004814
H    -2.2350264    2.3578540    2.0773007
H    -5.7078777   -0.0359601   -1.8983504
```

```
SCF energy GEOOPT =  -1169.195112371 H
ZPE =   1007. kJ/mol
FREEH energy =  1080.55 kJ/mol
FREEH entropy =   0.82952 kJ/mol/K

$vibrational spectrum
#  mode     symmetry     wave number   IR intensity    selection rules
#                         cm**(-1)        km/mol         IR     RAMAN
     1                     -0.00         0.00000         -        -
     2                     -0.00         0.00000         -        -
     3                      0.00         0.00000         -        -
     4                      0.00         0.00000         -        -
     5                      0.00         0.00000         -        -
     6                      0.00         0.00000         -        -
     7         a           10.43         0.43665        YES      YES
     8         a           16.04         0.72554        YES      YES
     9         a           22.59         0.62551        YES      YES
    10         a           23.68         0.24163        YES      YES
    11         a           26.37         0.16203        YES      YES
    12         a           34.69         0.70802        YES      YES
    13         a           49.83         0.78651        YES      YES
    14         a           53.96         0.63963        YES      YES
    15         a           60.27         0.43059        YES      YES
    16         a           64.50         0.48650        YES      YES
    17         a           68.37         0.28311        YES      YES
    18         a           81.93         0.90477        YES      YES
    19         a           90.09         0.72973        YES      YES
    20         a          102.91         2.22389        YES      YES
    21         a          113.68         0.35242        YES      YES
    22         a          129.31         3.99714        YES      YES
    23         a          136.17         2.10026        YES      YES
    24         a          149.20         1.13110        YES      YES
    25         a          165.61         0.09752        YES      YES
    26         a          173.01         3.38371        YES      YES
    27         a          197.98         0.11548        YES      YES
    28         a          214.45         0.53851        YES      YES
    29         a          221.71         1.46857        YES      YES
    30         a          229.78         2.21761        YES      YES
    31         a          260.22         0.26833        YES      YES
    32         a          278.95        15.96183        YES      YES
    33         a          290.81         2.56072        YES      YES
    34         a          293.56        40.24150        YES      YES
    35         a          310.23        23.30599        YES      YES
    36         a          327.00         7.29868        YES      YES
    37         a          332.32        15.64718        YES      YES
    38         a          360.89        16.08821        YES      YES
    39         a          371.08        21.97527        YES      YES
    40         a          415.21         5.32395        YES      YES
    41         a          423.25        24.83289        YES      YES
    42         a          445.95         0.12956        YES      YES
    43         a          474.26         4.12406        YES      YES
    44         a          520.82        11.15845        YES      YES
    45         a          554.79         3.99819        YES      YES
    46         a          573.32         0.77655        YES      YES
    47         a          574.94         1.48757        YES      YES
    48         a          585.91         4.81948        YES      YES
    49         a          610.51       129.70531        YES      YES
    50         a          712.21         1.11720        YES      YES
    51         a          731.32         0.46279        YES      YES
    52         a          779.95        25.51306        YES      YES
    53         a          782.96       104.09874        YES      YES
    54         a          805.94        19.77440        YES      YES
```

55	a	817.55	1.69842	YES	YES
56	a	826.15	0.80598	YES	YES
57	a	843.18	7.59648	YES	YES
58	a	852.75	47.25769	YES	YES
59	a	867.59	16.96146	YES	YES
60	a	879.09	9.19630	YES	YES
61	a	881.35	43.10710	YES	YES
62	a	921.78	1.53608	YES	YES
63	a	967.24	7.23150	YES	YES
64	a	1008.31	20.99170	YES	YES
65	a	1009.32	0.37618	YES	YES
66	a	1022.46	84.37086	YES	YES
67	a	1027.39	15.53734	YES	YES
68	a	1040.97	8.52185	YES	YES
69	a	1050.16	3.67136	YES	YES
70	a	1094.91	239.90565	YES	YES
71	a	1110.90	21.06587	YES	YES
72	a	1127.93	8.67456	YES	YES
73	a	1129.07	3.90894	YES	YES
74	a	1130.77	60.23919	YES	YES
75	a	1136.76	2.19485	YES	YES
76	a	1154.38	0.41882	YES	YES
77	a	1180.02	5.22559	YES	YES
78	a	1180.98	3.99516	YES	YES
79	a	1181.62	0.70622	YES	YES
80	a	1187.03	1.70687	YES	YES
81	a	1208.72	12.94463	YES	YES
82	a	1215.38	14.28603	YES	YES
83	a	1227.07	39.54924	YES	YES
84	a	1236.48	0.87237	YES	YES
85	a	1268.98	10.84876	YES	YES
86	a	1293.96	23.57879	YES	YES
87	a	1301.77	67.38925	YES	YES
88	a	1303.69	13.59021	YES	YES
89	a	1305.68	7.73675	YES	YES
90	a	1331.49	0.52292	YES	YES
91	a	1331.90	1.20936	YES	YES
92	a	1348.65	619.16929	YES	YES
93	a	1400.65	15.57855	YES	YES
94	a	1404.14	13.56843	YES	YES
95	a	1420.71	41.08488	YES	YES
96	a	1428.37	37.80557	YES	YES
97	a	1438.91	1.95783	YES	YES
98	a	1461.07	191.38230	YES	YES
99	a	1484.23	9.48579	YES	YES
100	a	1486.20	8.45960	YES	YES
101	a	1486.84	19.98934	YES	YES
102	a	1487.79	0.29651	YES	YES
103	a	1493.28	4.25005	YES	YES
104	a	1493.54	4.84848	YES	YES
105	a	1494.26	21.33412	YES	YES
106	a	1498.59	10.07090	YES	YES
107	a	1500.06	5.89188	YES	YES
108	a	1501.27	9.51031	YES	YES
109	a	1503.21	10.07150	YES	YES
110	a	1512.00	46.45385	YES	YES
111	a	1514.95	17.29175	YES	YES
112	a	1518.26	11.44491	YES	YES
113	a	1524.34	29.28307	YES	YES
114	a	1544.25	177.84042	YES	YES
115	a	1638.43	8.08101	YES	YES
116	a	1643.46	20.53668	YES	YES
117	a	1711.93	592.96390	YES	YES

118	a	3015.94	16.92760	YES	YES
119	a	3023.41	15.67563	YES	YES
120	a	3025.01	33.61589	YES	YES
121	a	3028.92	26.31118	YES	YES
122	a	3045.32	5.97190	YES	YES
123	a	3050.22	5.48763	YES	YES
124	a	3059.74	18.90048	YES	YES
125	a	3063.02	16.06547	YES	YES
126	a	3074.67	13.58036	YES	YES
127	a	3075.21	34.10563	YES	YES
128	a	3092.86	9.87398	YES	YES
129	a	3093.18	23.15630	YES	YES
130	a	3106.80	1.55486	YES	YES
131	a	3109.50	12.60525	YES	YES
132	a	3117.62	5.19996	YES	YES
133	a	3118.55	10.36212	YES	YES
134	a	3132.74	18.85573	YES	YES
135	a	3140.96	12.53200	YES	YES
136	a	3144.33	17.44414	YES	YES
137	a	3152.29	6.22501	YES	YES
138	a	3188.96	0.51957	YES	YES
139	a	3198.46	2.50839	YES	YES
140	a	3205.23	0.06585	YES	YES
141	a	3210.81	0.37386	YES	YES

$end

Total COSMO energy + OC corr. = -1169.2545161660 H

(*o*DFB)Li[Ga(C_2F_5)$_4$]

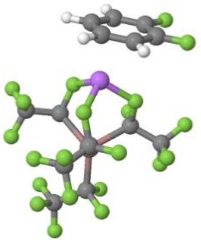

Method: (RI-)B3LYP(D3BJ)/def2-TZVPP
Symmetry: c1

Cartesian coordinates in Ångström:

F	1.8697692	3.4828073	-2.3572518
F	3.7262916	3.4981471	-1.2336479
F	-2.6532716	4.1763396	-0.9084048
C	2.3906781	3.5034471	-1.1284778
F	2.5532855	1.2116436	-0.7393680
F	2.0330384	4.6537092	-0.5310722
C	1.8751281	2.3019436	-0.3150117
C	-1.5961575	4.6585687	-0.2478701
F	-1.9786438	5.7554837	0.4217630
F	-2.0414195	3.2873540	1.5743563
F	0.4404905	1.1690637	-3.3420888
C	-1.0527675	3.5696168	0.6890078
Ga	-0.1534845	1.8608906	-0.0870849
F	-0.6625161	5.0180536	-1.1385374
F	2.3941377	2.5661286	1.0093634
F	-0.8134574	-0.4486361	-1.5754706
C	-0.8490910	1.3733866	-3.0301083

```
C    -1.1219818    0.8871399   -1.5979235
F    -0.0488373    4.2343540    1.4818409
F    -1.6040231    0.7448667   -3.9353229
F    -1.0966633    2.6923824   -3.1180864
F    -2.4769243    0.9868338   -1.3961653
F    -2.2286802    0.5824328    2.2973802
F     0.3079232    1.5986663    2.7258461
C    -1.1296891   -0.1667893    2.1436990
F    -0.8483615   -0.7433941    3.3206131
C     0.0377284    0.6924605    1.6333495
F    -1.3897375   -1.1289776    1.2530929
F     1.1300346   -0.1122237    1.5871499
Li    1.2702828    3.2051139    2.4075137
C     0.9278653    4.2812393    4.9723798
C     1.2270627    3.0315298    5.4878667
C     1.8400457    4.9054437    4.1190327
C     3.3267269    3.0050748    4.3002863
C     2.4219488    2.3957315    5.1528569
C     3.0355503    4.2694713    3.7841462
F     0.3718109    2.4155714    6.3028483
F     2.6765404    1.1886922    5.6561498
H     1.6143126    5.8846975    3.7216619
H     3.7430546    4.7522679    3.1252138
H     4.2402864    2.4860624    4.0476903
H    -0.0096554    4.7476549    5.2395638

SCF energy GEOOPT =  -4664.996143725 H
ZPE =   498.0 kJ/mol
FREEH energy =   600.90 kJ/mol
FREEH entropy =   1.05234 kJ/mol/K

$vibrational spectrum
#  mode     symmetry     wave number   IR intensity    selection rules
#                         cm**(-1)        km/mol          IR     RAMAN
     1                      -0.00         0.00000         -        -
     2                      -0.00         0.00000         -        -
     3                      -0.00         0.00000         -        -
     4                      -0.00         0.00000         -        -
     5                       0.00         0.00000         -        -
     6                       0.00         0.00000         -        -
     7         a             6.15         0.54074        YES      YES
     8         a            14.09         0.05757        YES      YES
     9         a            18.51         0.16131        YES      YES
    10         a            25.23         0.05211        YES      YES
    11         a            28.05         0.01790        YES      YES
    12         a            31.72         0.35104        YES      YES
    13         a            36.32         0.04129        YES      YES
    14         a            45.39         0.02048        YES      YES
    15         a            49.62         0.14499        YES      YES
    16         a            50.78         0.54395        YES      YES
    17         a            53.47         0.22737        YES      YES
    18         a            58.50         0.43118        YES      YES
    19         a            59.91         0.32489        YES      YES
    20         a            66.86         0.26041        YES      YES
    21         a            70.91         0.74785        YES      YES
    22         a            78.72         1.38296        YES      YES
    23         a            84.88         1.68875        YES      YES
    24         a            86.17         0.46188        YES      YES
    25         a            94.34         0.39613        YES      YES
    26         a           105.29         0.40351        YES      YES
    27         a           111.90         1.14465        YES      YES
    28         a           125.31         1.22190        YES      YES
    29         a           145.37         2.16499        YES      YES
```

30	a	181.87	0.09108	YES	YES
31	a	192.41	0.30110	YES	YES
32	a	193.04	1.54776	YES	YES
33	a	195.29	0.21504	YES	YES
34	a	208.22	0.86473	YES	YES
35	a	210.67	1.88290	YES	YES
36	a	218.06	0.54922	YES	YES
37	a	222.71	0.28552	YES	YES
38	a	228.88	2.76236	YES	YES
39	a	241.29	9.02904	YES	YES
40	a	250.10	4.07136	YES	YES
41	a	274.54	1.74441	YES	YES
42	a	277.28	2.65757	YES	YES
43	a	282.51	5.71241	YES	YES
44	a	290.66	0.50311	YES	YES
45	a	293.90	10.54736	YES	YES
46	a	299.00	9.75074	YES	YES
47	a	306.98	0.97232	YES	YES
48	a	319.31	126.77879	YES	YES
49	a	350.70	9.68301	YES	YES
50	a	356.21	10.14970	YES	YES
51	a	361.18	10.72715	YES	YES
52	a	364.37	0.52416	YES	YES
53	a	384.26	31.33163	YES	YES
54	a	388.69	56.39700	YES	YES
55	a	394.37	54.79172	YES	YES
56	a	426.95	2.05150	YES	YES
57	a	432.72	10.17843	YES	YES
58	a	438.60	41.06759	YES	YES
59	a	443.71	19.41896	YES	YES
60	a	447.32	0.03130	YES	YES
61	a	471.80	4.56177	YES	YES
62	a	521.94	1.91066	YES	YES
63	a	524.24	3.20653	YES	YES
64	a	524.70	5.93223	YES	YES
65	a	529.49	1.45657	YES	YES
66	a	555.21	4.06464	YES	YES
67	a	576.12	4.88326	YES	YES
68	a	577.33	4.99357	YES	YES
69	a	580.85	27.94734	YES	YES
70	a	581.98	0.22042	YES	YES
71	a	582.60	4.23930	YES	YES
72	a	583.80	1.12431	YES	YES
73	a	585.64	4.35282	YES	YES
74	a	590.19	1.02921	YES	YES
75	a	592.51	21.19340	YES	YES
76	a	600.44	1.56798	YES	YES
77	a	725.89	45.02768	YES	YES
78	a	727.34	16.62478	YES	YES
79	a	731.34	22.51522	YES	YES
80	a	734.03	0.10854	YES	YES
81	a	734.90	14.32493	YES	YES
82	a	783.15	25.83547	YES	YES
83	a	800.70	140.15211	YES	YES
84	a	871.79	26.16818	YES	YES
85	a	872.94	13.11771	YES	YES
86	a	881.01	47.74792	YES	YES
87	a	887.37	12.78959	YES	YES
88	a	909.49	261.75915	YES	YES
89	a	934.24	82.78499	YES	YES
90	a	944.95	36.79667	YES	YES
91	a	946.50	33.83896	YES	YES
92	a	949.86	58.68993	YES	YES

93	a	969.21	5.05461	YES	YES
94	a	1008.53	0.04736	YES	YES
95	a	1045.19	6.09413	YES	YES
96	a	1054.90	70.58499	YES	YES
97	a	1109.69	114.33673	YES	YES
98	a	1125.69	56.00064	YES	YES
99	a	1127.90	5.43977	YES	YES
100	a	1133.39	116.57172	YES	YES
101	a	1146.57	137.41047	YES	YES
102	a	1170.23	69.94285	YES	YES
103	a	1173.03	469.68685	YES	YES
104	a	1181.36	1.28519	YES	YES
105	a	1185.37	296.11909	YES	YES
106	a	1191.43	160.23504	YES	YES
107	a	1195.98	115.51052	YES	YES
108	a	1202.14	378.84576	YES	YES
109	a	1204.45	200.56524	YES	YES
110	a	1212.91	106.01236	YES	YES
111	a	1240.64	39.35444	YES	YES
112	a	1283.74	344.47213	YES	YES
113	a	1294.96	170.62902	YES	YES
114	a	1298.16	11.97946	YES	YES
115	a	1298.41	156.09795	YES	YES
116	a	1307.66	172.74851	YES	YES
117	a	1312.37	42.64155	YES	YES
118	a	1332.13	4.18965	YES	YES
119	a	1491.44	16.25371	YES	YES
120	a	1545.66	198.08450	YES	YES
121	a	1632.67	28.03917	YES	YES
122	a	1634.33	12.81705	YES	YES
123	a	3192.28	0.39036	YES	YES
124	a	3200.96	0.84932	YES	YES
125	a	3207.20	1.36839	YES	YES
126	a	3212.51	0.17231	YES	YES

$end

Total COSMO energy + OC corr. = -4665.0084784824 H

(FEC)Li[Ga(C_2F_5)$_4$]

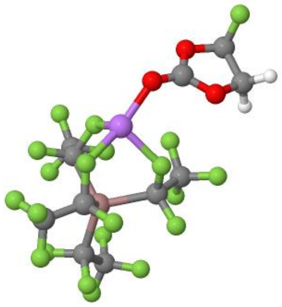

Method: (RI-)B3LYP(D3BJ)/def2-TZVPP
Symmetry: c1

Cartesian coordinates in Ångström:

F	1.4518401	4.6286348	-0.9843443
F	3.1369659	4.6382371	0.3809863
F	-3.1314931	3.7674361	-0.4287635
C	1.8374777	4.3107587	0.2489758
F	2.5661256	2.1317108	-0.1009321
F	1.1521452	5.0810181	1.1226550
C	1.5850019	2.8188404	0.5348005
C	-2.3534455	4.2116212	0.5632398
F	-3.1088748	4.9275861	1.4065489
F	-2.6810640	2.2588656	1.7768214
F	0.9303506	2.4357994	-2.8546475
C	-1.6826150	3.0150404	1.2529681
Ga	-0.2628156	1.8848941	0.2477988
F	-1.4249355	5.0295913	0.0415083
F	1.8881393	2.7015262	1.9283457
F	-0.0344690	0.0831914	-1.9229336
C	-0.3935220	2.2163469	-2.7907413
C	-0.7065470	1.2451984	-1.6413636
F	-1.0142612	3.5699095	2.3915006
F	-0.7984946	1.7548620	-3.9771614
F	-0.9998844	3.3966522	-2.5696091
F	-2.0476212	0.9588605	-1.7201235
F	-2.1652843	-0.4941538	1.7911256
F	-0.0440796	0.8652435	2.9217119
C	-0.8992918	-0.8927830	1.6179430
F	-0.5918996	-1.7488611	2.6028819
C	0.0477356	0.3173972	1.5915862
F	-0.8128614	-1.5431770	0.4522372
F	1.3113853	-0.1746815	1.5131857
Li	0.4681478	2.6746930	3.3052896
F	2.9528256	7.6235440	5.2926612
H	4.5069679	6.3902806	3.8343936
O	3.0367645	4.9416512	3.7135234
C	1.7925734	5.0164136	4.1711551
O	1.0865599	4.0699565	4.4008472
C	3.5150457	6.2671605	3.4109514
C	2.4863267	7.1653563	4.0920893
O	1.3987065	6.2930048	4.3366785
H	2.1319279	8.0148676	3.5157359
H	3.5309563	6.3871263	2.3303440

SCF energy GEOOPT = -4675.959049130 H
ZPE = 454.2 kJ/mol
FREEH energy = 554.97 kJ/mol
FREEH entropy = 1.02264 kJ/mol/K

$vibrational spectrum

# mode	symmetry	wave number cm**(-1)	IR intensity km/mol	selection rules IR	RAMAN
1		0.00	0.00000	-	-
2		0.00	0.00000	-	-
3		0.00	0.00000	-	-
4		0.00	0.00000	-	-
5		0.00	0.00000	-	-
6		0.00	0.00000	-	-
7	a	9.87	0.39523	YES	YES
8	a	19.83	1.80100	YES	YES
9	a	25.33	0.01464	YES	YES
10	a	30.42	0.12291	YES	YES
11	a	38.99	1.17748	YES	YES
12	a	39.89	0.01201	YES	YES
13	a	48.19	0.07679	YES	YES
14	a	49.86	2.87619	YES	YES
15	a	52.74	0.26254	YES	YES
16	a	56.58	1.14583	YES	YES
17	a	60.08	0.08773	YES	YES
18	a	61.93	1.92127	YES	YES
19	a	65.31	0.52469	YES	YES
20	a	69.36	0.76873	YES	YES
21	a	77.97	0.46127	YES	YES
22	a	80.65	0.04938	YES	YES
23	a	85.84	1.23602	YES	YES
24	a	90.88	1.09432	YES	YES
25	a	96.07	0.48529	YES	YES
26	a	105.14	0.27762	YES	YES
27	a	111.32	2.21581	YES	YES
28	a	121.84	0.45697	YES	YES
29	a	143.15	4.43808	YES	YES
30	a	149.72	0.93417	YES	YES
31	a	182.50	0.19796	YES	YES
32	a	192.20	1.76943	YES	YES
33	a	194.90	0.66589	YES	YES
34	a	207.71	0.31670	YES	YES
35	a	211.31	7.53352	YES	YES
36	a	220.20	1.85553	YES	YES
37	a	222.87	1.60915	YES	YES
38	a	225.49	6.91163	YES	YES
39	a	229.65	10.53605	YES	YES
40	a	241.81	8.14904	YES	YES
41	a	250.87	0.50690	YES	YES
42	a	268.57	4.82882	YES	YES
43	a	275.25	9.01659	YES	YES
44	a	282.37	6.59937	YES	YES
45	a	292.53	4.49113	YES	YES
46	a	297.13	10.27458	YES	YES
47	a	316.67	84.65774	YES	YES
48	a	327.06	60.64972	YES	YES
49	a	337.46	32.05185	YES	YES
50	a	362.24	0.08852	YES	YES
51	a	364.14	0.74889	YES	YES
52	a	368.47	2.46044	YES	YES
53	a	370.13	17.04905	YES	YES
54	a	395.56	7.28936	YES	YES
55	a	424.61	0.71660	YES	YES
56	a	426.08	1.19944	YES	YES
57	a	432.34	1.59944	YES	YES
58	a	434.93	7.98146	YES	YES
59	a	472.57	35.74667	YES	YES
60	a	519.26	22.38305	YES	YES

61	a	523.48	3.49367	YES	YES
62	a	525.30	2.20266	YES	YES
63	a	529.62	0.52548	YES	YES
64	a	535.75	141.55689	YES	YES
65	a	555.50	10.77921	YES	YES
66	a	577.20	4.96898	YES	YES
67	a	579.34	9.27606	YES	YES
68	a	581.66	22.52574	YES	YES
69	a	582.29	4.56038	YES	YES
70	a	584.73	0.30589	YES	YES
71	a	591.45	0.83412	YES	YES
72	a	593.98	23.61804	YES	YES
73	a	600.31	2.11307	YES	YES
74	a	726.49	35.86810	YES	YES
75	a	728.44	11.13521	YES	YES
76	a	731.54	18.98056	YES	YES
77	a	734.46	14.06947	YES	YES
78	a	751.93	48.65275	YES	YES
79	a	786.51	47.05929	YES	YES
80	a	829.11	18.02983	YES	YES
81	a	875.54	18.25893	YES	YES
82	a	888.74	35.93051	YES	YES
83	a	908.23	53.72585	YES	YES
84	a	922.84	44.70150	YES	YES
85	a	937.12	310.96355	YES	YES
86	a	939.54	22.62618	YES	YES
87	a	943.64	6.01851	YES	YES
88	a	947.11	38.56697	YES	YES
89	a	947.94	86.95734	YES	YES
90	a	1022.92	159.00281	YES	YES
91	a	1049.39	10.84867	YES	YES
92	a	1054.17	69.57665	YES	YES
93	a	1101.99	223.20715	YES	YES
94	a	1110.27	100.27291	YES	YES
95	a	1122.40	51.10538	YES	YES
96	a	1132.47	156.98514	YES	YES
97	a	1137.47	163.79941	YES	YES
98	a	1139.97	105.58460	YES	YES
99	a	1147.44	53.02496	YES	YES
100	a	1169.72	328.37555	YES	YES
101	a	1183.73	291.71103	YES	YES
102	a	1189.04	95.77982	YES	YES
103	a	1191.15	144.88049	YES	YES
104	a	1197.08	240.63820	YES	YES
105	a	1201.02	462.75171	YES	YES
106	a	1202.56	197.59658	YES	YES
107	a	1211.10	94.63861	YES	YES
108	a	1248.01	49.88532	YES	YES
109	a	1283.22	348.50157	YES	YES
110	a	1295.71	178.67263	YES	YES
111	a	1297.63	171.61339	YES	YES
112	a	1311.99	64.30409	YES	YES
113	a	1367.15	18.46861	YES	YES
114	a	1389.85	51.10017	YES	YES
115	a	1427.21	39.06253	YES	YES
116	a	1506.50	13.86556	YES	YES
117	a	1843.93	749.22176	YES	YES
118	a	3100.68	6.11092	YES	YES
119	a	3141.60	7.81856	YES	YES
120	a	3165.97	0.42069	YES	YES

$end
Total COSMO energy + OC corr. = -4675.9801675248 H

$(FEC)_2Li[Ga(C_2F_5)_4]$

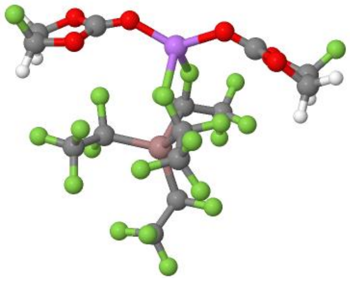

```
Method: (RI-)B3LYP(D3BJ)/def2-TZVPP
Symmetry: c1

Cartesian coordinates in Ångström:
F      0.6495720    4.9339459   -1.1031654
F      2.4939278    5.5557038   -0.1371753
F     -3.4055306    3.2319005    0.6940290
C      1.3236532    4.9006231    0.0456397
F      2.4112492    2.8903286   -0.3870497
F      0.6317768    5.5883864    0.9693669
C      1.5523509    3.4563147    0.5156643
C     -2.6012058    3.9561745    1.4808946
F     -3.3532036    4.5092034    2.4469843
F     -2.1773294    2.0818385    2.7781387
F      0.1309714    2.4542466   -2.5392954
C     -1.5035707    3.0506729    2.0590028
Ga    -0.0410461    2.1757985    0.8621566
F     -2.0653462    4.9428808    0.7545314
F      2.2814780    3.5906101    1.7000316
F     -0.0061504    0.1533559   -1.1423472
C     -1.0423204    2.0307105   -2.0468916
C     -0.8109494    1.2022193   -0.7730660
F     -0.8521490    3.8294557    3.0420880
F     -1.6693609    1.3350408   -3.0027265
F     -1.7898788    3.1128245   -1.7613545
F     -2.0322040    0.6592650   -0.4343870
F     -1.1016234   -0.5389889    2.4793296
F      0.6601085    1.4010618    3.5355653
C      0.2424066   -0.5789192    2.3613703
F      0.7242304   -1.2252649    3.4321265
C      0.8083607    0.8470252    2.2306891
F      0.5412284   -1.2891979    1.2728552
F      2.1505108    0.7110418    2.0615464
Li     0.2218229    3.0257063    4.5510937
F      3.3973063    7.7387839    3.8434431
H      5.1613529    6.0621145    3.4578386
O      3.7553439    4.6433339    3.9945233
C      2.4560045    4.8252747    4.1875969
O      1.7414308    4.0718893    4.7937919
C      4.2387198    5.6404705    3.0716206
C      3.1074090    6.6636658    3.0455915
O      2.0200987    5.9639927    3.6174804
H      2.8155689    7.0296367    2.0665897
H      4.3894550    5.1670811    2.1047289
H     -4.8589375    0.5411687    5.7942314
C     -4.0255728    0.7339044    5.1259287
O     -3.3131162    1.9000045    5.5844939
H     -4.3677496    0.9194652    4.1101805
F     -3.1241349   -1.1254350    6.3221726
C     -2.9696116   -0.3662216    5.1931504
```

```
C    -2.0094070    1.6477875    5.5621886
O    -1.1478217    2.4678052    5.7377068
H    -2.9101863   -1.0306746    4.3368198
O    -1.7576911    0.3514985    5.2978468

SCF energy GEOOPT =  -5117.636490510 H
ZPE =   634.1 kJ/mol
FREEH energy =   754.21 kJ/mol
FREEH entropy =   1.18483 kJ/mol/K

$vibrational spectrum
#  mode     symmetry     wave number   IR intensity    selection rules
#                         cm**(-1)        km/mol          IR     RAMAN
     1                      -0.00         0.00000         -        -
     2                      -0.00         0.00000         -        -
     3                      -0.00         0.00000         -        -
     4                       0.00         0.00000         -        -
     5                       0.00         0.00000         -        -
     6                       0.00         0.00000         -        -
     7         a             9.19         0.90083        YES      YES
     8         a            14.85         1.74193        YES      YES
     9         a            20.75         0.39065        YES      YES
    10         a            28.26         0.42504        YES      YES
    11         a            33.20         0.79818        YES      YES
    12         a            34.91         1.02222        YES      YES
    13         a            37.96         0.27912        YES      YES
    14         a            42.87         2.19581        YES      YES
    15         a            45.80         3.09811        YES      YES
    16         a            48.83         1.05822        YES      YES
    17         a            50.70         0.23149        YES      YES
    18         a            54.38         1.15580        YES      YES
    19         a            55.90         1.62831        YES      YES
    20         a            58.43         1.75144        YES      YES
    21         a            58.82         1.01520        YES      YES
    22         a            62.06         0.51957        YES      YES
    23         a            67.02         1.69906        YES      YES
    24         a            68.47         2.57991        YES      YES
    25         a            72.98         2.75402        YES      YES
    26         a            75.47         0.45070        YES      YES
    27         a            83.63         0.21449        YES      YES
    28         a            85.92         0.89433        YES      YES
    29         a            92.37         1.02995        YES      YES
    30         a            98.71         0.06819        YES      YES
    31         a           102.61         0.44110        YES      YES
    32         a           105.98         2.05231        YES      YES
    33         a           111.31         1.01880        YES      YES
    34         a           119.94         0.30167        YES      YES
    35         a           136.84         0.41575        YES      YES
    36         a           143.85         2.33903        YES      YES
    37         a           154.51         0.42531        YES      YES
    38         a           181.67         0.83479        YES      YES
    39         a           192.92         3.30413        YES      YES
    40         a           194.56         0.83756        YES      YES
    41         a           208.12         6.18803        YES      YES
    42         a           210.20         1.96037        YES      YES
    43         a           213.44         6.68652        YES      YES
    44         a           216.01        13.34354        YES      YES
    45         a           219.90         3.80145        YES      YES
    46         a           225.53         0.18343        YES      YES
    47         a           230.92         0.71989        YES      YES
    48         a           241.09         3.56985        YES      YES
    49         a           244.66         0.42751        YES      YES
    50         a           270.03        21.79509        YES      YES
```

51	a	279.97	9.57097	YES	YES
52	a	283.52	3.14637	YES	YES
53	a	292.04	16.94779	YES	YES
54	a	297.83	17.54200	YES	YES
55	a	303.82	39.93458	YES	YES
56	a	317.57	65.11918	YES	YES
57	a	353.40	67.46297	YES	YES
58	a	362.14	11.39314	YES	YES
59	a	364.78	1.55367	YES	YES
60	a	366.03	0.73502	YES	YES
61	a	375.02	11.18757	YES	YES
62	a	395.04	12.26621	YES	YES
63	a	395.28	0.78632	YES	YES
64	a	425.08	0.08688	YES	YES
65	a	430.09	2.30445	YES	YES
66	a	432.78	3.27213	YES	YES
67	a	434.40	2.54915	YES	YES
68	a	478.63	11.35434	YES	YES
69	a	479.73	0.04040	YES	YES
70	a	521.49	88.27692	YES	YES
71	a	526.01	28.94582	YES	YES
72	a	526.43	40.87764	YES	YES
73	a	527.24	0.58554	YES	YES
74	a	529.04	0.42878	YES	YES
75	a	559.29	2.51793	YES	YES
76	a	576.02	47.96966	YES	YES
77	a	579.43	10.90519	YES	YES
78	a	580.42	4.90451	YES	YES
79	a	582.44	10.32491	YES	YES
80	a	583.56	9.52224	YES	YES
81	a	589.11	2.25986	YES	YES
82	a	592.30	1.61038	YES	YES
83	a	598.44	9.92643	YES	YES
84	a	600.37	1.97834	YES	YES
85	a	727.93	31.71850	YES	YES
86	a	730.41	10.30811	YES	YES
87	a	732.16	13.97728	YES	YES
88	a	733.64	13.78625	YES	YES
89	a	749.71	19.73304	YES	YES
90	a	757.63	73.19228	YES	YES
91	a	784.57	60.82504	YES	YES
92	a	786.22	45.69061	YES	YES
93	a	827.58	14.25492	YES	YES
94	a	827.89	39.43149	YES	YES
95	a	875.73	19.37011	YES	YES
96	a	877.33	17.41212	YES	YES
97	a	914.11	76.01980	YES	YES
98	a	924.88	87.74286	YES	YES
99	a	936.91	34.38632	YES	YES
100	a	938.22	7.62253	YES	YES
101	a	940.83	48.27484	YES	YES
102	a	942.76	66.82165	YES	YES
103	a	945.44	37.42891	YES	YES
104	a	950.72	72.22414	YES	YES
105	a	998.31	186.23612	YES	YES
106	a	1021.13	240.72712	YES	YES
107	a	1024.37	55.02937	YES	YES
108	a	1041.41	59.04719	YES	YES
109	a	1051.16	7.87878	YES	YES
110	a	1053.36	14.32655	YES	YES
111	a	1077.78	135.48731	YES	YES
112	a	1101.58	262.11434	YES	YES
113	a	1102.88	34.20789	YES	YES

114	a	1104.71	79.46169	YES	YES
115	a	1109.23	145.53583	YES	YES
116	a	1128.65	126.92509	YES	YES
117	a	1134.25	49.14541	YES	YES
118	a	1136.21	118.65250	YES	YES
119	a	1149.11	185.50923	YES	YES
120	a	1165.59	104.06579	YES	YES
121	a	1167.14	442.79220	YES	YES
122	a	1181.97	268.99744	YES	YES
123	a	1191.29	111.37713	YES	YES
124	a	1194.42	242.76095	YES	YES
125	a	1199.69	343.47557	YES	YES
126	a	1205.33	177.59025	YES	YES
127	a	1206.49	126.80322	YES	YES
128	a	1212.22	233.76018	YES	YES
129	a	1247.11	46.14168	YES	YES
130	a	1249.72	39.14481	YES	YES
131	a	1284.48	375.01923	YES	YES
132	a	1292.91	134.59466	YES	YES
133	a	1295.61	137.78226	YES	YES
134	a	1310.38	77.88022	YES	YES
135	a	1366.42	14.34960	YES	YES
136	a	1370.38	14.57285	YES	YES
137	a	1391.76	39.95312	YES	YES
138	a	1392.41	50.56157	YES	YES
139	a	1425.96	34.90182	YES	YES
140	a	1429.49	40.30647	YES	YES
141	a	1502.62	20.75878	YES	YES
142	a	1503.59	13.20074	YES	YES
143	a	1837.31	1400.49099	YES	YES
144	a	1857.13	103.62952	YES	YES
145	a	3095.51	6.32483	YES	YES
146	a	3101.95	6.41398	YES	YES
147	a	3150.39	4.79481	YES	YES
148	a	3155.74	3.89089	YES	YES
149	a	3163.83	1.79305	YES	YES
150	a	3167.73	1.24329	YES	YES

$end

Total COSMO energy + OC corr. = -5117.6590128355 H

EC

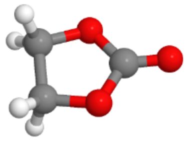

```
Method: (RI-)B3LYP(D3BJ)/def2-TZVPP
Symmetry: c2

Cartesian coordinates in Ångström:
O     1.0942878    0.2086586    0.7300756
C     0.0000000    0.0000000    1.5076646
O    -0.0000000    0.0000000    2.6944008
O    -1.0942878   -0.2086586    0.7300756
C    -0.6997783   -0.3083198   -0.6442472
C     0.6997783    0.3083198   -0.6442472
H    -1.4156884    0.2361874   -1.2544133
H    -0.6955456   -1.3601918   -0.9324478
H     0.6955456    1.3601918   -0.9324478
H     1.4156884   -0.2361874   -1.2544133

SCF energy GEOOPT =  -342.3875432189 H
ZPE =   196.5 kJ/mol
FREEH energy =   209.24 kJ/mol
FREEH entropy =   0.29666 kJ/mol/K

$vibrational spectrum
#  mode     symmetry     wave number   IR intensity    selection rules
#                         cm**(-1)        km/mol         IR      RAMAN
     1                      -0.00         0.00000        -        -
     2                      -0.00         0.00000        -        -
     3                       0.00         0.00000        -        -
     4                       0.00         0.00000        -        -
     5                       0.00         0.00000        -        -
     6                       0.00         0.00000        -        -
     7         a           148.70         0.23091       YES      YES
     8         b           188.27         1.27916       YES      YES
     9         b           522.62         0.17616       YES      YES
    10         b           701.46         0.62670       YES      YES
    11         a           723.42         4.82535       YES      YES
    12         b           773.21        21.54855       YES      YES
    13         b           886.44         0.71392       YES      YES
    14         a           892.47         3.58489       YES      YES
    15         a           968.15        21.25375       YES      YES
    16         b          1056.66         8.97581       YES      YES
    17         a          1094.61       209.86276       YES      YES
    18         b          1126.12       262.78567       YES      YES
    19         a          1158.56         2.93509       YES      YES
    20         b          1241.26         6.58663       YES      YES
    21         a          1249.77        12.28588       YES      YES
    22         a          1389.31         0.26335       YES      YES
    23         b          1408.16        33.09803       YES      YES
    24         b          1524.64         7.35752       YES      YES
    25         a          1532.88         0.42648       YES      YES
    26         a          1896.52       640.10021       YES      YES
    27         a          3054.34        29.38159       YES      YES
    28         b          3058.05        31.73372       YES      YES
    29         a          3117.91        12.30918       YES      YES
    30         b          3131.18        21.25936       YES      YES
$end
Total COSMO energy + OC corr. = -342.4026119150 H
```

EMC

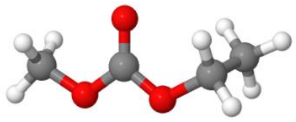

```
Method: (RI-)B3LYP(D3BJ)/def2-TZVPP
Symmetry: c1

Cartesian coordinates in Ångström:
H     2.6355552   -0.4075011   -1.0011931
H     2.1165944    0.8284717    0.1620104
C     2.4095772   -0.2141302    0.0475966
H    -2.2617483    1.9797707   -0.7021917
H     3.3174016   -0.3792985    0.6303562
O    -0.5557597    0.7768363    0.9475224
C    -2.7099168    0.9922431   -0.6063409
C    -0.7007109   -0.0112689    0.0465733
O    -1.7245606   -0.0299499   -0.8135111
H    -3.4554216    0.8350590   -1.3796085
C     1.3138155   -1.1366873    0.5398401
O     0.1194436   -1.0162090   -0.2674838
H    -3.1575975    0.8966670    0.3814034
H     1.5957194   -2.1824076    0.4372946
H     1.0576082   -0.9315952    1.5777321

SCF energy GEOOPT =  -382.9037589102 H
ZPE =   324.4 kJ/mol
FREEH energy =   345.68 kJ/mol
FREEH entropy =   0.36890 kJ/mol/K

$vibrational spectrum
#  mode     symmetry     wave number   IR intensity    selection rules
#                         cm**(-1)        km/mol         IR     RAMAN
     1                     -0.00          0.00000         -       -
     2                      0.00          0.00000         -       -
     3                      0.00          0.00000         -       -
     4                      0.00          0.00000         -       -
     5                      0.00          0.00000         -       -
     6                      0.00          0.00000         -       -
     7        a            81.43          1.88375        YES     YES
     8        a            87.45          3.16836        YES     YES
     9        a           128.52          0.52472        YES     YES
    10        a           174.77          2.15654        YES     YES
    11        a           204.14          3.08386        YES     YES
    12        a           268.43          5.19067        YES     YES
    13        a           354.88         21.65991        YES     YES
    14        a           401.36          5.78692        YES     YES
    15        a           532.85          1.01350        YES     YES
    16        a           691.31          0.36133        YES     YES
    17        a           803.34         23.38840        YES     YES
    18        a           812.96          4.11926        YES     YES
    19        a           887.52         16.36305        YES     YES
    20        a           943.66         23.28745        YES     YES
    21        a          1021.30         90.14685        YES     YES
    22        a          1110.99         17.24455        YES     YES
    23        a          1141.52         13.22917        YES     YES
    24        a          1180.80          1.05403        YES     YES
    25        a          1198.81         11.94833        YES     YES
    26        a          1224.54          3.70072        YES     YES
    27        a          1294.92        821.13050        YES     YES
```

28	a	1335.65	38.72918	YES	YES
29	a	1405.24	27.63653	YES	YES
30	a	1423.37	11.35353	YES	YES
31	a	1479.29	36.49507	YES	YES
32	a	1487.08	8.18782	YES	YES
33	a	1487.23	7.65278	YES	YES
34	a	1495.90	18.45736	YES	YES
35	a	1502.06	5.62490	YES	YES
36	a	1509.17	19.61812	YES	YES
37	a	1784.52	292.41278	YES	YES
38	a	3039.15	15.11873	YES	YES
39	a	3052.32	31.86249	YES	YES
40	a	3072.34	21.13946	YES	YES
41	a	3101.62	23.12077	YES	YES
42	a	3109.99	6.97649	YES	YES
43	a	3122.57	19.63730	YES	YES
44	a	3132.39	24.30331	YES	YES
45	a	3156.16	14.28583	YES	YES

$end

Total COSMO energy + OC corr. = -382.9129648512 H

$[PF_6]^-$

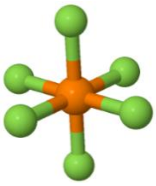

Method: (RI-)B3LYP(D3BJ)/def2-TZVPP
Symmetry: oh

Cartesian coordinates in Ångström:

P	-0.0000000	-0.0000000	-0.0000000
F	-1.6278198	0.0000000	0.0000000
F	1.6278198	-0.0000000	-0.0000000
F	-0.0000000	0.0000000	-1.6278198
F	0.0000000	-0.0000000	1.6278198
F	-0.0000000	-1.6278198	-0.0000000
F	0.0000000	1.6278198	0.0000000

SCF energy GEOOPT = -940.7619530980 H
ZPE = 49.28 kJ/mol
FREEH energy = 65.42 kJ/mol
FREEH entropy = 0.30212 kJ/mol/K

$vibrational spectrum

# mode	symmetry	wave number	IR intensity	selection rules	
#		cm**(-1)	km/mol	IR	RAMAN
1		-0.00	0.00000	-	-
2		-0.00	0.00000	-	-
3		-0.00	0.00000	-	-
4		-0.00	0.00000	-	-
5		-0.00	0.00000	-	-
6		0.00	0.00000	-	-
7	t2u	296.15	0.00000	NO	NO
8	t2u	296.15	0.00000	NO	NO
9	t2u	296.15	0.00000	NO	NO
10	t2g	454.94	0.00000	NO	YES

```
    11         t2g            454.94           0.00000        NO       YES
    12         t2g            454.94           0.00000        NO       YES
    13         t1u            545.03          25.43844        YES      NO
    14         t1u            545.03          25.43844        YES      NO
    15         t1u            545.03          25.43844        YES      NO
    16         eg             551.26           0.00000        NO       YES
    17         eg             551.26           0.00000        NO       YES
    18         a1g            714.94           0.00000        NO       YES
    19         t1u            844.24         457.81888        YES      NO
    20         t1u            844.24         457.81888        YES      NO
    21         t1u            844.24         457.81888        YES      NO
$end
```

```
Total COSMO energy + OC corr. = -940.8455398240 H
```

Li[PF$_6$]

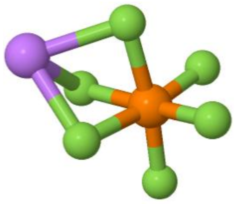

```
Method: (RI-)B3LYP(D3BJ)/def2-TZVPP
Symmetry: c3v

Cartesian coordinates in Ångström:
P    -0.0000000    0.0000000    0.3972793
F     0.6532931   -1.1315368   -0.6805369
F    -0.6681231    1.1572232    1.2283699
F    -0.6681231   -1.1572232    1.2283699
F     0.6532931    1.1315368   -0.6805369
F    -1.3065861    0.0000000   -0.6805369
F     1.3362462    0.0000000    1.2283699
Li   -0.0000000    0.0000000   -2.0413156

SCF energy GEOOPT =  -948.2661934722 H
ZPE =   55.80 kJ/mol
FREEH energy =    75.22 kJ/mol
FREEH entropy =   0.34022 kJ/mol/K

$vibrational spectrum
#  mode     symmetry     wave number   IR intensity    selection rules
#                         cm**(-1)       km/mol         IR     RAMAN
     1                      -0.00        0.00000        -       -
     2                      -0.00        0.00000        -       -
     3                      -0.00        0.00000        -       -
     4                       0.00        0.00000        -       -
     5                       0.00        0.00000        -       -
     6                       0.00        0.00000        -       -
     7         e            210.49        21.18592       YES      YES
     8         e            210.49        21.18592       YES      YES
     9         a2           276.00         0.00000       NO       NO
    10         e            326.59         0.01602       YES      YES
    11         e            326.59         0.01602       YES      YES
    12         a1           445.22        43.94177       YES      YES
    13         e            449.90         7.15238       YES      YES
    14         e            449.90         7.15238       YES      YES
```

15	e	513.60	8.33981	YES	YES
16	e	513.60	8.33981	YES	YES
17	e	548.89	99.36346	YES	YES
18	e	548.89	99.36346	YES	YES
19	a1	553.60	183.02848	YES	YES
20	a1	579.07	84.58832	YES	YES
21	a1	677.29	59.08369	YES	YES
22	a1	898.64	295.46889	YES	YES
23	e	900.40	388.92484	YES	YES
24	e	900.40	388.92484	YES	YES

$end

Total COSMO energy + OC corr. = -948.2994140008 H

[Li(EC)$_4$]$^+$

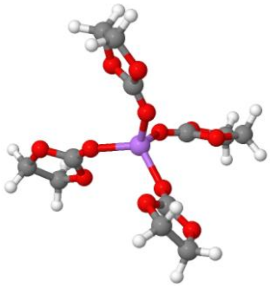

Method: (RI-)B3LYP(D3BJ)/def2-TZVPP
Symmetry: c1

Cartesian coordinates in Ångström:

O	0.4324895	0.3732519	0.5998022
C	-0.6613364	-0.1811243	1.1340744
O	-0.9186469	-0.1777703	2.3086105
O	-1.4424953	-0.7387459	0.2038481
C	-0.7800543	-0.6922450	-1.0785770
C	0.3611276	0.3017426	-0.8404984
H	-1.4985754	-0.3635838	-1.8232886
H	-0.4305644	-1.6954591	-1.3159470
H	0.1462580	1.2992297	-1.2197824
H	1.3220544	-0.0387216	-1.2141802
H	-0.6516616	4.6393641	7.2142851
H	-2.8168948	3.8591673	6.4856810
O	-1.6644056	2.8097616	5.1357405
C	-0.8207006	4.7323054	6.1429757
C	-0.3373459	2.8435438	4.9724179
C	-2.1205585	4.0673897	5.6790480
O	0.3038456	1.9722215	4.4476513
O	0.1963475	3.9680655	5.4594064
H	-0.7286835	5.7712897	5.8414205
H	-2.6234001	4.6175585	4.8856609
O	-0.9977525	-0.6884869	5.3603937
H	-3.4263246	-3.6052012	4.2685391
C	-1.6708056	-1.6766566	5.4865595
O	-1.5993605	-2.7459669	4.6850938
O	1.8071801	-0.6203253	3.7008027
O	-2.5803699	-1.8388030	6.4524002
C	-2.6489759	-3.6769184	5.0273509
H	-4.1828910	-3.1355556	6.5167102

```
C    -3.1042203   -3.1840027    6.4033984
C     2.8865282   -0.4910074    4.2143634
H    -2.2295441   -4.6782889    5.0373875
O     4.0359608   -0.8403175    3.6282789
O     3.0846092    0.0130919    5.4380802
H    -2.6682160   -3.7482335    7.2260901
H     4.7452999    1.2254703    5.5857293
C     4.5010047    0.1681384    5.6729542
C     5.1238656   -0.6864498    4.5660000
H     5.9515002   -0.2051226    4.0540701
H     5.4215703   -1.6768989    4.9060979
H     4.7230826   -0.1839429    6.6756905
Li    0.0453297    0.1243663    3.9556606

SCF energy GEOOPT =  -1377.042818447 H
ZPE =   803.5 kJ/mol
FREEH energy =   874.18 kJ/mol
FREEH entropy =   0.89280 kJ/mol/K

$vibrational spectrum
#  mode     symmetry     wave number   IR intensity    selection rules
#                         cm**(-1)        km/mol         IR     RAMAN
     1                       -0.00         0.00000        -       -
     2                       -0.00         0.00000        -       -
     3                       -0.00         0.00000        -       -
     4                        0.00         0.00000        -       -
     5                        0.00         0.00000        -       -
     6                        0.00         0.00000        -       -
     7         a              8.38         0.01522       YES     YES
     8         a             10.78         0.01415       YES     YES
     9         a             12.07         3.41300       YES     YES
    10         a             12.79         3.59575       YES     YES
    11         a             13.72         0.06042       YES     YES
    12         a             15.99         6.78298       YES     YES
    13         a             19.41         0.59068       YES     YES
    14         a             21.19         0.53717       YES     YES
    15         a             24.90         1.00234       YES     YES
    16         a             33.75         0.04942       YES     YES
    17         a             38.72         2.48150       YES     YES
    18         a             41.33         2.55783       YES     YES
    19         a             70.80         7.14892       YES     YES
    20         a             79.96         0.04337       YES     YES
    21         a             97.44         7.79057       YES     YES
    22         a             97.82        10.79163       YES     YES
    23         a             98.57         9.13897       YES     YES
    24         a            113.85         0.21151       YES     YES
    25         a            114.23         0.13655       YES     YES
    26         a            131.29         0.24354       YES     YES
    27         a            132.19         0.27611       YES     YES
    28         a            143.87         0.00378       YES     YES
    29         a            210.48         5.21255       YES     YES
    30         a            211.38         0.13244       YES     YES
    31         a            213.51         0.06608       YES     YES
    32         a            216.51         0.16120       YES     YES
    33         a            417.42       148.91673       YES     YES
    34         a            437.18       133.42731       YES     YES
    35         a            439.50       134.33840       YES     YES
    36         a            535.88         0.14963       YES     YES
    37         a            537.76         8.17107       YES     YES
    38         a            543.33        32.92594       YES     YES
    39         a            544.00        33.22838       YES     YES
    40         a            715.40         2.06878       YES     YES
    41         a            715.51         2.58549       YES     YES
```

42	a	720.78	2.06485	YES	YES
43	a	720.87	2.58584	YES	YES
44	a	740.62	1.08219	YES	YES
45	a	742.10	33.15622	YES	YES
46	a	742.83	56.33190	YES	YES
47	a	743.32	57.26420	YES	YES
48	a	783.39	7.29393	YES	YES
49	a	784.45	8.76126	YES	YES
50	a	784.73	7.50921	YES	YES
51	a	786.36	83.90333	YES	YES
52	a	877.79	1.23865	YES	YES
53	a	877.85	1.20503	YES	YES
54	a	880.96	1.01705	YES	YES
55	a	881.14	1.04782	YES	YES
56	a	914.27	1.93471	YES	YES
57	a	914.66	14.70807	YES	YES
58	a	914.92	6.86646	YES	YES
59	a	915.20	14.27559	YES	YES
60	a	977.28	23.15365	YES	YES
61	a	977.41	12.95627	YES	YES
62	a	978.48	23.87664	YES	YES
63	a	979.07	2.41659	YES	YES
64	a	1037.45	1.89186	YES	YES
65	a	1037.57	6.22792	YES	YES
66	a	1038.30	4.20113	YES	YES
67	a	1038.53	5.76113	YES	YES
68	a	1099.81	314.94251	YES	YES
69	a	1100.03	200.07650	YES	YES
70	a	1101.36	328.16727	YES	YES
71	a	1106.00	3.68683	YES	YES
72	a	1155.72	1.63650	YES	YES
73	a	1155.73	1.24276	YES	YES
74	a	1155.75	1.02584	YES	YES
75	a	1155.77	0.37737	YES	YES
76	a	1190.38	3.95855	YES	YES
77	a	1193.30	457.41106	YES	YES
78	a	1194.49	6.58659	YES	YES
79	a	1195.14	461.71145	YES	YES
80	a	1241.04	7.38458	YES	YES
81	a	1241.18	36.25084	YES	YES
82	a	1241.27	0.94965	YES	YES
83	a	1241.30	12.89628	YES	YES
84	a	1251.08	16.20794	YES	YES
85	a	1251.10	16.23209	YES	YES
86	a	1251.26	20.61396	YES	YES
87	a	1251.60	0.51619	YES	YES
88	a	1392.46	0.09483	YES	YES
89	a	1392.50	0.04176	YES	YES
90	a	1392.67	0.12625	YES	YES
91	a	1392.82	0.00045	YES	YES
92	a	1428.19	0.57584	YES	YES
93	a	1429.03	119.04213	YES	YES
94	a	1430.07	1.28085	YES	YES
95	a	1430.43	120.21560	YES	YES
96	a	1525.61	9.36484	YES	YES
97	a	1525.67	17.73871	YES	YES
98	a	1526.31	3.35069	YES	YES
99	a	1526.36	24.32803	YES	YES
100	a	1533.35	0.83582	YES	YES
101	a	1533.66	0.47356	YES	YES
102	a	1534.38	0.80557	YES	YES
103	a	1534.66	0.32678	YES	YES
104	a	1832.07	912.53263	YES	YES

105	a	1833.19	1376.48329	YES	YES
106	a	1833.93	1377.80760	YES	YES
107	a	1867.58	0.04951	YES	YES
108	a	3079.01	29.17195	YES	YES
109	a	3079.11	14.61319	YES	YES
110	a	3080.02	27.95010	YES	YES
111	a	3080.12	13.69898	YES	YES
112	a	3081.54	14.13268	YES	YES
113	a	3081.59	13.27127	YES	YES
114	a	3082.09	17.01072	YES	YES
115	a	3082.16	12.87857	YES	YES
116	a	3137.66	4.19900	YES	YES
117	a	3137.79	3.70531	YES	YES
118	a	3138.97	5.31875	YES	YES
119	a	3138.99	3.25242	YES	YES
120	a	3152.67	7.40183	YES	YES
121	a	3152.77	9.05567	YES	YES
122	a	3153.37	4.32016	YES	YES
123	a	3153.39	12.37575	YES	YES

$end

Total COSMO energy + OC corr. = -1377.1138637853 H

$[Li(EC)_5]^+$

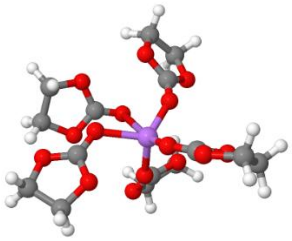

Method: (RI-)B3LYP(D3BJ)/def2-TZVPP
Symmetry: c1

Cartesian coordinates in Ångström:

O	-0.0090202	1.6965899	0.6241381
C	-0.3724281	0.6066265	1.3204139
O	-1.1523296	0.6273155	2.2362485
O	0.2369985	-0.4904879	0.8697410
C	1.2325602	-0.1270526	-0.1099836
C	0.8263481	1.2984452	-0.4883677
H	1.1759783	-0.8319798	-0.9334578
H	2.2102869	-0.1813285	0.3656187
H	0.2274119	1.3464674	-1.3961001
H	1.6614611	1.9890663	-0.5544511
H	0.2317501	5.0460527	3.2250796
H	-2.1860170	4.9277860	3.0605658
O	-1.8439339	3.4801036	4.4900828
C	-0.1633140	4.0552694	3.0039769
C	-0.6989508	2.9039525	4.8910139
C	-1.6813920	3.9699876	3.1412994
O	-0.5852568	2.2601926	5.8975158
O	0.2985273	3.1471514	4.0280924
H	0.2015569	3.7033044	2.0456894
H	-2.1223977	3.2419819	2.4634697
O	-1.6053106	-2.5477670	2.6514997
H	-3.4945796	-6.0210223	2.6923272

```
C    -1.7782114   -3.7115845    2.8980333
O    -2.1620254   -4.6136375    1.9899607
O     0.6675344   -0.6312587    4.1255716
O    -1.6090479   -4.2604289    4.1061073
C    -2.4166999   -5.8778240    2.6402041
H    -2.3890504   -6.0224016    4.8409677
C    -1.7666636   -5.6929627    4.0145760
C     1.1013888   -0.2027845    5.1670520
H    -1.9658905   -6.6659027    2.0447858
O     2.1233935    0.6488222    5.2436269
O     0.6294007   -0.5366160    6.3656466
H    -0.7803841   -6.1481808    4.0864432
H     0.5440119    0.8544148    7.8722314
C     1.2899420    0.2289869    7.3918993
C     2.3379678    1.0425382    6.6147341
H     2.1738597    2.1138524    6.6836322
H     3.3633817    0.7974646    6.8796849
H     1.7216432   -0.4669283    8.1065177
Li   -1.1730299   -0.9158175    3.5457300
H    -3.7818508    2.9979853    6.3475489
C    -3.9964978    1.9549064    6.5581298
O    -3.5939216    1.1783182    5.4123496
H    -5.0646514    1.8100934    6.7059626
C    -2.7761837    0.1944875    5.7890241
O    -2.3020128   -0.6079244    5.0282433
C    -3.1395094    1.3775380    7.6928204
H    -2.3227812    2.0366759    7.9749127
O    -2.5664475    0.1905132    7.1059989
H    -3.7130436    1.0817707    8.5664521

SCF energy GEOOPT =  -1719.456350364 H
ZPE =   1005. kJ/mol
FREEH energy =  1092.78 kJ/mol
FREEH entropy =   1.00184 kJ/mol/K

$vibrational spectrum
#  mode     symmetry     wave number   IR intensity    selection rules
#                         cm**(-1)        km/mol          IR     RAMAN
     1                      -0.00         0.00000         -        -
     2                      -0.00         0.00000         -        -
     3                       0.00         0.00000         -        -
     4                       0.00         0.00000         -        -
     5                       0.00         0.00000         -        -
     6                       0.00         0.00000         -        -
     7         a             7.77         0.69678        YES      YES
     8         a            12.26         2.04899        YES      YES
     9         a            14.34         5.94322        YES      YES
    10         a            15.85         4.27544        YES      YES
    11         a            18.27         1.03258        YES      YES
    12         a            25.27         1.03961        YES      YES
    13         a            27.57         1.49734        YES      YES
    14         a            33.24         0.34152        YES      YES
    15         a            38.22         2.65456        YES      YES
    16         a            40.52         4.10644        YES      YES
    17         a            43.85         0.93172        YES      YES
    18         a            50.34         1.87047        YES      YES
    19         a            56.63         1.83719        YES      YES
    20         a            62.17         1.01159        YES      YES
    21         a            64.32         2.98633        YES      YES
    22         a            72.25         1.69972        YES      YES
    23         a            75.27         0.73289        YES      YES
    24         a            77.89         1.52873        YES      YES
    25         a            84.62         2.18897        YES      YES
```

26	a	89.88	7.59322	YES	YES
27	a	92.84	14.05671	YES	YES
28	a	97.90	9.97509	YES	YES
29	a	105.81	0.55539	YES	YES
30	a	114.05	7.99205	YES	YES
31	a	118.01	0.39182	YES	YES
32	a	122.06	0.09387	YES	YES
33	a	146.29	0.21135	YES	YES
34	a	150.40	2.03380	YES	YES
35	a	185.03	0.26136	YES	YES
36	a	211.04	2.05844	YES	YES
37	a	213.88	3.03059	YES	YES
38	a	216.74	6.58852	YES	YES
39	a	227.46	6.32184	YES	YES
40	a	243.04	3.36717	YES	YES
41	a	348.66	89.70429	YES	YES
42	a	443.76	134.46379	YES	YES
43	a	468.07	152.47112	YES	YES
44	a	522.82	2.52854	YES	YES
45	a	529.43	0.91700	YES	YES
46	a	536.97	10.89878	YES	YES
47	a	540.31	11.51052	YES	YES
48	a	546.15	43.10683	YES	YES
49	a	703.32	2.41401	YES	YES
50	a	711.79	2.79195	YES	YES
51	a	718.36	2.21033	YES	YES
52	a	730.97	10.58659	YES	YES
53	a	732.90	22.80511	YES	YES
54	a	739.31	2.02389	YES	YES
55	a	742.29	14.83750	YES	YES
56	a	745.35	42.62905	YES	YES
57	a	750.26	54.00549	YES	YES
58	a	758.58	4.71163	YES	YES
59	a	780.66	45.47692	YES	YES
60	a	782.14	35.18702	YES	YES
61	a	783.42	26.41807	YES	YES
62	a	784.37	63.06801	YES	YES
63	a	789.28	12.18512	YES	YES
64	a	853.45	0.16662	YES	YES
65	a	869.15	0.46120	YES	YES
66	a	879.12	1.18372	YES	YES
67	a	884.51	1.27581	YES	YES
68	a	891.97	0.50633	YES	YES
69	a	912.32	2.30578	YES	YES
70	a	913.66	8.18213	YES	YES
71	a	915.27	10.08072	YES	YES
72	a	916.80	10.16266	YES	YES
73	a	918.31	17.67505	YES	YES
74	a	976.10	7.26841	YES	YES
75	a	977.10	29.84980	YES	YES
76	a	978.19	14.35512	YES	YES
77	a	984.69	5.85451	YES	YES
78	a	986.98	11.48248	YES	YES
79	a	1035.64	7.36648	YES	YES
80	a	1037.14	1.71950	YES	YES
81	a	1038.01	5.29037	YES	YES
82	a	1042.76	1.95943	YES	YES
83	a	1045.59	2.78090	YES	YES
84	a	1091.42	119.99822	YES	YES
85	a	1095.02	301.10678	YES	YES
86	a	1102.89	239.56582	YES	YES
87	a	1109.00	68.70642	YES	YES
88	a	1114.39	209.06898	YES	YES

89	a	1154.52	0.07362	YES	YES
90	a	1155.39	1.32591	YES	YES
91	a	1155.80	1.23621	YES	YES
92	a	1155.94	0.60063	YES	YES
93	a	1159.38	0.72665	YES	YES
94	a	1173.87	128.36623	YES	YES
95	a	1191.43	206.96633	YES	YES
96	a	1194.19	215.68502	YES	YES
97	a	1202.91	253.21762	YES	YES
98	a	1214.15	261.47360	YES	YES
99	a	1241.22	14.51728	YES	YES
100	a	1241.68	19.76601	YES	YES
101	a	1244.23	0.70280	YES	YES
102	a	1245.07	9.45167	YES	YES
103	a	1246.84	15.77536	YES	YES
104	a	1251.10	13.86159	YES	YES
105	a	1252.41	15.21181	YES	YES
106	a	1252.80	1.51576	YES	YES
107	a	1256.83	11.03850	YES	YES
108	a	1257.42	12.74920	YES	YES
109	a	1392.59	0.00561	YES	YES
110	a	1392.70	1.22152	YES	YES
111	a	1393.27	0.08378	YES	YES
112	a	1395.25	0.98858	YES	YES
113	a	1396.21	0.36509	YES	YES
114	a	1421.85	27.71109	YES	YES
115	a	1427.12	55.98764	YES	YES
116	a	1429.77	56.28533	YES	YES
117	a	1436.77	53.20981	YES	YES
118	a	1440.06	70.67988	YES	YES
119	a	1518.46	17.30066	YES	YES
120	a	1519.28	13.51373	YES	YES
121	a	1520.94	20.62419	YES	YES
122	a	1523.73	13.45173	YES	YES
123	a	1526.12	13.12899	YES	YES
124	a	1527.22	6.52990	YES	YES
125	a	1529.39	1.41754	YES	YES
126	a	1531.69	0.76501	YES	YES
127	a	1532.37	0.81379	YES	YES
128	a	1534.15	0.61122	YES	YES
129	a	1810.38	456.50430	YES	YES
130	a	1827.66	1238.11513	YES	YES
131	a	1833.47	1380.06165	YES	YES
132	a	1839.58	623.17655	YES	YES
133	a	1863.80	300.43869	YES	YES
134	a	3074.36	26.46012	YES	YES
135	a	3079.63	22.17378	YES	YES
136	a	3080.47	21.41947	YES	YES
137	a	3081.98	14.27268	YES	YES
138	a	3083.32	11.94363	YES	YES
139	a	3084.78	21.42283	YES	YES
140	a	3088.08	14.14742	YES	YES
141	a	3089.86	14.75120	YES	YES
142	a	3092.70	19.03640	YES	YES
143	a	3094.98	33.22590	YES	YES
144	a	3137.98	0.50250	YES	YES
145	a	3138.33	4.21795	YES	YES
146	a	3139.08	2.53168	YES	YES
147	a	3141.47	4.06570	YES	YES
148	a	3149.11	7.72562	YES	YES
149	a	3153.05	8.07227	YES	YES
150	a	3155.91	6.34960	YES	YES
151	a	3156.14	11.00401	YES	YES

```
   152         a            3157.17        13.41200        YES      YES
   153         a            3166.59         1.77831        YES      YES
$end

Total COSMO energy + OC corr. = -1719.5262160345 H
```

[Li(EMC)$_4$]$^+$

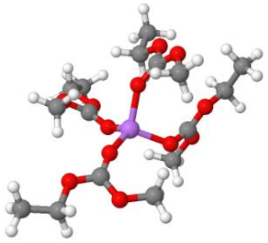

```
Method: (RI-)B3LYP(D3BJ)/def2-TZVPP
Symmetry: c1

Cartesian coordinates in Ångström:
H     0.5666372   -0.0127918   -3.0818110
H     1.4022894   -1.5218746   -2.6760061
C     0.4268467   -1.0456251   -2.7688644
H    -1.7843261    0.1886063    2.6195556
H    -0.1349609   -1.5693243   -3.5427244
O     0.6860682    0.2624304    1.6300536
C    -1.6351247   -0.8566291    2.3622696
C     0.0421836   -0.3407556    0.7893481
O    -1.1061979   -0.9552441    1.0164023
H    -2.5767645   -1.3933743    2.3329496
C    -0.3348996   -1.1214167   -1.4700957
O     0.4563021   -0.4139818   -0.4620686
H    -0.9473271   -1.3242868    3.0622027
H    -1.3077476   -0.6363714   -1.5347873
H    -0.4772855   -2.1458206   -1.1320191
H    -3.6103545    2.8596905   -1.6754535
C    -2.6684169    2.6262014   -2.1723959
H    -2.7329855    1.6120232   -2.5652668
H    -2.5394087    3.3133571   -3.0073116
C    -1.5371628    2.7539012   -1.1832124
H    -1.6488495    2.0687333   -0.3440758
H    -1.4501564    3.7670555   -0.7903177
O    -0.3066588    2.4303379   -1.8814456
C     0.8086300    2.4815655   -1.1741160
O     0.8808284    2.7999123    0.0005543
O     1.8301730    2.1470298   -1.9357600
C     3.1104764    2.0551200   -1.2785367
H     3.0784071    1.2697167   -0.5262522
H     3.3673814    3.0061387   -0.8181181
H     3.8151670    1.8057254   -2.0638749
O    -1.0567453    2.6596390    2.2994978
H     0.0502515    4.7928851    3.3695544
C    -1.9023732    3.5208126    2.1459992
H    -5.4046083    2.9454822    0.5130088
C    -0.3227440    5.2496705    2.4576007
H     0.3117431    4.9730768    1.6189606
H    -5.7571167    2.8701930    2.2459882
C    -5.4740436    3.5322246    1.4287113
O    -3.1553669    3.1968336    1.8890179
O    -1.6831635    4.8252076    2.2016871
```

```
H    -0.3781628    6.3279195    2.5568008
H    -6.2621801    4.2742309    1.3000363
C    -4.1722427    4.2332275    1.7255046
H    -3.8640171    4.8906693    0.9140082
H    -4.2183456    4.8142861    2.6448060
O     2.0280530    2.8354039    3.0329242
H     2.4901194    0.2916628    3.6093984
C     2.7321091    2.8934345    4.0237911
H     3.7447491    6.1941075    5.8476420
C     3.4982450    0.6917064    3.6763254
H     3.8799785    0.8911511    2.6777501
H     2.4214219    5.5747852    6.8498770
C     3.4561799    5.4129373    6.5499160
O     2.7761297    3.9817876    4.7616812
O     3.5053393    1.9089661    4.4559604
H     4.1537261    0.0112817    4.2083293
H     4.0861155    5.4991463    7.4354911
C     3.6371434    4.0446170    5.9428939
H     4.6630046    3.8676747    5.6248035
H     3.3390738    3.2490426    6.6234832
Li    0.7581835    2.1943181    1.8255492

SCF energy GEOOPT =  -1539.102809130 H
ZPE =    1314. kJ/mol
FREEH energy =  1417.85 kJ/mol
FREEH entropy =   1.09370 kJ/mol/K

$vibrational spectrum
#  mode     symmetry     wave number   IR intensity    selection rules
#                         cm**(-1)        km/mol         IR     RAMAN
     1                      -0.00         0.00000        -        -
     2                       0.00         0.00000        -        -
     3                       0.00         0.00000        -        -
     4                       0.00         0.00000        -        -
     5                       0.00         0.00000        -        -
     6                       0.00         0.00000        -        -
     7         a             5.88         0.21674       YES      YES
     8         a             7.94         0.04567       YES      YES
     9         a            10.09         0.55680       YES      YES
    10         a            18.65         0.35668       YES      YES
    11         a            20.51         0.39888       YES      YES
    12         a            25.11         1.04202       YES      YES
    13         a            28.58         1.16056       YES      YES
    14         a            30.59         1.31436       YES      YES
    15         a            36.57         1.10933       YES      YES
    16         a            47.23         0.36724       YES      YES
    17         a            55.72         3.01117       YES      YES
    18         a            61.02         0.06981       YES      YES
    19         a            63.89         0.41988       YES      YES
    20         a            65.49         0.54548       YES      YES
    21         a            68.62         0.30631       YES      YES
    22         a            71.11         1.68924       YES      YES
    23         a            75.11         0.28222       YES      YES
    24         a            78.83         1.71818       YES      YES
    25         a            89.09         3.11695       YES      YES
    26         a           101.00         4.33581       YES      YES
    27         a           102.63         2.05206       YES      YES
    28         a           116.78         7.58466       YES      YES
    29         a           125.12         1.06454       YES      YES
    30         a           136.46         0.67570       YES      YES
    31         a           141.97         0.28108       YES      YES
    32         a           143.74         1.07212       YES      YES
    33         a           151.54         0.49833       YES      YES
```

34	a	155.07	0.93591	YES	YES
35	a	170.43	2.65360	YES	YES
36	a	172.21	1.28785	YES	YES
37	a	175.62	1.52318	YES	YES
38	a	179.43	0.94247	YES	YES
39	a	180.56	2.08404	YES	YES
40	a	190.79	0.80523	YES	YES
41	a	192.82	1.59574	YES	YES
42	a	194.53	0.66565	YES	YES
43	a	196.41	0.67589	YES	YES
44	a	217.55	2.68273	YES	YES
45	a	262.01	0.33465	YES	YES
46	a	262.96	0.23988	YES	YES
47	a	263.45	0.71875	YES	YES
48	a	267.89	0.20150	YES	YES
49	a	306.42	3.87015	YES	YES
50	a	310.09	18.43854	YES	YES
51	a	311.78	9.94449	YES	YES
52	a	321.69	10.43829	YES	YES
53	a	368.28	1.99864	YES	YES
54	a	369.05	3.41511	YES	YES
55	a	369.65	1.60335	YES	YES
56	a	372.32	11.59351	YES	YES
57	a	392.78	84.94277	YES	YES
58	a	412.66	109.55311	YES	YES
59	a	482.58	86.79052	YES	YES
60	a	523.11	19.40863	YES	YES
61	a	556.17	31.60436	YES	YES
62	a	570.07	47.22343	YES	YES
63	a	582.50	186.68819	YES	YES
64	a	683.46	4.98980	YES	YES
65	a	685.43	6.47272	YES	YES
66	a	687.23	17.02482	YES	YES
67	a	719.65	0.46653	YES	YES
68	a	800.70	13.07034	YES	YES
69	a	802.25	40.52242	YES	YES
70	a	806.00	73.01691	YES	YES
71	a	811.49	14.06412	YES	YES
72	a	822.95	0.16896	YES	YES
73	a	823.70	0.00283	YES	YES
74	a	824.15	1.36891	YES	YES
75	a	829.33	1.49329	YES	YES
76	a	847.35	5.11845	YES	YES
77	a	848.03	3.33515	YES	YES
78	a	849.97	14.82052	YES	YES
79	a	893.42	29.40712	YES	YES
80	a	965.22	28.29150	YES	YES
81	a	968.83	10.20167	YES	YES
82	a	970.20	7.08050	YES	YES
83	a	974.13	3.71241	YES	YES
84	a	1029.78	59.69457	YES	YES
85	a	1034.29	98.05351	YES	YES
86	a	1036.74	148.45291	YES	YES
87	a	1037.84	59.87066	YES	YES
88	a	1115.89	43.34779	YES	YES
89	a	1120.36	41.36726	YES	YES
90	a	1124.85	73.57816	YES	YES
91	a	1131.66	7.71581	YES	YES
92	a	1132.32	13.38839	YES	YES
93	a	1134.68	11.15541	YES	YES
94	a	1138.68	2.16857	YES	YES
95	a	1148.93	2.19969	YES	YES
96	a	1175.06	2.73306	YES	YES

97	a	1176.20	2.70959	YES	YES
98	a	1177.06	2.58452	YES	YES
99	a	1177.41	2.74693	YES	YES
100	a	1178.09	4.51714	YES	YES
101	a	1181.04	0.46034	YES	YES
102	a	1184.96	0.97808	YES	YES
103	a	1185.43	1.12205	YES	YES
104	a	1224.75	12.08819	YES	YES
105	a	1225.38	21.86791	YES	YES
106	a	1225.90	33.34808	YES	YES
107	a	1229.06	6.31120	YES	YES
108	a	1304.61	1.38988	YES	YES
109	a	1305.66	1.97750	YES	YES
110	a	1306.42	1.86948	YES	YES
111	a	1306.51	5.00670	YES	YES
112	a	1330.06	372.30341	YES	YES
113	a	1331.55	191.93922	YES	YES
114	a	1340.28	801.51804	YES	YES
115	a	1341.80	959.18072	YES	YES
116	a	1410.99	13.71306	YES	YES
117	a	1411.65	28.18961	YES	YES
118	a	1413.28	81.63912	YES	YES
119	a	1415.84	133.01164	YES	YES
120	a	1430.09	13.50870	YES	YES
121	a	1431.03	20.28936	YES	YES
122	a	1432.30	18.42667	YES	YES
123	a	1434.44	18.97759	YES	YES
124	a	1484.20	6.89836	YES	YES
125	a	1486.65	1.25157	YES	YES
126	a	1486.84	7.96942	YES	YES
127	a	1487.27	68.14898	YES	YES
128	a	1487.53	17.11189	YES	YES
129	a	1488.43	57.20292	YES	YES
130	a	1489.23	74.92238	YES	YES
131	a	1489.45	43.54341	YES	YES
132	a	1491.71	4.18745	YES	YES
133	a	1491.99	7.80160	YES	YES
134	a	1492.49	31.99134	YES	YES
135	a	1493.51	26.59886	YES	YES
136	a	1496.17	32.26492	YES	YES
137	a	1498.39	4.02102	YES	YES
138	a	1499.51	13.57805	YES	YES
139	a	1499.80	6.97616	YES	YES
140	a	1500.69	1.29181	YES	YES
141	a	1502.03	13.21630	YES	YES
142	a	1502.80	22.35292	YES	YES
143	a	1504.37	26.65457	YES	YES
144	a	1518.87	5.53684	YES	YES
145	a	1519.71	0.65509	YES	YES
146	a	1520.09	0.55503	YES	YES
147	a	1529.07	21.16004	YES	YES
148	a	1724.01	146.50273	YES	YES
149	a	1741.34	900.49712	YES	YES
150	a	1751.05	1460.55784	YES	YES
151	a	1783.65	25.26727	YES	YES
152	a	3046.95	6.06184	YES	YES
153	a	3047.42	7.17929	YES	YES
154	a	3047.60	8.61401	YES	YES
155	a	3050.45	7.68728	YES	YES
156	a	3056.24	19.25817	YES	YES
157	a	3062.19	19.36059	YES	YES
158	a	3066.84	12.82503	YES	YES
159	a	3067.74	15.26242	YES	YES

160	a	3068.05	17.69009	YES	YES
161	a	3069.19	5.08626	YES	YES
162	a	3069.27	21.22378	YES	YES
163	a	3071.48	12.13186	YES	YES
164	a	3099.16	2.57328	YES	YES
165	a	3104.00	0.28279	YES	YES
166	a	3106.61	0.05028	YES	YES
167	a	3109.69	5.65715	YES	YES
168	a	3114.41	21.68077	YES	YES
169	a	3116.59	13.23919	YES	YES
170	a	3117.17	12.05070	YES	YES
171	a	3117.99	11.28128	YES	YES
172	a	3127.75	20.55168	YES	YES
173	a	3129.37	26.95069	YES	YES
174	a	3131.45	24.04573	YES	YES
175	a	3138.45	16.25506	YES	YES
176	a	3140.17	11.18081	YES	YES
177	a	3149.05	9.76026	YES	YES
178	a	3153.97	3.64037	YES	YES
179	a	3156.47	5.24311	YES	YES
180	a	3175.52	6.00358	YES	YES
181	a	3178.00	5.48102	YES	YES
182	a	3178.52	3.69073	YES	YES
183	a	3179.83	4.33089	YES	YES

$end

Total COSMO energy + OC corr. = -1539.1561233854 H

[Li(EMC)$_5$]$^+$

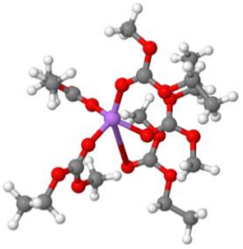

Method: (RI-)B3LYP(D3BJ)/def2-TZVPP
Symmetry: c1

Cartesian coordinates in Ångström:

H	3.4877923	-0.2973513	-0.6029257
H	2.3772418	-1.6713821	-0.4922258
C	2.7792788	-0.9091068	-1.1601661
H	-1.5176078	3.1208592	-2.1594084
H	3.3179979	-1.4094518	-1.9647855
O	-0.5840480	1.9568438	0.0778063
C	-1.7070650	2.0683032	-2.3420901
C	-0.0651649	1.3331845	-0.8284410
O	-0.5101522	1.3078566	-2.0736343
H	-1.9238418	1.8956648	-3.3905963
C	1.6735345	-0.0637171	-1.7425927
O	1.0062624	0.5880717	-0.6245847
H	-2.5193300	1.7131428	-1.7126153
H	2.0498821	0.7128333	-2.4065300
H	0.9445208	-0.6630766	-2.2849254
H	-1.5358922	6.7258591	-3.8739079
C	-0.8824290	5.8619586	-3.7505187

H	-1.4534623	4.9659143	-3.9935599
H	-0.0607105	5.9491763	-4.4602363
C	-0.3659785	5.8188809	-2.3325902
H	-1.1728420	5.7224707	-1.6044908
H	0.2093769	6.7076654	-2.0789070
O	0.4987218	4.6609347	-2.2167554
C	1.1814196	4.5601692	-1.0732073
O	1.0888817	5.3013106	-0.1268527
O	1.9825523	3.4958869	-1.1584095
C	2.8167440	3.2664733	-0.0037405
H	2.2079752	3.0284573	0.8629429
H	3.4234323	4.1459097	0.1988071
H	3.4462566	2.4242169	-0.2716408
O	-1.9316072	2.5663177	2.8805256
H	-0.3267685	4.6312814	2.1773589
C	-2.5848062	3.5626816	3.1291222
H	-6.0914492	3.5452049	4.8445619
C	-1.2610435	4.8878655	1.6870362
H	-1.4160062	4.2357029	0.8329516
H	-4.9974163	3.9406717	6.1787809
C	-5.4408958	4.3154773	5.2569873
O	-3.5936814	3.5139198	3.9759358
O	-2.3692267	4.7627516	2.6127857
H	-1.2527602	5.9278896	1.3857428
H	-6.0502579	5.1865799	5.4989995
C	-4.3754661	4.7115830	4.2653533
H	-4.7996654	5.0776597	3.3317319
H	-3.7049993	5.4714175	4.6638730
O	1.0823759	2.3001937	2.8385941
H	-0.5291677	1.9019792	4.9806410
C	1.8014294	2.4762434	3.8025746
H	5.5283211	3.6486816	3.6317789
C	0.3319232	1.2797523	5.2103717
H	0.3313968	0.4059876	4.5638361
H	4.6405471	5.1427854	3.9718680
C	4.9605380	4.1886409	4.3888103
O	2.9285618	3.1501862	3.6916905
O	1.5547514	2.0310534	5.0273056
H	0.3320074	0.9916107	6.2557619
H	5.6196465	4.3869402	5.2342561
C	3.7769350	3.3797847	4.8575263
H	4.0728601	2.4131696	5.2620722
H	3.1907397	3.9072125	5.6081926
Li	-0.5177689	1.6591202	2.0026650
H	-2.4344181	-2.7496598	-2.3504462
C	-2.3163593	-1.9119941	-1.6629326
H	-3.3011207	-1.6359954	-1.2879171
H	-1.9016514	-1.0705575	-2.2167795
C	-1.4033413	-2.3176728	-0.5327920
H	-1.7956202	-3.1637870	0.0285793
H	-0.4029769	-2.5675430	-0.8827902
O	-1.3051807	-1.1752702	0.3692011
C	-0.5146080	-1.2216757	1.4256400
O	-0.4085394	-0.2833021	2.1893563
O	0.1357135	-2.3713117	1.5579450
C	1.0271689	-2.4753791	2.6877656
H	1.7799747	-1.6920785	2.6396543
H	0.4636712	-2.3941658	3.6142620
H	1.4830036	-3.4556912	2.6026642

SCF energy GEOOPT = -1922.021784612 H
ZPE = 1641. kJ/mol
FREEH energy = 1771.42 kJ/mol

FREEH entropy =   1.29449 kJ/mol/K

$vibrational spectrum

# mode	symmetry	wave number cm**(-1)	IR intensity km/mol	selection rules IR	RAMAN
1		-0.00	0.00000	-	-
2		0.00	0.00000	-	-
3		0.00	0.00000	-	-
4		0.00	0.00000	-	-
5		0.00	0.00000	-	-
6		0.00	0.00000	-	-
7	a	7.00	0.22202	YES	YES
8	a	10.37	0.32308	YES	YES
9	a	10.72	0.59199	YES	YES
10	a	16.52	0.95945	YES	YES
11	a	21.75	0.03531	YES	YES
12	a	23.91	0.73300	YES	YES
13	a	24.59	0.78923	YES	YES
14	a	29.74	0.55350	YES	YES
15	a	34.10	0.90348	YES	YES
16	a	37.65	1.59340	YES	YES
17	a	40.14	0.92139	YES	YES
18	a	42.90	1.23802	YES	YES
19	a	53.03	0.73376	YES	YES
20	a	53.21	0.83606	YES	YES
21	a	59.06	0.58066	YES	YES
22	a	63.60	1.50959	YES	YES
23	a	65.47	0.10276	YES	YES
24	a	68.25	1.05681	YES	YES
25	a	72.17	1.63396	YES	YES
26	a	73.64	0.49263	YES	YES
27	a	75.81	3.02357	YES	YES
28	a	78.06	0.70388	YES	YES
29	a	82.54	1.04168	YES	YES
30	a	85.04	1.55162	YES	YES
31	a	91.97	4.32335	YES	YES
32	a	94.70	1.53392	YES	YES
33	a	96.28	0.90193	YES	YES
34	a	104.47	3.51443	YES	YES
35	a	124.60	5.18344	YES	YES
36	a	129.33	5.85352	YES	YES
37	a	130.39	0.14311	YES	YES
38	a	135.92	1.10868	YES	YES
39	a	139.52	7.47653	YES	YES
40	a	141.86	1.15794	YES	YES
41	a	146.71	0.24039	YES	YES
42	a	158.16	1.37834	YES	YES
43	a	168.80	0.01106	YES	YES
44	a	173.84	0.13730	YES	YES
45	a	175.49	3.43724	YES	YES
46	a	177.91	1.02374	YES	YES
47	a	178.38	1.23969	YES	YES
48	a	180.83	0.97558	YES	YES
49	a	181.89	0.61231	YES	YES
50	a	189.12	1.10904	YES	YES
51	a	192.35	1.18539	YES	YES
52	a	197.49	0.74075	YES	YES
53	a	199.45	2.32889	YES	YES
54	a	200.81	2.27629	YES	YES
55	a	210.93	2.02959	YES	YES
56	a	261.73	0.16737	YES	YES
57	a	261.91	0.59164	YES	YES
58	a	262.22	0.08174	YES	YES

59	a	264.50	0.70922	YES	YES
60	a	272.49	0.03635	YES	YES
61	a	305.44	5.43689	YES	YES
62	a	311.50	8.50047	YES	YES
63	a	312.37	12.73065	YES	YES
64	a	322.68	9.25009	YES	YES
65	a	325.48	1.05989	YES	YES
66	a	368.33	5.09920	YES	YES
67	a	369.03	0.49452	YES	YES
68	a	371.22	7.76765	YES	YES
69	a	372.42	8.96339	YES	YES
70	a	373.88	2.31313	YES	YES
71	a	393.54	62.68382	YES	YES
72	a	433.44	115.72365	YES	YES
73	a	469.66	157.41633	YES	YES
74	a	508.03	3.72885	YES	YES
75	a	557.51	36.27967	YES	YES
76	a	562.15	23.85049	YES	YES
77	a	577.00	77.66131	YES	YES
78	a	582.51	136.04953	YES	YES
79	a	682.75	12.12947	YES	YES
80	a	683.37	3.83563	YES	YES
81	a	691.18	4.75157	YES	YES
82	a	693.44	4.12191	YES	YES
83	a	719.75	1.02521	YES	YES
84	a	791.66	101.10835	YES	YES
85	a	798.81	15.90017	YES	YES
86	a	799.44	18.97830	YES	YES
87	a	801.95	28.34584	YES	YES
88	a	802.63	12.40184	YES	YES
89	a	820.52	2.11292	YES	YES
90	a	823.36	0.02248	YES	YES
91	a	823.55	0.00939	YES	YES
92	a	825.30	0.15339	YES	YES
93	a	825.64	0.11033	YES	YES
94	a	848.35	5.49646	YES	YES
95	a	848.79	4.01560	YES	YES
96	a	853.81	6.19816	YES	YES
97	a	858.07	6.94317	YES	YES
98	a	891.48	23.98713	YES	YES
99	a	952.01	39.85116	YES	YES
100	a	969.66	10.00528	YES	YES
101	a	971.97	6.82771	YES	YES
102	a	972.21	5.04663	YES	YES
103	a	973.24	3.11749	YES	YES
104	a	1028.29	85.57655	YES	YES
105	a	1036.76	135.70186	YES	YES
106	a	1038.63	104.31306	YES	YES
107	a	1041.33	59.80126	YES	YES
108	a	1045.46	78.14406	YES	YES
109	a	1117.69	36.83956	YES	YES
110	a	1120.23	48.70431	YES	YES
111	a	1123.48	79.22170	YES	YES
112	a	1124.69	55.25356	YES	YES
113	a	1131.15	13.32875	YES	YES
114	a	1132.11	6.89147	YES	YES
115	a	1132.29	18.72447	YES	YES
116	a	1132.77	10.12711	YES	YES
117	a	1132.86	20.26648	YES	YES
118	a	1136.27	6.28418	YES	YES
119	a	1173.04	1.31053	YES	YES
120	a	1177.22	6.47197	YES	YES
121	a	1177.72	3.07627	YES	YES

122	a	1178.05	0.19282	YES	YES
123	a	1178.50	8.97493	YES	YES
124	a	1179.52	1.28827	YES	YES
125	a	1180.04	0.55624	YES	YES
126	a	1181.17	0.39332	YES	YES
127	a	1182.07	0.84293	YES	YES
128	a	1185.10	6.04178	YES	YES
129	a	1219.23	18.84027	YES	YES
130	a	1221.10	24.64095	YES	YES
131	a	1225.63	16.37939	YES	YES
132	a	1226.03	8.44403	YES	YES
133	a	1227.21	25.57553	YES	YES
134	a	1284.09	365.41061	YES	YES
135	a	1301.94	9.78462	YES	YES
136	a	1305.48	1.66545	YES	YES
137	a	1305.64	1.59162	YES	YES
138	a	1305.82	3.09624	YES	YES
139	a	1306.23	4.08574	YES	YES
140	a	1325.66	214.08267	YES	YES
141	a	1332.41	148.24870	YES	YES
142	a	1335.40	1185.45127	YES	YES
143	a	1340.52	1118.07603	YES	YES
144	a	1407.02	10.36553	YES	YES
145	a	1408.80	128.87150	YES	YES
146	a	1410.48	8.48028	YES	YES
147	a	1412.04	102.90999	YES	YES
148	a	1413.99	13.00335	YES	YES
149	a	1428.67	26.29657	YES	YES
150	a	1429.54	20.48602	YES	YES
151	a	1430.07	2.41210	YES	YES
152	a	1430.60	12.80032	YES	YES
153	a	1431.28	1.75177	YES	YES
154	a	1477.97	95.27430	YES	YES
155	a	1481.82	63.86335	YES	YES
156	a	1483.87	44.45817	YES	YES
157	a	1485.42	25.65708	YES	YES
158	a	1485.91	3.37471	YES	YES
159	a	1486.50	7.70921	YES	YES
160	a	1486.70	10.08091	YES	YES
161	a	1487.26	14.76536	YES	YES
162	a	1488.09	24.15544	YES	YES
163	a	1489.18	11.69640	YES	YES
164	a	1489.63	50.03077	YES	YES
165	a	1491.20	47.20994	YES	YES
166	a	1492.38	7.47075	YES	YES
167	a	1493.86	7.54945	YES	YES
168	a	1496.09	26.33400	YES	YES
169	a	1497.67	12.19229	YES	YES
170	a	1498.80	9.94157	YES	YES
171	a	1499.30	1.07387	YES	YES
172	a	1499.57	5.32740	YES	YES
173	a	1499.87	8.46511	YES	YES
174	a	1501.50	21.34139	YES	YES
175	a	1502.56	4.04941	YES	YES
176	a	1504.30	5.48638	YES	YES
177	a	1507.22	9.22858	YES	YES
178	a	1508.25	21.35839	YES	YES
179	a	1519.51	2.21076	YES	YES
180	a	1520.12	0.53634	YES	YES
181	a	1520.23	0.34961	YES	YES
182	a	1524.75	0.06673	YES	YES
183	a	1524.91	7.07203	YES	YES
184	a	1745.42	186.88429	YES	YES

185	a	1749.71	821.21894	YES	YES
186	a	1755.55	1326.99162	YES	YES
187	a	1784.03	470.16461	YES	YES
188	a	1790.71	182.96087	YES	YES
189	a	3043.84	8.51063	YES	YES
190	a	3045.40	10.38671	YES	YES
191	a	3047.05	12.67660	YES	YES
192	a	3047.07	3.48222	YES	YES
193	a	3048.43	6.94390	YES	YES
194	a	3049.93	20.26651	YES	YES
195	a	3064.05	19.18912	YES	YES
196	a	3064.96	13.29015	YES	YES
197	a	3066.11	16.12345	YES	YES
198	a	3066.81	10.09207	YES	YES
199	a	3068.83	13.85707	YES	YES
200	a	3069.66	20.37367	YES	YES
201	a	3070.95	11.98287	YES	YES
202	a	3074.61	17.35837	YES	YES
203	a	3082.93	13.82408	YES	YES
204	a	3095.08	0.56759	YES	YES
205	a	3101.49	1.93021	YES	YES
206	a	3103.25	0.48201	YES	YES
207	a	3104.14	0.29166	YES	YES
208	a	3105.70	1.04275	YES	YES
209	a	3113.93	20.24016	YES	YES
210	a	3115.53	14.07637	YES	YES
211	a	3116.14	17.14649	YES	YES
212	a	3116.18	10.57146	YES	YES
213	a	3117.20	12.25606	YES	YES
214	a	3122.35	28.86309	YES	YES
215	a	3127.70	36.41488	YES	YES
216	a	3128.79	26.31046	YES	YES
217	a	3129.34	24.24186	YES	YES
218	a	3130.82	18.75475	YES	YES
219	a	3142.70	11.36776	YES	YES
220	a	3153.34	11.90703	YES	YES
221	a	3154.07	3.06500	YES	YES
222	a	3160.74	7.45030	YES	YES
223	a	3167.59	5.12081	YES	YES
224	a	3173.88	8.38081	YES	YES
225	a	3174.03	5.26529	YES	YES
226	a	3176.51	4.11468	YES	YES
227	a	3180.54	4.47124	YES	YES
228	a	3194.72	3.07287	YES	YES

$end

Total COSMO energy + OC corr. = -1922.0787187364 H

$[Li(EC)_2(EMC)_2]^+$

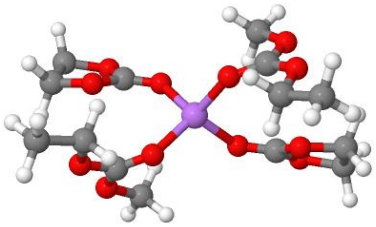

Method: (RI-)B3LYP(D3BJ)/def2-TZVPP
Symmetry: c2

```
Cartesian coordinates in Ångström:
H     0.9674754   -4.8618116   -2.9293807
H    -0.6010274   -4.6884333   -3.7292820
C     0.3055003   -4.1603861   -3.4365403
H    -2.8283177   -1.6838395    1.2087352
H     0.8057163   -3.8108908   -4.3399478
O    -0.8932408   -1.4022583   -0.5592762
C    -2.1145791   -2.3906904    1.6240443
C    -1.0381066   -2.6092555   -0.4612157
O    -1.5758868   -3.2291687    0.5788688
H    -2.6054934   -3.0733321    2.3088013
C    -0.0152210   -2.9798588   -2.5546634
O    -0.6852495   -3.4946248   -1.3675657
H    -1.3189153   -1.8486909    2.1298890
H     0.8780765   -2.4476594   -2.2303035
H    -0.6842567   -2.2710117   -3.0400484
H    -0.8780765    2.4476594   -2.2303035
H    -0.9674754    4.8618116   -2.9293807
H    -0.8057163    3.8108908   -4.3399478
C    -0.3055003    4.1603861   -3.4365403
C     0.0152210    2.9798588   -2.5546634
H     1.3189153    1.8486909    2.1298890
O     0.6852495    3.4946248   -1.3675657
C     1.0381066    2.6092555   -0.4612157
O     0.8932408    1.4022583   -0.5592762
H     0.6842567    2.2710117   -3.0400484
O     1.5758868    3.2291687    0.5788688
C     2.1145791    2.3906904    1.6240443
H     0.6010274    4.6884333   -3.7292820
H     2.6054934    3.0733321    2.3088013
H     2.8283177    1.6838395    1.2087352
H     3.6902723   -3.9607310    2.4129470
H     2.7272019   -4.6897766    0.3132729
O     1.6737147   -2.9321959    0.5657275
C     2.6029184   -4.0043780    2.4055891
C     1.6399084   -2.1023790    1.6178738
C     2.0176401   -4.2644488    1.0157301
O     1.2295674   -0.9712677    1.5665480
O     2.0992212   -2.6842084    2.7236434
H     2.2514299   -4.6939532    3.1664252
H     1.1041472   -4.8559588    1.0340286
H    -1.1041472    4.8559588    1.0340286
H    -2.2514299    4.6939532    3.1664252
O    -2.0992212    2.6842084    2.7236434
C    -2.0176401    4.2644488    1.0157301
C    -1.6399084    2.1023790    1.6178738
C    -2.6029184    4.0043780    2.4055891
O    -1.2295674    0.9712677    1.5665480
O    -1.6737147    2.9321959    0.5657275
H    -2.7272019    4.6897766    0.3132729
H    -3.6902723    3.9607310    2.4129470
Li    0.0000000   -0.0000000    0.3719814

SCF energy GEOOPT =  -1458.075979753 H
ZPE =   1060. kJ/mol
FREEH energy =  1146.26 kJ/mol
FREEH entropy =   0.95445 kJ/mol/K

$vibrational spectrum
#  mode     symmetry     wave number   IR intensity    selection rules
#                         cm**(-1)        km/mol          IR     RAMAN
     1                        0.00         0.00000         -       -
```

2		0.00	0.00000	-	-
3		0.00	0.00000	-	-
4		0.00	0.00000	-	-
5		0.00	0.00000	-	-
6		0.00	0.00000	-	-
7	b	10.38	0.55991	YES	YES
8	a	10.64	2.29943	YES	YES
9	a	13.16	0.00690	YES	YES
10	a	17.91	0.24400	YES	YES
11	b	20.61	3.90735	YES	YES
12	a	26.49	0.17721	YES	YES
13	b	28.58	0.09467	YES	YES
14	b	37.05	1.01931	YES	YES
15	a	47.70	0.05685	YES	YES
16	b	60.56	1.21440	YES	YES
17	a	62.27	0.61187	YES	YES
18	b	64.96	0.98230	YES	YES
19	a	65.22	2.14527	YES	YES
20	a	69.31	1.17659	YES	YES
21	b	81.73	1.12868	YES	YES
22	a	82.95	1.40000	YES	YES
23	b	87.30	4.17604	YES	YES
24	a	88.41	1.52586	YES	YES
25	b	115.25	12.98702	YES	YES
26	a	118.98	2.49723	YES	YES
27	b	142.28	2.23198	YES	YES
28	a	143.18	0.12320	YES	YES
29	b	148.32	0.88675	YES	YES
30	a	149.99	0.00947	YES	YES
31	b	163.68	5.06886	YES	YES
32	a	165.93	0.02630	YES	YES
33	b	177.58	3.01252	YES	YES
34	a	189.50	0.20420	YES	YES
35	b	192.28	2.58850	YES	YES
36	a	198.42	0.81231	YES	YES
37	b	220.81	4.80061	YES	YES
38	a	225.46	0.60753	YES	YES
39	b	264.07	0.20729	YES	YES
40	a	264.23	0.46925	YES	YES
41	b	319.78	6.74607	YES	YES
42	a	320.88	13.55128	YES	YES
43	a	360.25	1.72378	YES	YES
44	b	370.67	28.67671	YES	YES
45	b	385.97	102.19185	YES	YES
46	a	396.93	74.19944	YES	YES
47	b	479.57	103.50345	YES	YES
48	a	518.00	2.50874	YES	YES
49	b	535.36	3.71015	YES	YES
50	a	538.02	5.27173	YES	YES
51	b	551.35	183.01111	YES	YES
52	a	712.72	3.74283	YES	YES
53	b	712.74	1.27545	YES	YES
54	b	718.23	0.96525	YES	YES
55	a	718.26	0.13150	YES	YES
56	b	737.74	72.35385	YES	YES
57	a	738.15	2.56003	YES	YES
58	a	788.21	1.00307	YES	YES
59	b	789.26	62.86374	YES	YES
60	b	807.38	39.24735	YES	YES
61	a	807.59	25.40482	YES	YES
62	a	824.32	5.36727	YES	YES
63	b	824.52	8.68781	YES	YES
64	a	882.22	1.25324	YES	YES

65	b	882.23	0.19141	YES	YES
66	b	887.11	19.15006	YES	YES
67	a	887.27	57.28266	YES	YES
68	b	915.24	22.53132	YES	YES
69	a	915.28	2.06597	YES	YES
70	a	960.35	17.80791	YES	YES
71	b	962.01	25.24041	YES	YES
72	b	977.17	25.50032	YES	YES
73	a	977.62	0.06036	YES	YES
74	b	1024.32	35.17560	YES	YES
75	a	1025.16	135.62536	YES	YES
76	b	1029.97	0.98555	YES	YES
77	a	1030.13	4.18190	YES	YES
78	b	1094.48	368.00481	YES	YES
79	a	1096.85	0.00070	YES	YES
80	b	1134.93	4.37126	YES	YES
81	a	1135.26	5.59548	YES	YES
82	b	1145.97	1.62509	YES	YES
83	a	1146.04	7.00225	YES	YES
84	b	1155.78	1.39465	YES	YES
85	a	1155.79	0.00278	YES	YES
86	b	1176.06	0.06142	YES	YES
87	a	1176.24	0.82309	YES	YES
88	a	1181.30	2.49032	YES	YES
89	b	1181.51	6.42142	YES	YES
90	b	1195.47	66.81682	YES	YES
91	a	1196.68	329.63674	YES	YES
92	b	1227.36	3.70503	YES	YES
93	a	1227.53	19.67133	YES	YES
94	b	1242.36	0.38439	YES	YES
95	a	1242.46	34.91704	YES	YES
96	b	1251.12	25.60724	YES	YES
97	a	1251.24	0.88092	YES	YES
98	a	1304.18	0.41447	YES	YES
99	b	1304.20	2.44875	YES	YES
100	b	1337.75	274.97640	YES	YES
101	a	1340.77	1075.87063	YES	YES
102	b	1393.56	0.45906	YES	YES
103	a	1393.63	0.13819	YES	YES
104	b	1414.96	27.07460	YES	YES
105	a	1415.37	97.11297	YES	YES
106	b	1429.59	13.72259	YES	YES
107	a	1430.11	126.13619	YES	YES
108	b	1434.54	27.58808	YES	YES
109	a	1435.12	36.68661	YES	YES
110	b	1486.66	8.21012	YES	YES
111	a	1486.68	4.25318	YES	YES
112	b	1488.47	57.88958	YES	YES
113	a	1488.70	63.89738	YES	YES
114	a	1498.14	26.03865	YES	YES
115	b	1498.43	50.91968	YES	YES
116	b	1499.24	3.20310	YES	YES
117	a	1499.60	3.16973	YES	YES
118	b	1500.08	6.70267	YES	YES
119	a	1500.10	51.69697	YES	YES
120	b	1520.08	19.23854	YES	YES
121	a	1521.10	19.23972	YES	YES
122	b	1525.36	6.40625	YES	YES
123	a	1525.44	37.09749	YES	YES
124	b	1532.98	0.18158	YES	YES
125	a	1533.02	2.87209	YES	YES
126	b	1729.37	666.52800	YES	YES
127	a	1741.20	6.01157	YES	YES

128	b	1822.05	1865.51835	YES	YES
129	a	1843.90	14.97462	YES	YES
130	b	3046.98	12.29281	YES	YES
131	a	3047.00	4.72751	YES	YES
132	b	3065.20	20.39623	YES	YES
133	a	3065.49	3.66944	YES	YES
134	a	3067.69	17.54838	YES	YES
135	b	3067.72	16.03970	YES	YES
136	b	3084.76	29.00077	YES	YES
137	a	3084.80	0.09455	YES	YES
138	a	3087.53	5.03143	YES	YES
139	b	3087.54	15.80045	YES	YES
140	a	3102.38	0.02832	YES	YES
141	b	3102.40	1.07955	YES	YES
142	b	3115.68	15.57109	YES	YES
143	a	3115.76	12.85980	YES	YES
144	a	3128.00	11.09561	YES	YES
145	b	3128.06	39.99290	YES	YES
146	b	3146.35	9.23918	YES	YES
147	a	3146.36	0.35212	YES	YES
148	a	3146.47	2.70772	YES	YES
149	b	3146.49	13.41735	YES	YES
150	a	3160.35	1.94197	YES	YES
151	b	3160.35	6.76056	YES	YES
152	b	3174.76	9.11452	YES	YES
153	a	3174.79	3.34145	YES	YES

$end

Total COSMO energy + OC corr. = -1458.1396011718 H

$[Li(EC)_2(EMC)_3]^+$

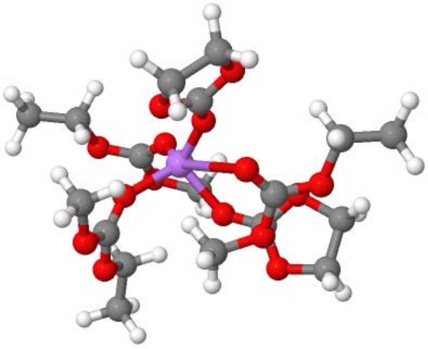

Method: (RI-)B3LYP(D3BJ)/def2-TZVPP
Symmetry: c1

Cartesian coordinates in Ångström:

H	2.5044821	-2.2403760	-0.2341983
H	2.6283753	-1.6668772	-1.9044390
C	2.7474449	-1.3824173	-0.8599294
H	-2.6249202	1.5757747	-1.3619322
H	3.7926369	-1.1199109	-0.6939853
O	-0.2525572	1.4157967	-0.0375593
C	-2.7416398	0.7152991	-0.7064090
C	-0.4348116	0.3072274	-0.5156964
O	-1.6179865	-0.1755071	-0.8566928
H	-3.6065246	0.1293015	-0.9976143
C	1.8785423	-0.1989768	-0.5162666
O	0.4966969	-0.5848243	-0.7738791

```
H    -2.8288082    1.0562102    0.3214760
H     1.9602391    0.0815984    0.5312902
H     2.1061249    0.6648533   -1.1402457
H     1.0595448    5.0416038   -1.2231537
H     1.9584882    5.0255574   -3.5720861
H     0.2120868    4.7579303   -3.5656226
C     1.1726438    4.3340019   -3.2713871
C     1.1849499    4.1134429   -1.7794250
H     4.2623700    3.3566694    1.9697333
O     2.4882415    3.5571295   -1.4367174
C     2.7016981    3.2823532   -0.1670967
O     1.8965086    3.4276480    0.7371958
H     0.4212690    3.4077529   -1.4573781
O     3.9297526    2.8139372   -0.0145244
C     4.3188395    2.4774520    1.3318784
H     1.3119515    3.3956998   -3.8074972
H     5.3421215    2.1274264    1.2516927
H     3.6721376    1.6964467    1.7263036
H     0.4694826   -1.2181496    5.7824850
H     0.5299095    0.9814872    6.7219758
O     1.0958206    1.4990561    4.8120495
C     1.3935418   -0.6836372    5.5783856
C     1.2418493    0.6824499    3.7721870
C     1.3473093    0.7760936    6.0402035
O     1.1430348    1.0257807    2.6189572
O     1.5278701   -0.5652289    4.1423697
H     2.2482362   -1.2372038    5.9543787
H     2.2903344    1.1313206    6.4504795
H    -4.8972696    4.0988471    1.3964375
H    -4.2620989    5.4757912    3.2711629
O    -2.6034101    4.2897918    3.5515893
C    -4.3418897    3.5676105    2.1637594
C    -2.0956289    3.6081662    2.5260885
C    -4.0333999    4.4205980    3.4014922
O    -0.9250404    3.3520813    2.4103994
O    -3.0308392    3.2615085    1.6390314
H    -4.8275540    2.6288417    2.4134116
H    -4.5037933    4.0405662    4.3031299
Li    0.4452674    2.4354930    1.4629013
H    -1.0399175   -1.4022890    2.6378957
C    -1.7752851   -0.6297192    2.8336687
H    -1.6565961    0.1933443    2.1347482
H    -2.7836679   -1.0314210    2.7692098
O    -1.5042433   -0.1752857    4.1737369
C    -2.2807793    0.8178850    4.6165491
O    -3.1658173    1.3471564    3.9868555
O    -1.9096581    1.1169605    5.8556698
C    -2.6303435    2.2071676    6.4946113
H    -3.6893831    1.9524248    6.5061854
H    -2.4890560    3.1025651    5.8916139
C    -2.0716505    2.3672913    7.8869160
H    -2.1813703    1.4487122    8.4625694
H    -1.0179077    2.6447378    7.8592855
H    -2.6121041    3.1596428    8.4049859

SCF energy GEOOPT =  -1841.002950865 H
ZPE =   1389. kJ/mol
FREEH energy =  1501.50 kJ/mol
FREEH entropy =   1.16382 kJ/mol/K

$vibrational spectrum
#  mode     symmetry     wave number   IR intensity    selection rules
#                         cm**(-1)        km/mol          IR     RAMAN
```

1		-0.00	0.00000	-	-
2		-0.00	0.00000	-	-
3		0.00	0.00000	-	-
4		0.00	0.00000	-	-
5		0.00	0.00000	-	-
6		0.00	0.00000	-	-
7	a	10.61	0.09774	YES	YES
8	a	13.24	0.02319	YES	YES
9	a	15.09	0.11220	YES	YES
10	a	17.75	0.92032	YES	YES
11	a	22.51	0.15393	YES	YES
12	a	25.31	0.23434	YES	YES
13	a	31.85	0.35885	YES	YES
14	a	37.08	2.29066	YES	YES
15	a	39.58	0.26235	YES	YES
16	a	42.95	1.48720	YES	YES
17	a	45.17	0.34378	YES	YES
18	a	49.25	0.03271	YES	YES
19	a	50.53	0.17288	YES	YES
20	a	52.85	0.71243	YES	YES
21	a	59.48	1.40788	YES	YES
22	a	65.42	0.10574	YES	YES
23	a	67.25	0.59136	YES	YES
24	a	71.71	0.63049	YES	YES
25	a	78.89	1.52118	YES	YES
26	a	82.04	3.26801	YES	YES
27	a	89.08	1.83429	YES	YES
28	a	90.26	1.95012	YES	YES
29	a	95.18	3.22085	YES	YES
30	a	100.46	2.15150	YES	YES
31	a	105.98	1.61076	YES	YES
32	a	112.98	7.44482	YES	YES
33	a	115.90	2.55178	YES	YES
34	a	121.78	3.93040	YES	YES
35	a	123.81	2.66173	YES	YES
36	a	130.52	5.04091	YES	YES
37	a	132.68	1.45824	YES	YES
38	a	147.46	0.66167	YES	YES
39	a	148.70	2.34809	YES	YES
40	a	154.92	14.17327	YES	YES
41	a	160.05	4.89234	YES	YES
42	a	171.27	0.71138	YES	YES
43	a	177.15	4.07598	YES	YES
44	a	183.97	0.60967	YES	YES
45	a	185.80	0.66977	YES	YES
46	a	188.60	1.55632	YES	YES
47	a	197.70	1.46766	YES	YES
48	a	227.79	6.56270	YES	YES
49	a	233.52	0.82619	YES	YES
50	a	261.83	0.45841	YES	YES
51	a	262.99	0.46375	YES	YES
52	a	270.23	0.42363	YES	YES
53	a	315.16	10.52444	YES	YES
54	a	320.98	10.53202	YES	YES
55	a	323.81	6.20270	YES	YES
56	a	369.78	15.10591	YES	YES
57	a	370.99	8.45227	YES	YES
58	a	371.26	11.00565	YES	YES
59	a	388.51	109.44696	YES	YES
60	a	418.07	92.15830	YES	YES
61	a	477.45	127.03296	YES	YES
62	a	509.87	2.05165	YES	YES
63	a	516.77	7.92643	YES	YES

64	a	525.11	27.18269	YES	YES
65	a	534.68	12.60343	YES	YES
66	a	540.19	59.14176	YES	YES
67	a	719.54	0.20382	YES	YES
68	a	719.74	0.16346	YES	YES
69	a	720.14	0.86517	YES	YES
70	a	726.93	3.44739	YES	YES
71	a	741.94	4.87882	YES	YES
72	a	745.72	35.21296	YES	YES
73	a	751.76	39.59237	YES	YES
74	a	776.62	51.07557	YES	YES
75	a	783.25	44.17662	YES	YES
76	a	804.26	15.80096	YES	YES
77	a	810.40	45.78434	YES	YES
78	a	810.77	29.11060	YES	YES
79	a	827.54	1.10117	YES	YES
80	a	827.71	6.79674	YES	YES
81	a	835.17	1.13048	YES	YES
82	a	866.08	0.33353	YES	YES
83	a	876.30	0.75250	YES	YES
84	a	887.03	11.51626	YES	YES
85	a	887.73	48.73781	YES	YES
86	a	888.86	31.73652	YES	YES
87	a	916.16	17.31567	YES	YES
88	a	917.56	15.68257	YES	YES
89	a	955.68	28.22574	YES	YES
90	a	964.50	23.43271	YES	YES
91	a	965.27	31.90007	YES	YES
92	a	985.46	12.08763	YES	YES
93	a	988.07	8.18633	YES	YES
94	a	1023.70	32.51599	YES	YES
95	a	1026.03	120.07457	YES	YES
96	a	1027.78	82.58535	YES	YES
97	a	1032.70	5.26563	YES	YES
98	a	1038.27	3.83817	YES	YES
99	a	1102.90	179.00704	YES	YES
100	a	1109.16	155.38827	YES	YES
101	a	1134.39	5.04270	YES	YES
102	a	1135.58	2.04336	YES	YES
103	a	1138.00	4.02607	YES	YES
104	a	1139.05	5.53738	YES	YES
105	a	1147.42	1.31868	YES	YES
106	a	1148.67	1.47356	YES	YES
107	a	1154.70	0.91202	YES	YES
108	a	1155.36	0.45714	YES	YES
109	a	1178.27	4.49899	YES	YES
110	a	1181.95	1.09131	YES	YES
111	a	1184.59	1.20912	YES	YES
112	a	1185.34	3.12588	YES	YES
113	a	1187.56	5.78413	YES	YES
114	a	1188.31	6.65275	YES	YES
115	a	1205.45	130.49879	YES	YES
116	a	1209.01	132.62753	YES	YES
117	a	1223.81	14.21033	YES	YES
118	a	1228.16	11.08889	YES	YES
119	a	1229.94	9.33827	YES	YES
120	a	1245.42	6.30118	YES	YES
121	a	1247.27	5.61691	YES	YES
122	a	1257.03	21.78089	YES	YES
123	a	1257.69	5.92774	YES	YES
124	a	1302.26	812.37329	YES	YES
125	a	1306.46	11.07361	YES	YES
126	a	1308.02	1.06631	YES	YES

127	a	1311.31	13.89356	YES	YES
128	a	1338.78	264.07638	YES	YES
129	a	1344.91	1028.91023	YES	YES
130	a	1396.36	0.33937	YES	YES
131	a	1397.79	0.72941	YES	YES
132	a	1410.88	23.92081	YES	YES
133	a	1416.62	31.09702	YES	YES
134	a	1417.55	81.38478	YES	YES
135	a	1431.38	5.68684	YES	YES
136	a	1435.84	16.14443	YES	YES
137	a	1437.34	17.33620	YES	YES
138	a	1438.64	72.07785	YES	YES
139	a	1439.61	95.16692	YES	YES
140	a	1484.05	46.88036	YES	YES
141	a	1486.19	5.76434	YES	YES
142	a	1486.41	7.21894	YES	YES
143	a	1489.23	9.22717	YES	YES
144	a	1489.80	69.43458	YES	YES
145	a	1490.92	3.99064	YES	YES
146	a	1491.43	22.17193	YES	YES
147	a	1491.78	14.03696	YES	YES
148	a	1492.56	64.73616	YES	YES
149	a	1497.45	49.18828	YES	YES
150	a	1501.15	6.06996	YES	YES
151	a	1501.34	10.06937	YES	YES
152	a	1502.48	12.11443	YES	YES
153	a	1503.80	13.36154	YES	YES
154	a	1506.42	12.34795	YES	YES
155	a	1518.87	18.24224	YES	YES
156	a	1519.63	10.16272	YES	YES
157	a	1525.04	3.62203	YES	YES
158	a	1526.15	19.27293	YES	YES
159	a	1527.23	6.19325	YES	YES
160	a	1531.50	13.48254	YES	YES
161	a	1531.56	4.53269	YES	YES
162	a	1722.73	320.05093	YES	YES
163	a	1743.25	780.07477	YES	YES
164	a	1770.99	166.65604	YES	YES
165	a	1817.15	1012.77158	YES	YES
166	a	1845.28	646.05582	YES	YES
167	a	3044.49	8.93524	YES	YES
168	a	3046.66	8.54109	YES	YES
169	a	3047.70	8.06637	YES	YES
170	a	3061.48	18.04752	YES	YES
171	a	3065.04	11.56265	YES	YES
172	a	3066.19	14.39894	YES	YES
173	a	3066.41	4.05663	YES	YES
174	a	3066.77	19.95952	YES	YES
175	a	3069.89	17.57735	YES	YES
176	a	3088.17	20.36268	YES	YES
177	a	3089.17	18.36450	YES	YES
178	a	3097.06	13.33510	YES	YES
179	a	3098.33	18.75262	YES	YES
180	a	3102.09	2.51805	YES	YES
181	a	3106.02	0.71855	YES	YES
182	a	3112.48	4.00651	YES	YES
183	a	3113.79	14.75475	YES	YES
184	a	3114.01	14.56937	YES	YES
185	a	3115.19	15.04257	YES	YES
186	a	3127.19	20.52368	YES	YES
187	a	3130.35	18.85503	YES	YES
188	a	3136.28	9.02453	YES	YES
189	a	3137.74	12.69301	YES	YES

190	a	3141.44	1.58080	YES	YES
191	a	3145.93	13.82883	YES	YES
192	a	3150.55	9.31126	YES	YES
193	a	3152.33	4.86553	YES	YES
194	a	3160.43	8.74278	YES	YES
195	a	3170.59	2.47285	YES	YES
196	a	3172.46	5.90766	YES	YES
197	a	3174.99	5.92915	YES	YES
198	a	3175.89	4.24633	YES	YES

$end

Total COSMO energy + OC corr. = -1841.0645841998 H

$[Li(EC)_3(EMC)_2]^+$

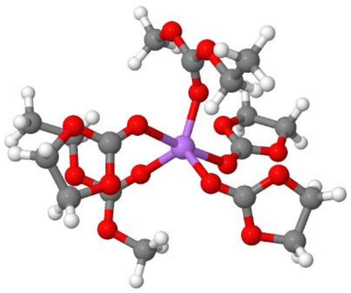

Method: (RI-)B3LYP(D3BJ)/def2-TZVPP
Symmetry: c1

Cartesian coordinates in Ångström:

H	-0.2375133	-2.9722049	0.6266004
H	0.9620383	-2.1431407	-0.3746953
C	0.5963886	-2.2712488	0.6435231
H	-2.2783325	3.1622685	0.1948328
H	1.3971900	-2.7037572	1.2441388
O	-1.1312584	1.3439743	1.8030256
C	-2.9992046	2.3472003	0.2035228
C	-1.4152496	0.7256859	0.7902080
O	-2.3422185	1.1002410	-0.0783642
H	-3.7280961	2.4726107	-0.5898710
C	0.1855052	-0.9486133	1.2403599
O	-0.8779570	-0.4095616	0.4036842
H	-3.4876403	2.3095314	1.1749369
H	-0.1958608	-1.0523846	2.2551275
H	1.0016007	-0.2297104	1.2536483
H	-0.7964462	5.9530810	0.7631558
H	0.2841627	7.3024980	-1.0754419
H	-0.8789890	6.1435409	-1.7288307
C	0.0715788	6.2420803	-1.2041481
C	-0.0157581	5.5378010	0.1264894
H	2.1896849	4.8443425	4.4471378
O	1.2597353	5.7287333	0.8033278
C	1.4258277	5.0849499	1.9506660
O	0.6392360	4.2898639	2.4274472
H	-0.1890815	4.4695216	0.0140857
O	2.5816628	5.4350943	2.4847148
C	2.9594631	4.7278341	3.6883287
H	0.8473207	5.7991954	-1.8279611
H	3.8871564	5.1902042	4.0070718

```
H      3.1029443    3.6737852    3.4656898
H      3.4503697   -0.0302539    6.6168000
H      1.5652151   -1.5003001    6.2274569
O      1.3623053   -0.2709051    4.5832919
C      2.5022160    0.4820550    6.4625036
C      2.0052107    0.8621082    4.2750284
C      1.3758933   -0.4524691    6.0159161
O      2.0136677    1.3596249    3.1800615
O      2.6471000    1.3697875    5.3324343
H      2.2535571    1.0778660    7.3353863
H      0.4020246   -0.1548835    6.4002754
H     -3.8579141    3.6354412    5.7293916
H     -3.4701565    1.7672715    7.2099409
O     -1.8253714    1.5313824    5.9857828
C     -3.6925850    2.6879103    5.2193707
C     -1.4919036    2.2192723    4.8785268
C     -3.2578204    1.5648264    6.1645093
O     -0.3780121    2.2672307    4.4390208
O     -2.5524737    2.8375437    4.3472467
H     -4.5580333    2.4369921    4.6135081
H     -3.6592382    0.5923672    5.8842080
Li     0.3951987    2.3461717    2.4505035
O      2.1969111    3.1360619   -1.4237476
H      3.3879831    4.5757406   -2.2968784
C      3.5079120    3.6800801   -1.6949993
H      4.0775669    2.9339412   -2.2460225
O      1.1586817    2.2487548    0.3589547
C      2.1131021    2.7851975   -0.1343566
C      4.0613370    3.9512603   -0.2944394
O      3.2158044    3.1334432    0.5446777
H      3.9425710    4.9861439    0.0202337
H      5.0894016    3.6298007   -0.1600467

SCF energy GEOOPT =  -1800.482113463 H
ZPE =    1259. kJ/mol
FREEH energy =  1363.43 kJ/mol
FREEH entropy =   1.11455 kJ/mol/K

$vibrational spectrum
#  mode     symmetry     wave number   IR intensity    selection rules
#                         cm**(-1)        km/mol          IR     RAMAN
     1                      -0.00         0.00000         -       -
     2                      -0.00         0.00000         -       -
     3                      -0.00         0.00000         -       -
     4                      -0.00         0.00000         -       -
     5                       0.00         0.00000         -       -
     6                       0.00         0.00000         -       -
     7         a             8.88         2.38974        YES     YES
     8         a            13.04         0.90579        YES     YES
     9         a            17.46         1.04439        YES     YES
    10         a            19.36         1.55096        YES     YES
    11         a            21.95         0.28867        YES     YES
    12         a            23.51         1.09116        YES     YES
    13         a            27.88         0.39664        YES     YES
    14         a            31.89         0.12866        YES     YES
    15         a            32.91         3.27518        YES     YES
    16         a            35.19         1.29965        YES     YES
    17         a            40.08         0.42138        YES     YES
    18         a            44.96         0.41100        YES     YES
    19         a            50.41         0.03004        YES     YES
    20         a            54.73         1.38753        YES     YES
    21         a            60.96         0.47749        YES     YES
    22         a            62.19         2.28736        YES     YES
```

23	a	67.66	0.24052	YES	YES
24	a	70.09	4.31760	YES	YES
25	a	79.32	2.75094	YES	YES
26	a	82.27	3.06838	YES	YES
27	a	89.14	6.17769	YES	YES
28	a	94.91	0.05136	YES	YES
29	a	102.58	4.54404	YES	YES
30	a	103.85	5.00065	YES	YES
31	a	122.01	1.84691	YES	YES
32	a	124.67	1.89347	YES	YES
33	a	126.99	10.89405	YES	YES
34	a	131.32	1.95974	YES	YES
35	a	146.20	1.51667	YES	YES
36	a	150.37	0.61464	YES	YES
37	a	154.22	0.57267	YES	YES
38	a	161.64	2.39307	YES	YES
39	a	170.96	2.85495	YES	YES
40	a	172.69	2.18401	YES	YES
41	a	186.01	5.30256	YES	YES
42	a	188.11	3.10095	YES	YES
43	a	201.70	1.12856	YES	YES
44	a	216.44	1.87769	YES	YES
45	a	223.18	14.27386	YES	YES
46	a	241.47	25.31982	YES	YES
47	a	255.65	119.99191	YES	YES
48	a	263.30	3.19821	YES	YES
49	a	265.42	7.23993	YES	YES
50	a	322.24	12.27108	YES	YES
51	a	324.57	1.31817	YES	YES
52	a	370.63	2.13579	YES	YES
53	a	372.44	7.67982	YES	YES
54	a	386.73	152.91617	YES	YES
55	a	448.65	126.40353	YES	YES
56	a	516.62	10.06001	YES	YES
57	a	522.92	20.25775	YES	YES
58	a	527.57	10.58392	YES	YES
59	a	531.60	2.77911	YES	YES
60	a	532.42	6.60519	YES	YES
61	a	713.06	1.70244	YES	YES
62	a	713.42	2.06284	YES	YES
63	a	715.87	2.00502	YES	YES
64	a	719.05	0.64125	YES	YES
65	a	721.70	0.38602	YES	YES
66	a	733.85	34.62302	YES	YES
67	a	734.85	14.54727	YES	YES
68	a	735.86	16.51566	YES	YES
69	a	781.52	17.60399	YES	YES
70	a	785.84	30.87265	YES	YES
71	a	788.13	70.45297	YES	YES
72	a	809.98	35.11081	YES	YES
73	a	811.64	25.34649	YES	YES
74	a	825.61	6.10560	YES	YES
75	a	827.22	8.56784	YES	YES
76	a	880.46	0.85475	YES	YES
77	a	882.24	0.77531	YES	YES
78	a	884.48	1.32062	YES	YES
79	a	887.50	18.46150	YES	YES
80	a	889.30	39.33445	YES	YES
81	a	910.17	5.65407	YES	YES
82	a	911.95	6.81903	YES	YES
83	a	914.47	5.96261	YES	YES
84	a	957.83	37.57096	YES	YES
85	a	969.37	24.58094	YES	YES

86	a	976.49	11.88986	YES	YES
87	a	977.01	29.31834	YES	YES
88	a	977.85	12.64640	YES	YES
89	a	1024.68	30.66261	YES	YES
90	a	1027.62	103.32027	YES	YES
91	a	1035.63	5.45932	YES	YES
92	a	1037.35	4.56854	YES	YES
93	a	1038.78	5.07498	YES	YES
94	a	1093.29	203.19495	YES	YES
95	a	1094.98	237.34050	YES	YES
96	a	1098.50	178.41470	YES	YES
97	a	1133.70	5.41606	YES	YES
98	a	1136.85	5.04318	YES	YES
99	a	1140.93	2.64768	YES	YES
100	a	1150.32	0.61178	YES	YES
101	a	1155.35	1.66200	YES	YES
102	a	1156.63	0.49793	YES	YES
103	a	1157.43	0.97134	YES	YES
104	a	1175.88	4.18518	YES	YES
105	a	1178.58	97.58286	YES	YES
106	a	1180.19	8.71929	YES	YES
107	a	1181.11	117.52700	YES	YES
108	a	1182.87	163.21078	YES	YES
109	a	1187.22	12.21013	YES	YES
110	a	1188.18	194.63358	YES	YES
111	a	1227.56	10.91678	YES	YES
112	a	1229.61	4.43519	YES	YES
113	a	1241.57	9.38520	YES	YES
114	a	1243.46	11.33009	YES	YES
115	a	1244.36	6.53658	YES	YES
116	a	1252.04	16.55532	YES	YES
117	a	1252.45	10.85250	YES	YES
118	a	1254.83	18.91667	YES	YES
119	a	1306.27	16.42239	YES	YES
120	a	1311.37	1.17674	YES	YES
121	a	1327.97	531.89251	YES	YES
122	a	1346.21	838.55237	YES	YES
123	a	1393.53	0.05002	YES	YES
124	a	1394.41	1.73844	YES	YES
125	a	1395.12	0.09967	YES	YES
126	a	1413.39	44.12316	YES	YES
127	a	1416.03	59.66819	YES	YES
128	a	1424.71	34.39927	YES	YES
129	a	1425.51	78.73283	YES	YES
130	a	1428.24	63.48514	YES	YES
131	a	1433.73	21.05344	YES	YES
132	a	1439.06	30.43854	YES	YES
133	a	1486.45	4.46923	YES	YES
134	a	1486.55	55.44162	YES	YES
135	a	1487.13	2.74873	YES	YES
136	a	1488.07	39.69750	YES	YES
137	a	1491.46	74.07220	YES	YES
138	a	1494.85	33.75971	YES	YES
139	a	1497.72	6.42900	YES	YES
140	a	1498.88	7.71366	YES	YES
141	a	1502.01	5.32285	YES	YES
142	a	1503.76	12.11345	YES	YES
143	a	1521.58	30.35226	YES	YES
144	a	1523.50	9.08396	YES	YES
145	a	1524.74	15.47583	YES	YES
146	a	1525.62	11.53601	YES	YES
147	a	1527.16	20.88087	YES	YES
148	a	1531.36	0.72632	YES	YES

149	a	1532.90	4.51988	YES	YES
150	a	1533.87	0.25881	YES	YES
151	a	1731.19	513.96949	YES	YES
152	a	1746.80	269.07433	YES	YES
153	a	1825.73	1045.31598	YES	YES
154	a	1840.05	1107.45114	YES	YES
155	a	1856.46	299.09959	YES	YES
156	a	3045.44	11.31478	YES	YES
157	a	3047.59	9.11705	YES	YES
158	a	3059.59	21.82731	YES	YES
159	a	3065.23	14.61628	YES	YES
160	a	3068.92	15.68058	YES	YES
161	a	3072.87	11.03878	YES	YES
162	a	3078.13	21.86984	YES	YES
163	a	3079.68	24.16564	YES	YES
164	a	3080.61	14.29003	YES	YES
165	a	3081.56	21.29884	YES	YES
166	a	3084.23	11.66195	YES	YES
167	a	3087.96	9.82372	YES	YES
168	a	3106.96	1.67928	YES	YES
169	a	3108.76	4.03267	YES	YES
170	a	3113.18	16.30018	YES	YES
171	a	3114.66	15.70920	YES	YES
172	a	3130.86	22.74550	YES	YES
173	a	3131.49	16.88304	YES	YES
174	a	3135.69	18.90614	YES	YES
175	a	3136.90	4.35653	YES	YES
176	a	3139.92	4.40503	YES	YES
177	a	3140.47	3.90663	YES	YES
178	a	3151.55	8.38347	YES	YES
179	a	3152.37	7.01283	YES	YES
180	a	3154.78	7.52655	YES	YES
181	a	3156.12	7.05705	YES	YES
182	a	3169.30	8.29486	YES	YES
183	a	3175.46	5.62489	YES	YES

$end

Total COSMO energy + OC corr. = -1800.5466450699 H

[(EC)Li(PF_6)]

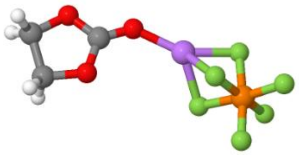

Method: (RI-)B3LYP(D3BJ)/def2-TZVPP
Symmetry: c1

Cartesian coordinates in Ångström:

P	-0.2225174	-0.1380783	0.1263209
F	0.4146374	-1.3213980	-0.8806593
F	-0.7639337	1.0930082	0.9584945
F	-1.5748619	-0.9510264	0.2480213
F	1.2099430	0.6823836	-0.1861321
F	-0.7591642	0.5698794	-1.2992781
F	0.4342702	-0.8358318	1.3848450

```
Li    0.9904130    0.1369604   -2.0378377
H     1.2127797   -0.6183495   -7.6435721
H    -0.4121644   -2.1204683   -6.6697020
O     0.4415487   -1.4391897   -4.9216113
C     1.6281647   -1.3168420   -6.9194709
C     1.4731892   -0.6076416   -4.7692736
C     0.5586715   -2.1311420   -6.1839632
O     1.6904246    0.0215609   -3.7640210
O     2.2392471   -0.5499227   -5.8578791
H     2.3973564   -1.9243126   -7.3864612
H     0.8633362   -3.1558696   -5.9798202

SCF energy GEOOPT =  -1290.693234206 H
ZPE =   256.0 kJ/mol
FREEH energy =   293.65 kJ/mol
FREEH entropy =   0.54516 kJ/mol/K

$vibrational spectrum
#  mode     symmetry     wave number   IR intensity    selection rules
#                         cm**(-1)        km/mol         IR     RAMAN
     1                      -0.00          0.00000        -       -
     2                      -0.00          0.00000        -       -
     3                      -0.00          0.00000        -       -
     4                       0.00          0.00000        -       -
     5                       0.00          0.00000        -       -
     6                       0.00          0.00000        -       -
     7         a            10.17          2.04266       YES     YES
     8         a            13.04          1.38347       YES     YES
     9         a            16.20          1.09560       YES     YES
    10         a            63.94          3.20118       YES     YES
    11         a            76.85          5.30459       YES     YES
    12         a           118.67          0.33790       YES     YES
    13         a           130.29          2.58450       YES     YES
    14         a           196.17         19.24276       YES     YES
    15         a           223.30          4.84145       YES     YES
    16         a           229.02         19.54289       YES     YES
    17         a           280.80          0.01712       YES     YES
    18         a           316.90          0.03857       YES     YES
    19         a           318.36          0.16664       YES     YES
    20         a           453.75          2.07877       YES     YES
    21         a           453.82          2.12263       YES     YES
    22         a           462.60          8.63488       YES     YES
    23         a           522.62          7.81561       YES     YES
    24         a           524.14          1.99439       YES     YES
    25         a           527.66         12.79215       YES     YES
    26         a           548.74         77.85397       YES     YES
    27         a           550.17         76.39127       YES     YES
    28         a           570.21        103.48085       YES     YES
    29         a           616.60        300.26609       YES     YES
    30         a           690.18         53.17319       YES     YES
    31         a           721.15          2.93574       YES     YES
    32         a           754.18        103.58718       YES     YES
    33         a           785.64         22.48488       YES     YES
    34         a           877.84          3.45425       YES     YES
    35         a           885.40        381.92525       YES     YES
    36         a           886.87        386.01618       YES     YES
    37         a           890.04        328.51473       YES     YES
    38         a           918.96          9.66826       YES     YES
    39         a           979.18          6.87121       YES     YES
    40         a          1037.42          3.34020       YES     YES
    41         a          1107.53        158.52096       YES     YES
    42         a          1156.38          0.77946       YES     YES
    43         a          1205.21        224.56838       YES     YES
```

44	a	1241.59	19.32452	YES	YES
45	a	1250.77	11.72197	YES	YES
46	a	1392.33	0.04524	YES	YES
47	a	1434.71	66.36426	YES	YES
48	a	1526.28	14.90652	YES	YES
49	a	1534.19	0.34597	YES	YES
50	a	1834.74	844.27138	YES	YES
51	a	3081.88	19.57411	YES	YES
52	a	3084.18	13.30942	YES	YES
53	a	3139.68	3.61317	YES	YES
54	a	3154.77	7.98503	YES	YES

$end

Total COSMO energy + OC corr. = -1290.7220424801 H

[(EMC)Li(PF_6)]

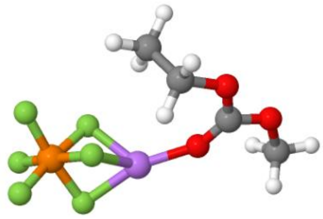

Method: (RI-)B3LYP(D3BJ)/def2-TZVPP
Symmetry: c1

Cartesian coordinates in Ångström:

P	0.9122579	0.1707430	-0.2479163
F	0.8060999	0.9288911	-1.7435290
F	0.8811024	-0.6454092	1.1045442
F	2.4820129	0.0413923	-0.4164481
F	-0.7643349	0.2505357	-0.2625134
F	0.6967702	-1.2500619	-1.1229296
F	0.9945536	1.5736493	0.4728652
Li	-0.6772478	-0.2778002	-2.1363431
O	-1.1569497	-0.8076610	-3.8112417
H	0.7022628	-2.3756670	-3.2752876
H	-3.5394384	-0.8979962	-4.9829073
H	1.6663934	-0.1151647	-3.8768051
H	2.9176515	-1.2567491	-3.4294402
C	-0.9119519	-1.2795489	-4.9150654
C	1.1820304	-2.1956112	-4.2333095
H	-2.7060718	0.6040354	-5.4526892
C	-2.9326957	-0.4182316	-5.7477506
C	2.1541215	-1.0404366	-4.1769057
O	0.1489083	-1.9729219	-5.2455825
H	1.6609149	-3.1116940	-4.5677264
O	-1.7140628	-1.1591495	-5.9562044
H	-3.4407545	-0.4356764	-6.7056465
H	2.6386279	-0.8940572	-5.1417281

SCF energy GEOOPT = -1331.214229689 H
ZPE = 384.7 kJ/mol
FREEH energy = 430.04 kJ/mol
FREEH entropy = 0.57413 kJ/mol/K

$vibrational spectrum

# mode	symmetry	wave number	IR intensity	selection rules	
#		cm**(-1)	km/mol	IR	RAMAN
1		-0.00	0.00000	-	-
2		0.00	0.00000	-	-
3		0.00	0.00000	-	-

4		0.00	0.00000	-	-
5		0.00	0.00000	-	-
6		0.00	0.00000	-	-
7	a	21.28	0.64233	YES	YES
8	a	36.69	1.93915	YES	YES
9	a	45.82	1.72609	YES	YES
10	a	74.37	0.71537	YES	YES
11	a	78.70	0.55523	YES	YES
12	a	100.02	2.04445	YES	YES
13	a	109.74	4.05525	YES	YES
14	a	119.06	0.99426	YES	YES
15	a	139.96	1.12483	YES	YES
16	a	176.44	2.27125	YES	YES
17	a	200.98	1.63529	YES	YES
18	a	213.07	17.32290	YES	YES
19	a	255.15	24.45104	YES	YES
20	a	282.96	0.10322	YES	YES
21	a	286.42	10.23497	YES	YES
22	a	315.34	0.23185	YES	YES
23	a	318.25	1.14257	YES	YES
24	a	354.46	8.30638	YES	YES
25	a	398.33	6.78839	YES	YES
26	a	454.10	1.90135	YES	YES
27	a	454.93	3.62266	YES	YES
28	a	464.91	4.86425	YES	YES
29	a	522.34	8.46983	YES	YES
30	a	523.98	3.38668	YES	YES
31	a	528.73	14.41556	YES	YES
32	a	545.65	67.16400	YES	YES
33	a	548.00	76.34579	YES	YES
34	a	568.98	90.00577	YES	YES
35	a	626.06	318.42865	YES	YES
36	a	688.87	56.83577	YES	YES
37	a	695.56	1.03521	YES	YES
38	a	807.67	23.15385	YES	YES
39	a	822.12	5.43276	YES	YES
40	a	874.84	11.21678	YES	YES
41	a	883.94	368.55667	YES	YES
42	a	888.90	358.35751	YES	YES
43	a	890.03	376.37418	YES	YES
44	a	967.63	13.86319	YES	YES
45	a	1004.17	96.25546	YES	YES
46	a	1111.85	17.18566	YES	YES
47	a	1142.54	6.66779	YES	YES
48	a	1178.69	1.12170	YES	YES
49	a	1199.85	8.41523	YES	YES
50	a	1232.73	1.45248	YES	YES
51	a	1325.17	13.40835	YES	YES
52	a	1354.00	615.52428	YES	YES
53	a	1418.61	161.15812	YES	YES
54	a	1427.63	21.88114	YES	YES
55	a	1486.69	25.58440	YES	YES
56	a	1489.84	113.89990	YES	YES
57	a	1491.58	10.71135	YES	YES
58	a	1498.69	5.56326	YES	YES
59	a	1501.84	5.17270	YES	YES
60	a	1521.65	81.32640	YES	YES
61	a	1727.29	422.35712	YES	YES
62	a	3050.92	7.12480	YES	YES
63	a	3061.33	20.75137	YES	YES
64	a	3096.99	12.35357	YES	YES
65	a	3116.64	10.79038	YES	YES
66	a	3127.90	6.26572	YES	YES

67	a	3137.45	12.86349	YES	YES
68	a	3154.42	11.70142	YES	YES
69	a	3172.37	7.96336	YES	YES

$end

Total COSMO energy + OC corr. = -1331.2353030846 H

(EC)$_2$Li[PF$_6$]

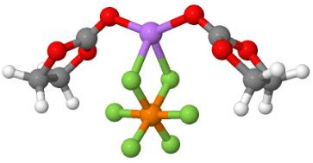

Method: (RI-)B3LYP(D3BJ)/def2-TZVPP
Symmetry: c2

Cartesian coordinates in Ångström:

P	0.0000000	0.0000000	1.3170290
F	-0.6511235	-1.4871677	1.2677562
F	0.6511235	1.4871677	1.2677562
F	-1.0624817	0.4638052	2.4055435
F	1.0349974	-0.4545495	0.0932790
F	-1.0349974	0.4545495	0.0932790
F	1.0624817	-0.4638052	2.4055435
Li	0.0000000	-0.0000000	-1.5310756
H	-1.7265947	-5.1754936	-0.2897073
H	-3.7033845	-3.8007542	-0.1951115
O	-2.5291873	-2.2769234	-0.9233440
C	-1.5353864	-4.1593544	0.0470935
C	-1.2882107	-2.4121639	-1.3842008
C	-2.7933279	-3.2750303	0.0822759
O	-0.7618917	-1.6277325	-2.1379800
O	-0.6964745	-3.5145537	-0.9321336
H	-0.9995398	-4.1659564	0.9917144
H	-2.9206847	-2.7637433	1.0319648
H	2.9206847	2.7637433	1.0319648
H	0.9995398	4.1659564	0.9917144
O	0.6964745	3.5145537	-0.9321336
C	2.7933279	3.2750303	0.0822759
C	1.2882107	2.4121639	-1.3842008
C	1.5353864	4.1593544	0.0470935
O	0.7618917	1.6277325	-2.1379800
O	2.5291873	2.2769234	-0.9233440
H	3.7033845	3.8007542	-0.1951115
H	1.7265947	5.1754936	-0.2897073

SCF energy GEOOPT = -1633.119493529 H
ZPE = 457.6 kJ/mol
FREEH energy = 512.21 kJ/mol
FREEH entropy = 0.67068 kJ/mol/K

$vibrational spectrum

# mode	symmetry	wave number	IR intensity	selection rules	
#		cm**(-1)	km/mol	IR	RAMAN
1		-0.00	0.00000	-	-
2		-0.00	0.00000	-	-
3		-0.00	0.00000	-	-
4		0.00	0.00000	-	-

5		0.00	0.00000	-	-
6		0.00	0.00000	-	-
7	b	8.82	7.57922	YES	YES
8	a	15.17	0.01465	YES	YES
9	a	47.31	0.10207	YES	YES
10	b	52.94	4.96359	YES	YES
11	a	54.89	3.54840	YES	YES
12	b	61.25	0.13567	YES	YES
13	a	65.26	0.07418	YES	YES
14	a	77.87	2.67824	YES	YES
15	b	81.86	1.59713	YES	YES
16	a	83.60	0.01792	YES	YES
17	b	84.45	9.46231	YES	YES
18	b	88.25	7.16198	YES	YES
19	b	114.72	0.68948	YES	YES
20	a	162.53	1.77193	YES	YES
21	b	206.26	18.90751	YES	YES
22	b	240.47	14.32924	YES	YES
23	a	251.24	5.65453	YES	YES
24	a	300.10	0.03936	YES	YES
25	b	304.21	0.39528	YES	YES
26	a	314.29	2.32867	YES	YES
27	a	376.18	111.98910	YES	YES
28	a	454.40	0.00437	YES	YES
29	b	459.61	0.04481	YES	YES
30	a	474.00	7.28750	YES	YES
31	b	518.23	22.18503	YES	YES
32	a	524.00	0.28494	YES	YES
33	b	530.75	28.85969	YES	YES
34	b	536.11	21.41792	YES	YES
35	a	551.47	6.79413	YES	YES
36	b	553.55	149.34578	YES	YES
37	b	555.26	60.91336	YES	YES
38	a	559.99	13.35218	YES	YES
39	a	710.61	39.65043	YES	YES
40	a	738.03	20.11953	YES	YES
41	b	743.81	26.18806	YES	YES
42	a	756.56	0.04049	YES	YES
43	b	756.62	5.10071	YES	YES
44	b	790.38	226.72348	YES	YES
45	a	795.07	58.94520	YES	YES
46	b	845.70	419.48428	YES	YES
47	a	857.49	1.58614	YES	YES
48	b	858.40	43.74770	YES	YES
49	a	873.62	260.18475	YES	YES
50	b	873.73	294.43776	YES	YES
51	a	917.28	12.99757	YES	YES
52	b	917.92	10.82379	YES	YES
53	b	986.26	10.74921	YES	YES
54	a	986.50	7.46406	YES	YES
55	a	1044.66	0.03227	YES	YES
56	b	1044.92	4.45214	YES	YES
57	b	1111.97	195.11762	YES	YES
58	a	1113.48	158.25479	YES	YES
59	a	1156.54	0.02162	YES	YES
60	b	1156.55	0.10332	YES	YES
61	a	1210.53	0.06538	YES	YES
62	b	1212.63	450.65205	YES	YES
63	b	1245.81	3.18927	YES	YES
64	a	1245.83	1.78131	YES	YES
65	a	1255.19	0.16942	YES	YES
66	b	1255.37	11.57826	YES	YES
67	b	1393.51	0.39205	YES	YES

68	a	1393.59	0.88755	YES	YES
69	a	1440.61	0.04770	YES	YES
70	b	1441.32	122.35528	YES	YES
71	a	1521.62	0.02037	YES	YES
72	b	1521.67	32.83552	YES	YES
73	b	1531.42	0.00979	YES	YES
74	a	1531.88	0.02438	YES	YES
75	b	1804.36	1086.65671	YES	YES
76	a	1823.48	462.27670	YES	YES
77	b	3085.85	32.64228	YES	YES
78	a	3085.87	0.70957	YES	YES
79	b	3091.38	60.28439	YES	YES
80	a	3091.50	8.65840	YES	YES
81	a	3135.50	0.29717	YES	YES
82	b	3135.50	0.00468	YES	YES
83	b	3154.31	13.15132	YES	YES
84	a	3154.35	10.57326	YES	YES

$end

Total COSMO energy + OC corr. = -1633.1470086519 H

(EMC)$_2$Li[PF$_6$]

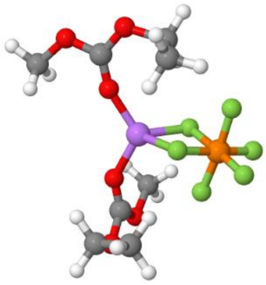

Method: (RI-)B3LYP(D3BJ)/def2-TZVPP
Symmetry: c1

Cartesian coordinates in Ångström:

P	0.8621156	-1.5394948	-1.1684078
F	-0.3138150	-0.4663785	-1.7229359
F	1.9455420	-2.6236199	-0.7568979
F	1.9790810	-0.6031572	-1.8448979
F	-0.3373762	-2.4667271	-0.6025699
F	0.6574272	-2.2963667	-2.6633290
F	0.9529630	-0.7470933	0.2033557
Li	-0.9607009	-1.4294641	-3.2638176
O	-0.8959402	-0.6053757	-4.9245905
H	1.4596419	-0.2467998	-4.2185866
H	-2.1891704	-1.6133170	-7.0118012
H	0.0099784	1.6312780	-3.3855545
H	1.6749940	1.8391820	-2.8748649
C	-0.4585369	0.0376924	-5.8654814
C	1.4835898	0.7337566	-4.6832201
H	-3.0431102	-0.1765364	-6.4059311
C	-2.3356001	-0.5435813	-7.1465651
C	1.0196698	1.8225504	-3.7446462
O	0.6690218	0.7053940	-5.8993506
H	2.4848930	0.9236685	-5.0597222
O	-1.0815018	0.1521869	-7.0295783
H	-2.6865928	-0.3293948	-8.1503938

```
H     1.0510653    2.7961386   -4.2335642
H    -1.4507086   -5.9587793   -2.3227736
H    -1.7747972   -4.7889261   -3.6322633
C    -1.5288417   -4.9076810   -2.5787087
H    -0.6054529   -4.3857977   -2.3441927
O    -2.6005465   -4.4024556   -1.7567506
O    -2.4776907   -2.4034350   -2.7921106
C    -2.9369989   -3.1407289   -1.9322525
O    -3.8728188   -2.8096559   -1.0661917
C    -4.2957812   -1.4192622   -1.0294237
H    -4.2832620   -1.0254213   -2.0436403
H    -2.3896043   -0.5800893   -0.4457149
C    -3.4186435   -0.6153114   -0.0948836
H    -5.3231573   -1.4721529   -0.6770079
H    -3.4298256   -1.0439168    0.9066395
H    -3.7977291    0.4064725   -0.0356944

SCF energy GEOOPT =  -1714.152851451 H
ZPE =   714.0 kJ/mol
FREEH energy =   784.81 kJ/mol
FREEH entropy =   0.78286 kJ/mol/K

$vibrational spectrum
#  mode     symmetry     wave number   IR intensity    selection rules
#                         cm**(-1)        km/mol          IR     RAMAN
     1                      -0.00         0.00000         -        -
     2                      -0.00         0.00000         -        -
     3                      -0.00         0.00000         -        -
     4                       0.00         0.00000         -        -
     5                       0.00         0.00000         -        -
     6                       0.00         0.00000         -        -
     7        a             16.31         0.07749        YES      YES
     8        a             19.75         0.03634        YES      YES
     9        a             35.65         1.00236        YES      YES
    10        a             42.80         0.33860        YES      YES
    11        a             48.42         0.68950        YES      YES
    12        a             52.42         0.35186        YES      YES
    13        a             61.66         0.52415        YES      YES
    14        a             65.63         0.21319        YES      YES
    15        a             74.07         3.93523        YES      YES
    16        a             75.82         0.77648        YES      YES
    17        a             90.60         1.99962        YES      YES
    18        a            100.31         3.27105        YES      YES
    19        a            105.46         2.38236        YES      YES
    20        a            108.18         4.00386        YES      YES
    21        a            120.44         1.57111        YES      YES
    22        a            135.79         1.06233        YES      YES
    23        a            142.94         0.18938        YES      YES
    24        a            168.52         1.51581        YES      YES
    25        a            176.75         1.74652        YES      YES
    26        a            186.42         3.37630        YES      YES
    27        a            203.22         4.01014        YES      YES
    28        a            208.23         0.43579        YES      YES
    29        a            261.37         7.28751        YES      YES
    30        a            269.89         4.10564        YES      YES
    31        a            278.69         1.42671        YES      YES
    32        a            302.84         0.30358        YES      YES
    33        a            310.85         1.00190        YES      YES
    34        a            324.46         1.84614        YES      YES
    35        a            351.58        16.15369        YES      YES
    36        a            359.15        16.60874        YES      YES
    37        a            399.98         6.29055        YES      YES
    38        a            402.78         2.84608        YES      YES
```

39	a	439.64	59.45406	YES	YES
40	a	458.53	0.32530	YES	YES
41	a	461.96	1.39461	YES	YES
42	a	490.55	72.35929	YES	YES
43	a	508.05	16.25556	YES	YES
44	a	511.18	86.44743	YES	YES
45	a	537.64	15.26230	YES	YES
46	a	542.79	30.92162	YES	YES
47	a	552.61	48.40286	YES	YES
48	a	553.49	16.67414	YES	YES
49	a	556.60	37.68302	YES	YES
50	a	567.20	144.40492	YES	YES
51	a	690.21	0.62492	YES	YES
52	a	692.33	2.92112	YES	YES
53	a	704.18	65.30908	YES	YES
54	a	807.33	23.72549	YES	YES
55	a	807.75	52.15077	YES	YES
56	a	817.29	6.34932	YES	YES
57	a	821.19	7.45773	YES	YES
58	a	866.59	355.25725	YES	YES
59	a	870.86	266.29548	YES	YES
60	a	875.65	304.03349	YES	YES
61	a	879.34	133.76295	YES	YES
62	a	885.56	80.63914	YES	YES
63	a	959.59	14.43888	YES	YES
64	a	966.44	11.75375	YES	YES
65	a	1006.34	101.10850	YES	YES
66	a	1010.66	94.11017	YES	YES
67	a	1113.40	17.94876	YES	YES
68	a	1115.10	19.02107	YES	YES
69	a	1140.30	4.48052	YES	YES
70	a	1146.36	8.56143	YES	YES
71	a	1179.19	0.80161	YES	YES
72	a	1180.36	0.77136	YES	YES
73	a	1199.43	7.82575	YES	YES
74	a	1201.34	7.51728	YES	YES
75	a	1231.52	3.32722	YES	YES
76	a	1234.46	1.51274	YES	YES
77	a	1325.01	59.57032	YES	YES
78	a	1328.59	46.96504	YES	YES
79	a	1348.15	572.43501	YES	YES
80	a	1349.34	596.54794	YES	YES
81	a	1414.20	111.79713	YES	YES
82	a	1416.57	101.17219	YES	YES
83	a	1425.61	40.45188	YES	YES
84	a	1429.47	17.49751	YES	YES
85	a	1485.71	76.43839	YES	YES
86	a	1487.60	36.12351	YES	YES
87	a	1488.40	82.82116	YES	YES
88	a	1489.83	7.06240	YES	YES
89	a	1490.06	11.82300	YES	YES
90	a	1493.30	7.97642	YES	YES
91	a	1497.98	0.26217	YES	YES
92	a	1499.38	12.77913	YES	YES
93	a	1501.88	25.25889	YES	YES
94	a	1507.31	7.29513	YES	YES
95	a	1514.98	77.93779	YES	YES
96	a	1519.29	60.49820	YES	YES
97	a	1725.32	889.97835	YES	YES
98	a	1749.66	51.86020	YES	YES
99	a	3045.04	14.68627	YES	YES
100	a	3049.60	12.04326	YES	YES
101	a	3058.85	22.75878	YES	YES

102	a	3063.82	28.13348	YES	YES
103	a	3077.84	23.72970	YES	YES
104	a	3101.49	11.21720	YES	YES
105	a	3107.27	17.79146	YES	YES
106	a	3117.13	13.24068	YES	YES
107	a	3123.27	9.50367	YES	YES
108	a	3128.46	12.87596	YES	YES
109	a	3132.73	14.89064	YES	YES
110	a	3146.84	7.07667	YES	YES
111	a	3148.52	10.53717	YES	YES
112	a	3163.43	7.27898	YES	YES
113	a	3167.32	11.33491	YES	YES
114	a	3174.25	4.08481	YES	YES

$end

Total COSMO energy + OC corr. = -1714.1723688031 H

(EC)(EMC)Li[PF_6]

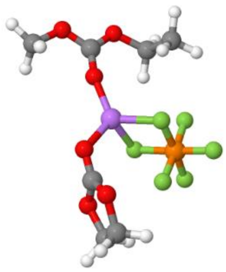

Method: (RI-)B3LYP(D3BJ)/def2-TZVPP
Symmetry: c1

Cartesian coordinates in Ångström:

P	0.2188879	-2.5739055	-1.4004338
F	-0.3771349	-1.0668737	-1.8279635
F	0.7068801	-4.0542455	-1.0853420
F	1.5471368	-2.2609932	-2.2507477
F	-1.1859592	-2.8481713	-0.6244328
F	-0.5159716	-3.1036410	-2.8106327
F	0.8585723	-1.9622485	-0.0818289
Li	-1.4403522	-1.5000109	-3.3947844
O	-0.9916401	-0.6702189	-4.9764878
H	1.3166867	-1.4220372	-4.5068903
H	-2.7255630	-0.7710431	-6.9790773
H	0.8717530	0.7592181	-3.3479447
H	2.4937171	0.1841439	-3.0113669
C	-0.3848535	-0.1530179	-5.9000745
C	1.7344432	-0.4926496	-4.8812196
H	-2.8403735	0.8029775	-6.1609566
C	-2.4091585	0.2695861	-7.0054627
C	1.8410156	0.5568817	-3.7999555
O	0.9159128	-0.0183791	-5.9981580
H	2.6955121	-0.6942517	-5.3464146
O	-0.9720946	0.3568022	-6.9721458
H	-2.6996569	0.7345851	-7.9414472
H	2.2562050	1.4831670	-4.1969240
H	-4.5404831	-5.1639761	-0.8654071
H	-5.2459206	-3.2284197	0.3881216
O	-4.0170866	-2.0725352	-0.7903797
C	-3.7730976	-4.4005381	-0.7705230
C	-3.5855220	-2.4882825	-1.9786745

```
C    -4.2002029   -3.1999040    0.0895397
O    -3.2697807   -1.7452888   -2.8775053
O    -3.5576644   -3.8166181   -2.0704841
H    -2.8339975   -4.8381634   -0.4438319
H    -3.5627093   -3.0540288    0.9566656

SCF energy GEOOPT =  -1673.636063406 H
ZPE =   585.5 kJ/mol
FREEH energy =   648.42 kJ/mol
FREEH entropy =   0.73888 kJ/mol/K

$vibrational spectrum
#  mode     symmetry     wave number   IR intensity    selection rules
#                         cm**(-1)        km/mol          IR     RAMAN
     1                      -0.00         0.00000         -        -
     2                      -0.00         0.00000         -        -
     3                       0.00         0.00000         -        -
     4                       0.00         0.00000         -        -
     5                       0.00         0.00000         -        -
     6                       0.00         0.00000         -        -
     7         a             8.14         1.88671        YES      YES
     8         a            22.30         0.52111        YES      YES
     9         a            24.88         0.16327        YES      YES
    10         a            40.93         5.09709        YES      YES
    11         a            46.23         0.89149        YES      YES
    12         a            52.18         0.67577        YES      YES
    13         a            59.62         2.55673        YES      YES
    14         a            69.43         0.84131        YES      YES
    15         a            85.08         5.56066        YES      YES
    16         a            86.64         3.36926        YES      YES
    17         a            90.67         2.89530        YES      YES
    18         a           100.50         1.36938        YES      YES
    19         a           102.73         1.98888        YES      YES
    20         a           114.79         1.57122        YES      YES
    21         a           137.50         0.92887        YES      YES
    22         a           144.73         0.87271        YES      YES
    23         a           177.83         1.83116        YES      YES
    24         a           205.00         0.99258        YES      YES
    25         a           240.68        10.15031        YES      YES
    26         a           242.81        12.92635        YES      YES
    27         a           276.77         1.02518        YES      YES
    28         a           300.75         0.07288        YES      YES
    29         a           312.38         1.18331        YES      YES
    30         a           313.08         1.24591        YES      YES
    31         a           352.03        15.95720        YES      YES
    32         a           399.44         7.44031        YES      YES
    33         a           419.77        86.93320        YES      YES
    34         a           456.01         0.03034        YES      YES
    35         a           460.02         0.58117        YES      YES
    36         a           476.44        28.25390        YES      YES
    37         a           514.39        68.39326        YES      YES
    38         a           519.09        14.52470        YES      YES
    39         a           528.53         1.79707        YES      YES
    40         a           545.24        26.53063        YES      YES
    41         a           550.87        68.99048        YES      YES
    42         a           553.50        48.77619        YES      YES
    43         a           558.51        19.24655        YES      YES
    44         a           569.44       144.20762        YES      YES
    45         a           691.76         1.78849        YES      YES
    46         a           706.39        59.46838        YES      YES
    47         a           739.36        29.67059        YES      YES
    48         a           750.59         2.89058        YES      YES
    49         a           791.58       112.42701        YES      YES
```

50	a	807.11	20.23456	YES	YES
51	a	820.47	8.97437	YES	YES
52	a	860.40	87.55774	YES	YES
53	a	864.19	336.23841	YES	YES
54	a	867.24	334.63622	YES	YES
55	a	872.71	281.59222	YES	YES
56	a	879.27	85.53115	YES	YES
57	a	917.55	10.87038	YES	YES
58	a	967.83	10.77775	YES	YES
59	a	984.73	10.96767	YES	YES
60	a	1006.25	102.55950	YES	YES
61	a	1044.49	2.35527	YES	YES
62	a	1110.38	186.93555	YES	YES
63	a	1113.19	23.66198	YES	YES
64	a	1139.15	5.57837	YES	YES
65	a	1156.37	0.33324	YES	YES
66	a	1178.82	0.78273	YES	YES
67	a	1199.75	7.91934	YES	YES
68	a	1208.95	224.06952	YES	YES
69	a	1232.26	3.02393	YES	YES
70	a	1245.22	3.54663	YES	YES
71	a	1255.30	7.60460	YES	YES
72	a	1325.31	57.42236	YES	YES
73	a	1348.77	593.68155	YES	YES
74	a	1393.45	0.36493	YES	YES
75	a	1414.25	120.29853	YES	YES
76	a	1425.64	36.09859	YES	YES
77	a	1439.41	59.71639	YES	YES
78	a	1487.06	26.25946	YES	YES
79	a	1488.65	91.16308	YES	YES
80	a	1490.34	11.04333	YES	YES
81	a	1497.87	0.33731	YES	YES
82	a	1499.54	13.70976	YES	YES
83	a	1518.38	65.53467	YES	YES
84	a	1521.43	15.20042	YES	YES
85	a	1531.24	0.16618	YES	YES
86	a	1744.56	685.93066	YES	YES
87	a	1816.07	640.18023	YES	YES
88	a	3049.15	11.91021	YES	YES
89	a	3059.83	22.25073	YES	YES
90	a	3082.59	23.36130	YES	YES
91	a	3092.66	28.43967	YES	YES
92	a	3099.00	13.11483	YES	YES
93	a	3116.57	10.96268	YES	YES
94	a	3127.56	12.86563	YES	YES
95	a	3134.51	0.87946	YES	YES
96	a	3134.63	13.44500	YES	YES
97	a	3153.80	11.37275	YES	YES
98	a	3160.60	8.92808	YES	YES
99	a	3167.95	11.56508	YES	YES

$end

Total COSMO energy + OC corr. = -1673.6599369504 H

(EC)$_3$Li[PF$_6$]

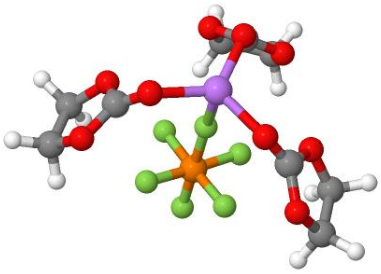

```
Method: (RI-)B3LYP(D3BJ)/def2-TZVPP
Symmetry: c1

Cartesian coordinates in Ångström:
H    -1.1311344   -4.8548992   -0.9271503
H    -0.7200101   -3.1328518   -1.1819861
C    -1.4025333   -3.9354600   -1.4425869
F    -1.5989809    1.2193621   -2.3277472
O    -1.2925740   -4.1693735   -2.8603385
F    -0.9057553   -0.9784877   -2.3905402
C    -2.8755302   -3.5416435   -1.2580920
P    -1.9472623   -0.0521987   -3.2285397
C    -2.4866615   -4.0442493   -3.4424754
F    -3.1394431   -0.4882287   -2.2167595
H    -3.4014370   -4.1552938   -0.5314047
F    -0.7700909    0.3213719   -4.2775705
H    -3.0028642   -2.4860018   -1.0382449
O    -2.6714854   -4.1535292   -4.6290346
Li   -2.6591709   -2.5742932   -5.7686660
O    -3.4514164   -3.7926466   -2.5567721
F    -3.0064510    0.8037872   -4.1053726
F    -2.3060703   -1.3814970   -4.1619420
O    -5.3354784   -1.1864498   -4.4588422
H    -6.9644495   -0.2844710   -3.5888890
H    -5.3640021    0.2644024   -3.0100800
C    -5.9499162   -0.0202320   -3.8782761
O    -4.2569535   -1.6862437   -6.3586363
C    -4.8942353   -0.9137743   -5.6859864
C    -5.8873290    1.0174676   -5.0115624
O    -5.2513993    0.3045692   -6.0913894
H    -6.8652659    1.3463375   -5.3552431
H    -5.2638922    1.8710169   -4.7623481
O     0.7302644   -0.7949105   -6.6432242
H     2.7294402   -0.5739728   -6.2117061
C     1.7750609   -0.4961868   -5.6960944
H     1.6190817    0.5160716   -5.3347579
C    -0.0301670   -1.7889513   -6.1846112
O    -1.0348678   -2.1747617   -6.7287090
C     1.5820566   -1.5534787   -4.5957591
O     0.4565935   -2.3194147   -5.0626372
H     2.4280627   -2.2288879   -4.4912469
H     1.3140372   -1.1212771   -3.6366280

SCF energy GEOOPT =  -1975.533714297 H
ZPE =   658.9 kJ/mol
FREEH energy =   730.60 kJ/mol
FREEH entropy =   0.80847 kJ/mol/K

$vibrational spectrum
#  mode     symmetry     wave number   IR intensity    selection rules
#                         cm**(-1)        km/mol          IR     RAMAN
```

1		0.00	0.00000	-	-
2		0.00	0.00000	-	-
3		0.00	0.00000	-	-
4		0.00	0.00000	-	-
5		0.00	0.00000	-	-
6		0.00	0.00000	-	-
7	a	14.58	0.44713	YES	YES
8	a	24.09	3.50222	YES	YES
9	a	27.85	2.72299	YES	YES
10	a	39.82	1.92096	YES	YES
11	a	44.02	0.12761	YES	YES
12	a	54.14	1.49196	YES	YES
13	a	57.94	2.12842	YES	YES
14	a	59.88	1.73825	YES	YES
15	a	66.47	1.03845	YES	YES
16	a	74.06	2.23477	YES	YES
17	a	76.06	0.68308	YES	YES
18	a	77.60	4.00758	YES	YES
19	a	85.54	5.54431	YES	YES
20	a	91.82	1.46341	YES	YES
21	a	93.64	5.98228	YES	YES
22	a	102.39	0.68033	YES	YES
23	a	105.40	3.64933	YES	YES
24	a	113.80	6.63482	YES	YES
25	a	119.49	2.91125	YES	YES
26	a	126.78	1.69042	YES	YES
27	a	165.90	9.82508	YES	YES
28	a	231.75	19.33371	YES	YES
29	a	234.27	27.05151	YES	YES
30	a	241.92	32.83100	YES	YES
31	a	265.37	45.39465	YES	YES
32	a	299.85	0.02739	YES	YES
33	a	304.17	0.67684	YES	YES
34	a	305.64	0.13311	YES	YES
35	a	435.69	96.68325	YES	YES
36	a	454.98	2.64302	YES	YES
37	a	456.37	3.12191	YES	YES
38	a	463.99	38.28264	YES	YES
39	a	464.54	99.12637	YES	YES
40	a	522.39	4.52883	YES	YES
41	a	532.10	10.19563	YES	YES
42	a	538.31	8.08258	YES	YES
43	a	545.44	17.39321	YES	YES
44	a	546.29	40.76729	YES	YES
45	a	547.33	43.96730	YES	YES
46	a	554.33	9.73800	YES	YES
47	a	560.14	1.41011	YES	YES
48	a	722.38	23.06187	YES	YES
49	a	737.15	22.11459	YES	YES
50	a	739.40	15.83304	YES	YES
51	a	740.85	16.77228	YES	YES
52	a	746.62	1.04891	YES	YES
53	a	758.11	3.52868	YES	YES
54	a	758.60	1.36153	YES	YES
55	a	785.43	96.14996	YES	YES
56	a	787.37	84.62762	YES	YES
57	a	792.17	96.98940	YES	YES
58	a	844.07	307.73209	YES	YES
59	a	847.41	334.44404	YES	YES
60	a	856.57	217.27958	YES	YES
61	a	860.10	31.03581	YES	YES
62	a	860.72	35.26350	YES	YES
63	a	869.99	13.97000	YES	YES

64	a	915.53	3.68514	YES	YES
65	a	916.28	15.11732	YES	YES
66	a	916.39	5.31866	YES	YES
67	a	984.64	5.16056	YES	YES
68	a	985.37	7.69412	YES	YES
69	a	986.82	16.22169	YES	YES
70	a	1043.27	3.90639	YES	YES
71	a	1046.80	2.97162	YES	YES
72	a	1048.51	4.91777	YES	YES
73	a	1106.14	83.70113	YES	YES
74	a	1110.50	173.30294	YES	YES
75	a	1114.57	257.50917	YES	YES
76	a	1159.07	0.00508	YES	YES
77	a	1159.41	0.01507	YES	YES
78	a	1160.98	0.25574	YES	YES
79	a	1196.91	217.76069	YES	YES
80	a	1209.25	35.77762	YES	YES
81	a	1209.94	471.39342	YES	YES
82	a	1246.79	2.04792	YES	YES
83	a	1247.09	1.31016	YES	YES
84	a	1248.64	3.60785	YES	YES
85	a	1254.66	2.06617	YES	YES
86	a	1255.31	12.75067	YES	YES
87	a	1258.28	7.46491	YES	YES
88	a	1393.02	1.01354	YES	YES
89	a	1393.43	1.03416	YES	YES
90	a	1395.64	1.04633	YES	YES
91	a	1435.74	43.50690	YES	YES
92	a	1439.77	104.91393	YES	YES
93	a	1440.16	33.90666	YES	YES
94	a	1521.56	10.97033	YES	YES
95	a	1522.88	14.01988	YES	YES
96	a	1523.14	14.46978	YES	YES
97	a	1532.85	0.74189	YES	YES
98	a	1533.60	0.20417	YES	YES
99	a	1535.58	1.19085	YES	YES
100	a	1808.42	252.90032	YES	YES
101	a	1812.08	813.36082	YES	YES
102	a	1846.45	939.39221	YES	YES
103	a	3077.74	28.85818	YES	YES
104	a	3083.08	13.81627	YES	YES
105	a	3083.75	13.11550	YES	YES
106	a	3088.33	57.73019	YES	YES
107	a	3089.07	37.98824	YES	YES
108	a	3093.61	38.39691	YES	YES
109	a	3135.90	2.27789	YES	YES
110	a	3138.00	3.00645	YES	YES
111	a	3142.81	1.40294	YES	YES
112	a	3154.91	7.76409	YES	YES
113	a	3157.32	5.92987	YES	YES
114	a	3159.63	3.30484	YES	YES

$end

Total COSMO energy + OC corr. = -1975.5656180536 H

(EMC)$_3$Li[PF$_6$]

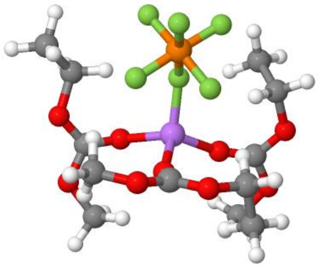

Method: (RI-)B3LYP(D3BJ)/def2-TZVPP
Symmetry: c1

Cartesian coordinates in Ångström:

```
F    -0.2480539    0.3255569    0.2767583
F    -0.6824741   -1.2819771   -1.3239992
P    -0.6829318    0.3454531   -1.2527203
F    -2.2615911    0.3023205   -0.8506286
F     0.8435513    0.3624091   -1.7602390
Li   -2.4379681   -0.6757988   -3.8002245
H     0.3682425   -1.4723719   -3.6445890
H    -3.0287742   -3.4186166   -6.5123441
F    -0.7398727    1.9507679   -1.2929668
O    -1.5847207   -1.5802512   -5.2300886
H     1.9817780   -1.9314813   -4.2040408
C     1.0531895   -1.4427790   -4.4846102
C    -0.7465434   -2.2311304   -5.8270085
F    -1.1711320    0.3397232   -2.8757855
O     0.5356140   -2.3005490   -5.5550359
C    -2.3924483   -3.0196865   -7.2996014
O    -1.0197708   -3.0031871   -6.8740418
H    -2.4136325   -3.6669771   -8.1700461
H    -2.7209691   -2.0154049   -7.5592494
C     1.2814389   -0.0334732   -4.9756789
H     1.7004374    0.5543379   -4.1604212
H     0.3438698    0.4360078   -5.2688743
H     1.9718656   -0.0213120   -5.8191007
H    -4.7482836    3.8445847   -1.0785875
H    -3.7895322    2.4817003   -0.4546739
C    -3.8351084    3.2640792   -1.2099208
H    -2.9763966    3.9179866   -1.0591218
H    -5.3617201   -1.6858873   -4.2740286
H    -7.0847017   -1.2883702   -4.5456012
C    -6.0642375   -0.9234951   -4.6035730
O    -6.0173548    0.2072888   -3.7157902
C    -4.8480374    0.8390907   -3.6467200
C    -3.7538902    2.6685744   -2.5914132
O    -4.9346803    1.8217805   -2.7811999
H    -3.7865978    3.4277595   -3.3730749
H    -5.8257359   -0.6164863   -5.6199147
H    -2.8641205    2.0641708   -2.7172202
O    -3.8657341    0.5314127   -4.3001471
H    -2.6942013   -5.6917860   -3.3793555
H    -2.9708832   -4.1529268   -4.2156976
C    -2.2249356   -4.7667755   -3.7137457
H    -1.4458968   -5.0200305   -4.4349846
H    -5.6985042   -1.4004001   -1.5095821
H    -5.5362376   -1.3798125    0.2667837
C    -5.0019593   -1.3421624   -0.6764204
O    -4.1381359   -2.4969952   -0.6616788
C    -3.3529376   -2.6581570   -1.7082749
```

```
C    -1.6101094   -4.0187819   -2.5504189
O    -2.5916490   -3.7196190   -1.5266409
H    -0.8727351   -4.6229870   -2.0286879
H    -4.4066487   -0.4372781   -0.7440326
H    -1.1424272   -3.0892505   -2.8581502
O    -3.3630825   -1.9500771   -2.7028700

SCF energy GEOOPT =  -2097.078582932 H
ZPE =   1042. kJ/mol
FREEH energy =  1138.93 kJ/mol
FREEH entropy =   0.98448 kJ/mol/K

$vibrational spectrum
#  mode     symmetry     wave number   IR intensity    selection rules
#                         cm**(-1)        km/mol          IR     RAMAN
     1                      -0.00         0.00000         -        -
     2                       0.00         0.00000         -        -
     3                       0.00         0.00000         -        -
     4                       0.00         0.00000         -        -
     5                       0.00         0.00000         -        -
     6                       0.00         0.00000         -        -
     7         a            13.96         0.29780        YES      YES
     8         a            19.58         0.09766        YES      YES
     9         a            28.60         0.02681        YES      YES
    10         a            33.94         0.42568        YES      YES
    11         a            40.03         0.10919        YES      YES
    12         a            43.59         1.28689        YES      YES
    13         a            46.76         0.08851        YES      YES
    14         a            51.55         0.54013        YES      YES
    15         a            56.85         0.81876        YES      YES
    16         a            60.65         0.05983        YES      YES
    17         a            63.81         0.33686        YES      YES
    18         a            67.38         1.32521        YES      YES
    19         a            73.69         0.29554        YES      YES
    20         a            76.06         1.91603        YES      YES
    21         a            81.91         0.10740        YES      YES
    22         a            88.13         2.48666        YES      YES
    23         a            93.58         0.64044        YES      YES
    24         a            99.98         1.90578        YES      YES
    25         a           103.35         2.49228        YES      YES
    26         a           109.25         2.80465        YES      YES
    27         a           119.73         1.44695        YES      YES
    28         a           128.43         8.56768        YES      YES
    29         a           136.10         0.19358        YES      YES
    30         a           142.37         1.16422        YES      YES
    31         a           155.11         0.89984        YES      YES
    32         a           161.77         1.19822        YES      YES
    33         a           176.19         2.47513        YES      YES
    34         a           179.63         2.34606        YES      YES
    35         a           184.05         2.25756        YES      YES
    36         a           187.52         3.92455        YES      YES
    37         a           190.07         2.86932        YES      YES
    38         a           204.74         2.79866        YES      YES
    39         a           215.95         2.99163        YES      YES
    40         a           263.99         1.16397        YES      YES
    41         a           271.76         1.81563        YES      YES
    42         a           280.09         0.82708        YES      YES
    43         a           294.57         0.18317        YES      YES
    44         a           303.27         1.73851        YES      YES
    45         a           304.62         1.28155        YES      YES
    46         a           333.82        13.26905        YES      YES
    47         a           344.28        14.54350        YES      YES
    48         a           350.51        24.20315        YES      YES
```

49	a	374.54	9.17841	YES	YES
50	a	391.58	24.71022	YES	YES
51	a	402.37	11.80420	YES	YES
52	a	408.16	78.50701	YES	YES
53	a	416.44	50.19010	YES	YES
54	a	450.90	1.58823	YES	YES
55	a	465.21	108.83505	YES	YES
56	a	466.40	2.82060	YES	YES
57	a	470.79	27.24446	YES	YES
58	a	514.49	44.93005	YES	YES
59	a	520.82	13.32310	YES	YES
60	a	538.48	28.63037	YES	YES
61	a	542.19	11.77840	YES	YES
62	a	544.99	41.25952	YES	YES
63	a	548.39	38.84079	YES	YES
64	a	563.69	14.62222	YES	YES
65	a	564.47	8.17560	YES	YES
66	a	691.19	0.65617	YES	YES
67	a	691.60	0.08554	YES	YES
68	a	713.20	0.24476	YES	YES
69	a	717.60	50.37117	YES	YES
70	a	803.66	32.98960	YES	YES
71	a	805.99	27.64386	YES	YES
72	a	810.11	33.35952	YES	YES
73	a	812.02	56.06291	YES	YES
74	a	816.76	7.97088	YES	YES
75	a	822.35	11.00894	YES	YES
76	a	855.43	360.73517	YES	YES
77	a	857.71	342.36503	YES	YES
78	a	863.63	318.66788	YES	YES
79	a	878.03	34.27806	YES	YES
80	a	880.94	52.73195	YES	YES
81	a	885.15	33.31326	YES	YES
82	a	957.77	19.30209	YES	YES
83	a	962.85	7.37342	YES	YES
84	a	964.10	23.03351	YES	YES
85	a	1005.75	79.20304	YES	YES
86	a	1017.54	88.57778	YES	YES
87	a	1018.66	115.54676	YES	YES
88	a	1112.20	9.02484	YES	YES
89	a	1114.94	27.17875	YES	YES
90	a	1133.18	4.00520	YES	YES
91	a	1137.86	2.93025	YES	YES
92	a	1140.77	1.07654	YES	YES
93	a	1149.61	14.86671	YES	YES
94	a	1177.72	0.96675	YES	YES
95	a	1181.04	0.67559	YES	YES
96	a	1184.43	0.25294	YES	YES
97	a	1186.91	4.86071	YES	YES
98	a	1196.91	7.21381	YES	YES
99	a	1199.92	15.58819	YES	YES
100	a	1228.03	16.84674	YES	YES
101	a	1229.52	5.72629	YES	YES
102	a	1233.41	0.89758	YES	YES
103	a	1305.50	118.80333	YES	YES
104	a	1323.68	110.09411	YES	YES
105	a	1330.03	35.00110	YES	YES
106	a	1337.58	252.41937	YES	YES
107	a	1342.17	877.09856	YES	YES
108	a	1344.41	515.16568	YES	YES
109	a	1406.51	51.71294	YES	YES
110	a	1411.13	21.28787	YES	YES
111	a	1411.56	138.23550	YES	YES

112	a	1424.40	36.94296	YES	YES
113	a	1427.17	44.82038	YES	YES
114	a	1431.32	19.20565	YES	YES
115	a	1484.62	10.82849	YES	YES
116	a	1484.98	41.16044	YES	YES
117	a	1486.19	68.23860	YES	YES
118	a	1486.92	95.10664	YES	YES
119	a	1488.13	14.18868	YES	YES
120	a	1488.77	1.28395	YES	YES
121	a	1489.89	25.98546	YES	YES
122	a	1490.73	33.19717	YES	YES
123	a	1492.53	14.68862	YES	YES
124	a	1497.11	2.21413	YES	YES
125	a	1498.30	5.78679	YES	YES
126	a	1500.07	12.26569	YES	YES
127	a	1503.36	6.61554	YES	YES
128	a	1506.24	8.77437	YES	YES
129	a	1510.15	4.46114	YES	YES
130	a	1512.73	64.98348	YES	YES
131	a	1517.86	47.77383	YES	YES
132	a	1523.52	18.99368	YES	YES
133	a	1722.64	306.77283	YES	YES
134	a	1735.35	878.72678	YES	YES
135	a	1764.77	89.55380	YES	YES
136	a	3040.62	11.91433	YES	YES
137	a	3047.30	16.19536	YES	YES
138	a	3048.49	14.98839	YES	YES
139	a	3057.92	14.08039	YES	YES
140	a	3058.89	38.17173	YES	YES
141	a	3064.93	25.22777	YES	YES
142	a	3073.18	27.09255	YES	YES
143	a	3096.58	2.16465	YES	YES
144	a	3105.88	14.10735	YES	YES
145	a	3106.49	13.00156	YES	YES
146	a	3113.30	15.77196	YES	YES
147	a	3117.12	13.50077	YES	YES
148	a	3119.88	27.11784	YES	YES
149	a	3127.51	15.76928	YES	YES
150	a	3130.91	10.26472	YES	YES
151	a	3131.93	14.10439	YES	YES
152	a	3134.37	10.23701	YES	YES
153	a	3157.26	9.69335	YES	YES
154	a	3160.72	10.70755	YES	YES
155	a	3161.34	10.47292	YES	YES
156	a	3164.97	11.72527	YES	YES
157	a	3174.50	5.40339	YES	YES
158	a	3179.29	2.95902	YES	YES
159	a	3185.00	0.34599	YES	YES

$end

Total COSMO energy + OC corr. = -2097.1034120238 H

$(EC)_2(EMC)Li[PF_6]$

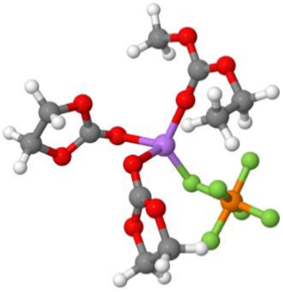

Method: (RI-)B3LYP(D3BJ)/def2-TZVPP
Symmetry: c1

Cartesian coordinates in Ångström:

H	-4.6253593	-3.1969446	-0.0733431
H	-3.0567310	-4.0374538	-0.2865485
C	-3.5998803	-3.1105725	-0.4301705
F	1.1394069	-3.4566351	-1.1574756
O	-3.6445363	-2.8381151	-1.8479482
F	-1.0146153	-4.2241435	-1.4194940
C	-2.8627605	-1.8893634	0.1250678
P	0.0965668	-3.5190969	-2.3669603
C	-3.2496609	-1.5891711	-2.0813710
F	-0.5193212	-2.0535544	-1.9319126
H	-3.3251604	-1.4562064	1.0068616
F	0.6156298	-4.9423258	-2.8575462
H	-1.8065630	-2.0846363	0.2912096
O	-3.1746616	-1.1055797	-3.1875914
Li	-1.7534534	-2.0338094	-4.4616953
H	0.9372044	-0.4713949	-3.2691888
O	-2.9673972	-0.9322868	-0.9558118
H	-0.5737955	-0.9143146	-7.7364903
F	1.1242500	-2.7633317	-3.3563135
O	-0.6862131	-0.6718550	-5.1105562
H	1.8232611	1.0430867	-3.0047458
C	0.8700569	0.6111455	-3.2982303
C	-0.0621904	0.3132859	-5.4538970
F	-1.0344046	-3.5189074	-3.6165845
O	0.7283813	1.0348619	-4.6925424
C	-0.9178623	0.1117262	-7.6235302
O	-0.0876217	0.8172889	-6.6820409
H	-0.8128207	0.6550155	-8.5569859
H	-1.9529590	0.1107383	-7.2892167
C	-0.2698463	1.1318630	-2.4533655
H	-0.1086934	0.8287970	-1.4186189
H	-1.2209539	0.7100658	-2.7720420
H	-0.3232967	2.2203746	-2.4949637
O	-4.2322717	-0.5223610	-5.9895399
H	-5.3297084	0.4460221	-4.5466252
H	-5.8650158	0.7121218	-6.2304777
C	-5.4948857	-0.0186427	-5.5175118
O	-3.1594959	-2.4915594	-5.8259044
C	-4.1634967	-1.8417481	-5.7356832
C	-6.3459851	-1.2865963	-5.4317233
O	-5.3567768	-2.3318551	-5.3779387
H	-6.9539891	-1.3438282	-4.5335794
H	-6.9666540	-1.4459139	-6.3128842

SCF energy GEOOPT = -2016.037884836 H
ZPE =   785.5 kJ/mol

FREEH energy =   865.87 kJ/mol
FREEH entropy =   0.88410 kJ/mol/K

$vibrational spectrum

# mode	symmetry	wave number cm**(-1)	IR intensity km/mol	selection rules IR	RAMAN
1		-0.00	0.00000	-	-
2		0.00	0.00000	-	-
3		0.00	0.00000	-	-
4		0.00	0.00000	-	-
5		0.00	0.00000	-	-
6		0.00	0.00000	-	-
7	a	10.20	0.24568	YES	YES
8	a	20.90	0.62792	YES	YES
9	a	27.19	2.74212	YES	YES
10	a	27.84	1.14485	YES	YES
11	a	32.26	0.73367	YES	YES
12	a	35.00	4.97942	YES	YES
13	a	40.45	3.39306	YES	YES
14	a	44.97	0.38054	YES	YES
15	a	52.75	2.07626	YES	YES
16	a	61.30	1.05149	YES	YES
17	a	61.81	0.67298	YES	YES
18	a	65.03	0.78629	YES	YES
19	a	80.58	1.45301	YES	YES
20	a	91.66	2.28431	YES	YES
21	a	94.36	4.77274	YES	YES
22	a	103.14	4.31538	YES	YES
23	a	103.98	2.84332	YES	YES
24	a	113.38	12.74567	YES	YES
25	a	121.04	1.44324	YES	YES
26	a	140.36	1.04923	YES	YES
27	a	149.08	0.88477	YES	YES
28	a	163.54	0.51451	YES	YES
29	a	181.87	1.74831	YES	YES
30	a	186.27	0.48910	YES	YES
31	a	212.71	1.69699	YES	YES
32	a	228.65	20.80204	YES	YES
33	a	242.37	1.85495	YES	YES
34	a	264.03	44.08325	YES	YES
35	a	276.19	4.43790	YES	YES
36	a	292.65	0.96376	YES	YES
37	a	302.82	0.98313	YES	YES
38	a	309.65	17.65113	YES	YES
39	a	352.68	20.76833	YES	YES
40	a	393.71	27.46983	YES	YES
41	a	407.80	130.44939	YES	YES
42	a	449.41	7.56143	YES	YES
43	a	452.09	30.72487	YES	YES
44	a	468.51	1.99463	YES	YES
45	a	494.18	57.87941	YES	YES
46	a	520.99	26.96420	YES	YES
47	a	527.20	0.78251	YES	YES
48	a	532.76	11.91222	YES	YES
49	a	543.16	4.92279	YES	YES
50	a	545.27	46.24902	YES	YES
51	a	548.98	35.68813	YES	YES
52	a	560.25	2.88175	YES	YES
53	a	573.90	61.57546	YES	YES
54	a	692.45	0.13789	YES	YES
55	a	706.49	1.38729	YES	YES
56	a	715.19	68.35926	YES	YES
57	a	720.29	2.99388	YES	YES

58	a	731.89	15.74857	YES	YES
59	a	735.59	22.57817	YES	YES
60	a	784.65	58.35882	YES	YES
61	a	792.77	72.42346	YES	YES
62	a	805.69	21.34350	YES	YES
63	a	821.43	4.20034	YES	YES
64	a	845.06	301.77194	YES	YES
65	a	859.31	403.10969	YES	YES
66	a	877.33	382.96776	YES	YES
67	a	878.84	35.58620	YES	YES
68	a	883.79	14.33971	YES	YES
69	a	886.81	1.30294	YES	YES
70	a	913.28	2.32018	YES	YES
71	a	919.75	10.74257	YES	YES
72	a	968.38	11.01669	YES	YES
73	a	972.38	14.66007	YES	YES
74	a	981.55	13.62695	YES	YES
75	a	1007.06	97.59925	YES	YES
76	a	1034.54	2.87423	YES	YES
77	a	1046.30	4.91852	YES	YES
78	a	1096.16	209.92531	YES	YES
79	a	1099.51	170.09599	YES	YES
80	a	1113.06	18.15852	YES	YES
81	a	1136.73	4.42899	YES	YES
82	a	1156.83	2.12643	YES	YES
83	a	1157.19	0.58086	YES	YES
84	a	1172.14	188.08721	YES	YES
85	a	1181.28	7.07393	YES	YES
86	a	1201.57	4.22998	YES	YES
87	a	1204.93	244.79270	YES	YES
88	a	1233.26	4.34491	YES	YES
89	a	1241.51	10.73157	YES	YES
90	a	1245.67	13.15223	YES	YES
91	a	1252.46	13.59391	YES	YES
92	a	1257.83	14.34197	YES	YES
93	a	1327.84	247.80077	YES	YES
94	a	1351.27	456.30924	YES	YES
95	a	1391.71	0.06658	YES	YES
96	a	1394.66	0.00505	YES	YES
97	a	1413.14	47.39976	YES	YES
98	a	1421.18	44.17924	YES	YES
99	a	1430.03	39.07033	YES	YES
100	a	1435.01	63.72299	YES	YES
101	a	1486.63	16.37159	YES	YES
102	a	1488.63	62.81274	YES	YES
103	a	1491.66	18.72393	YES	YES
104	a	1502.60	8.60947	YES	YES
105	a	1504.76	6.16216	YES	YES
106	a	1515.62	14.48866	YES	YES
107	a	1519.96	46.31453	YES	YES
108	a	1520.56	17.16215	YES	YES
109	a	1524.62	0.94082	YES	YES
110	a	1529.34	0.36644	YES	YES
111	a	1760.45	672.26848	YES	YES
112	a	1804.04	686.94140	YES	YES
113	a	1848.35	603.26304	YES	YES
114	a	3042.82	17.05597	YES	YES
115	a	3060.49	22.65014	YES	YES
116	a	3069.76	29.35285	YES	YES
117	a	3072.55	38.81383	YES	YES
118	a	3077.38	15.67485	YES	YES
119	a	3095.31	20.45766	YES	YES
120	a	3097.10	23.13233	YES	YES

121	a	3106.48	18.45099	YES	YES
122	a	3129.70	12.49874	YES	YES
123	a	3132.37	7.15807	YES	YES
124	a	3137.09	9.34376	YES	YES
125	a	3147.17	14.36352	YES	YES
126	a	3151.67	7.53880	YES	YES
127	a	3163.67	12.67315	YES	YES
128	a	3170.60	2.92850	YES	YES
129	a	3170.73	0.95282	YES	YES

$end

Total COSMO energy + OC corr. = -2016.0716218853 H

(EC)(EMC)$_2$Li[PF$_6$]

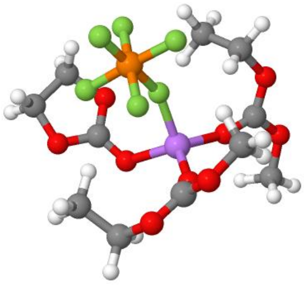

Method: (RI-)B3LYP(D3BJ)/def2-TZVPP
Symmetry: c1

Cartesian coordinates in Ångström:

F	-1.6103511	0.1720279	0.5297129
F	-0.0965981	-0.6717199	-1.0050404
P	-1.6476298	-0.2330947	-1.0093811
F	-2.0906214	-1.7467322	-0.6520881
F	-1.2386856	1.2658055	-1.4634486
Li	-2.4354319	-1.7313799	-3.9923623
H	0.4105321	-0.9628272	-3.7124736
H	-1.4108508	-3.6160334	-7.2666951
F	-3.2232609	0.1831553	-1.1245059
O	-1.2156956	-1.7240645	-5.4268750
H	1.9619395	-0.2520298	-4.1908270
C	0.9016304	-0.3050962	-4.4223558
C	-0.2182202	-1.5577885	-6.1042542
F	-1.7043275	-0.6513404	-2.6348911
O	0.8783513	-0.9288554	-5.7458664
C	-1.2100390	-2.7342103	-7.8719366
O	-0.0838437	-2.0106716	-7.3455064
H	-0.9206193	-3.0188920	-8.8780923
H	-2.0931376	-2.0986777	-7.8917576
C	0.2616142	1.0625937	-4.4534356
H	0.3263086	1.5042688	-3.4597718
H	-0.7946532	0.9967481	-4.7081524
H	0.7683549	1.7115487	-5.1680675
O	-5.3829004	0.6366881	-3.0656985
H	-6.0686020	2.5624285	-2.8306740
H	-5.2006460	1.8683343	-1.4310943
C	-5.2208385	1.9599424	-2.5128428
O	-4.1367673	-0.7755723	-4.2832986
C	-4.3166048	0.3143586	-3.7970154
C	-3.8764429	2.4334497	-3.0902665
O	-3.4782896	1.3401841	-3.9431501

```
H    -3.9587450    3.3256639   -3.7058425
H    -3.1092874    2.5550711   -2.3318945
H    -4.6457848   -1.8419534   -1.4818730
H    -5.6259235   -2.8135150   -0.3796450
C    -5.4553547   -2.5654185   -1.4268958
H    -0.3171665   -3.2336017   -1.6843535
H    -6.3553289   -2.0978975   -1.8285207
C    -0.4693258   -4.2855569   -1.9126625
H     0.1837234   -4.9041139   -1.3061361
C    -2.7858498   -4.1114635   -2.2221352
O    -1.8060314   -4.6738360   -1.5399707
C    -5.1364249   -3.8080705   -2.2273877
H    -0.3016196   -4.4737384   -2.9712296
O    -3.9458295   -4.4564802   -1.6992247
H    -4.9764997   -3.5817342   -3.2787359
O    -2.6328275   -3.4133228   -3.2098679
H    -5.9117982   -4.5650504   -2.1330426

SCF energy GEOOPT =  -2056.566698022 H
ZPE =   914.9 kJ/mol
FREEH energy =  1003.15 kJ/mol
FREEH entropy =   0.93117 kJ/mol/K

$vibrational spectrum
#  mode     symmetry     wave number   IR intensity    selection rules
#                         cm**(-1)        km/mol          IR     RAMAN
     1                      -0.00         0.00000         -        -
     2                      -0.00         0.00000         -        -
     3                       0.00         0.00000         -        -
     4                       0.00         0.00000         -        -
     5                       0.00         0.00000         -        -
     6                       0.00         0.00000         -        -
     7         a            16.23         0.11635        YES      YES
     8         a            17.60         0.43285        YES      YES
     9         a            24.56         0.35040        YES      YES
    10         a            30.04         0.43631        YES      YES
    11         a            33.42         0.44452        YES      YES
    12         a            44.11         0.45443        YES      YES
    13         a            45.99         2.46152        YES      YES
    14         a            52.97         0.45579        YES      YES
    15         a            56.74         0.26378        YES      YES
    16         a            63.89         0.80597        YES      YES
    17         a            71.07         0.34261        YES      YES
    18         a            72.08         1.92143        YES      YES
    19         a            79.88         4.28448        YES      YES
    20         a            83.51         0.88965        YES      YES
    21         a            84.96         0.27401        YES      YES
    22         a            96.67         1.52071        YES      YES
    23         a            99.25         5.05184        YES      YES
    24         a           102.37         1.37004        YES      YES
    25         a           104.27         7.13957        YES      YES
    26         a           113.45         6.60214        YES      YES
    27         a           122.52         0.65641        YES      YES
    28         a           125.08         2.06637        YES      YES
    29         a           139.55         1.68936        YES      YES
    30         a           146.73         1.62516        YES      YES
    31         a           161.85         4.08220        YES      YES
    32         a           177.14         1.62319        YES      YES
    33         a           189.94         3.86639        YES      YES
    34         a           207.56         2.50706        YES      YES
    35         a           217.43         1.62105        YES      YES
    36         a           239.26         6.31364        YES      YES
    37         a           273.33         1.81314        YES      YES
```

38	a	282.50	1.22476	YES	YES
39	a	298.76	0.22389	YES	YES
40	a	303.58	0.37194	YES	YES
41	a	304.44	4.33050	YES	YES
42	a	349.76	11.96254	YES	YES
43	a	365.52	49.59399	YES	YES
44	a	366.65	32.90136	YES	YES
45	a	401.12	8.56277	YES	YES
46	a	406.10	4.60596	YES	YES
47	a	449.92	96.71746	YES	YES
48	a	453.58	16.83696	YES	YES
49	a	461.48	40.64584	YES	YES
50	a	465.62	0.60397	YES	YES
51	a	479.82	110.01164	YES	YES
52	a	525.98	25.62577	YES	YES
53	a	536.10	2.85988	YES	YES
54	a	538.40	18.85628	YES	YES
55	a	544.01	24.68117	YES	YES
56	a	545.48	34.04412	YES	YES
57	a	548.66	74.74017	YES	YES
58	a	562.16	9.12917	YES	YES
59	a	566.54	3.24449	YES	YES
60	a	690.17	0.03261	YES	YES
61	a	691.79	1.13167	YES	YES
62	a	721.68	39.84224	YES	YES
63	a	736.64	20.06558	YES	YES
64	a	756.53	2.52266	YES	YES
65	a	787.42	82.54436	YES	YES
66	a	803.55	61.37218	YES	YES
67	a	805.59	29.31351	YES	YES
68	a	816.94	10.47337	YES	YES
69	a	820.87	16.21222	YES	YES
70	a	849.08	283.88212	YES	YES
71	a	856.94	336.04515	YES	YES
72	a	859.55	305.08028	YES	YES
73	a	861.80	54.95417	YES	YES
74	a	881.37	42.53303	YES	YES
75	a	887.08	39.41460	YES	YES
76	a	915.18	11.53499	YES	YES
77	a	962.42	8.96288	YES	YES
78	a	964.24	21.04050	YES	YES
79	a	986.34	12.01965	YES	YES
80	a	1007.38	86.43618	YES	YES
81	a	1009.88	95.79601	YES	YES
82	a	1042.75	3.45672	YES	YES
83	a	1107.54	186.49604	YES	YES
84	a	1114.80	5.54880	YES	YES
85	a	1115.47	30.14457	YES	YES
86	a	1138.46	3.52504	YES	YES
87	a	1142.28	9.96026	YES	YES
88	a	1158.90	0.05179	YES	YES
89	a	1179.87	1.06771	YES	YES
90	a	1183.50	1.02397	YES	YES
91	a	1199.70	4.50665	YES	YES
92	a	1201.29	5.51762	YES	YES
93	a	1207.23	230.03083	YES	YES
94	a	1230.56	5.14359	YES	YES
95	a	1233.98	1.55785	YES	YES
96	a	1248.15	2.16224	YES	YES
97	a	1257.17	6.90352	YES	YES
98	a	1324.19	130.35163	YES	YES
99	a	1324.36	100.63517	YES	YES
100	a	1342.25	440.46159	YES	YES

101	a	1347.39	636.69610	YES	YES
102	a	1395.29	1.53680	YES	YES
103	a	1413.93	96.98324	YES	YES
104	a	1414.63	95.03429	YES	YES
105	a	1426.73	15.36582	YES	YES
106	a	1428.51	11.35404	YES	YES
107	a	1439.97	74.12041	YES	YES
108	a	1484.18	76.34189	YES	YES
109	a	1485.65	78.45321	YES	YES
110	a	1485.81	9.22676	YES	YES
111	a	1489.23	11.35014	YES	YES
112	a	1489.36	10.22496	YES	YES
113	a	1492.14	12.46945	YES	YES
114	a	1499.13	3.83394	YES	YES
115	a	1500.45	12.75905	YES	YES
116	a	1501.35	13.23356	YES	YES
117	a	1508.26	5.85847	YES	YES
118	a	1514.27	25.15124	YES	YES
119	a	1515.13	99.16243	YES	YES
120	a	1522.22	17.37039	YES	YES
121	a	1533.83	0.39607	YES	YES
122	a	1736.87	746.42785	YES	YES
123	a	1758.82	327.76922	YES	YES
124	a	1820.14	559.67575	YES	YES
125	a	3046.90	15.62017	YES	YES
126	a	3048.00	13.26977	YES	YES
127	a	3057.26	23.34753	YES	YES
128	a	3061.69	30.10321	YES	YES
129	a	3080.78	30.06770	YES	YES
130	a	3085.04	18.08188	YES	YES
131	a	3091.99	43.04150	YES	YES
132	a	3099.88	15.15325	YES	YES
133	a	3107.72	15.47184	YES	YES
134	a	3115.77	9.55495	YES	YES
135	a	3129.72	8.95324	YES	YES
136	a	3130.21	7.97916	YES	YES
137	a	3130.65	15.87277	YES	YES
138	a	3140.08	3.05994	YES	YES
139	a	3143.77	7.13977	YES	YES
140	a	3151.75	9.41966	YES	YES
141	a	3159.42	4.00583	YES	YES
142	a	3163.35	6.87019	YES	YES
143	a	3164.59	13.51555	YES	YES
144	a	3167.71	8.83945	YES	YES

$end

Total COSMO energy + OC corr. = -2056.5916268277 H

[*pf*]$^{\cdot 2-}$

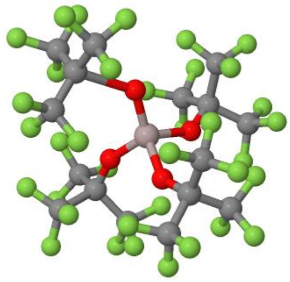

Method: (RI-)B3LYP(D3BJ)/def2-TZVPP
Symmetry: c1

Cartesian coordinates in Ångström:

Al	-0.1709038	0.1157408	0.0294015
O	-0.0890145	-1.5487940	-0.6202382
O	1.5266597	0.3684807	0.5050100
O	-0.9178723	1.3476360	-0.8502216
O	-1.0752598	-0.1777504	1.5606190
C	2.6980466	-0.0891723	0.9313312
F	2.4697622	-2.4918948	0.8357515
C	2.5780995	-1.4677261	1.6930896
C	-0.0813961	2.4579640	-2.5587306
F	-1.6196229	1.3329810	-3.9374947
C	-1.7983058	0.4511158	2.4857537
F	-2.8181297	-0.1352040	4.6408680
C	-0.7778126	-2.5897552	-1.0793572
F	2.1940573	1.8611437	-2.5795560
C	1.0016933	1.6646969	-3.1813633
F	1.2231139	1.9749523	-4.5195009
F	0.7862749	0.3309460	-3.1913184
C	-1.3568898	2.5027949	-3.3144263
F	-1.3389230	3.4477399	-4.3474032
F	-2.4445555	2.8297419	-2.6033853
C	0.3230289	3.6850778	-1.8345220
F	1.0432883	4.5777248	-2.6346538
F	-0.7312456	4.4041113	-1.3981014
F	1.1276094	3.4782751	-0.7771789
F	4.7744565	-1.0311305	0.0411892
F	1.5062840	-1.4792733	2.4821676
F	3.6563273	-1.7276959	2.4776811
C	3.6888797	-0.2799400	-0.2817355
F	4.1552284	0.9014058	-0.7147644
F	3.0799784	-0.8791508	-1.2995937
F	3.1571135	2.2092134	1.4572684
C	3.3171582	0.9721061	1.9211511
F	2.7178307	0.9108987	3.1239011
F	4.6447095	0.7927197	2.1354152
F	-3.5731957	0.5697585	0.8764480
C	-3.0118424	1.2563963	1.8679113
F	-3.9884406	1.4888263	2.7929021
F	-2.6342965	2.4463256	1.4040203
F	-2.8579956	-3.1853141	-2.2108126
C	-2.0007474	-2.1551463	-1.9740098
F	-2.6982525	-1.1898582	-1.3872772
F	-1.5874179	-1.7106235	-3.1663373
F	-0.4501912	-4.3859043	-2.7177737
C	0.1951817	-3.4727335	-1.9516563
F	1.0495115	-4.1607393	-1.1696033
F	0.9237191	-2.7162759	-2.7682406

```
F    -0.4033129   -3.5754118    1.0795517
C    -1.3184319   -3.4719022    0.1148047
F    -2.4212216   -2.9376959    0.6567701
F    -1.6401112   -4.7359112   -0.2645476
F    -1.6396352    2.2991488    4.0850229
C    -2.3761303   -0.6428371    3.4642437
F    -1.4501634   -1.5584064    3.7603967
F    -3.4133311   -1.2916252    2.9064420
C    -0.9105539    1.4486727    3.3215085
F    -0.1442423    2.1842587    2.5225094
F    -0.0985674    0.7770187    4.1606741

SCF energy GEOOPT =  -4748.000709881 H
ZPE =   565.9 kJ/mol
FREEH energy =   713.49 kJ/mol
FREEH entropy =   1.37889 kJ/mol/K

$vibrational spectrum
#  mode     symmetry     wave number   IR intensity    selection rules
#                         cm**(-1)        km/mol          IR     RAMAN
     1                      -0.00          0.00000         -       -
     2                      -0.00          0.00000         -       -
     3                       0.00          0.00000         -       -
     4                       0.00          0.00000         -       -
     5                       0.00          0.00000         -       -
     6                       0.00          0.00000         -       -
     7         a            10.26          0.03279        YES     YES
     8         a            11.87          0.00195        YES     YES
     9         a            14.13          0.04916        YES     YES
    10         a            16.19          0.00854        YES     YES
    11         a            19.06          0.10469        YES     YES
    12         a            24.99          0.28864        YES     YES
    13         a            29.84          0.33548        YES     YES
    14         a            33.90          0.22915        YES     YES
    15         a            36.43          0.19887        YES     YES
    16         a            40.12          0.23845        YES     YES
    17         a            41.80          0.01209        YES     YES
    18         a            46.33          0.29204        YES     YES
    19         a            53.91          0.10188        YES     YES
    20         a            56.28          0.13833        YES     YES
    21         a            57.82          0.10796        YES     YES
    22         a            65.95          0.10749        YES     YES
    23         a            69.51          0.04391        YES     YES
    24         a            70.27          0.03927        YES     YES
    25         a            71.14          0.07677        YES     YES
    26         a            71.82          0.15682        YES     YES
    27         a            75.42          0.14429        YES     YES
    28         a            80.34          0.11249        YES     YES
    29         a            82.02          0.03524        YES     YES
    30         a            83.88          0.07971        YES     YES
    31         a            87.51          0.12815        YES     YES
    32         a            90.53          0.00332        YES     YES
    33         a            92.21          0.02798        YES     YES
    34         a            94.10          0.09911        YES     YES
    35         a            96.49          0.10192        YES     YES
    36         a           103.67          0.29680        YES     YES
    37         a           147.23          1.30240        YES     YES
    38         a           148.74          1.72218        YES     YES
    39         a           160.19          0.04588        YES     YES
    40         a           163.71          0.06502        YES     YES
    41         a           166.03          0.45425        YES     YES
    42         a           167.35          0.41631        YES     YES
    43         a           167.69          0.14436        YES     YES
```

44	a	170.82	0.57494	YES	YES
45	a	177.04	1.41733	YES	YES
46	a	180.45	0.25445	YES	YES
47	a	194.85	1.10093	YES	YES
48	a	203.82	4.88008	YES	YES
49	a	207.87	2.25255	YES	YES
50	a	235.09	4.66590	YES	YES
51	a	257.99	0.30804	YES	YES
52	a	265.62	1.21084	YES	YES
53	a	275.08	1.84812	YES	YES
54	a	277.91	2.07914	YES	YES
55	a	280.26	2.18479	YES	YES
56	a	283.65	1.00320	YES	YES
57	a	288.96	0.11116	YES	YES
58	a	289.26	0.08944	YES	YES
59	a	289.64	0.16760	YES	YES
60	a	294.79	17.36681	YES	YES
61	a	304.19	13.20929	YES	YES
62	a	305.10	10.98629	YES	YES
63	a	308.43	1.76072	YES	YES
64	a	311.05	1.30871	YES	YES
65	a	312.37	2.02900	YES	YES
66	a	314.61	0.11679	YES	YES
67	a	316.06	1.88000	YES	YES
68	a	321.21	0.31677	YES	YES
69	a	322.02	1.99619	YES	YES
70	a	325.42	3.29747	YES	YES
71	a	327.36	3.07324	YES	YES
72	a	334.47	4.16709	YES	YES
73	a	336.78	0.40049	YES	YES
74	a	344.51	0.26940	YES	YES
75	a	346.97	1.70541	YES	YES
76	a	348.36	1.34240	YES	YES
77	a	354.44	11.61700	YES	YES
78	a	360.78	11.33780	YES	YES
79	a	365.94	7.44963	YES	YES
80	a	376.47	33.42331	YES	YES
81	a	412.30	56.25820	YES	YES
82	a	434.33	64.41413	YES	YES
83	a	446.52	73.89246	YES	YES
84	a	493.45	0.03295	YES	YES
85	a	522.20	0.23812	YES	YES
86	a	526.54	2.09170	YES	YES
87	a	527.32	2.55808	YES	YES
88	a	527.79	7.22473	YES	YES
89	a	528.68	3.71936	YES	YES
90	a	528.84	5.38861	YES	YES
91	a	529.40	7.17721	YES	YES
92	a	530.50	3.70409	YES	YES
93	a	531.55	10.88518	YES	YES
94	a	532.79	7.36437	YES	YES
95	a	533.61	9.54394	YES	YES
96	a	537.35	0.06674	YES	YES
97	a	537.89	1.04249	YES	YES
98	a	541.65	16.50226	YES	YES
99	a	543.65	22.60423	YES	YES
100	a	545.31	0.39695	YES	YES
101	a	547.01	0.28045	YES	YES
102	a	562.17	0.34432	YES	YES
103	a	563.14	0.60390	YES	YES
104	a	563.55	0.58678	YES	YES
105	a	564.63	0.95482	YES	YES
106	a	565.04	1.26198	YES	YES

107	a	566.10	4.54127	YES	YES
108	a	695.58	18.50649	YES	YES
109	a	697.09	16.58641	YES	YES
110	a	714.02	30.50523	YES	YES
111	a	719.20	11.04277	YES	YES
112	a	719.93	3.40589	YES	YES
113	a	720.19	10.63155	YES	YES
114	a	721.37	35.95272	YES	YES
115	a	721.40	46.52968	YES	YES
116	a	722.14	68.22679	YES	YES
117	a	728.47	2.95839	YES	YES
118	a	736.74	22.50332	YES	YES
119	a	740.31	15.32188	YES	YES
120	a	747.25	24.01334	YES	YES
121	a	767.88	10.38361	YES	YES
122	a	774.99	14.84497	YES	YES
123	a	782.29	8.45323	YES	YES
124	a	876.08	471.04268	YES	YES
125	a	892.87	132.58134	YES	YES
126	a	906.66	145.70949	YES	YES
127	a	956.36	48.46005	YES	YES
128	a	958.78	21.98447	YES	YES
129	a	961.59	60.27421	YES	YES
130	a	962.81	231.18016	YES	YES
131	a	967.38	168.70629	YES	YES
132	a	968.37	364.80357	YES	YES
133	a	1007.34	197.62150	YES	YES
134	a	1010.45	192.32763	YES	YES
135	a	1018.87	614.93663	YES	YES
136	a	1086.86	162.36966	YES	YES
137	a	1087.48	95.80690	YES	YES
138	a	1096.96	12.69967	YES	YES
139	a	1102.29	38.39454	YES	YES
140	a	1103.28	16.83769	YES	YES
141	a	1105.78	87.42460	YES	YES
142	a	1106.18	64.21893	YES	YES
143	a	1109.29	16.63704	YES	YES
144	a	1113.31	4.22465	YES	YES
145	a	1113.62	5.36458	YES	YES
146	a	1162.15	59.66181	YES	YES
147	a	1178.17	24.09808	YES	YES
148	a	1194.09	1122.06342	YES	YES
149	a	1196.94	604.17142	YES	YES
150	a	1199.25	557.97240	YES	YES
151	a	1207.94	36.37860	YES	YES
152	a	1210.56	221.58430	YES	YES
153	a	1212.02	227.91747	YES	YES
154	a	1213.19	225.75520	YES	YES
155	a	1215.46	144.30383	YES	YES
156	a	1222.93	29.12271	YES	YES
157	a	1240.18	63.24797	YES	YES
158	a	1243.23	87.01157	YES	YES
159	a	1245.91	76.08722	YES	YES
160	a	1247.82	58.13139	YES	YES
161	a	1253.43	54.25870	YES	YES
162	a	1257.45	105.33590	YES	YES
163	a	1263.34	621.73148	YES	YES
164	a	1267.53	561.20711	YES	YES
165	a	1269.44	136.67047	YES	YES
166	a	1275.22	475.73067	YES	YES
167	a	1330.70	77.09507	YES	YES
168	a	1335.83	569.41646	YES	YES
169	a	1338.56	637.11717	YES	YES

```
   170         a            1341.48       451.11768       YES      YES
   171         a            1360.70       145.24092       YES      YES
$end

Total COSMO energy + OC corr. = -4748.1720327762 H
```

$[Ga(C_2F_5)_4]^{\cdot 2-}$

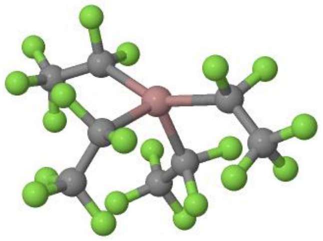

```
Method: (RI-)B3LYP(D3BJ)/def2-TZVPP
Symmetry: c1

Cartesian coordinates in Ångström:
F     1.6315734    3.2822172   -2.9528415
F     3.7108778    3.6413220   -2.4540068
F    -2.4783499    3.6311040   -0.3924601
C     2.4457739    3.5230280   -1.9050118
F     2.8603896    1.3092930   -1.4252364
F     2.1504081    4.7491329   -1.4333466
C     2.3146852    2.4527712   -0.8243408
C    -1.3540573    4.3333659   -0.1671513
F    -1.7639909    5.5711869    0.2566045
F    -1.1909086    3.6062611    2.0056968
F     0.5106278    0.5564612   -2.9726717
C    -0.4366238    3.6246338    0.8365341
Ga    0.5049040    1.7052134    0.4626637
F    -0.7477833    4.5304848   -1.3495271
F     3.2749336    2.8442469    0.1209220
F    -0.4902885   -0.7323436   -0.7861091
C    -0.7037364    0.8223443   -2.4672968
C    -0.7460061    0.6323455   -0.9472980
F     0.5703456    4.5610836    1.0840700
F    -1.5760078   -0.0004126   -3.1317483
F    -1.0388994    2.0750727   -2.8215955
F    -2.0984465    0.7763571   -0.6384096
F    -2.6550882    1.1649384    2.6014535
F     0.0168348    1.3292850    3.4352556
C    -1.7876548    0.1356665    2.6388270
F    -1.9480187   -0.4254464    3.8954909
C    -0.3612387    0.5756303    2.3178300
F    -2.2275470   -0.8015144    1.7777677
F     0.3745320   -0.6136581    2.4336058

SCF energy GEOOPT =  -4226.664170573 H
ZPE =   254.6 kJ/mol
FREEH energy =   336.20 kJ/mol
FREEH entropy =   0.86334 kJ/mol/K

$vibrational spectrum
#  mode     symmetry     wave number   IR intensity    selection rules
#                         cm**(-1)        km/mol         IR      RAMAN
     1                       -0.00         0.00000        -        -
     2                       -0.00         0.00000        -        -
     3                       -0.00         0.00000        -        -
```

4		-0.00	0.00000	-	-
5		-0.00	0.00000	-	-
6		0.00	0.00000	-	-
7	a	19.70	0.10782	YES	YES
8	a	32.07	0.02056	YES	YES
9	a	35.71	0.24127	YES	YES
10	a	42.44	0.08838	YES	YES
11	a	48.53	0.84642	YES	YES
12	a	52.09	0.03347	YES	YES
13	a	56.57	0.09985	YES	YES
14	a	62.44	0.06265	YES	YES
15	a	69.67	0.33900	YES	YES
16	a	73.91	0.03109	YES	YES
17	a	77.02	3.24193	YES	YES
18	a	78.79	1.64629	YES	YES
19	a	82.85	0.03339	YES	YES
20	a	86.52	0.03465	YES	YES
21	a	92.95	3.35008	YES	YES
22	a	97.59	0.51809	YES	YES
23	a	115.03	1.52437	YES	YES
24	a	129.74	10.11481	YES	YES
25	a	151.55	14.20394	YES	YES
26	a	167.65	0.36433	YES	YES
27	a	178.39	8.56665	YES	YES
28	a	182.01	3.59015	YES	YES
29	a	203.81	8.87867	YES	YES
30	a	219.64	2.39005	YES	YES
31	a	224.23	1.29665	YES	YES
32	a	228.72	0.18418	YES	YES
33	a	229.12	1.91744	YES	YES
34	a	239.09	14.26816	YES	YES
35	a	244.45	22.86930	YES	YES
36	a	262.30	8.01396	YES	YES
37	a	273.25	4.80666	YES	YES
38	a	282.33	9.15987	YES	YES
39	a	283.14	9.95516	YES	YES
40	a	356.92	6.26951	YES	YES
41	a	359.10	0.24011	YES	YES
42	a	361.10	1.94536	YES	YES
43	a	362.04	1.27505	YES	YES
44	a	421.36	0.17654	YES	YES
45	a	422.04	0.04149	YES	YES
46	a	426.32	0.15707	YES	YES
47	a	428.36	0.31280	YES	YES
48	a	507.35	4.51127	YES	YES
49	a	509.13	0.91663	YES	YES
50	a	517.10	0.79847	YES	YES
51	a	519.98	0.55859	YES	YES
52	a	570.23	1.46888	YES	YES
53	a	570.91	0.78487	YES	YES
54	a	574.27	1.23518	YES	YES
55	a	578.05	3.74677	YES	YES
56	a	578.81	1.70230	YES	YES
57	a	579.34	0.90948	YES	YES
58	a	585.80	0.06631	YES	YES
59	a	588.46	0.70557	YES	YES
60	a	701.81	21.60329	YES	YES
61	a	704.95	1.84295	YES	YES
62	a	718.89	17.19276	YES	YES
63	a	719.86	5.61788	YES	YES
64	a	820.44	154.71588	YES	YES
65	a	828.31	30.12782	YES	YES
66	a	874.83	67.93679	YES	YES

67	a	881.83	87.79721	YES	YES
68	a	937.62	53.02644	YES	YES
69	a	942.96	61.01086	YES	YES
70	a	963.33	128.05191	YES	YES
71	a	970.73	194.32357	YES	YES
72	a	980.52	48.05140	YES	YES
73	a	983.87	15.77605	YES	YES
74	a	1041.51	243.60038	YES	YES
75	a	1049.00	270.36234	YES	YES
76	a	1050.31	97.96523	YES	YES
77	a	1058.47	420.94084	YES	YES
78	a	1092.27	529.62872	YES	YES
79	a	1100.71	53.60723	YES	YES
80	a	1158.57	96.08587	YES	YES
81	a	1161.58	346.40876	YES	YES
82	a	1174.00	169.93013	YES	YES
83	a	1179.20	158.92739	YES	YES
84	a	1231.30	746.97230	YES	YES
85	a	1249.94	55.29741	YES	YES
86	a	1266.69	181.76576	YES	YES
87	a	1283.82	341.32375	YES	YES

$end

Total COSMO energy + OC corr. = -4226.8716950995 H

[(DEC)Li]$^+$

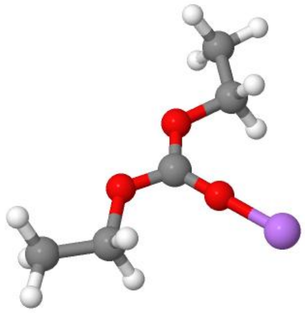

Method: (RI-)B3LYP(D3BJ)/def2-TZVPP
Symmetry: c2v

Cartesian coordinates in Ångström:

Li	0.0000000	0.0000000	-3.2829870
H	-0.8896511	-2.4453334	-0.8727596
H	-0.8858187	-3.3336895	1.4704497
H	-0.8896511	2.4453334	-0.8727596
C	0.0000000	-2.3850360	-0.2463302
O	0.0000000	0.0000000	-1.5720559
H	0.0000000	-4.4166997	0.3909872
C	0.0000000	-3.4252225	0.8440937
C	0.0000000	0.0000000	-0.3285721
O	0.0000000	-1.0741444	0.4046412
C	0.0000000	2.3850360	-0.2463302
H	-0.8858187	3.3336895	1.4704497
O	0.0000000	1.0741444	0.4046412
H	0.8896511	-2.4453334	-0.8727596
C	0.0000000	3.4252225	0.8440937
H	0.0000000	4.4166997	0.3909872
H	0.8858187	-3.3336895	1.4704497
H	0.8896511	2.4453334	-0.8727596

```
H     0.8858187    3.3336895    1.4704497

SCF energy GEOOPT =  -429.5719153762 H
ZPE =   403.5 kJ/mol
FREEH energy =   432.76 kJ/mol
FREEH entropy =   0.43294 kJ/mol/K

$vibrational spectrum
#  mode     symmetry     wave number   IR intensity    selection rules
#                         cm**(-1)        km/mol          IR     RAMAN
     1                      -0.00          0.00000         -       -
     2                      -0.00          0.00000         -       -
     3                      -0.00          0.00000         -       -
     4                      -0.00          0.00000         -       -
     5                       0.00          0.00000         -       -
     6                       0.00          0.00000         -       -
     7         b1           56.62         10.45419        YES     YES
     8         a2           64.24          0.00000        NO      YES
     9         b2           75.64         67.38303        YES     YES
    10         b1           98.58          5.91324        YES     YES
    11         a1          121.29          0.11183        YES     YES
    12         b1          139.27         45.30703        YES     YES
    13         a2          158.92          0.00000        NO      YES
    14         b2          252.69         10.05404        YES     YES
    15         b1          257.17          0.95242        YES     YES
    16         a2          263.97          0.00000        NO      YES
    17         a1          343.01          0.35939        YES     YES
    18         b2          383.46         30.07576        YES     YES
    19         a1          476.01         36.13285        YES     YES
    20         a1          635.69        161.79601        YES     YES
    21         b2          733.71          1.13773        YES     YES
    22         b1          806.77         30.75811        YES     YES
    23         a2          826.05          0.00000        NO      YES
    24         b1          831.09          0.40527        YES     YES
    25         b2          845.68         90.23411        YES     YES
    26         a1          932.04          8.20532        YES     YES
    27         b2         1012.78        153.66116        YES     YES
    28         a1         1020.46          0.30680        YES     YES
    29         b2         1134.47          0.35167        YES     YES
    30         a1         1136.90          1.88052        YES     YES
    31         a2         1177.07          0.00000        NO      YES
    32         b1         1180.65          9.65547        YES     YES
    33         a1         1191.58          3.18205        YES     YES
    34         a2         1301.71          0.00000        NO      YES
    35         b1         1303.41          3.82471        YES     YES
    36         b2         1354.84        588.68623        YES     YES
    37         a1         1394.20         26.34633        YES     YES
    38         b2         1427.70         65.45995        YES     YES
    39         a1         1430.70         14.90113        YES     YES
    40         b2         1485.42        375.47722        YES     YES
    41         a2         1485.48          0.00000        NO      YES
    42         b1         1485.63         18.96763        YES     YES
    43         b2         1498.32          0.43393        YES     YES
    44         a1         1498.47          2.70425        YES     YES
    45         a1         1514.14         29.82494        YES     YES
    46         b2         1528.71        175.31465        YES     YES
    47         a1         1663.66        499.03786        YES     YES
    48         b2         3052.72          5.03410        YES     YES
    49         a1         3052.86          0.24713        YES     YES
    50         b2         3054.22          0.11505        YES     YES
    51         a1         3054.83         39.52127        YES     YES
    52         a2         3099.69          0.00000        NO      YES
    53         b1         3100.26         16.02305        YES     YES
```

54	b2	3122.10	3.83976	YES	YES
55	a1	3122.15	10.69094	YES	YES
56	a2	3135.03	0.00000	NO	YES
57	b1	3135.21	28.98379	YES	YES

$end

Total COSMO energy + OC corr. = -429.6702876222 H

[(FEC)Li]$^+$

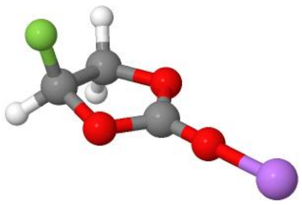

Method: (RI-)B3LYP(D3BJ)/def2-TZVPP
Symmetry: c1

Cartesian coordinates in Ångström:

Li	-2.4721314	-3.1602655	0.3491074
F	0.4401966	1.5198840	1.5236720
H	2.1115338	0.4170743	0.0812463
O	0.6102277	-0.9410682	-0.3669278
C	-0.6451361	-0.8633825	0.0089754
O	-1.3842154	-1.8257993	0.1069463
C	1.1649096	0.4003539	-0.4495372
C	0.1006442	1.2516545	0.2404311
O	-1.0400134	0.3795295	0.2625067
H	-0.1833984	2.1742072	-0.2567298
H	1.2973828	0.6478121	-1.4996906

SCF energy GEOOPT = -449.0006699186 H
ZPE = 181.8 kJ/mol
FREEH energy = 200.46 kJ/mol
FREEH entropy = 0.35630 kJ/mol/K

$vibrational spectrum

# mode	symmetry	wave number	IR intensity	selection rules	
#		cm**(-1)	km/mol	IR	RAMAN
1		-0.00	0.00000	-	-
2		0.00	0.00000	-	-
3		0.00	0.00000	-	-
4		0.00	0.00000	-	-
5		0.00	0.00000	-	-
6		0.00	0.00000	-	-
7	a	60.58	86.34383	YES	YES
8	a	87.81	58.46611	YES	YES
9	a	127.15	35.19032	YES	YES
10	a	212.32	4.11932	YES	YES
11	a	395.34	9.52953	YES	YES
12	a	468.16	37.57292	YES	YES
13	a	521.25	82.79127	YES	YES
14	a	563.13	37.39685	YES	YES
15	a	781.52	49.39037	YES	YES
16	a	790.82	126.77098	YES	YES
17	a	831.06	33.40351	YES	YES
18	a	879.10	18.60098	YES	YES

19	a	945.25	107.21356	YES	YES
20	a	1006.08	48.26853	YES	YES
21	a	1036.40	31.22790	YES	YES
22	a	1111.39	116.71804	YES	YES
23	a	1156.29	57.89025	YES	YES
24	a	1226.37	117.33610	YES	YES
25	a	1254.77	81.86748	YES	YES
26	a	1365.26	18.07381	YES	YES
27	a	1397.52	56.05728	YES	YES
28	a	1448.12	77.53087	YES	YES
29	a	1503.57	30.40621	YES	YES
30	a	1802.80	978.65768	YES	YES
31	a	3105.30	1.84435	YES	YES
32	a	3151.22	2.76676	YES	YES
33	a	3174.52	0.80106	YES	YES

$end

Total COSMO energy + OC corr. = -449.1067988731 H

Li(*o*-DFB)$_2$]$^+$ (F$_{o\text{-DFB}}$-coordination)

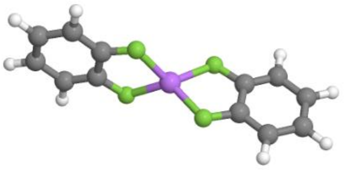

Method: (RI-)B3LYP(D3BJ)/def2-TZVPP
Symmetry: c2

Cartesian coordinates in Ångström:

C	-1.7875523	-2.0842768	-0.4885066
C	-0.9661946	-2.5864479	0.4990457
C	-1.1267364	-3.8561993	0.9979042
H	-0.4711033	-4.2281488	1.7718567
C	-2.1601297	-4.6253621	0.4635822
H	-2.3139915	-5.6284881	0.8334845
C	-2.9883593	-4.1189542	-0.5327389
H	-3.7833944	-4.7300280	-0.9342785
C	-2.8100640	-2.8270115	-1.0266734
H	-3.4406793	-2.4125094	-1.7997398
F	-1.5226762	-0.7938281	-0.9039793
F	0.0252498	-1.7400825	0.9562571
C	1.1267364	3.8561993	0.9979042
H	0.4711033	4.2281488	1.7718567
C	2.1601297	4.6253621	0.4635822
H	2.3139915	5.6284881	0.8334845
C	2.9883593	4.1189542	-0.5327389
H	3.7833944	4.7300280	-0.9342785
C	2.8100640	2.8270115	-1.0266734
H	3.4406793	2.4125094	-1.7997398
C	1.7875523	2.0842768	-0.4885066
C	0.9661946	2.5864479	0.4990457
F	1.5226762	0.7938281	-0.9039793
F	-0.0252498	1.7400825	0.9562571
Li	-0.0000000	0.0000000	0.0469228

SCF energy GEOOPT = -868.7869032400 H
ZPE = 449.8 kJ/mol
FREEH energy = 489.63 kJ/mol

FREEH entropy =   0.54743 kJ/mol/K

$vibrational spectrum

# mode	symmetry	wave number cm**(-1)	IR intensity km/mol	selection rules IR	RAMAN
1		-0.00	0.00000	-	-
2		-0.00	0.00000	-	-
3		-0.00	0.00000	-	-
4		0.00	0.00000	-	-
5		0.00	0.00000	-	-
6		0.00	0.00000	-	-
7	a	13.12	0.00228	YES	YES
8	b	14.15	0.00982	YES	YES
9	a	15.95	0.00073	YES	YES
10	a	70.14	3.47421	YES	YES
11	b	71.39	3.58377	YES	YES
12	a	90.00	0.00235	YES	YES
13	b	198.73	0.00013	YES	YES
14	a	199.43	0.00061	YES	YES
15	b	268.26	29.29639	YES	YES
16	a	269.68	28.46256	YES	YES
17	a	296.29	9.80657	YES	YES
18	b	296.48	9.68487	YES	YES
19	b	333.97	0.07907	YES	YES
20	a	344.20	0.00472	YES	YES
21	b	448.54	3.90548	YES	YES
22	a	448.76	4.45844	YES	YES
23	b	449.86	1.50176	YES	YES
24	a	449.96	0.83508	YES	YES
25	b	504.18	305.05387	YES	YES
26	a	553.65	0.00036	YES	YES
27	b	553.77	0.00064	YES	YES
28	b	557.86	7.20848	YES	YES
29	a	557.87	7.53972	YES	YES
30	a	574.09	0.01530	YES	YES
31	b	580.53	185.26183	YES	YES
32	b	708.59	0.00007	YES	YES
33	a	708.70	0.00001	YES	YES
34	b	763.80	159.16561	YES	YES
35	a	767.96	0.04013	YES	YES
36	a	775.82	76.85074	YES	YES
37	b	775.83	81.11445	YES	YES
38	b	836.13	23.26929	YES	YES
39	a	836.25	24.19781	YES	YES
40	b	876.72	0.00001	YES	YES
41	a	876.88	0.00004	YES	YES
42	a	974.38	2.82808	YES	YES
43	b	974.38	2.99414	YES	YES
44	b	1021.60	0.00001	YES	YES
45	a	1021.62	0.00002	YES	YES
46	b	1039.93	8.30827	YES	YES
47	a	1039.94	0.00222	YES	YES
48	b	1105.11	36.40632	YES	YES
49	a	1105.31	37.98161	YES	YES
50	b	1164.16	8.57291	YES	YES
51	a	1164.34	9.02827	YES	YES
52	b	1185.43	13.56608	YES	YES
53	a	1185.62	0.00199	YES	YES
54	b	1253.40	310.93272	YES	YES
55	a	1259.93	0.05548	YES	YES
56	b	1294.71	0.00529	YES	YES
57	a	1294.71	0.00559	YES	YES
58	b	1341.49	21.83807	YES	YES

59	a	1341.85	0.00443	YES	YES
60	b	1500.96	9.95332	YES	YES
61	a	1500.96	10.44598	YES	YES
62	b	1525.14	348.72356	YES	YES
63	a	1527.78	0.07804	YES	YES
64	b	1631.29	1.28777	YES	YES
65	a	1631.35	1.32379	YES	YES
66	b	1667.42	14.02956	YES	YES
67	a	1667.71	0.00243	YES	YES
68	b	3201.20	1.16505	YES	YES
69	a	3201.20	1.22632	YES	YES
70	b	3209.57	1.82062	YES	YES
71	a	3209.59	0.00007	YES	YES
72	a	3215.42	5.68385	YES	YES
73	b	3215.42	5.39775	YES	YES
74	b	3220.36	1.77092	YES	YES
75	a	3220.36	0.00499	YES	YES

$end

Total COSMO energy + OC corr. = -868.8495543297 H

[Li(DME)(*o*-DFB)]$^+$ (F$_{o\text{-DFB}}$-coordination)

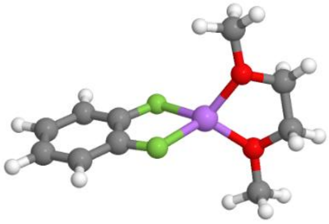

Method: (RI-)B3LYP(D3BJ)/def2-TZVPP
Symmetry: c1

Cartesian coordinates in Ångström:

C	-0.4281586	2.3322693	0.4328268
O	0.4871126	1.2800839	0.7674124
Li	-0.0675971	-0.2779610	-0.1506630
O	-1.1991634	0.8009219	-1.2146735
C	-0.8071848	2.1665102	-1.0195600
C	1.0865572	1.4300311	2.0601407
H	0.0467452	3.3044914	0.5827870
H	-1.3073637	2.2645110	1.0800905
C	-1.7762879	0.5476360	-2.5014655
H	-1.6368460	2.8337623	-1.2639009
H	0.0369235	2.3966499	-1.6761527
H	-2.0297204	-0.5083720	-2.5384243
H	-1.0641161	0.7850024	-3.2942514
H	-2.6811958	1.1429100	-2.6284766
H	1.7506707	0.5826474	2.2072947
H	0.3211406	1.4394799	2.8386429
H	1.6635241	2.3546628	2.0991657
H	2.4995839	-3.7873008	-1.7456127
F	1.1672716	-1.6117226	-1.0064936
C	1.7558800	-3.9418420	-0.9773020
C	1.0053062	-2.8862887	-0.5159393
H	2.0903608	-6.0439369	-0.7589612
C	1.5161234	-5.1954724	-0.4167109
C	0.0487798	-3.0525860	0.4650142
F	-0.6405308	-1.9259886	0.8484895
C	0.5518299	-5.3631166	0.5716517

```
C    -0.2003726   -4.2818766    1.0282048
H     0.3785403   -6.3415249    0.9953065
H    -0.9547824   -4.3876905    1.7943602

SCF energy GEOOPT =  -746.9498578748 H
ZPE =   604.0 kJ/mol
FREEH energy =   647.92 kJ/mol
FREEH entropy =   0.57641 kJ/mol/K

$vibrational spectrum
#  mode     symmetry     wave number   IR intensity    selection rules
#                         cm**(-1)        km/mol          IR     RAMAN
     1                      -0.00         0.00000         -        -
     2                      -0.00         0.00000         -        -
     3                       0.00         0.00000         -        -
     4                       0.00         0.00000         -        -
     5                       0.00         0.00000         -        -
     6                       0.00         0.00000         -        -
     7         a            15.20         0.01497        YES      YES
     8         a            16.07         0.01506        YES      YES
     9         a            27.80         0.18051        YES      YES
    10         a            58.26         2.76822        YES      YES
    11         a            68.47         2.35970        YES      YES
    12         a            92.67         2.88393        YES      YES
    13         a           119.15         2.25218        YES      YES
    14         a           128.61         0.13200        YES      YES
    15         a           161.98         0.06116        YES      YES
    16         a           198.32         0.00002        YES      YES
    17         a           206.48         0.06298        YES      YES
    18         a           210.28         0.12548        YES      YES
    19         a           266.79        41.08512        YES      YES
    20         a           280.58         0.87133        YES      YES
    21         a           288.62         0.57389        YES      YES
    22         a           332.04         0.57643        YES      YES
    23         a           332.75         7.52583        YES      YES
    24         a           356.00         9.26083        YES      YES
    25         a           446.38         2.04182        YES      YES
    26         a           451.23        12.48402        YES      YES
    27         a           461.12        35.44149        YES      YES
    28         a           555.93         0.00094        YES      YES
    29         a           557.79         6.40741        YES      YES
    30         a           560.57        91.66969        YES      YES
    31         a           575.52         5.65427        YES      YES
    32         a           584.84       177.83967        YES      YES
    33         a           712.31         0.00118        YES      YES
    34         a           769.29        74.45344        YES      YES
    35         a           775.97        78.12925        YES      YES
    36         a           839.56         4.84685        YES      YES
    37         a           841.58        21.71196        YES      YES
    38         a           877.01         0.40623        YES      YES
    39         a           877.98        61.21150        YES      YES
    40         a           973.27         2.71430        YES      YES
    41         a          1019.86         0.00095        YES      YES
    42         a          1024.63        16.78049        YES      YES
    43         a          1037.39         4.91733        YES      YES
    44         a          1041.50         4.08056        YES      YES
    45         a          1086.16       336.76490        YES      YES
    46         a          1111.93        31.80811        YES      YES
    47         a          1122.17        57.46579        YES      YES
    48         a          1128.12         1.96976        YES      YES
    49         a          1171.08        10.77526        YES      YES
    50         a          1180.42         4.36033        YES      YES
    51         a          1185.31         4.95766        YES      YES
```

52	a	1186.76	1.21068	YES	YES
53	a	1212.01	21.97179	YES	YES
54	a	1234.33	0.97130	YES	YES
55	a	1263.40	148.37400	YES	YES
56	a	1268.18	14.96205	YES	YES
57	a	1294.78	0.00180	YES	YES
58	a	1303.52	10.50430	YES	YES
59	a	1341.27	8.87324	YES	YES
60	a	1404.25	16.31783	YES	YES
61	a	1438.85	1.56423	YES	YES
62	a	1486.19	0.96244	YES	YES
63	a	1486.91	0.98373	YES	YES
64	a	1490.61	15.20138	YES	YES
65	a	1490.89	6.69078	YES	YES
66	a	1500.70	10.60307	YES	YES
67	a	1502.37	12.21409	YES	YES
68	a	1504.30	9.01943	YES	YES
69	a	1516.82	9.27565	YES	YES
70	a	1519.53	5.06401	YES	YES
71	a	1529.25	176.96981	YES	YES
72	a	1633.82	1.09570	YES	YES
73	a	1664.99	8.28008	YES	YES
74	a	3021.50	15.34524	YES	YES
75	a	3024.16	13.35200	YES	YES
76	a	3029.36	50.86887	YES	YES
77	a	3029.61	1.92561	YES	YES
78	a	3069.80	13.99270	YES	YES
79	a	3079.74	23.88321	YES	YES
80	a	3098.23	3.24410	YES	YES
81	a	3098.38	22.36302	YES	YES
82	a	3143.78	2.98790	YES	YES
83	a	3143.87	18.69938	YES	YES
84	a	3199.99	1.11728	YES	YES
85	a	3208.20	1.27299	YES	YES
86	a	3214.08	3.44770	YES	YES
87	a	3219.42	0.48733	YES	YES

$end

Total COSMO energy + OC corr. = -747.0083510871 H

[Li(*o*-DFB)(FEC)$_2$]$^+$ (F$_{o\text{-DFB}}$-coordination)

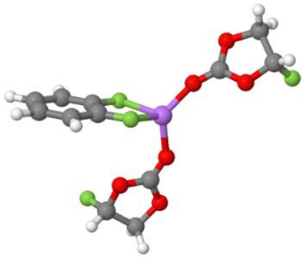

Method: (RI-)B3LYP(D3BJ)/def2-TZVPP
Symmetry: c1

Cartesian coordinates in Ångström:

O	1.1360716	1.0628116	-1.4008642
C	-0.1996839	0.9378328	-1.3153725
O	-0.7621656	0.4304963	-0.3797103
O	-0.8145380	1.4521288	-2.3688439
C	0.1632874	2.0481791	-3.2511440
C	1.4878150	1.5289690	-2.6975350

```
H     0.0725282    3.1301301   -3.1868982
H    -0.0193950    1.7013402   -4.2634877
H     2.2907132    2.2540677   -2.6030129
F     1.9395639    0.4687229   -3.4231146
F    -2.6655102   -4.6649295    3.9846304
H    -0.9575858   -6.1612638    3.0235694
O    -0.2027889   -4.4745461    2.0922616
C    -0.6041596   -3.2270122    2.2689926
O    -0.4747121   -2.3423938    1.4637605
C    -0.4548138   -5.2376836    3.2934616
C    -1.3541411   -4.3116770    4.1085280
O    -1.1746603   -3.0505840    3.4805093
H    -1.1192084   -4.2163857    5.1644988
H     0.4963222   -5.4396104    3.7808603
Li   -1.0839943   -0.6151176    1.0905961
F    -1.1137681    0.4148190    2.8657420
C    -2.3168342    0.3480998    3.5158125
C    -2.4856685    0.8847236    4.7714691
C    -3.3530132   -0.2825607    2.8559485
C    -3.7422957    0.7714790    5.3630775
F    -3.0854477   -0.7828807    1.6124086
C    -4.5988706   -0.4027656    3.4277475
C    -4.7857942    0.1351184    4.6994451
H    -1.6598915    1.3763999    5.2651336
H    -3.9019568    1.1869779    6.3473913
H    -5.3938473   -0.9000232    2.8910526
H    -5.7555069    0.0558077    5.1688463

SCF energy GEOOPT =  -1321.432996457 H
ZPE =   585.7 kJ/mol
FREEH energy =   644.08 kJ/mol
FREEH entropy =   0.76469 kJ/mol/K

$vibrational spectrum
#  mode     symmetry     wave number   IR intensity    selection rules
#                         cm**(-1)       km/mol          IR     RAMAN
     1                      -0.00         0.00000         -       -
     2                      -0.00         0.00000         -       -
     3                      -0.00         0.00000         -       -
     4                       0.00         0.00000         -       -
     5                       0.00         0.00000         -       -
     6                       0.00         0.00000         -       -
     7         a             3.19         1.00647        YES     YES
     8         a             9.83         0.89019        YES     YES
     9         a            11.01         0.57353        YES     YES
    10         a            12.04         1.19249        YES     YES
    11         a            17.69         2.44751        YES     YES
    12         a            23.37         1.45654        YES     YES
    13         a            30.48         0.39223        YES     YES
    14         a            45.25         2.66682        YES     YES
    15         a            59.63         2.51160        YES     YES
    16         a            78.24         5.39238        YES     YES
    17         a            81.68         2.97547        YES     YES
    18         a           119.50         2.05585        YES     YES
    19         a           119.78         4.08201        YES     YES
    20         a           126.39         1.98006        YES     YES
    21         a           192.43        12.76989        YES     YES
    22         a           200.62         3.77413        YES     YES
    23         a           202.87         1.08139        YES     YES
    24         a           224.74        24.69155        YES     YES
    25         a           291.98         0.04313        YES     YES
    26         a           325.77         1.08827        YES     YES
    27         a           385.41        83.17607        YES     YES
```

28	a	395.68	5.77017	YES	YES
29	a	401.58	89.78922	YES	YES
30	a	446.44	0.86528	YES	YES
31	a	454.20	5.20261	YES	YES
32	a	479.21	17.58002	YES	YES
33	a	482.91	2.54259	YES	YES
34	a	520.18	154.72298	YES	YES
35	a	557.65	4.89775	YES	YES
36	a	559.07	1.19678	YES	YES
37	a	559.21	15.29064	YES	YES
38	a	578.53	21.98228	YES	YES
39	a	584.22	73.00354	YES	YES
40	a	714.13	0.00532	YES	YES
41	a	755.66	30.24297	YES	YES
42	a	768.40	194.18395	YES	YES
43	a	773.53	25.42107	YES	YES
44	a	775.00	94.06251	YES	YES
45	a	778.85	26.52665	YES	YES
46	a	779.25	10.46309	YES	YES
47	a	829.11	13.91196	YES	YES
48	a	829.84	18.07330	YES	YES
49	a	847.86	20.44086	YES	YES
50	a	874.51	0.91021	YES	YES
51	a	876.42	15.38903	YES	YES
52	a	877.66	18.47040	YES	YES
53	a	935.70	24.05552	YES	YES
54	a	940.71	55.21379	YES	YES
55	a	969.03	3.86166	YES	YES
56	a	1012.99	0.04806	YES	YES
57	a	1019.06	182.44775	YES	YES
58	a	1022.10	133.26430	YES	YES
59	a	1038.19	0.16459	YES	YES
60	a	1040.93	2.16728	YES	YES
61	a	1043.24	4.91276	YES	YES
62	a	1095.40	147.82115	YES	YES
63	a	1101.40	192.03784	YES	YES
64	a	1117.50	25.87677	YES	YES
65	a	1141.46	59.02075	YES	YES
66	a	1144.94	63.26366	YES	YES
67	a	1180.74	14.83230	YES	YES
68	a	1183.99	3.34649	YES	YES
69	a	1205.32	194.69146	YES	YES
70	a	1206.62	189.77621	YES	YES
71	a	1246.40	38.11650	YES	YES
72	a	1247.07	69.48879	YES	YES
73	a	1271.25	141.30046	YES	YES
74	a	1294.53	0.15872	YES	YES
75	a	1341.25	6.41195	YES	YES
76	a	1365.27	23.43846	YES	YES
77	a	1365.87	18.01437	YES	YES
78	a	1391.10	42.36403	YES	YES
79	a	1392.15	57.90142	YES	YES
80	a	1428.60	54.40678	YES	YES
81	a	1430.43	46.40328	YES	YES
82	a	1500.33	9.49906	YES	YES
83	a	1504.95	16.50278	YES	YES
84	a	1505.31	16.73599	YES	YES
85	a	1533.23	179.74530	YES	YES
86	a	1637.84	1.95486	YES	YES
87	a	1662.97	11.28997	YES	YES
88	a	1837.29	1780.95490	YES	YES
89	a	1858.38	180.83674	YES	YES
90	a	3097.43	4.82902	YES	YES

91	a	3097.61	3.95271	YES	YES
92	a	3144.16	5.94348	YES	YES
93	a	3144.76	6.34394	YES	YES
94	a	3165.86	0.43857	YES	YES
95	a	3166.12	0.48030	YES	YES
96	a	3197.15	0.99789	YES	YES
97	a	3206.17	2.07735	YES	YES
98	a	3212.55	1.37159	YES	YES
99	a	3217.71	0.09464	YES	YES

$end

Total COSMO energy + OC corr. = -1321.4993068103 H

[Li(DME)(*o*-DFB)$_2$]$^+$ (F$_{o\text{-DFB}}$-coordination)

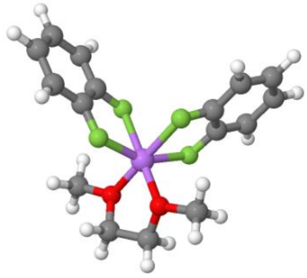

Method: (RI-)B3LYP(D3BJ)/def2-TZVPP
Symmetry: c2

Cartesian coordinates in Ångström:

C	-2.0013483	0.6360503	-1.4181419
C	-1.7534123	1.8483102	-0.8033341
C	-2.3607104	3.0061880	-1.2353486
H	-2.1493178	3.9424494	-0.7391189
C	-3.2373725	2.9223161	-2.3145369
H	-3.7252287	3.8181453	-2.6701133
C	-3.4870203	1.7020212	-2.9330529
H	-4.1689540	1.6493361	-3.7691983
C	-2.8652469	0.5385508	-2.4859594
H	-3.0421944	-0.4212717	-2.9496701
F	-1.3594521	-0.4627854	-0.9239411
F	-0.8847922	1.8542003	0.2494132
C	2.3607104	-3.0061880	-1.2353486
H	2.1493178	-3.9424494	-0.7391189
C	3.2373725	-2.9223161	-2.3145369
H	3.7252287	-3.8181453	-2.6701133
C	3.4870203	-1.7020212	-2.9330529
H	4.1689540	-1.6493361	-3.7691983
C	2.8652469	-0.5385508	-2.4859594
H	3.0421944	0.4212717	-2.9496701
C	2.0013483	-0.6360503	-1.4181419
C	1.7534123	-1.8483102	-0.8033341
F	1.3594521	0.4627854	-0.9239411
F	0.8847922	-1.8542003	0.2494132
Li	0.0000000	0.0000000	0.7212672
H	-3.0192750	-1.2480691	2.8941557
H	-2.8807909	-1.1178570	1.1271801
C	-2.6408081	-0.6388087	2.0717287
H	-3.0996566	0.3524356	2.1118752
O	-1.2182395	-0.5388606	2.1449706
H	-1.1566731	-0.5012477	4.2215362
C	-0.7527961	0.0434044	3.3641451
H	-1.0829921	1.0854968	3.4203599

```
C     0.7527961   -0.0434044    3.3641451
H     1.0829921   -1.0854968    3.4203599
O     1.2182395    0.5388606    2.1449706
H     1.1566731    0.5012477    4.2215362
C     2.6408081    0.6388087    2.0717287
H     3.0996566   -0.3524356    2.1118752
H     2.8807909    1.1178570    1.1271801
H     3.0192750    1.2480691    2.8941557

SCF energy GEOOPT =  -1177.675693745 H
ZPE =   827.3 kJ/mol
FREEH energy =   891.91 kJ/mol
FREEH entropy =   0.73817 kJ/mol/K

$vibrational spectrum
#  mode     symmetry     wave number   IR intensity    selection rules
#                         cm**(-1)        km/mol          IR     RAMAN
     1                      -0.00         0.00000         -        -
     2                       0.00         0.00000         -        -
     3                       0.00         0.00000         -        -
     4                       0.00         0.00000         -        -
     5                       0.00         0.00000         -        -
     6                       0.00         0.00000         -        -
     7         a            10.87         0.10657        YES      YES
     8         b            17.50         0.95064        YES      YES
     9         a            24.71         0.00150        YES      YES
    10         b            33.55         0.42269        YES      YES
    11         b            43.49         0.61890        YES      YES
    12         a            45.28         0.31188        YES      YES
    13         b            62.13         1.64006        YES      YES
    14         b            71.11         0.97483        YES      YES
    15         a            72.54         0.87514        YES      YES
    16         a            90.59         0.22512        YES      YES
    17         b            92.62         3.37980        YES      YES
    18         a            97.30         5.69583        YES      YES
    19         b           132.66         1.60448        YES      YES
    20         a           135.06         0.15067        YES      YES
    21         a           180.00         0.10215        YES      YES
    22         b           201.13         0.08123        YES      YES
    23         a           205.90         0.09939        YES      YES
    24         a           218.61         0.01509        YES      YES
    25         b           219.58         0.66995        YES      YES
    26         b           285.66        71.61423        YES      YES
    27         a           286.79         0.89479        YES      YES
    28         b           291.89         0.44042        YES      YES
    29         a           294.45         0.13138        YES      YES
    30         b           313.88        12.39008        YES      YES
    31         a           321.03         5.26346        YES      YES
    32         b           344.42        23.44220        YES      YES
    33         a           347.23        16.75726        YES      YES
    34         b           412.67       106.09061        YES      YES
    35         a           445.26         0.81549        YES      YES
    36         b           446.92         0.59737        YES      YES
    37         a           456.56        10.17209        YES      YES
    38         b           456.73         2.81790        YES      YES
    39         a           467.87       137.23234        YES      YES
    40         b           556.95         8.36960        YES      YES
    41         a           557.06         3.21037        YES      YES
    42         b           559.26         0.00717        YES      YES
    43         a           559.65         0.04876        YES      YES
    44         b           577.10         6.25169        YES      YES
    45         b           579.36        22.56963        YES      YES
    46         a           580.42        14.25279        YES      YES
```

47	b	716.70	0.01303	YES	YES
48	a	716.89	0.00091	YES	YES
49	b	771.39	91.30185	YES	YES
50	a	775.19	39.17706	YES	YES
51	b	775.62	90.78568	YES	YES
52	a	776.12	74.97832	YES	YES
53	b	845.36	3.25172	YES	YES
54	a	849.13	9.64198	YES	YES
55	b	851.42	27.73496	YES	YES
56	b	876.05	0.00059	YES	YES
57	a	876.40	0.00333	YES	YES
58	a	880.56	54.84910	YES	YES
59	b	970.25	3.38200	YES	YES
60	a	970.37	2.80601	YES	YES
61	b	1014.50	0.00124	YES	YES
62	a	1014.55	0.00000	YES	YES
63	a	1028.46	17.92779	YES	YES
64	b	1043.59	7.98020	YES	YES
65	a	1044.71	3.42012	YES	YES
66	b	1044.72	5.35823	YES	YES
67	b	1097.16	300.12959	YES	YES
68	a	1118.74	7.50574	YES	YES
69	b	1120.67	41.40886	YES	YES
70	a	1126.06	18.07574	YES	YES
71	a	1134.68	46.13738	YES	YES
72	b	1181.15	2.63514	YES	YES
73	a	1182.58	10.51076	YES	YES
74	b	1184.34	4.47454	YES	YES
75	a	1184.44	1.98601	YES	YES
76	b	1187.67	25.91840	YES	YES
77	a	1188.32	0.90987	YES	YES
78	a	1214.47	17.65076	YES	YES
79	b	1234.48	0.30319	YES	YES
80	b	1267.62	14.20868	YES	YES
81	b	1270.42	212.17242	YES	YES
82	a	1278.20	110.82854	YES	YES
83	a	1294.34	0.06538	YES	YES
84	b	1294.50	0.15305	YES	YES
85	a	1304.04	8.49516	YES	YES
86	b	1340.21	6.02485	YES	YES
87	a	1340.53	3.68317	YES	YES
88	b	1403.55	17.09620	YES	YES
89	a	1439.59	2.84555	YES	YES
90	b	1483.52	1.01690	YES	YES
91	a	1484.28	0.07174	YES	YES
92	b	1495.08	21.73597	YES	YES
93	a	1495.41	9.87189	YES	YES
94	a	1499.89	6.35148	YES	YES
95	b	1499.94	17.15036	YES	YES
96	b	1501.39	9.61887	YES	YES
97	a	1503.48	4.41522	YES	YES
98	b	1516.52	7.05420	YES	YES
99	a	1518.23	7.79023	YES	YES
100	b	1533.47	234.04557	YES	YES
101	a	1536.22	144.94074	YES	YES
102	a	1637.68	0.98697	YES	YES
103	b	1638.88	1.48157	YES	YES
104	b	1661.07	15.14042	YES	YES
105	a	1661.37	9.33720	YES	YES
106	a	3013.06	17.14291	YES	YES
107	b	3015.21	12.01997	YES	YES
108	b	3020.77	67.77773	YES	YES
109	a	3020.87	2.87720	YES	YES

110	a	3059.64	25.43230	YES	YES
111	b	3068.69	30.94173	YES	YES
112	a	3084.44	10.91447	YES	YES
113	b	3084.60	29.42837	YES	YES
114	b	3149.94	2.23133	YES	YES
115	a	3150.02	16.50372	YES	YES
116	a	3197.10	0.38933	YES	YES
117	b	3197.10	1.46391	YES	YES
118	b	3205.27	2.99597	YES	YES
119	a	3205.29	1.74007	YES	YES
120	b	3211.39	1.54506	YES	YES
121	a	3211.40	0.33677	YES	YES
122	a	3217.34	0.02589	YES	YES
123	b	3217.34	0.07851	YES	YES

$end

Total COSMO energy + OC corr. = -1177.7290992698 H

[Li(*o*-DFB)$_2$(FEC)]$^+$ (F$_{o\text{-DFB}}$-coordination)

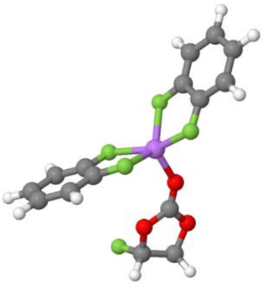

Method: (RI-)B3LYP(D3BJ)/def2-TZVPP
Symmetry: c1

Cartesian coordinates in Ångström:

C	0.0289491	0.2550258	1.8531638
C	-0.1422890	-1.0593346	1.4677600
C	-0.9374127	-1.9187298	2.1907371
H	-1.0570747	-2.9435345	1.8702653
C	-1.5687933	-1.4185061	3.3278272
H	-2.2007015	-2.0722785	3.9111450
C	-1.3965039	-0.0933872	3.7136036
H	-1.8950639	0.2814918	4.5955635
C	-0.5869673	0.7652137	2.9721031
H	-0.4386625	1.7989191	3.2492071
F	0.8446968	1.0291131	1.0681871
F	0.5147977	-1.4644316	0.3408690
C	1.2178777	0.5633570	-4.5001700
H	0.7244130	-0.3204686	-4.8776639
C	1.5740838	1.6262862	-5.3283577
H	1.3513708	1.5699387	-6.3838535
C	2.2092633	2.7504933	-4.8114868
H	2.4791845	3.5661121	-5.4663277
C	2.5057085	2.8424334	-3.4527907
H	2.9987021	3.7049659	-3.0283220
C	2.1479013	1.7855159	-2.6483649
C	1.5174683	0.6705223	-3.1623513
F	2.3975969	1.7850563	-1.3019658
F	1.2047072	-0.3248689	-2.2705505
Li	1.8466651	0.0278836	-0.3828457
F	3.1333949	-3.4382987	3.2746188
H	5.4647143	-3.5837757	2.4811969

```
O     4.9842898   -2.0166116    1.2177526
C     3.8522873   -1.3348960    1.1991501
O     3.4208396   -0.7436738    0.2433548
C     5.2170921   -2.5289946    2.5495867
C     3.8790987   -2.2969211    3.2471635
O     3.2276913   -1.3647853    2.3954602
H     3.9223568   -1.8863075    4.2515439
H     6.0314778   -1.9654338    2.9991115

SCF energy GEOOPT =  -1310.475244865 H
ZPE =   629.7 kJ/mol
FREEH energy =   689.16 kJ/mol
FREEH entropy =   0.74395 kJ/mol/K

$vibrational spectrum
#  mode     symmetry     wave number   IR intensity    selection rules
#                         cm**(-1)        km/mol          IR     RAMAN
     1                      -0.00         0.00000         -        -
     2                      -0.00         0.00000         -        -
     3                       0.00         0.00000         -        -
     4                       0.00         0.00000         -        -
     5                       0.00         0.00000         -        -
     6                       0.00         0.00000         -        -
     7         a             6.26         0.25298        YES      YES
     8         a            12.25         0.06796        YES      YES
     9         a            14.75         0.83385        YES      YES
    10         a            18.64         0.38072        YES      YES
    11         a            22.96         0.77872        YES      YES
    12         a            29.96         0.46629        YES      YES
    13         a            37.50         2.77124        YES      YES
    14         a            63.85         1.75760        YES      YES
    15         a            74.18         1.39459        YES      YES
    16         a            75.56         2.18162        YES      YES
    17         a            82.20         0.89428        YES      YES
    18         a           112.16         3.78496        YES      YES
    19         a           124.06         2.31443        YES      YES
    20         a           197.69         1.64272        YES      YES
    21         a           198.90         0.01481        YES      YES
    22         a           205.56         1.81299        YES      YES
    23         a           249.92        31.36504        YES      YES
    24         a           289.69         1.10759        YES      YES
    25         a           292.32         0.09936        YES      YES
    26         a           326.07         0.80824        YES      YES
    27         a           330.97         1.08072        YES      YES
    28         a           379.88       163.85669        YES      YES
    29         a           398.49        53.80358        YES      YES
    30         a           444.42         6.82893        YES      YES
    31         a           449.52         4.08984        YES      YES
    32         a           452.33         6.73002        YES      YES
    33         a           453.53         9.47451        YES      YES
    34         a           474.76       194.17507        YES      YES
    35         a           483.34         1.77759        YES      YES
    36         a           557.54         0.11811        YES      YES
    37         a           557.79         1.91938        YES      YES
    38         a           558.67         3.89744        YES      YES
    39         a           559.21         4.79522        YES      YES
    40         a           573.18        13.17858        YES      YES
    41         a           578.25        50.99618        YES      YES
    42         a           579.09        15.46636        YES      YES
    43         a           713.71         0.00096        YES      YES
    44         a           714.08         0.00785        YES      YES
    45         a           757.42        81.55012        YES      YES
    46         a           771.01       123.19579        YES      YES
```

47	a	774.47	46.19221	YES	YES
48	a	775.05	28.48235	YES	YES
49	a	775.29	105.60496	YES	YES
50	a	779.27	12.07975	YES	YES
51	a	829.39	12.26591	YES	YES
52	a	844.09	4.03306	YES	YES
53	a	848.70	32.51025	YES	YES
54	a	874.68	1.66301	YES	YES
55	a	875.23	0.10796	YES	YES
56	a	876.45	15.71390	YES	YES
57	a	936.24	28.47922	YES	YES
58	a	969.55	3.03592	YES	YES
59	a	970.14	3.94534	YES	YES
60	a	1014.04	0.16404	YES	YES
61	a	1014.74	0.00070	YES	YES
62	a	1018.78	153.92166	YES	YES
63	a	1039.88	1.53248	YES	YES
64	a	1042.09	5.00234	YES	YES
65	a	1042.82	4.74246	YES	YES
66	a	1095.58	160.35501	YES	YES
67	a	1113.82	5.19287	YES	YES
68	a	1118.38	45.50252	YES	YES
69	a	1141.47	59.85605	YES	YES
70	a	1173.35	2.17936	YES	YES
71	a	1182.55	29.80140	YES	YES
72	a	1184.31	4.09271	YES	YES
73	a	1184.47	4.36754	YES	YES
74	a	1207.05	203.67732	YES	YES
75	a	1246.64	52.90216	YES	YES
76	a	1266.07	291.79500	YES	YES
77	a	1273.13	36.61585	YES	YES
78	a	1294.46	0.08658	YES	YES
79	a	1294.77	0.42793	YES	YES
80	a	1341.65	12.62913	YES	YES
81	a	1342.03	4.09473	YES	YES
82	a	1365.25	22.60893	YES	YES
83	a	1391.40	49.42350	YES	YES
84	a	1429.50	55.08005	YES	YES
85	a	1500.44	2.14481	YES	YES
86	a	1500.79	16.29126	YES	YES
87	a	1504.79	16.31258	YES	YES
88	a	1530.86	326.41026	YES	YES
89	a	1534.10	54.70729	YES	YES
90	a	1635.68	0.49945	YES	YES
91	a	1638.11	2.40427	YES	YES
92	a	1664.12	14.32672	YES	YES
93	a	1664.99	8.26972	YES	YES
94	a	1845.28	875.68024	YES	YES
95	a	3098.26	3.93780	YES	YES
96	a	3145.05	5.58691	YES	YES
97	a	3166.60	0.42904	YES	YES
98	a	3197.54	1.00248	YES	YES
99	a	3198.40	1.01193	YES	YES
100	a	3206.55	2.03741	YES	YES
101	a	3207.43	1.91051	YES	YES
102	a	3212.92	1.67770	YES	YES
103	a	3213.79	1.93969	YES	YES
104	a	3217.98	0.13287	YES	YES
105	a	3218.73	0.17172	YES	YES

$end

Total COSMO energy + OC corr. = -1310.5345420141 H

[Li(*o*-DFB)$_2$(FEC)$_2$]$^+$ (F$_{o\text{-DFB}}$-coordination)

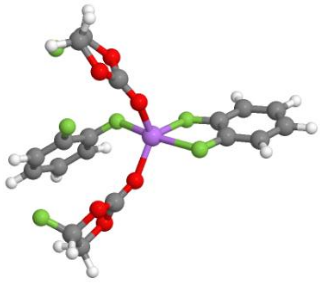

Method: (RI-)B3LYP(D3BJ)/def2-TZVPP
Symmetry: c1

Cartesian coordinates in Ångström:

C	1.5397841	-3.1309492	2.1745840
C	2.0422456	-4.3464120	1.7396404
C	1.3130066	-5.5063196	1.9166127
H	1.7224814	-6.4428669	1.5672810
C	0.0702773	-5.4314842	2.5377189
H	-0.5026613	-6.3353403	2.6881986
C	-0.4294254	-4.2084198	2.9714769
H	-1.3933666	-4.1558702	3.4565086
C	0.3089287	-3.0435941	2.7888179
H	-0.0549408	-2.0789355	3.1121074
F	2.2783954	-2.0071471	1.9676171
F	3.2408768	-4.3792620	1.1244280
C	3.3554662	2.4966012	-2.0594069
H	3.5911471	2.2849946	-3.0923672
C	3.3187259	3.7962934	-1.5594578
H	3.5312956	4.6231916	-2.2213787
C	3.0126061	4.0361674	-0.2242760
H	2.9871491	5.0488350	0.1509947
C	2.7364761	2.9807179	0.6421204
H	2.4961163	3.1397522	1.6833860
C	2.7768027	1.7028328	0.1327919
C	3.0802328	1.4617858	-1.1931497
F	2.5187893	0.6145985	0.9199571
F	3.0945577	0.1643990	-1.6022811
Li	2.8148498	-1.2223341	0.0306642
F	5.4582159	-4.1620470	3.3425081
H	7.0091245	-4.8542123	1.5624976
O	6.2362963	-3.3000786	0.4384508
C	5.3501650	-2.4022636	0.8469166
O	4.6034667	-1.8000961	0.1238289
C	7.0043693	-3.7684886	1.5661378
C	6.2432341	-3.2053470	2.7641198
O	5.4014372	-2.2255615	2.1843759
H	6.8433594	-2.7408397	3.5411179
H	8.0152563	-3.3748828	1.4860805
O	-0.4445735	-3.4567893	-0.8411772
H	-1.2478816	-5.3596052	-0.7858675
C	-0.8076604	-4.6815020	-1.5106173
H	-1.5128533	-4.4474776	-2.3047532
O	1.3791849	-2.1632795	-0.7415757
C	0.8166549	-3.1535563	-1.1207109
C	0.5285566	-5.2027500	-2.0376101
O	1.3939906	-4.0900122	-1.8996540
F	1.0024894	-6.2181458	-1.2560648
H	0.5426913	-5.5350494	-3.0714716

```
SCF energy GEOOPT =  -1752.151812630 H
ZPE =   809.0 kJ/mol
FREEH energy =   888.24 kJ/mol
FREEH entropy =   0.91407 kJ/mol/K

$vibrational spectrum
#  mode     symmetry     wave number   IR intensity    selection rules
#                         cm**(-1)        km/mol          IR     RAMAN
     1                      -0.00         0.00000         -        -
     2                      -0.00         0.00000         -        -
     3                      -0.00         0.00000         -        -
     4                      -0.00         0.00000         -        -
     5                       0.00         0.00000         -        -
     6                       0.00         0.00000         -        -
     7         a             7.71         0.33821        YES      YES
     8         a             9.19         1.59437        YES      YES
     9         a            14.29         0.78824        YES      YES
    10         a            19.63         2.31134        YES      YES
    11         a            21.55         0.88480        YES      YES
    12         a            24.68         0.19032        YES      YES
    13         a            26.50         1.58939        YES      YES
    14         a            32.53         2.65949        YES      YES
    15         a            38.86         0.33692        YES      YES
    16         a            48.30         0.25575        YES      YES
    17         a            56.99         0.28778        YES      YES
    18         a            60.31         1.36686        YES      YES
    19         a            63.36         3.02978        YES      YES
    20         a            67.91         1.11893        YES      YES
    21         a            75.84         2.37131        YES      YES
    22         a            76.33         2.89387        YES      YES
    23         a           101.02         2.98985        YES      YES
    24         a           113.05         2.74647        YES      YES
    25         a           125.40         4.06119        YES      YES
    26         a           134.90         3.27120        YES      YES
    27         a           193.16        31.17536        YES      YES
    28         a           198.24         0.16593        YES      YES
    29         a           202.74        10.62753        YES      YES
    30         a           210.84         6.57595        YES      YES
    31         a           230.46        62.73199        YES      YES
    32         a           290.77         0.12000        YES      YES
    33         a           293.44         1.20151        YES      YES
    34         a           302.54         0.63542        YES      YES
    35         a           322.35         2.39942        YES      YES
    36         a           385.62       187.97362        YES      YES
    37         a           393.33         9.89128        YES      YES
    38         a           395.84        33.35148        YES      YES
    39         a           446.36         2.39382        YES      YES
    40         a           447.80         2.79699        YES      YES
    41         a           454.61         8.41978        YES      YES
    42         a           466.57         7.92237        YES      YES
    43         a           473.78        69.35060        YES      YES
    44         a           481.71         2.46475        YES      YES
    45         a           492.38        73.34284        YES      YES
    46         a           551.93         5.11344        YES      YES
    47         a           555.99         1.57890        YES      YES
    48         a           558.23         5.12374        YES      YES
    49         a           560.23         0.33894        YES      YES
    50         a           565.04         0.56125        YES      YES
    51         a           568.20        16.75644        YES      YES
    52         a           579.65        24.74451        YES      YES
    53         a           584.43         8.91154        YES      YES
    54         a           716.49         0.00250        YES      YES
    55         a           719.79         0.12841        YES      YES
```

56	a	754.73	63.19746	YES	YES
57	a	759.21	41.24926	YES	YES
58	a	772.47	60.69693	YES	YES
59	a	773.64	33.42643	YES	YES
60	a	774.37	82.48664	YES	YES
61	a	776.05	50.89110	YES	YES
62	a	777.32	109.42602	YES	YES
63	a	779.77	18.28110	YES	YES
64	a	821.47	26.03668	YES	YES
65	a	826.98	26.36061	YES	YES
66	a	850.78	15.87586	YES	YES
67	a	859.24	15.84598	YES	YES
68	a	873.98	1.67489	YES	YES
69	a	874.11	6.83818	YES	YES
70	a	875.61	19.57503	YES	YES
71	a	876.64	11.02446	YES	YES
72	a	936.76	21.59486	YES	YES
73	a	939.39	18.29104	YES	YES
74	a	967.10	2.09739	YES	YES
75	a	967.36	5.69780	YES	YES
76	a	1007.72	0.68250	YES	YES
77	a	1009.22	0.00376	YES	YES
78	a	1021.50	124.72821	YES	YES
79	a	1024.82	168.71371	YES	YES
80	a	1043.85	5.32428	YES	YES
81	a	1047.49	12.34177	YES	YES
82	a	1047.54	4.80924	YES	YES
83	a	1049.72	5.00030	YES	YES
84	a	1097.66	163.00226	YES	YES
85	a	1098.96	141.25407	YES	YES
86	a	1119.85	25.91589	YES	YES
87	a	1124.88	28.17602	YES	YES
88	a	1139.15	104.44026	YES	YES
89	a	1140.10	48.91969	YES	YES
90	a	1182.53	2.41330	YES	YES
91	a	1183.36	6.29413	YES	YES
92	a	1185.71	15.30252	YES	YES
93	a	1197.44	112.46249	YES	YES
94	a	1198.83	80.72638	YES	YES
95	a	1205.98	160.48291	YES	YES
96	a	1244.78	21.60541	YES	YES
97	a	1245.14	55.91155	YES	YES
98	a	1275.80	203.56639	YES	YES
99	a	1285.33	106.20344	YES	YES
100	a	1294.40	1.95291	YES	YES
101	a	1295.39	20.10991	YES	YES
102	a	1337.28	0.34161	YES	YES
103	a	1341.43	7.59291	YES	YES
104	a	1366.10	29.39210	YES	YES
105	a	1366.15	3.12748	YES	YES
106	a	1390.49	22.23235	YES	YES
107	a	1390.83	50.17545	YES	YES
108	a	1427.07	19.10503	YES	YES
109	a	1427.94	47.74236	YES	YES
110	a	1497.52	12.52899	YES	YES
111	a	1500.08	9.88481	YES	YES
112	a	1504.39	14.75082	YES	YES
113	a	1505.38	14.47509	YES	YES
114	a	1535.06	252.34939	YES	YES
115	a	1540.93	147.57213	YES	YES
116	a	1638.71	1.90123	YES	YES
117	a	1641.23	2.65063	YES	YES
118	a	1654.39	16.92536	YES	YES

119	a	1662.12	16.94884	YES	YES
120	a	1844.57	969.81011	YES	YES
121	a	1865.21	683.60929	YES	YES
122	a	3095.23	6.28029	YES	YES
123	a	3096.76	6.32044	YES	YES
124	a	3140.97	9.56678	YES	YES
125	a	3142.05	8.23720	YES	YES
126	a	3162.57	1.09571	YES	YES
127	a	3163.13	0.82519	YES	YES
128	a	3192.10	0.97962	YES	YES
129	a	3196.07	0.91318	YES	YES
130	a	3202.90	3.26282	YES	YES
131	a	3205.36	2.88335	YES	YES
132	a	3211.43	0.28691	YES	YES
133	a	3211.97	0.52763	YES	YES
134	a	3215.69	0.07773	YES	YES
135	a	3217.13	0.00113	YES	YES

$end

Total COSMO energy + OC corr. = -1752.2138247603 H

[Li(*o*-DFB)(FEC)$_3$]$^+$ (F$_{o\text{-DFB}}$-coordination)

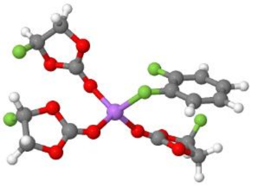

Method: (RI-)B3LYP(D3BJ)/def2-TZVPP
Symmetry: c1

Cartesian coordinates in Ångström:

O	2.3232386	-1.6935246	2.2026423
C	1.7786809	-0.7074960	1.4477053
O	0.6021566	-0.6041634	1.2356593
O	2.7092359	0.1228852	0.9958792
C	3.9999082	-0.2709795	1.5089692
C	3.7353394	-1.6626923	2.0756933
H	4.3044733	0.4433592	2.2709791
H	4.7120435	-0.2926851	0.6898853
H	4.1804874	-1.8886701	3.0396156
F	4.1075989	-2.6292756	1.1881433
F	3.1667229	-4.4345585	3.7523415
H	1.4757549	-4.2251058	5.5342488
O	0.1500094	-3.8529309	3.9897532
C	0.3714039	-3.9836414	2.6821721
O	-0.2390185	-3.4193788	1.8205602
C	1.0089427	-4.7582938	4.7119462
C	2.0184807	-5.1826098	3.6517364
O	1.3769544	-4.8531432	2.4392848
H	2.2988311	-6.2312115	3.6363166
H	0.4122833	-5.5911978	5.0786639
Li	-0.9468935	-1.7395899	1.2950802
F	-2.0318476	-0.9497776	2.8299012
C	-3.3913777	-0.8292394	2.7762701
C	-3.9785109	0.4109185	2.8922813
C	-4.1438435	-1.9747463	2.5738219
C	-5.3646845	0.4989373	2.8010136
F	-3.5131000	-3.1542674	2.4541392
C	-5.5196418	-1.8907613	2.4746967

```
C    -6.1279474   -0.6443109    2.5913349
H    -3.3580315    1.2822672    3.0445836
H    -5.8432879    1.4627878    2.8974040
H    -6.0913506   -2.7922140    2.3081432
H    -7.2042123   -0.5726169    2.5244759
H    -5.5258083   -2.1025113   -2.6190591
F    -6.1838951   -2.4328377   -0.7655765
C    -5.2774957   -1.8356145   -1.5960644
O    -3.9903160   -2.3379880   -1.2881058
C    -3.2997436   -1.4231449   -0.5795122
C    -5.1591098   -0.3431460   -1.2927266
O    -2.2365355   -1.6433435   -0.0704420
H    -5.0525192    0.2629384   -2.1895130
H    -5.9810407    0.0101267   -0.6775268
O    -3.9386343   -0.2582227   -0.5303148

SCF energy GEOOPT =  -1763.107953993 H
ZPE =   765.3 kJ/mol
FREEH energy =   842.73 kJ/mol
FREEH entropy =   0.91297 kJ/mol/K

$vibrational spectrum
#  mode     symmetry     wave number   IR intensity    selection rules
#                         cm**(-1)        km/mol          IR     RAMAN
     1                      -0.00          0.00000         -       -
     2                      -0.00          0.00000         -       -
     3                      -0.00          0.00000         -       -
     4                      -0.00          0.00000         -       -
     5                       0.00          0.00000         -       -
     6                       0.00          0.00000         -       -
     7         a             5.92          2.29959        YES     YES
     8         a             8.02          1.96677        YES     YES
     9         a            11.87          0.07794        YES     YES
    10         a            19.15          3.35823        YES     YES
    11         a            21.07          0.14142        YES     YES
    12         a            29.37          3.88985        YES     YES
    13         a            32.16          0.95673        YES     YES
    14         a            37.37          2.03882        YES     YES
    15         a            40.41          0.47731        YES     YES
    16         a            42.94          0.38420        YES     YES
    17         a            44.43          1.25775        YES     YES
    18         a            50.04          0.39289        YES     YES
    19         a            55.67          0.92521        YES     YES
    20         a            61.12          1.09798        YES     YES
    21         a            66.52          1.65108        YES     YES
    22         a            78.20          4.11862        YES     YES
    23         a           103.66          9.03764        YES     YES
    24         a           121.62          1.39044        YES     YES
    25         a           127.35          4.85490        YES     YES
    26         a           135.58          2.32329        YES     YES
    27         a           147.32          3.28456        YES     YES
    28         a           195.71          6.07305        YES     YES
    29         a           199.19          0.33742        YES     YES
    30         a           206.75          0.33539        YES     YES
    31         a           213.05          1.42862        YES     YES
    32         a           292.19          0.37326        YES     YES
    33         a           299.22         34.69851        YES     YES
    34         a           312.86         59.90688        YES     YES
    35         a           388.62         38.93788        YES     YES
    36         a           394.18          9.37993        YES     YES
    37         a           398.61          6.71656        YES     YES
    38         a           410.98         61.27173        YES     YES
    39         a           446.25          1.86715        YES     YES
```

40	a	464.05	30.32724	YES	YES
41	a	469.50	103.99802	YES	YES
42	a	483.80	1.38171	YES	YES
43	a	492.05	9.14535	YES	YES
44	a	522.08	170.47043	YES	YES
45	a	548.46	0.42356	YES	YES
46	a	548.95	7.88491	YES	YES
47	a	552.55	6.53364	YES	YES
48	a	566.70	0.66004	YES	YES
49	a	567.60	7.85346	YES	YES
50	a	583.46	13.95663	YES	YES
51	a	719.26	0.05958	YES	YES
52	a	750.31	42.57295	YES	YES
53	a	753.56	23.20716	YES	YES
54	a	758.53	112.22116	YES	YES
55	a	772.95	21.93931	YES	YES
56	a	775.26	36.35855	YES	YES
57	a	775.85	16.88623	YES	YES
58	a	777.36	89.63249	YES	YES
59	a	778.24	32.63506	YES	YES
60	a	824.97	26.56833	YES	YES
61	a	832.25	13.14937	YES	YES
62	a	837.10	17.41339	YES	YES
63	a	859.17	17.30198	YES	YES
64	a	871.84	26.83285	YES	YES
65	a	872.95	6.52565	YES	YES
66	a	876.22	12.62816	YES	YES
67	a	876.48	18.00738	YES	YES
68	a	930.75	9.10104	YES	YES
69	a	932.27	21.63641	YES	YES
70	a	939.09	31.38963	YES	YES
71	a	965.70	4.11282	YES	YES
72	a	1005.28	25.90020	YES	YES
73	a	1007.19	0.33414	YES	YES
74	a	1019.07	305.73990	YES	YES
75	a	1023.78	90.40103	YES	YES
76	a	1044.21	4.58018	YES	YES
77	a	1048.02	9.17910	YES	YES
78	a	1048.57	6.55458	YES	YES
79	a	1051.46	9.42491	YES	YES
80	a	1090.65	59.24625	YES	YES
81	a	1098.36	403.14773	YES	YES
82	a	1100.39	58.30181	YES	YES
83	a	1124.16	29.12015	YES	YES
84	a	1129.82	119.63068	YES	YES
85	a	1139.20	73.52213	YES	YES
86	a	1140.06	49.35520	YES	YES
87	a	1182.22	2.18926	YES	YES
88	a	1186.57	210.03090	YES	YES
89	a	1194.26	86.85359	YES	YES
90	a	1195.31	273.48728	YES	YES
91	a	1200.63	29.07391	YES	YES
92	a	1241.46	33.91348	YES	YES
93	a	1243.96	32.49065	YES	YES
94	a	1245.60	50.36795	YES	YES
95	a	1284.81	94.07219	YES	YES
96	a	1297.84	42.85718	YES	YES
97	a	1337.81	1.45376	YES	YES
98	a	1365.04	28.28955	YES	YES
99	a	1366.03	11.00867	YES	YES
100	a	1366.60	18.41621	YES	YES
101	a	1384.29	43.01308	YES	YES
102	a	1386.50	35.11686	YES	YES

103	a	1389.62	36.58162	YES	YES
104	a	1421.06	32.78040	YES	YES
105	a	1424.08	42.62745	YES	YES
106	a	1426.07	38.40057	YES	YES
107	a	1497.43	13.31704	YES	YES
108	a	1503.31	11.53882	YES	YES
109	a	1504.96	15.68163	YES	YES
110	a	1505.68	14.89450	YES	YES
111	a	1540.28	179.75334	YES	YES
112	a	1638.89	5.79614	YES	YES
113	a	1655.74	16.28495	YES	YES
114	a	1844.21	582.17650	YES	YES
115	a	1855.65	1596.93070	YES	YES
116	a	1886.25	353.96192	YES	YES
117	a	3092.37	6.74109	YES	YES
118	a	3094.19	6.11223	YES	YES
119	a	3095.55	7.38162	YES	YES
120	a	3142.57	8.77573	YES	YES
121	a	3149.23	7.55561	YES	YES
122	a	3149.46	5.42516	YES	YES
123	a	3162.27	1.10090	YES	YES
124	a	3162.56	0.82501	YES	YES
125	a	3164.13	0.73674	YES	YES
126	a	3191.34	0.95475	YES	YES
127	a	3202.36	3.22689	YES	YES
128	a	3210.66	0.32279	YES	YES
129	a	3214.71	0.07228	YES	YES

$end

Total COSMO energy + OC corr. = -1763.1768111359 H

[Li(*o*-DFB)(FEC)(DME)]$^+$ ($F_{o\text{-DFB}}$-coordination)

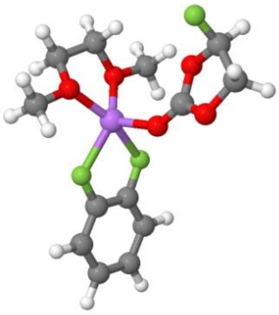

Method: (RI-)B3LYP(D3BJ)/def2-TZVPP
Symmetry: c1

Cartesian coordinates in Ångström:

C	0.7405088	2.1954907	-0.5606800
O	0.9096762	1.0362374	0.2567479
Li	-0.4353892	-0.2820864	-0.2586754
O	-0.6221574	0.7943431	-1.8928437
C	0.4592239	1.7228564	-1.9651999
C	1.3116703	1.3403873	1.5926921
H	1.6479906	2.8048919	-0.5462713
H	-0.0927192	2.7923107	-0.1763749
C	-1.0868305	0.3561490	-3.1697566
H	0.1892785	2.5731953	-2.5973574
H	1.3375783	1.2304377	-2.3948845
H	-1.9022015	-0.3394941	-2.9927817
H	-0.2883540	-0.1478720	-3.7194167

```
H    -1.4501946    1.2057465   -3.7509182
H     1.4143348    0.3965858    2.1198020
H     0.5594147    1.9597743    2.0867561
H     2.2705183    1.8618949    1.5885021
H    -0.7234930   -4.5942798   -2.1012662
F    -0.4769280   -2.1029685   -1.2720757
C    -0.3505645   -4.4844355   -1.0931968
C    -0.1826001   -3.2292006   -0.5545117
H    -0.1487858   -6.5787735   -0.7013315
C    -0.0260637   -5.5831159   -0.3007076
C     0.2915958   -3.0523398    0.7304625
F     0.4244042   -1.7723910    1.1764345
C     0.4522140   -5.4074863    0.9931140
C     0.6162112   -4.1301335    1.5239415
H     0.7014829   -6.2671508    1.5979641
H     0.9868530   -3.9692095    2.5260081
F    -3.6695096    3.5285981    1.0242562
H    -5.4989244    1.9900641    1.6327027
O    -4.1081894    0.4755278    1.4022014
C    -2.8093902    0.6136491    1.1775912
O    -2.1104970   -0.2064629    0.6496342
C    -4.6011764    1.6257700    2.1226341
C    -3.4473663    2.6189972    2.0178682
O    -2.3510941    1.8047190    1.6339998
H    -3.1894686    3.1562131    2.9258428
H    -4.8105880    1.3301107    3.1483444

SCF energy GEOOPT =  -1188.630877334 H
ZPE =   783.2 kJ/mol
FREEH energy =   846.54 kJ/mol
FREEH entropy =   0.75902 kJ/mol/K

$vibrational spectrum
#  mode     symmetry     wave number   IR intensity    selection rules
#                         cm**(-1)        km/mol          IR     RAMAN
     1                        0.00         0.00000         -        -
     2                        0.00         0.00000         -        -
     3                        0.00         0.00000         -        -
     4                        0.00         0.00000         -        -
     5                        0.00         0.00000         -        -
     6                        0.00         0.00000         -        -
     7         a              7.32         0.15121        YES      YES
     8         a             12.32         0.45632        YES      YES
     9         a             19.88         0.73237        YES      YES
    10         a             21.25         0.82267        YES      YES
    11         a             30.37         1.00147        YES      YES
    12         a             46.04         3.13832        YES      YES
    13         a             49.33         0.41726        YES      YES
    14         a             60.96         1.45150        YES      YES
    15         a             66.76         0.76898        YES      YES
    16         a             75.48         2.25188        YES      YES
    17         a             82.54         2.98073        YES      YES
    18         a            105.53         4.94806        YES      YES
    19         a            123.27         4.08493        YES      YES
    20         a            129.37         2.65320        YES      YES
    21         a            144.25         3.76606        YES      YES
    22         a            170.42         0.11340        YES      YES
    23         a            197.99         0.21641        YES      YES
    24         a            203.52         1.12591        YES      YES
    25         a            218.28         0.39960        YES      YES
    26         a            222.52         3.65936        YES      YES
    27         a            285.67         1.19821        YES      YES
    28         a            290.70         0.34692        YES      YES
```

29	a	314.26	14.33031	YES	YES
30	a	333.86	14.53968	YES	YES
31	a	349.27	29.87679	YES	YES
32	a	386.39	47.01252	YES	YES
33	a	396.58	6.87420	YES	YES
34	a	403.88	135.82997	YES	YES
35	a	446.79	4.79135	YES	YES
36	a	454.68	5.04337	YES	YES
37	a	483.11	1.72763	YES	YES
38	a	490.70	112.17264	YES	YES
39	a	557.31	5.04592	YES	YES
40	a	559.55	0.24779	YES	YES
41	a	569.64	13.03765	YES	YES
42	a	577.65	2.48599	YES	YES
43	a	580.02	23.33328	YES	YES
44	a	715.10	0.00060	YES	YES
45	a	752.29	55.62704	YES	YES
46	a	773.76	76.70571	YES	YES
47	a	774.69	71.30953	YES	YES
48	a	777.13	20.43934	YES	YES
49	a	829.30	13.96553	YES	YES
50	a	846.56	14.00977	YES	YES
51	a	850.95	13.01222	YES	YES
52	a	872.98	0.00471	YES	YES
53	a	875.89	17.39932	YES	YES
54	a	882.00	43.67883	YES	YES
55	a	931.38	17.64567	YES	YES
56	a	967.02	3.27528	YES	YES
57	a	1010.49	0.00071	YES	YES
58	a	1016.45	180.09750	YES	YES
59	a	1029.00	29.83408	YES	YES
60	a	1043.34	4.06329	YES	YES
61	a	1044.14	14.26116	YES	YES
62	a	1044.57	4.96633	YES	YES
63	a	1092.41	164.75187	YES	YES
64	a	1098.40	253.81666	YES	YES
65	a	1120.18	44.64852	YES	YES
66	a	1128.43	12.06837	YES	YES
67	a	1135.61	40.48362	YES	YES
68	a	1139.03	76.61488	YES	YES
69	a	1181.83	3.05846	YES	YES
70	a	1183.93	8.21757	YES	YES
71	a	1185.10	20.20737	YES	YES
72	a	1188.53	3.13600	YES	YES
73	a	1198.16	205.27907	YES	YES
74	a	1215.74	20.67880	YES	YES
75	a	1235.38	0.69457	YES	YES
76	a	1245.49	47.86174	YES	YES
77	a	1268.20	15.80162	YES	YES
78	a	1275.09	176.20582	YES	YES
79	a	1294.17	0.92245	YES	YES
80	a	1303.22	8.50433	YES	YES
81	a	1341.20	6.57092	YES	YES
82	a	1365.49	20.84696	YES	YES
83	a	1390.06	43.62266	YES	YES
84	a	1403.42	13.23505	YES	YES
85	a	1424.89	42.68494	YES	YES
86	a	1439.31	2.65706	YES	YES
87	a	1484.19	0.49504	YES	YES
88	a	1485.42	0.29494	YES	YES
89	a	1492.18	17.90113	YES	YES
90	a	1495.70	9.71822	YES	YES
91	a	1499.99	11.17368	YES	YES

92	a	1501.34	8.22900	YES	YES
93	a	1503.35	8.93150	YES	YES
94	a	1505.29	14.10269	YES	YES
95	a	1516.74	5.53515	YES	YES
96	a	1518.72	5.35314	YES	YES
97	a	1535.52	208.84844	YES	YES
98	a	1638.45	2.05494	YES	YES
99	a	1662.82	15.59451	YES	YES
100	a	1857.24	873.54732	YES	YES
101	a	3011.32	18.26650	YES	YES
102	a	3014.44	12.83385	YES	YES
103	a	3021.35	42.58678	YES	YES
104	a	3023.28	31.41120	YES	YES
105	a	3056.43	24.10183	YES	YES
106	a	3066.54	36.91734	YES	YES
107	a	3084.07	19.29760	YES	YES
108	a	3085.00	23.64554	YES	YES
109	a	3095.94	4.75762	YES	YES
110	a	3142.75	6.76916	YES	YES
111	a	3144.93	10.57083	YES	YES
112	a	3151.22	7.97061	YES	YES
113	a	3164.39	0.45717	YES	YES
114	a	3196.76	0.75071	YES	YES
115	a	3205.38	2.62394	YES	YES
116	a	3211.67	0.44609	YES	YES
117	a	3217.37	0.01855	YES	YES

$end

Total COSMO energy + OC corr. = -1188.6900035657 H

[Li(DEC)(*o*-DFB)$_2$]$^+$ (F$_{o\text{-DFB}}$-coordination)

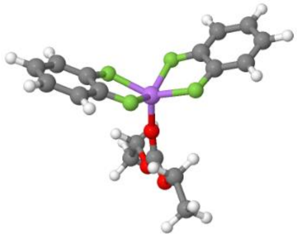

Method: (RI-)B3LYP(D3BJ)/def2-TZVPP
Symmetry: c1

Cartesian coordinates in Ångström:

C	0.3492789	-0.7339156	2.4450857
C	0.2220574	-2.0043767	1.9201392
C	-0.5662212	-2.9556608	2.5258139
H	-0.6516061	-3.9428650	2.0951824
C	-1.2363096	-2.5952842	3.6932552
H	-1.8622924	-3.3231636	4.1884599
C	-1.1080612	-1.3152165	4.2217333
H	-1.6345774	-1.0497869	5.1268273
C	-0.3061398	-0.3624845	3.5962787
H	-0.1930376	0.6387210	3.9862654
F	1.1570637	0.1381498	1.7639209
F	0.9143514	-2.2703219	0.7700871
C	1.4042196	-0.3393772	-4.0301685
H	1.2604984	-1.3336017	-4.4278695
C	1.3841677	0.7938617	-4.8408419
H	1.2207678	0.6803790	-5.9025871
C	1.5700655	2.0608142	-4.2980846

```
H     1.5507725    2.9303474   -4.9387262
C     1.7809906    2.2271353   -2.9306980
H     1.9252814    3.2011845   -2.4860533
C     1.7988189    1.0987800   -2.1435051
C     1.6140110   -0.1586478   -2.6825102
F     1.9987655    1.1680709   -0.7907760
F     1.6476956   -1.2153398   -1.8124125
Li    2.1171172   -0.7155633    0.1258886
H     4.5491284   -3.6147862    0.6805302
H     6.8936953   -4.4285833    1.0706622
H     3.9101588    0.8915301    2.3240852
C     5.3883458   -3.2327451    0.1004907
O     3.8367985   -1.0641343    0.5105700
H     6.2278491   -5.1242156   -0.4112192
C     6.5469577   -4.1966876    0.0646277
C     5.0069829   -1.0284732    0.8826800
O     5.8614637   -2.0106933    0.7433257
C     4.7613991    1.1805709    1.7078798
H     6.0070250    1.8209254    3.3433056
O     5.5755769   -0.0040270    1.4741710
H     5.0344924   -2.9729420   -0.8965171
C     5.6375661    2.1998817    2.3915319
H     5.0579885    3.1033698    2.5823812
H     7.3790195   -3.7866453   -0.5060297
H     4.3931976    1.5349836    0.7453003
H     6.4895472    2.4635734    1.7665600

SCF energy GEOOPT =  -1291.045740018 H
ZPE =   851.2 kJ/mol
FREEH energy =   921.80 kJ/mol
FREEH entropy =   0.83681 kJ/mol/K

$vibrational spectrum
#  mode     symmetry     wave number   IR intensity    selection rules
#                         cm**(-1)        km/mol         IR     RAMAN
     1                      -0.00         0.00000         -       -
     2                      -0.00         0.00000         -       -
     3                      -0.00         0.00000         -       -
     4                       0.00         0.00000         -       -
     5                       0.00         0.00000         -       -
     6                       0.00         0.00000         -       -
     7        a              5.72         0.01710        YES     YES
     8        a              7.53         0.05454        YES     YES
     9        a             11.13         0.33626        YES     YES
    10        a             12.27         0.10623        YES     YES
    11        a             17.61         0.00055        YES     YES
    12        a             34.94         0.04172        YES     YES
    13        a             40.28         0.01000        YES     YES
    14        a             55.81         0.74992        YES     YES
    15        a             57.86         0.04579        YES     YES
    16        a             64.71         0.17530        YES     YES
    17        a             72.53         5.20471        YES     YES
    18        a             75.36         0.07199        YES     YES
    19        a             81.51         0.08922        YES     YES
    20        a             86.53         0.39535        YES     YES
    21        a            123.30         7.05860        YES     YES
    22        a            137.68         0.38753        YES     YES
    23        a            161.86         0.00035        YES     YES
    24        a            197.81         0.05869        YES     YES
    25        a            199.94         0.01270        YES     YES
    26        a            233.84        19.30221        YES     YES
    27        a            256.39         8.91210        YES     YES
    28        a            257.33         0.72335        YES     YES
```

29	a	264.90	0.00324	YES	YES
30	a	288.98	1.07013	YES	YES
31	a	290.36	0.13286	YES	YES
32	a	323.59	3.02386	YES	YES
33	a	327.70	1.45648	YES	YES
34	a	347.13	0.24132	YES	YES
35	a	376.82	252.93738	YES	YES
36	a	386.78	21.82140	YES	YES
37	a	443.92	0.15236	YES	YES
38	a	448.45	1.85734	YES	YES
39	a	453.02	13.29880	YES	YES
40	a	453.19	0.30506	YES	YES
41	a	469.43	64.99860	YES	YES
42	a	557.53	0.01855	YES	YES
43	a	557.91	0.00392	YES	YES
44	a	557.99	0.27289	YES	YES
45	a	558.49	9.83269	YES	YES
46	a	571.86	86.63952	YES	YES
47	a	577.72	45.36183	YES	YES
48	a	580.21	48.18842	YES	YES
49	a	714.74	0.00044	YES	YES
50	a	715.10	0.00660	YES	YES
51	a	740.08	1.49003	YES	YES
52	a	770.93	129.15305	YES	YES
53	a	774.60	20.33790	YES	YES
54	a	775.11	39.72864	YES	YES
55	a	775.16	122.78866	YES	YES
56	a	804.87	25.63933	YES	YES
57	a	821.91	0.33451	YES	YES
58	a	829.44	2.03885	YES	YES
59	a	844.40	0.03693	YES	YES
60	a	848.63	17.38153	YES	YES
61	a	856.30	68.58210	YES	YES
62	a	875.16	0.00309	YES	YES
63	a	875.51	0.00312	YES	YES
64	a	926.21	0.71113	YES	YES
65	a	970.04	0.77487	YES	YES
66	a	970.21	5.91742	YES	YES
67	a	1015.22	0.00865	YES	YES
68	a	1015.26	0.02656	YES	YES
69	a	1022.34	63.68511	YES	YES
70	a	1024.62	70.59946	YES	YES
71	a	1042.84	8.17985	YES	YES
72	a	1042.91	1.40158	YES	YES
73	a	1114.39	0.09488	YES	YES
74	a	1118.92	45.89990	YES	YES
75	a	1134.18	0.20079	YES	YES
76	a	1137.57	2.84428	YES	YES
77	a	1173.24	0.60158	YES	YES
78	a	1174.94	1.44972	YES	YES
79	a	1178.91	0.04044	YES	YES
80	a	1181.67	10.81717	YES	YES
81	a	1183.48	20.99096	YES	YES
82	a	1184.58	7.41173	YES	YES
83	a	1184.69	1.21290	YES	YES
84	a	1266.62	293.46886	YES	YES
85	a	1273.19	61.89571	YES	YES
86	a	1294.28	0.02952	YES	YES
87	a	1294.62	0.02531	YES	YES
88	a	1303.10	0.63448	YES	YES
89	a	1305.80	2.52601	YES	YES
90	a	1341.32	13.32064	YES	YES
91	a	1341.64	6.03494	YES	YES

92	a	1346.51	674.78202	YES	YES
93	a	1400.02	16.19613	YES	YES
94	a	1424.63	77.92928	YES	YES
95	a	1430.47	16.37314	YES	YES
96	a	1468.45	245.47538	YES	YES
97	a	1486.23	1.54209	YES	YES
98	a	1486.41	13.97400	YES	YES
99	a	1499.09	5.27174	YES	YES
100	a	1500.00	8.02337	YES	YES
101	a	1500.28	0.01181	YES	YES
102	a	1500.77	11.86214	YES	YES
103	a	1518.09	36.86696	YES	YES
104	a	1524.43	30.93659	YES	YES
105	a	1531.09	332.56535	YES	YES
106	a	1534.27	60.87174	YES	YES
107	a	1635.44	0.01333	YES	YES
108	a	1637.86	3.16178	YES	YES
109	a	1663.74	19.20334	YES	YES
110	a	1664.20	8.79098	YES	YES
111	a	1712.85	566.40239	YES	YES
112	a	3049.75	7.29185	YES	YES
113	a	3049.87	4.52494	YES	YES
114	a	3054.92	17.63304	YES	YES
115	a	3060.54	17.79166	YES	YES
116	a	3097.19	6.42881	YES	YES
117	a	3101.53	4.68698	YES	YES
118	a	3117.90	8.61624	YES	YES
119	a	3118.04	13.44794	YES	YES
120	a	3130.04	11.28318	YES	YES
121	a	3130.59	29.61439	YES	YES
122	a	3198.23	0.58643	YES	YES
123	a	3198.25	1.21080	YES	YES
124	a	3206.77	2.91443	YES	YES
125	a	3206.80	1.16034	YES	YES
126	a	3212.94	1.21972	YES	YES
127	a	3212.97	1.74950	YES	YES
128	a	3218.35	0.19186	YES	YES
129	a	3218.36	0.15612	YES	YES

$end

Total COSMO energy + OC corr. = -1291.0999025069 H

[Li(DEC)$_2$(*o*-DFB)]$^+$ (F$_{o\text{-DFB}}$-coordination)

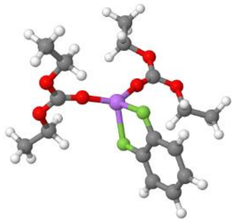

Method: (RI-)B3LYP(D3BJ)/def2-TZVPP
Symmetry: c1

Cartesian coordinates in Ångström:

Li	0.4559573	-0.5788186	-0.2940695
O	2.1436579	-1.2116489	-0.2887467
H	3.4706503	-5.0145963	-2.4901164
H	6.2807263	0.2572798	1.0264839
H	3.8136923	0.6017213	0.7058269
H	1.6913054	-3.5550084	-1.4880591

```
H     5.5093816    0.7150066    2.5493416
H     2.2410066   -4.6098465   -3.6932581
C     3.0322687   -4.2001768   -3.0650246
C     5.5720673   -0.0635086    1.7887165
C     3.2594188   -1.6915415   -0.4666675
C     4.2048695   -0.2900470    1.1946141
C     2.4517001   -3.1476031   -2.1543339
O     3.5412827   -2.6331605   -1.3366463
O     4.3382826   -1.3355927    0.1869751
H     3.7997859   -3.7764225   -3.7110881
H     5.9497830   -0.9714027    2.2568503
H     3.4824024   -0.6275293    1.9370892
H     2.0213914   -2.3146179   -2.7099363
O    -1.0240926   -0.9361000    0.6852292
H    -2.6234620   -4.0919819    3.6000720
H    -4.7520200    1.7339044    0.4863301
H    -2.3261810    1.2941877    0.0273020
H    -0.9117314   -3.2703855    1.9555897
H    -4.3995938    1.8911541   -1.2387856
H    -1.0143690   -4.0690186    4.3304504
C    -1.8096807   -3.4443474    3.9232087
C    -4.4458739    1.1893242   -0.4056245
C    -2.1132118   -1.0359453    1.2420521
C    -3.0909175    0.5562481   -0.2136531
C    -1.2683957   -2.6393547    2.7689667
O    -2.3640277   -1.8227436    2.2590158
O    -3.1939506   -0.3695199    0.9029392
H    -2.1749367   -2.7932298    4.7161142
H    -5.1998831    0.4366744   -0.6311981
H    -2.7745475   -0.0056779   -1.0920203
H    -0.4631906   -1.9709739    3.0721514
C    -0.2652370    3.1950738   -2.1094560
C    -0.0568107    1.8678808   -1.8084111
C    -0.8476833    3.5029679   -3.3371615
C    -0.9891400    1.1560183   -3.9078692
C    -0.4121491    0.8665068   -2.6918242
C    -1.2056700    2.4951238   -4.2260681
F     0.4989909    1.4872955   -0.6202802
F    -0.1776222   -0.4226200   -2.3047794
H    -1.0188962    4.5376719   -3.5960747
H    -1.6551766    2.7466474   -5.1756143
H    -1.2581469    0.3554601   -4.5819186
H     0.0222968    3.9601128   -1.4026139

SCF energy GEOOPT =  -1282.574239336 H
ZPE =    1029. kJ/mol
FREEH energy =  1109.36 kJ/mol
FREEH entropy =    0.92607 kJ/mol/K

$vibrational spectrum
#  mode     symmetry     wave number   IR intensity    selection rules
#                         cm**(-1)        km/mol          IR     RAMAN
     1                       -0.00         0.00000         -       -
     2                       -0.00         0.00000         -       -
     3                       -0.00         0.00000         -       -
     4                       -0.00         0.00000         -       -
     5                       -0.00         0.00000         -       -
     6                       -0.00         0.00000         -       -
     7         a              3.40         0.04920        YES     YES
     8         a              5.38         0.00647        YES     YES
     9         a              9.03         0.05690        YES     YES
    10         a             10.40         0.07160        YES     YES
    11         a             17.56         0.47272        YES     YES
```

12	a	29.74	0.04847	YES	YES
13	a	32.96	0.07012	YES	YES
14	a	42.21	0.00453	YES	YES
15	a	52.61	0.71172	YES	YES
16	a	57.94	0.48270	YES	YES
17	a	65.34	0.56557	YES	YES
18	a	65.67	1.65218	YES	YES
19	a	68.25	0.37710	YES	YES
20	a	80.74	1.12213	YES	YES
21	a	82.88	1.32633	YES	YES
22	a	84.40	1.36698	YES	YES
23	a	120.20	4.97174	YES	YES
24	a	121.12	2.04684	YES	YES
25	a	129.18	1.33546	YES	YES
26	a	156.20	3.06947	YES	YES
27	a	161.57	0.01242	YES	YES
28	a	162.49	0.00376	YES	YES
29	a	198.77	19.62303	YES	YES
30	a	200.34	7.85479	YES	YES
31	a	254.46	4.45679	YES	YES
32	a	255.36	7.32728	YES	YES
33	a	257.17	0.80952	YES	YES
34	a	257.38	1.05369	YES	YES
35	a	264.70	0.00383	YES	YES
36	a	265.12	0.01554	YES	YES
37	a	292.92	0.09345	YES	YES
38	a	319.66	5.00535	YES	YES
39	a	344.00	20.52397	YES	YES
40	a	346.78	0.32840	YES	YES
41	a	376.64	57.51543	YES	YES
42	a	386.89	20.43088	YES	YES
43	a	391.65	101.49709	YES	YES
44	a	445.28	0.23397	YES	YES
45	a	456.21	4.97492	YES	YES
46	a	487.96	26.33837	YES	YES
47	a	529.97	13.81798	YES	YES
48	a	556.68	4.72787	YES	YES
49	a	559.59	0.62673	YES	YES
50	a	579.24	19.67768	YES	YES
51	a	630.58	232.60452	YES	YES
52	a	716.31	0.00283	YES	YES
53	a	739.40	0.98976	YES	YES
54	a	739.85	1.46400	YES	YES
55	a	772.81	66.26538	YES	YES
56	a	776.82	84.25268	YES	YES
57	a	804.64	24.09425	YES	YES
58	a	805.20	22.30289	YES	YES
59	a	822.40	0.18177	YES	YES
60	a	822.91	0.73235	YES	YES
61	a	829.22	1.16199	YES	YES
62	a	831.80	0.00345	YES	YES
63	a	849.10	9.06158	YES	YES
64	a	857.51	61.36157	YES	YES
65	a	858.51	65.76779	YES	YES
66	a	876.79	0.00169	YES	YES
67	a	923.87	0.13382	YES	YES
68	a	928.62	2.46307	YES	YES
69	a	971.25	4.09854	YES	YES
70	a	1016.20	0.00778	YES	YES
71	a	1022.19	36.51481	YES	YES
72	a	1023.03	36.00913	YES	YES
73	a	1024.60	116.39260	YES	YES
74	a	1027.25	101.15582	YES	YES

75	a	1044.27	4.98357	YES	YES
76	a	1119.08	22.22985	YES	YES
77	a	1134.28	0.19517	YES	YES
78	a	1134.65	0.87418	YES	YES
79	a	1138.01	2.75831	YES	YES
80	a	1139.34	3.81165	YES	YES
81	a	1172.83	0.20429	YES	YES
82	a	1176.07	1.89803	YES	YES
83	a	1178.53	1.03171	YES	YES
84	a	1179.25	2.46502	YES	YES
85	a	1181.40	1.25028	YES	YES
86	a	1182.24	7.55249	YES	YES
87	a	1184.78	2.96851	YES	YES
88	a	1186.86	10.47027	YES	YES
89	a	1272.56	146.68171	YES	YES
90	a	1294.71	0.06159	YES	YES
91	a	1302.46	0.67647	YES	YES
92	a	1302.73	0.24998	YES	YES
93	a	1304.47	2.97894	YES	YES
94	a	1304.67	3.84515	YES	YES
95	a	1340.15	1.72332	YES	YES
96	a	1343.22	707.10437	YES	YES
97	a	1344.86	764.96240	YES	YES
98	a	1398.64	28.39606	YES	YES
99	a	1399.39	8.11926	YES	YES
100	a	1424.29	85.97207	YES	YES
101	a	1424.65	81.11403	YES	YES
102	a	1430.16	21.93552	YES	YES
103	a	1430.48	11.63752	YES	YES
104	a	1465.15	236.02934	YES	YES
105	a	1466.34	260.35816	YES	YES
106	a	1486.14	0.01367	YES	YES
107	a	1486.25	3.65969	YES	YES
108	a	1486.32	13.45835	YES	YES
109	a	1486.41	14.61678	YES	YES
110	a	1499.00	1.89460	YES	YES
111	a	1499.20	6.16340	YES	YES
112	a	1499.77	6.49348	YES	YES
113	a	1500.12	3.12950	YES	YES
114	a	1500.53	2.57913	YES	YES
115	a	1518.01	63.36529	YES	YES
116	a	1518.60	5.82848	YES	YES
117	a	1524.16	23.29772	YES	YES
118	a	1524.44	25.44845	YES	YES
119	a	1533.81	203.77269	YES	YES
120	a	1637.44	1.84239	YES	YES
121	a	1660.84	14.21299	YES	YES
122	a	1703.69	1016.87204	YES	YES
123	a	1727.66	131.92070	YES	YES
124	a	3049.56	6.53084	YES	YES
125	a	3049.77	7.12669	YES	YES
126	a	3049.83	5.90235	YES	YES
127	a	3049.90	4.33826	YES	YES
128	a	3053.74	18.55255	YES	YES
129	a	3055.72	23.54966	YES	YES
130	a	3057.95	22.65806	YES	YES
131	a	3058.79	20.88967	YES	YES
132	a	3095.73	6.31549	YES	YES
133	a	3098.78	2.50368	YES	YES
134	a	3099.16	6.18379	YES	YES
135	a	3099.96	6.13164	YES	YES
136	a	3117.19	13.97125	YES	YES
137	a	3117.94	6.25961	YES	YES

138	a	3118.00	11.60272	YES	YES
139	a	3118.03	16.46856	YES	YES
140	a	3129.70	6.52750	YES	YES
141	a	3130.04	33.25907	YES	YES
142	a	3130.32	12.85049	YES	YES
143	a	3130.90	22.03901	YES	YES
144	a	3197.57	0.93812	YES	YES
145	a	3205.55	1.96646	YES	YES
146	a	3211.55	1.44390	YES	YES
147	a	3217.49	0.16302	YES	YES

$end

Total COSMO energy + OC corr. = -1282.6296086911 H

Li(DME)(DEC)(*o*-DFB)]$^+$ (F$_{o\text{-DFB}}$-coordination)

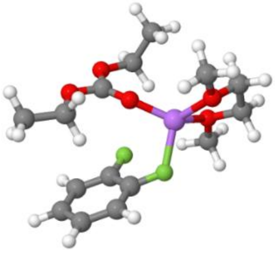

Method: (RI-)B3LYP(D3BJ)/def2-TZVPP
Symmetry: c1

Cartesian coordinates in Ångström:

C	0.6355825	2.1523634	-2.5903596
O	0.6358900	1.8753313	-1.1889445
Li	-0.4978187	0.3474602	-0.8743689
O	-0.4092964	0.0308730	-2.7959881
C	0.6587164	0.8292473	-3.3142552
C	0.6949519	3.0469448	-0.3739601
H	1.5154760	2.7412590	-2.8622030
H	-0.2623395	2.7230719	-2.8482477
C	-0.5407197	-1.2308027	-3.4579881
H	0.5258876	0.9882489	-4.3875415
H	1.6097521	0.3129489	-3.1505432
H	-1.3606822	-1.7600220	-2.9823304
H	0.3773499	-1.8138288	-3.3568045
H	-0.7625750	-1.0788199	-4.5155872
H	0.6594251	2.7196523	0.6612617
H	-0.1550456	3.7021114	-0.5792440
H	1.6252367	3.5875209	-0.5560816
H	1.2189855	-2.0476149	2.2281109
F	0.2708963	-1.3371694	-0.0966684
C	0.2132539	-2.3924291	2.0346833
C	-0.3912823	-2.0930997	0.8339228
H	-0.0455636	-3.3844487	3.9143522
C	-0.5036592	-3.1365368	2.9679945
C	-1.6754267	-2.5180686	0.5417688
F	-2.2184151	-2.1843934	-0.6474788
C	-1.7977818	-3.5593824	2.6852069
C	-2.3929977	-3.2517886	1.4653126
H	-2.3494067	-4.1364510	3.4131733
H	-3.3966549	-3.5702824	1.2222917
O	-1.9063747	0.6875558	0.2556345
H	-2.5804210	2.6973228	-1.4672753
H	-3.8284438	-0.9758178	4.0013474

```
H    -1.8409458   -0.4397278    2.5797701
H    -2.9924716    0.2650442   -1.9793732
H    -2.6091193   -0.1060907    4.9392632
H    -3.3417120    2.5069337   -3.0515775
C    -3.4881245    2.3787548   -1.9784312
C    -3.3131657   -0.0224924    4.1110939
C    -3.0934183    0.6220890    0.5602470
C    -2.5603468    0.3280585    2.8531437
C    -3.8041368    0.9281478   -1.6873417
O    -4.1004485    0.7153290   -0.2802608
O    -3.5410845    0.4411497    1.7793636
H    -4.3089809    3.0237656   -1.6681469
H    -4.0453910    0.7445257    4.3588066
H    -2.0422451    1.2832761    2.9330845
H    -4.7143985    0.6091410   -2.1869621

SCF energy GEOOPT =  -1169.203065369 H
ZPE =   1007. kJ/mol
FREEH energy =  1080.21 kJ/mol
FREEH entropy =   0.80648 kJ/mol/K

$vibrational spectrum
#  mode     symmetry     wave number   IR intensity    selection rules
#                         cm**(-1)        km/mol          IR     RAMAN
     1                      -0.00          0.00000         -        -
     2                      -0.00          0.00000         -        -
     3                       0.00          0.00000         -        -
     4                       0.00          0.00000         -        -
     5                       0.00          0.00000         -        -
     6                       0.00          0.00000         -        -
     7         a            18.27          0.05586        YES      YES
     8         a            24.22          0.14253        YES      YES
     9         a            29.37          1.28967        YES      YES
    10         a            34.46          0.81856        YES      YES
    11         a            39.15          0.53397        YES      YES
    12         a            41.13          0.34366        YES      YES
    13         a            44.02          0.14037        YES      YES
    14         a            53.23          2.00470        YES      YES
    15         a            57.83          0.96178        YES      YES
    16         a            63.98          0.61777        YES      YES
    17         a            81.51          1.36667        YES      YES
    18         a            85.34          0.49579        YES      YES
    19         a            94.93          0.85608        YES      YES
    20         a           105.55          3.69939        YES      YES
    21         a           126.16          3.02598        YES      YES
    22         a           135.38          0.83853        YES      YES
    23         a           147.75          1.13167        YES      YES
    24         a           158.16          2.77182        YES      YES
    25         a           165.27          0.34534        YES      YES
    26         a           176.96          0.70147        YES      YES
    27         a           206.55          0.36276        YES      YES
    28         a           216.94          1.60124        YES      YES
    29         a           223.78          0.44738        YES      YES
    30         a           230.63          2.64786        YES      YES
    31         a           261.53          0.72083        YES      YES
    32         a           281.27          2.14576        YES      YES
    33         a           292.23          0.48042        YES      YES
    34         a           305.19          3.90088        YES      YES
    35         a           327.47         28.66400        YES      YES
    36         a           328.84          1.92991        YES      YES
    37         a           344.12         34.41772        YES      YES
    38         a           362.18         25.61800        YES      YES
    39         a           374.25          7.43833        YES      YES
```

40	a	410.36	16.07533	YES	YES
41	a	430.51	39.02718	YES	YES
42	a	450.89	18.05693	YES	YES
43	a	464.12	3.99252	YES	YES
44	a	518.16	25.67569	YES	YES
45	a	551.27	4.20419	YES	YES
46	a	562.87	0.17093	YES	YES
47	a	577.94	7.13251	YES	YES
48	a	582.77	6.04218	YES	YES
49	a	589.95	162.37937	YES	YES
50	a	712.63	0.24488	YES	YES
51	a	718.70	0.01856	YES	YES
52	a	771.01	57.55831	YES	YES
53	a	778.83	85.93542	YES	YES
54	a	805.47	25.86094	YES	YES
55	a	816.78	1.33749	YES	YES
56	a	822.91	2.82682	YES	YES
57	a	843.59	8.19941	YES	YES
58	a	854.57	44.32971	YES	YES
59	a	855.72	20.64769	YES	YES
60	a	877.06	0.05549	YES	YES
61	a	879.19	54.95849	YES	YES
62	a	918.66	2.92781	YES	YES
63	a	970.45	3.60683	YES	YES
64	a	1005.87	19.10916	YES	YES
65	a	1014.78	0.08250	YES	YES
66	a	1023.15	104.63328	YES	YES
67	a	1026.82	8.08190	YES	YES
68	a	1042.22	12.06352	YES	YES
69	a	1048.43	5.23025	YES	YES
70	a	1093.23	270.18716	YES	YES
71	a	1111.34	18.10842	YES	YES
72	a	1122.37	20.99246	YES	YES
73	a	1123.86	28.07552	YES	YES
74	a	1134.84	37.17211	YES	YES
75	a	1136.78	2.04417	YES	YES
76	a	1152.32	3.08096	YES	YES
77	a	1180.18	5.40757	YES	YES
78	a	1181.21	4.12059	YES	YES
79	a	1184.08	3.61753	YES	YES
80	a	1187.50	4.12395	YES	YES
81	a	1192.71	21.36414	YES	YES
82	a	1207.82	16.97310	YES	YES
83	a	1215.30	18.37281	YES	YES
84	a	1233.86	0.29726	YES	YES
85	a	1268.03	10.68569	YES	YES
86	a	1279.08	69.39687	YES	YES
87	a	1295.04	4.67332	YES	YES
88	a	1302.74	9.11389	YES	YES
89	a	1304.83	4.54112	YES	YES
90	a	1325.93	8.56975	YES	YES
91	a	1338.16	1.57796	YES	YES
92	a	1344.35	652.87961	YES	YES
93	a	1397.63	12.21874	YES	YES
94	a	1403.46	13.24414	YES	YES
95	a	1418.18	41.50515	YES	YES
96	a	1426.37	45.97031	YES	YES
97	a	1439.34	3.40448	YES	YES
98	a	1456.35	183.37561	YES	YES
99	a	1485.03	0.94499	YES	YES
100	a	1485.94	7.79030	YES	YES
101	a	1486.39	4.46957	YES	YES
102	a	1487.62	2.74269	YES	YES

103	a	1490.34	23.78763	YES	YES
104	a	1494.62	15.87510	YES	YES
105	a	1497.39	3.24934	YES	YES
106	a	1499.00	13.59802	YES	YES
107	a	1499.58	9.94326	YES	YES
108	a	1502.67	5.59926	YES	YES
109	a	1504.10	4.91069	YES	YES
110	a	1514.75	27.80406	YES	YES
111	a	1517.05	11.97290	YES	YES
112	a	1519.13	6.41860	YES	YES
113	a	1524.29	39.98297	YES	YES
114	a	1538.58	200.38987	YES	YES
115	a	1637.61	1.31846	YES	YES
116	a	1657.11	17.74203	YES	YES
117	a	1712.18	394.36690	YES	YES
118	a	3013.03	16.01959	YES	YES
119	a	3016.42	13.03230	YES	YES
120	a	3022.41	35.38663	YES	YES
121	a	3025.39	30.31198	YES	YES
122	a	3046.16	8.18180	YES	YES
123	a	3049.26	5.64513	YES	YES
124	a	3059.52	23.78441	YES	YES
125	a	3068.37	10.55108	YES	YES
126	a	3068.71	32.48368	YES	YES
127	a	3086.77	19.73634	YES	YES
128	a	3087.50	9.87617	YES	YES
129	a	3088.80	18.53220	YES	YES
130	a	3111.56	14.45831	YES	YES
131	a	3115.23	1.58246	YES	YES
132	a	3117.43	8.11569	YES	YES
133	a	3117.47	10.59077	YES	YES
134	a	3139.42	17.09818	YES	YES
135	a	3145.03	10.43058	YES	YES
136	a	3146.58	16.80161	YES	YES
137	a	3156.16	6.03397	YES	YES
138	a	3195.56	0.86296	YES	YES
139	a	3203.53	2.15556	YES	YES
140	a	3209.69	0.78219	YES	YES
141	a	3215.99	0.03787	YES	YES

$end

Total COSMO energy + OC corr. = -1169.2583263620 H

(*o*-DFB)Li[Ga(C_2F_5)$_4$] ($F_{o\text{-DFB}}$-coordination)

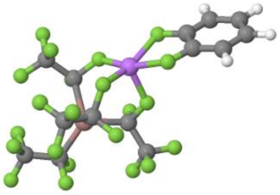

•

Method: (RI-)B3LYP(D3BJ)/def2-TZVPP
Symmetry: c1

Cartesian coordinates in Ångström:

F	1.7219563	3.2876835	-2.7231595
F	3.6346770	3.5902106	-1.7436362
F	-2.7490132	4.0458981	-0.9520971
C	2.3096458	3.5446749	-1.5507381
F	2.6311731	1.3580272	-0.8154421

```
F     1.9143563    4.7575850   -1.1319323
C     1.9174049    2.4705295   -0.5198482
C    -1.6639705    4.6620848   -0.4702921
F    -2.0468708    5.8223413    0.0816231
F    -1.8603235    3.5376362    1.5530432
F     0.2746215    0.8730450   -3.2852436
C    -0.9736374    3.7379171    0.5434632
Ga   -0.0617489    1.9780147   -0.0871763
F    -0.8410684    4.9367873   -1.4907197
F     2.4955982    2.9658249    0.7047333
F    -0.7249758   -0.5310917   -1.2107949
C    -0.9860238    1.0801750   -2.8744865
C    -1.1026701    0.7779745   -1.3722663
F     0.0605931    4.5421155    1.1340721
F    -1.7972092    0.3160418   -3.6127922
F    -1.2960920    2.3678972   -3.1066726
F    -2.4386406    0.8471780   -1.0573521
F    -1.9106908    0.9174521    2.5703731
F     0.6054139    2.0733594    2.6925110
C    -0.7869851    0.2022353    2.4353219
F    -0.4033579   -0.2040932    3.6565002
C     0.3020362    1.0354689    1.7413935
F    -1.0556968   -0.8798436    1.6987603
F     1.4227277    0.2674345    1.7101387
Li    1.4690336    3.7004425    2.1585140
C     1.2775304    4.7438387    6.2595042
C     1.6552976    4.4471304    4.9697272
C     2.1648107    4.4290823    7.2864902
C     3.7590831    3.5373984    5.6992054
C     2.8727723    3.8551657    4.6957316
C     3.3905664    3.8332502    7.0098811
F     0.8478649    4.7162432    3.9063506
F     3.1510072    3.5956877    3.3857324
H     1.8906536    4.6504289    8.3077709
H     4.0678369    3.5921827    7.8163113
H     4.7027674    3.0706818    5.4569667
H     0.3176372    5.2025635    6.4472208

SCF energy GEOOPT =  -4665.000574912 H
ZPE =   497.5 kJ/mol
FREEH energy =   600.17 kJ/mol
FREEH entropy =   1.04185 kJ/mol/K

$vibrational spectrum
#  mode     symmetry     wave number   IR intensity    selection rules
#                         cm**(-1)        km/mol          IR     RAMAN
     1                      -0.00         0.00000         -        -
     2                      -0.00         0.00000         -        -
     3                       0.00         0.00000         -        -
     4                       0.00         0.00000         -        -
     5                       0.00         0.00000         -        -
     6                       0.00         0.00000         -        -
     7        a              7.95         0.74909        YES      YES
     8        a             17.36         0.02793        YES      YES
     9        a             18.52         0.16406        YES      YES
    10        a             24.64         0.04753        YES      YES
    11        a             29.19         0.06870        YES      YES
    12        a             36.42         0.03498        YES      YES
    13        a             44.71         0.08980        YES      YES
    14        a             46.72         0.47786        YES      YES
    15        a             50.52         0.05519        YES      YES
    16        a             55.10         0.31057        YES      YES
    17        a             58.86         0.22776        YES      YES
```

18	a	64.29	0.16407	YES	YES
19	a	66.70	0.03970	YES	YES
20	a	70.13	0.65097	YES	YES
21	a	71.35	0.28905	YES	YES
22	a	76.99	0.15319	YES	YES
23	a	84.87	0.36646	YES	YES
24	a	86.22	1.11152	YES	YES
25	a	95.33	0.52375	YES	YES
26	a	106.92	0.34637	YES	YES
27	a	113.32	1.39884	YES	YES
28	a	124.78	0.76881	YES	YES
29	a	145.59	2.45742	YES	YES
30	a	182.19	0.07269	YES	YES
31	a	193.98	1.24044	YES	YES
32	a	195.98	0.17609	YES	YES
33	a	201.20	0.01551	YES	YES
34	a	208.30	0.53438	YES	YES
35	a	214.19	1.43806	YES	YES
36	a	219.42	1.86513	YES	YES
37	a	223.81	0.56863	YES	YES
38	a	228.42	4.34248	YES	YES
39	a	241.19	10.02398	YES	YES
40	a	251.08	2.11440	YES	YES
41	a	274.29	1.95753	YES	YES
42	a	277.11	0.80373	YES	YES
43	a	282.67	6.66648	YES	YES
44	a	292.27	3.25674	YES	YES
45	a	294.46	6.71816	YES	YES
46	a	298.54	8.73050	YES	YES
47	a	321.77	103.77774	YES	YES
48	a	325.14	16.28571	YES	YES
49	a	348.54	33.04930	YES	YES
50	a	351.63	17.90870	YES	YES
51	a	363.13	6.02418	YES	YES
52	a	364.51	0.47534	YES	YES
53	a	373.68	20.63503	YES	YES
54	a	382.37	34.07299	YES	YES
55	a	408.19	155.05948	YES	YES
56	a	425.86	1.78379	YES	YES
57	a	431.09	6.52348	YES	YES
58	a	438.34	69.55039	YES	YES
59	a	439.48	16.43357	YES	YES
60	a	447.04	8.70753	YES	YES
61	a	454.19	3.86517	YES	YES
62	a	522.59	1.20291	YES	YES
63	a	524.03	7.32737	YES	YES
64	a	524.64	1.39560	YES	YES
65	a	529.24	1.44818	YES	YES
66	a	559.14	3.06165	YES	YES
67	a	561.76	1.70035	YES	YES
68	a	576.85	5.53202	YES	YES
69	a	578.14	3.10925	YES	YES
70	a	579.06	34.89074	YES	YES
71	a	581.71	9.64191	YES	YES
72	a	582.85	6.40372	YES	YES
73	a	584.00	0.95199	YES	YES
74	a	590.49	0.89458	YES	YES
75	a	593.32	20.47347	YES	YES
76	a	600.35	1.40934	YES	YES
77	a	715.04	0.00085	YES	YES
78	a	727.00	41.15302	YES	YES
79	a	728.09	14.90448	YES	YES
80	a	731.84	23.30586	YES	YES

81	a	734.73	13.78403	YES	YES
82	a	771.18	77.95368	YES	YES
83	a	777.91	69.27361	YES	YES
84	a	851.30	18.18271	YES	YES
85	a	871.98	0.15966	YES	YES
86	a	883.47	25.45198	YES	YES
87	a	893.07	56.15650	YES	YES
88	a	920.14	269.49209	YES	YES
89	a	934.62	99.41384	YES	YES
90	a	945.42	27.80705	YES	YES
91	a	946.58	44.64436	YES	YES
92	a	948.71	63.65033	YES	YES
93	a	963.94	4.07068	YES	YES
94	a	1000.97	0.01030	YES	YES
95	a	1043.01	5.48540	YES	YES
96	a	1051.32	71.52883	YES	YES
97	a	1108.27	114.46983	YES	YES
98	a	1119.63	11.22162	YES	YES
99	a	1122.27	58.56609	YES	YES
100	a	1129.89	118.01413	YES	YES
101	a	1142.97	158.25426	YES	YES
102	a	1171.25	179.54810	YES	YES
103	a	1175.21	335.65648	YES	YES
104	a	1182.14	11.33635	YES	YES
105	a	1182.68	219.91656	YES	YES
106	a	1185.82	176.18557	YES	YES
107	a	1188.78	141.08432	YES	YES
108	a	1196.30	176.39452	YES	YES
109	a	1203.47	395.15107	YES	YES
110	a	1204.44	116.19260	YES	YES
111	a	1210.40	127.45760	YES	YES
112	a	1277.48	149.88488	YES	YES
113	a	1283.71	385.62525	YES	YES
114	a	1294.88	0.32502	YES	YES
115	a	1295.09	178.21862	YES	YES
116	a	1298.09	161.53832	YES	YES
117	a	1312.04	58.90545	YES	YES
118	a	1343.03	7.45048	YES	YES
119	a	1500.53	8.85644	YES	YES
120	a	1536.00	193.12800	YES	YES
121	a	1640.96	2.25032	YES	YES
122	a	1662.81	13.53013	YES	YES
123	a	3194.27	1.43613	YES	YES
124	a	3205.16	4.19468	YES	YES
125	a	3213.04	0.52761	YES	YES
126	a	3217.11	0.04714	YES	YES

$end

Total COSMO energy + OC corr. = -4665.0152713695 H

[Li(*o*-DFB)(DME)$_2$]$^+$ (F$_{o\text{-DFB}}$-coordination)

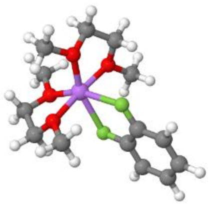

Method: (RI-)B3LYP(D3BJ)/def2-TZVPP
Symmetry: c2

```
Cartesian coordinates in Ångström:
C     0.2826806    2.7900721   -0.9457072
O     1.1261376    1.7016660   -0.5711428
Li    0.0000000   -0.0000000   -0.6511612
O    -1.1771891    1.1212734   -1.7776701
C    -0.5390630    2.3450867   -2.1293283
C     2.0818061    2.0499272    0.4282087
H     0.8848065    3.6607491   -1.2200708
H    -0.3619785    3.0596409   -0.1035147
C    -2.0427401    0.6197853   -2.7924325
H    -1.2833802    3.1084748   -2.3737393
H     0.0984099    2.1869425   -3.0058712
H    -2.4418343   -0.3230932   -2.4312103
H    -1.4915816    0.4529519   -3.7224530
H    -2.8584281    1.3211280   -2.9793920
H     2.6741892    1.1622626    0.6293352
H     1.5843714    2.3740425    1.3461756
H     2.7319102    2.8505872    0.0696746
H     1.9647037   -1.5181199    3.6399246
F     1.0534533   -0.8156367    1.2869883
C     1.1094962   -0.8579697    3.6667106
C     0.5468194   -0.4235904    2.4852943
H     0.9785695   -0.7563157    5.8005478
C     0.5500224   -0.4252769    4.8657753
C    -0.5468194    0.4235904    2.4852943
F    -1.0534533    0.8156367    1.2869883
C    -0.5500224    0.4252769    4.8657753
C    -1.1094962    0.8579697    3.6667106
H    -0.9785695    0.7563157    5.8005478
H    -1.9647037    1.5181199    3.6399246
H    -1.5843714   -2.3740425    1.3461756
H     0.3619785   -3.0596409   -0.1035147
C    -2.0818061   -2.0499272    0.4282087
C    -0.2826806   -2.7900721   -0.9457072
O    -1.1261376   -1.7016660   -0.5711428
H    -2.7319102   -2.8505872    0.0696746
H    -2.6741892   -1.1622626    0.6293352
H    -0.8848065   -3.6607491   -1.2200708
H     1.2833802   -3.1084748   -2.3737393
O     1.1771891   -1.1212734   -1.7776701
C     0.5390630   -2.3450867   -2.1293283
H     2.8584281   -1.3211280   -2.9793920
C     2.0427401   -0.6197853   -2.7924325
H     2.4418343    0.3230932   -2.4312103
H    -0.0984099   -2.1869425   -3.0058712
H     1.4915816   -0.4529519   -3.7224530

SCF energy GEOOPT =  -1055.827640320 H
ZPE =   980.8 kJ/mol
FREEH energy =  1048.83 kJ/mol
FREEH entropy =   0.74739 kJ/mol/K

$vibrational spectrum
#  mode     symmetry     wave number   IR intensity    selection rules
#                         cm**(-1)        km/mol          IR      RAMAN
     1                        0.00         0.00000         -        -
     2                        0.00         0.00000         -        -
     3                        0.00         0.00000         -        -
     4                        0.00         0.00000         -        -
     5                        0.00         0.00000         -        -
     6                        0.00         0.00000         -        -
     7         b             10.81         0.87835        YES      YES
     8         b             30.55         0.07475        YES      YES
```

9	a	40.96	0.00002	YES	YES
10	b	43.47	2.00120	YES	YES
11	a	45.11	0.75751	YES	YES
12	a	52.76	0.34108	YES	YES
13	a	67.28	2.25001	YES	YES
14	b	68.95	0.09290	YES	YES
15	b	82.37	1.22359	YES	YES
16	a	85.25	0.70572	YES	YES
17	b	105.09	6.69191	YES	YES
18	b	113.13	3.28944	YES	YES
19	a	116.84	0.12297	YES	YES
20	b	148.03	2.00872	YES	YES
21	a	150.27	0.46707	YES	YES
22	a	152.09	6.60582	YES	YES
23	a	179.39	0.42328	YES	YES
24	b	181.74	0.26577	YES	YES
25	a	203.70	0.06800	YES	YES
26	b	218.68	0.13814	YES	YES
27	a	225.23	0.53564	YES	YES
28	b	228.39	4.18202	YES	YES
29	a	236.52	2.41187	YES	YES
30	b	286.55	2.61272	YES	YES
31	a	291.84	0.04874	YES	YES
32	b	296.05	0.27006	YES	YES
33	a	297.37	29.81072	YES	YES
34	a	334.41	40.65473	YES	YES
35	b	336.71	28.21441	YES	YES
36	b	350.58	8.29165	YES	YES
37	a	353.95	19.28708	YES	YES
38	b	364.44	24.57844	YES	YES
39	a	388.98	57.80464	YES	YES
40	b	445.19	0.13940	YES	YES
41	b	461.17	5.22023	YES	YES
42	b	470.32	109.30070	YES	YES
43	b	555.23	5.71841	YES	YES
44	a	561.37	0.00150	YES	YES
45	a	576.92	4.71512	YES	YES
46	b	578.52	2.82680	YES	YES
47	a	581.70	11.02817	YES	YES
48	a	718.24	0.00560	YES	YES
49	a	774.07	64.71180	YES	YES
50	b	776.70	75.34010	YES	YES
51	a	847.01	10.10882	YES	YES
52	b	849.51	0.60238	YES	YES
53	b	854.65	17.39466	YES	YES
54	a	876.04	0.00049	YES	YES
55	b	877.93	80.98727	YES	YES
56	a	885.71	14.05108	YES	YES
57	b	969.52	3.03565	YES	YES
58	a	1012.67	0.00000	YES	YES
59	b	1028.82	23.97336	YES	YES
60	a	1032.88	2.89881	YES	YES
61	a	1044.54	23.60909	YES	YES
62	b	1045.09	4.81205	YES	YES
63	a	1047.10	3.73128	YES	YES
64	a	1102.05	217.54775	YES	YES
65	b	1103.15	304.79405	YES	YES
66	b	1123.20	22.50986	YES	YES
67	a	1128.24	4.41362	YES	YES
68	b	1128.84	10.86832	YES	YES
69	a	1139.17	3.35345	YES	YES
70	b	1140.52	104.37036	YES	YES
71	b	1181.87	8.10836	YES	YES

72	a	1182.12	0.09940	YES	YES
73	a	1184.25	2.53565	YES	YES
74	b	1188.01	6.02207	YES	YES
75	a	1188.11	0.19558	YES	YES
76	b	1194.63	14.58124	YES	YES
77	a	1215.86	3.65766	YES	YES
78	b	1215.88	28.73902	YES	YES
79	b	1233.95	2.02656	YES	YES
80	a	1234.02	0.00765	YES	YES
81	a	1269.10	0.79490	YES	YES
82	b	1269.99	24.53386	YES	YES
83	a	1280.34	162.00354	YES	YES
84	b	1294.04	0.43078	YES	YES
85	b	1304.12	14.37355	YES	YES
86	a	1304.70	1.00837	YES	YES
87	a	1338.62	1.75213	YES	YES
88	a	1402.98	13.24318	YES	YES
89	b	1405.32	15.26023	YES	YES
90	b	1439.98	4.26533	YES	YES
91	a	1440.39	0.58124	YES	YES
92	a	1479.58	0.01765	YES	YES
93	b	1480.44	2.34804	YES	YES
94	a	1483.54	0.61668	YES	YES
95	b	1483.96	1.61339	YES	YES
96	b	1493.71	1.84236	YES	YES
97	a	1493.83	16.25185	YES	YES
98	b	1496.84	41.50542	YES	YES
99	a	1497.76	2.16494	YES	YES
100	b	1499.06	6.09274	YES	YES
101	a	1501.10	1.52803	YES	YES
102	b	1502.49	3.78550	YES	YES
103	a	1503.56	0.87045	YES	YES
104	b	1504.87	20.64550	YES	YES
105	b	1515.99	0.17978	YES	YES
106	a	1517.95	1.75731	YES	YES
107	b	1518.39	18.20687	YES	YES
108	a	1521.33	8.70973	YES	YES
109	a	1537.65	191.26019	YES	YES
110	b	1639.58	0.91672	YES	YES
111	a	1657.44	14.94495	YES	YES
112	a	3004.61	3.45578	YES	YES
113	b	3005.43	25.29933	YES	YES
114	b	3011.98	9.30062	YES	YES
115	a	3013.19	54.83591	YES	YES
116	b	3013.40	14.04401	YES	YES
117	a	3013.79	52.10768	YES	YES
118	b	3018.54	37.39803	YES	YES
119	a	3018.93	27.15923	YES	YES
120	b	3050.38	59.25579	YES	YES
121	a	3050.70	1.46973	YES	YES
122	b	3062.54	48.48304	YES	YES
123	a	3062.69	37.59627	YES	YES
124	b	3073.51	36.35284	YES	YES
125	a	3074.20	1.36724	YES	YES
126	b	3076.78	29.19329	YES	YES
127	a	3077.01	22.48680	YES	YES
128	a	3147.79	0.92744	YES	YES
129	b	3147.83	14.74633	YES	YES
130	a	3151.35	1.23327	YES	YES
131	b	3151.49	14.51870	YES	YES
132	b	3195.57	0.79053	YES	YES
133	a	3203.02	2.76613	YES	YES
134	b	3209.18	0.28328	YES	YES

```
   135        a            3216.05          0.00106       YES     YES
$end
```

Total COSMO energy + OC corr. = -1055.8810830797 H

References

[1] E. O. Stejskal, J. E. Tanner, *J. Chem. Phys.* **1965**, *42*, 288.

[2] A. Hockmann, F. Ackermann, D. Diddens, I. Cekic-Laskovic, M. Schönhoff, *Faraday Discuss.* **2024**, *253*, 343.

[3] a) R. Ahlrichs, M. Bär, M. Häser, H. Horn, C. Kölmel, *Chem. Phys. Lett.* **1989**, *162*, 165; b) M. von Arnim, R. Ahlrichs, *J. Comput. Chem.* **1998**, *19*, 1746; c) K. Eichkorn, O. Treutler, H. Öhm, M. Häser, R. Ahlrichs, *Chem. Phys. Lett.* **1995**, *242*, 652; d) A. Schäfer, H. Horn, R. Ahlrichs, *J. Chem. Phys.* **1992**, *97*, 2571; e) P. Deglmann, F. Furche, *J. Chem. Phys.* **2002**, *117*, 9535.

[4] O. Treutler, R. Ahlrichs, *J. Chem. Phys.* **1995**, *102*, 346.

[5] A. D. Becke, *Phys. Rev. A* **1988**, *38*, 3098.

[6] J. P. Perdew, *Phys. Rev. B: Condens.* **1986**, *33*, 8822.

[7] J. P. Perdew, *Phys. Rev. B: Condens.* **1986**, *34*, 7406.

[8] P. Deglmann, F. Furche, R. Ahlrichs, *Chem. Phys. Lett.* **2002**, *362*, 511.

[9] a) R. Ahlrichs, *Phys. Chem. Chem. Phys.* **2004**, *6*, 5119; b) M. Sierka, A. Hogekamp, R. Ahlrichs, *J. Chem. Phys.* **2003**, *118*, 9136; c) F. Weigend, *Phys. Chem. Chem. Phys.* **2006**, *8*, 1057.

[10] a) S. Grimme, J. Antony, S. Ehrlich, H. Krieg, *J. Chem. Phys.* **2010**, *132*, 154104; b) S. Grimme, S. Ehrlich, L. Goerigk, *J. Comput. Chem.* **2011**, *32*, 1456.

[11] a) A. D. Becke, *J. Chem. Phys.* **1993**, *98*, 1372; b) A. D. Becke, *J. Chem. Phys.* **1993**, *98*, 5648; c) C. Lee, W. Yang, R. G. Parr, *Phys. Rev. B: Condens. Matter* **1988**, *37*, 785.

[12] F. Weigend, R. Ahlrichs, *Phys. Chem. Chem. Phys.* **2005**, *7*, 3297.

[13] A. Klamt, G. Schüürmann, *Perkin Trans. 2* **1993**, 799.

[14] W. M. Haynes (Ed.), *CRC handbook of chemistry and physics // CRC Handbook of chemistry and physics*. *A ready-reference book of chemical and physical data,* 91. Aufl., CRC Press, Boca Raton, Fla., **2017**.

[15] J. Self, N. T. Hahn, K. A. Persson, *Energy Environ. Mater.* **2024**, *7*, e12494.

[16] M. Ligare, *Am. J. Phys.* **2010**, *78*, 815.
